# Contribution of Transcription Factor Binding Site Motif Variants to Condition-Specific Gene Expression Patterns in Budding Yeast

**DOI:** 10.1371/journal.pone.0032274

**Published:** 2012-02-23

**Authors:** Joshua S. Rest, Kevin Bullaughey, Geoffrey P. Morris, Wen-Hsiung Li

**Affiliations:** Department of Ecology and Evolution, The University of Chicago, Chicago, Illinois, United States of America; University of Wyoming, United States of America

## Abstract

It is now experimentally well known that variant sequences of a *cis* transcription factor binding site motif can contribute to differential regulation of genes. We characterize the relationship between motif variants and gene expression by analyzing expression microarray data and binding site predictions. To accomplish this, we statistically detect motif variants with effects that differ among environments. Such environmental specificity may be due to either affinity differences between variants or, more likely, differential interactions of TFs bound to these variants with cofactors, and with differential presence of cofactors across environments. We examine conservation of functional variants across four *Saccharomyces* species, and find that about a third of transcription factors have target genes that are differentially expressed in a condition-specific manner that is correlated with the nucleotide at variant motif positions. We find good correspondence between our results and some cases in the experimental literature (Reb1, Sum1, Mcm1, and Rap1). These results and growing consensus in the literature indicates that motif variants may often be functionally distinct, that this may be observed in genomic data, and that variants play an important role in condition-specific gene regulation.

## Introduction

Transcription of genes into mRNA is mediated by transcription factor (TF) binding sites in upstream promoter and enhancer sequences. Mutations in these promoter sequences therefore affect gene regulation and may contribute to pathogenesis or evolution [1], [2], [3], [4], [5], [6], [7], [8], [9], [10]. Different types of promoter variation are rapidly being explored, including heterozygous variation between promoter copies resulting in allele-specific expression [11], [12], complete gain and loss of regulatory function by single nucleotide substitutions [13], [14], and differences in binding properties among binding site motif variants (BSMVs) that promote differential interactions with co-activators [15]. The observation that BSMVs from co-associating sites in the genome often co-vary with each other to maintain function has led to a method for discovering binding sites by searching for correlated SNPs 1–2 kb apart among individuals [16], [17]. Promoter variation is an important source of data that will aid understanding the encoding of regulatory function in promoter and other regulatory sequences. The function of several promoters has now been modeled computationally [18], [19]. However, predicting the activity of promoters on a genome-wide scale will require a sophisticated understanding of the functional effect of BSMVs, the interaction of bound TFs with dynamically changing cofactors, the combinatorial interactions between these sites, and with other epigenetic factors.

Functional BSMVs have been shown to be important in promoting condition-specific activity of transcription factors. BSMVs that have different rates of occupancy (or affinity) by a TF can result in differential gene expression [20], [21], [22], [23], [24]. McCord *et al.* [25] showed a predictive relationship between binding site affinity for many TFs and condition specific differential expression using genome-wide expression data. Ordered binding affinities can explain linear chains of activation, shutoff, or synchronization in dynamic pathways [26]. Differential affinity has been shown to act in coordination with higher order chromatin modifications [27] and methylation [15]. Computational and data mining approaches to learn these patterns from genomic sequence and expression data will be an important approach for elucidating cases and principles where BSMVs contribute to functionality. For example, Michal *et al.* [28] showed that sets of short sequences from promoters can be grouped together according to the expression of associated genes, and that single mutations between these sequence groups are related to known functionally-relevant BSMVs.

One common assumption is that affinity differences between alternative nucleotides provide the biological basis for BSMVs, yet the explanatory power of affinity differences alone is relatively weak. Further, the experimental literature suggests that the mechanisms by which BSMVs mediate differential expression are far more complex. In this more complex class of studied cases, BSMVs cause the bound TF to adopt different conformations, directing interactions with specific cofactors [15], [29], [30], [31], [32], [33], [34], [35]; these have been termed allosteric regulators [36]. For example, in the mouse, a single nucleotide difference within the Pit-1 TF binding site determines activation or repression of growth hormone in different cell–types of the posterior pituitary caused by different conformations of the DNA binding domain when it sits on alternative BSMVs [37]. Variation in interaction with sets of cofactors represents a more realistic view of the combinatorial nature of cellular interactions, where the presence or absence of an effected cofactor in different conditions determines whether the BSMV will actually cause a functional effect [38]. Differential ability to interact with cofactors (including repressors versus activators) or TFs bound at cognate binding sites may be a primary basis for regulatory differentiation of BSMVs. For example, the energetics and orientation of the Jun-Fos heterodimer when bound to DNA is altered by single nucleotide variants of the TGACTCA binding site motif [30]. These BSMVs cause differential regulation both among genes and among individuals.

BSMVs may only have simple binding affinity differences for TFs, where one variant is a “higher quality” binding site and has a higher occupancy or recruitment rate [25], [26], [27]. Such BSMVs would not be directly causal of complete differences in regulatory activity among their respectively regulated genes. For example, an activating TF at low concentrations may drive higher expression of genes with a high affinity BSMV than genes with a low affinity BSMV. At high enough concentrations of the TF, both high and low affinity BSMVs may be fully occupied, and the expression level of genes with both BSMVs would be the same. Even if expression levels are not comparable between genes, the steepness of the TF concentration-gene expression response curve will vary between BSMVs with different affinities.

In contrast, a proportion of BSMVs that are affected by allosteric effects are expected to show a regulatory impact that is, at the extreme, completely reversed between BSMVs. The most obvious examples are BSMVs that switch between activators and repressors, depending on the presence of cofactors [39]. Such reversals require condition-specific cofactors that are responsible for the differential function of the BSMV across conditions. A condition-specific cofactor may bind differentially to the TF depending on the TF conformation induced by the specific BSMV of the motif [36]. The result is that expression is both BSMV-specific and condition-specific, dependent upon the presence or the activity of the cofactor across conditions. While the regulatory effects of BSMVs that differ in affinity is never expected to be reversed, by searching for BSMVs that are associated with opposite regulatory effects, we propose to identify BSMVs whose action is due to more complex interactions than affinity alone. We applied a statistic focused on detecting instances where the relative expression levels of target genes with distinct BSMVs are maximally different between conditions. We use this statistic to assess the minimum contribution of allosteric interactions to the function of BSMVs, to identify novel candidates for further investigation, and to assess the contribution of these more complex regulatory types to the evolution of regulatory systems.

## Results

We tested whether changes in gene expression patterns can be attributed to functional BSMVs by comparing distances between pairs of expression profiles associated with each nucleotide variant at each position of a binding site, where the expression of each gene is ranked across different experimental conditions. We emphasize that BSMVs discussed here are considered only at a single motif position at a time, and the variation in the motif is observed at different promoters in the same genome (as opposed to, for example, population-level variation). Specifically, the effect size was calculated from the average difference between BSMVs in the ranking of expression values for genes controlled by those BSMVs across experiments. We call this metric the variant distance of ranked experiments (VDRE). For each variable TF motif position, VDRE subdivides genes into groups based on the nucleotide at that position in the binding site of the gene's promoter. All variants are simultaneously considered, resulting in a maximum of four BSMV groupings, one for each of the four nucleotides. Significance is measured by comparing within- versus between- BSMV distributions of VDRE with a distribution based on permuted data. The largest effect size in VDRE would occur if the relative ranking of gene expression across conditions is exactly reversed between BSMVs.

The gene expression data used in the VDRE analysis was obtained from 211 published Affymetrix S98 expression microarrays from *Saccharomyces cerevisiae*. The BSMVs were obtained from genome-wide binding site annotations for 77 TFs in *S. cerevisiae*, derived by computationally scanning the genome with motif models based on ChIP-chip binding assays, conservation and motif overrepresentation [40]. We divided putative binding sites into a primary (high probability) set and a secondary (low probability) set. We considered only target genes with a single primary binding site. This allowed us to consider 195 variable positions (each with two or more BSMVs) from 48 TF binding motifs.

**Figure 1:**
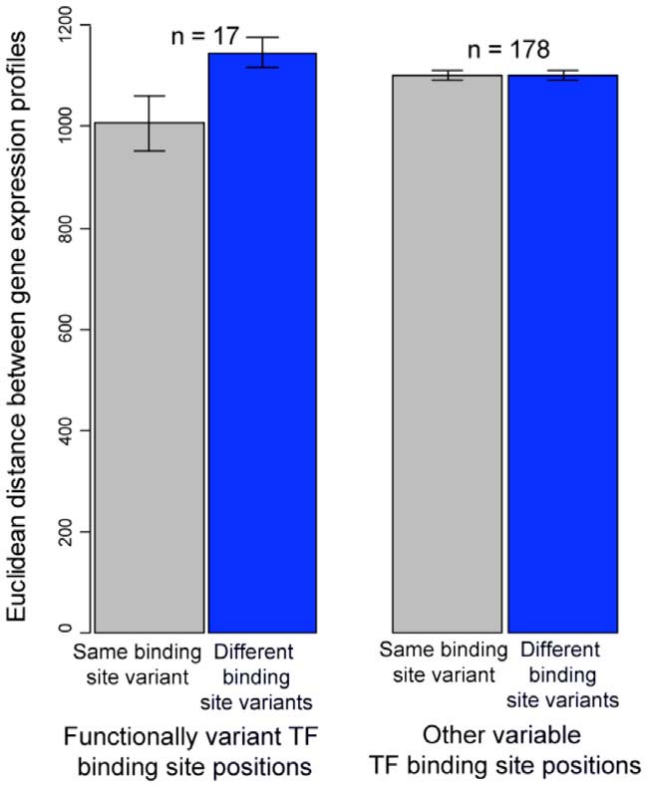
The expression profile distance between genes with the same binding site motif variant (BSMV) is smaller than the distance between genes with a different BSMV for functionally variant positions, but not for other positions with BSMVs. VDRE distances are based on ranked expression profiles for 211 S98 Affymetrix microarrays, and all within-BSMV (grey) or between BSMV (blue) distances are grouped together either from all functionally variant binding site positions (first two bars) or all other positions with BSMVs (third and fourth bar).

Using this data set with VDRE, we found that ∼29% of TF binding motifs have functional BSMVs (Table 1; Table S1; Fig. S1). In total, we identified 9% (17/195) of the motif positions as functionally variant (p<0.05) across the conditions surveyed in this study at a false discovery rate of 0.3, suggesting that ∼12/17 functional BSMVs are true positives. As expected, average distance in expression profile between genes with the same BSMV is significantly smaller than the distance between genes with a different BSMV for functionally variant positions, but not for other variable binding site positions (Fig. 1). In our analysis, only genes with a single primary input are considered; however, if additional target genes with multiple primary inputs are also included, some functional BSMVs are still detected, even though complex regulation was not considered (Fig. S2; Table S2).

**Table 1:** Quantities of functional binding site motif variants (BSMVs) discovered among datasets.

Species (array data)	# of BSMs^a^ with functional BSMVs	# of BSMs considered	% of BSMs	# of positions with functional BSMVs^b^	# of positions considered	% of positions
*S. cerevisiae* (Affymetrix)	14	48	29%	17	195	9%
*S. cerevisiae* (cDNA)	11	31	36%	13	112	12%
*S. paradoxus* (cDNA)	10	33	30%	13	119	11%
*S. mikatae* (cDNA)	11	33	33%	12	126	10%
*S. kudriavzevii* (cDNA)	13	33	39%	16	126	13%

a Binding site motifs ^b^p-value<0.05, false discovery rate = 0.3.

We further tested the functional BSMVs identified according to the VDRE statistic (single primary inputs only) to see if they display reversal of their regulatory effects between different experiments—that is, whether their rankings of expression across conditions are reversed between BSMVs. We tested all pairwise combinations of BSMVs for a significant reversal in the ranks of expression levels between genes associated with different BSMVs across experiments. We also tested whether, when experiments are ordered according to the average difference in rank between BSMVs, a line fitted through the average ranking of one BSMV has a positive slope, and a line fitted through the average ranking of the second BSMV has a negative slope. We found that 8 of the 17 functional BSMVs pass both of these tests (14/37 individual comparisons). For these cases, we suspect that the simple binding affinity model can be rejected in favor of a cofactor interaction model.

### Condition specificity of functional TF binding site variants

Our test can only detect functional BSMVs given a dataset of expression patterns across heterogeneous experimental conditions. These conditions must be different enough from each other to create condition-specific expression patterns that cause differential change in the activity of BSMVs. Given this requirement for environmental heterogeneity, the VDRE statistic is agnostic about the relationship between individual gene expression experiments, whether they represent replicates, points in a time series, different concentrations of media additives, or equivalent treatments conducted in different labs. Yet the VDRE approach predicts that the effect of the BSMV will group individual experiments into biologically meaningful clusters, because there will be a detectable and consistent reordering of the expression ranks only when a proportion of the experiments are similarly affected. To test this prediction, we grouped the experiments into classes according to basic treatment (starvation, sporulation, etc.), and examined whether like-experiments cluster together in their explanation of the functional BSMVs.

**Figure 2:**
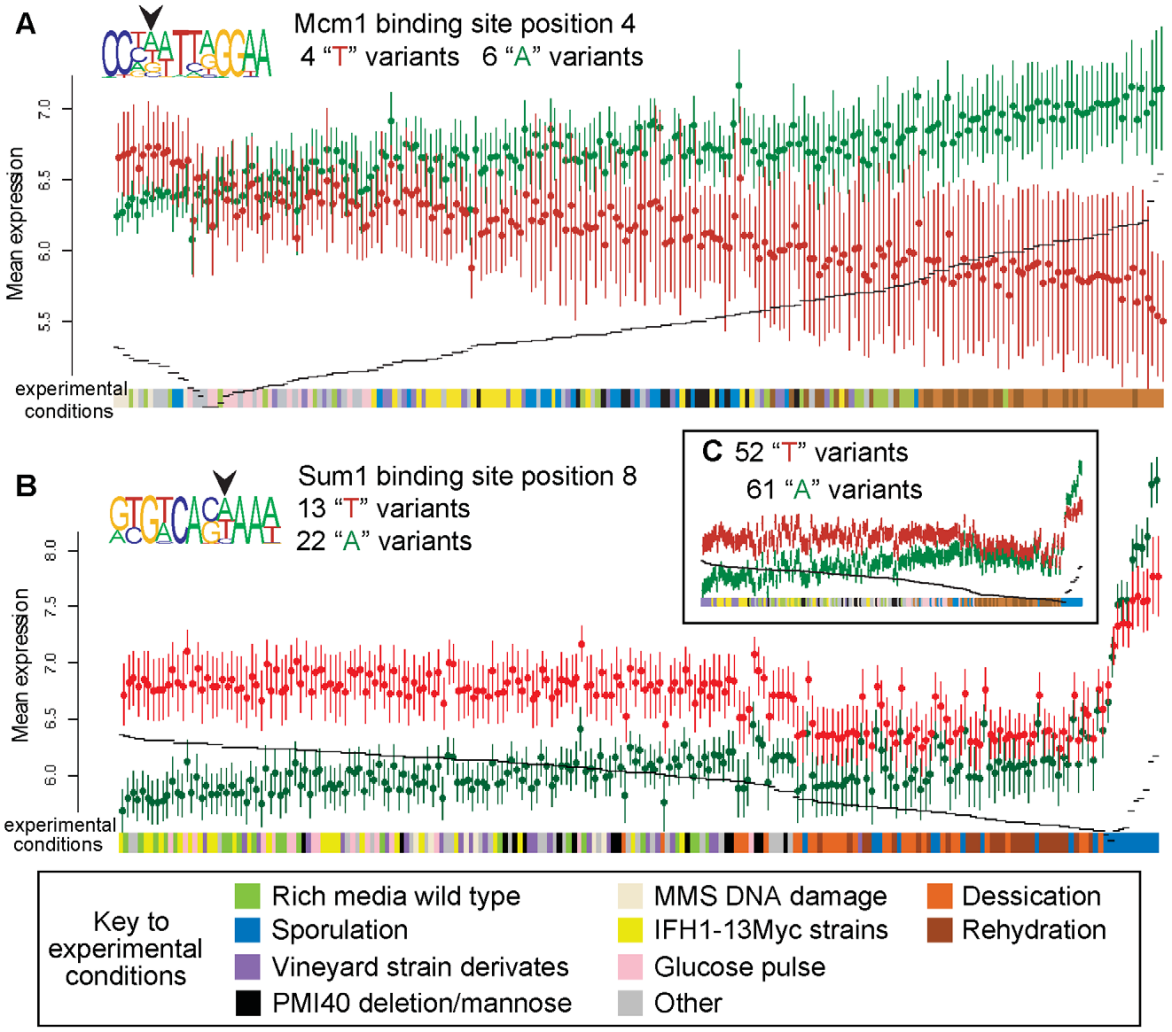
Examples of binding site motif variants (BSMVs) associated with condition-specific gene expression. Mean expression values (Affymetrix; y axis) of genes with each of two BSMVs are plotted on each graph (standard error of mean shown), although more BSMVs may be present at that position. The means are ordered across conditions (x axis) according to the difference in mean expression between the two BSMVs (black dashes). (**A**) Mcm1, involved in cell-type-specific transcription and pheromone response, has functional variants at position 4 of its binding motif. Genes with “T” at position 4 of the Mcm1 binding site (red) are induced relative to genes with “A” BSMVs (green) after DNA damage with MMS. While undergoing desiccation and rehydration, genes with “A” BSMVs are induced in comparison to genes with “T” BSMVs. (**B**) Sum1, a regulator of sporulation-specific genes, has functional variants at position 8 of its binding motif. Genes with “T” (red) at position 8 of the Sum1 binding site have higher expression than genes with “A” BSMVs (green) during rich media growth in lab or IFH1 myc-tagged strains or glucose pulse after starvation. In sporulation, genes with “A” BSMVs are expressed higher than genes with “T” BSMVs. (**C**) The effect of the functional variant at position 8 of Sum1 on target genes remains the same when also considering target genes under more complex regulatory control (multiple primary binding sites).

For each pairwise combination of nucleotides observed at a functionally variant motif position, we ordered all the experiments by the relative mean expression difference between the sets of target genes of each BSMV, and found that similar experimental treatments group together (p<0.05) in all 47 pairwise comparisons in the Affymetrix dataset (Table S3). For example, the effect of “A” and “T” BSMVs at position 4 of the Mcm1 binding site are different after exposure to MMS compared to desiccation and rehydration (p<0.0001; Fig. 2A). Similarly, the effect of “A” and “T” BSMVs at position 8 of the Sum1 binding site are different during sporulation compared to all other treatments (p<0.0001; Fig. 2B, 2C).

**Figure 3:**
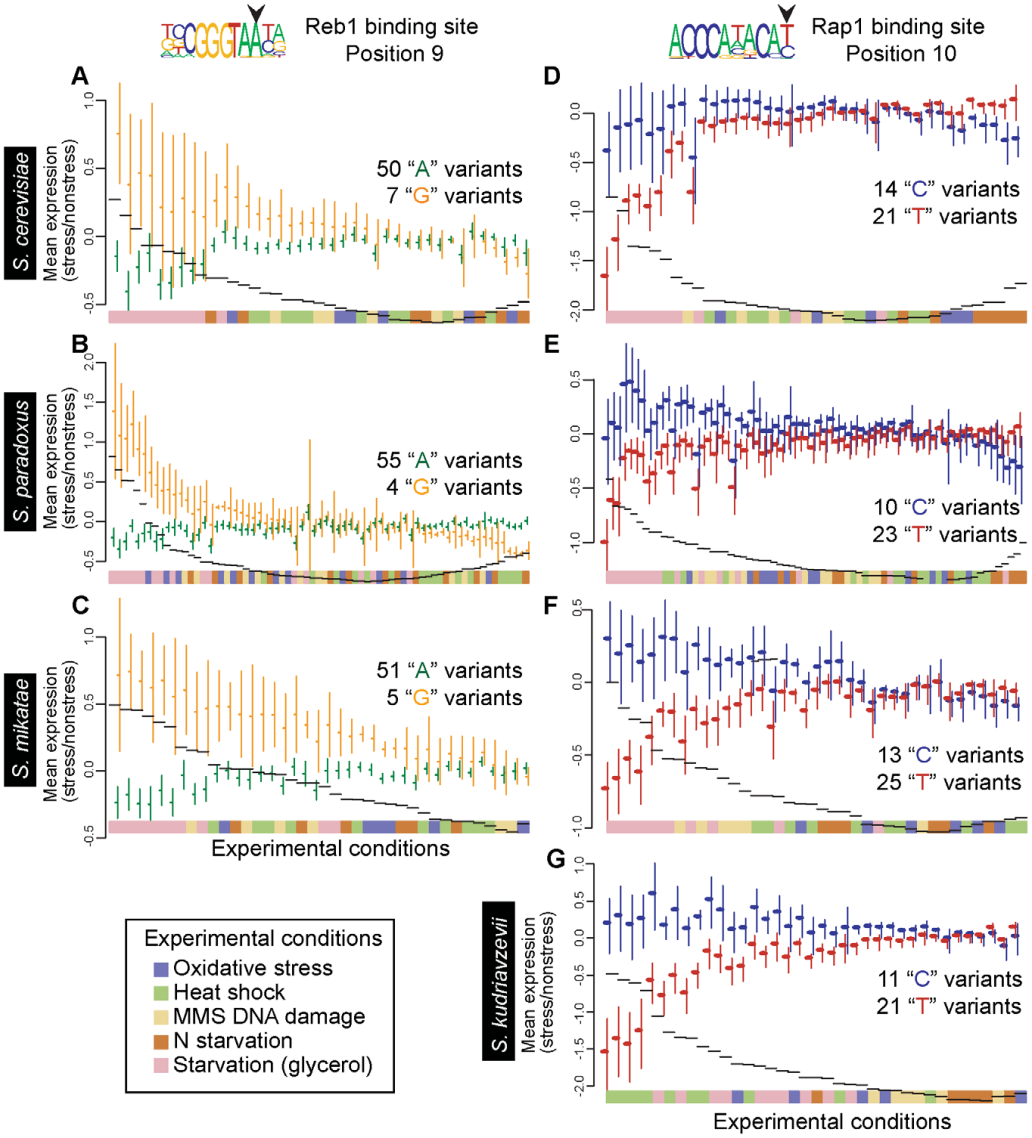
Conserved expression patterns associated with functional binding site motif variants (BSMVs). The y-axis of each plot is the mean expression (Y6.4kv6 arrays) standard error of mean shown) of the stress condition relative to the non-stress condition and the x-axis is experimental treatment, ordered by the difference between the means of genes with each BSMV (black dashes). The function of variant nucleotides at position 9 of the Reb1 binding motif is conserved in (**A**) *Saccharomyces cerevisiae*, (**B**) *S. paradoxus*, and (**C**) *S. mikatae*. In all three species, genes associated with the “G” BSMV (orange) are more highly expressed than genes associated with the “A” BSMV (green) in starvation conditions (glycerol). The function of variant nucleotides at position 10 of the Rap1 binding motif is conserved in (**d**) *S. cerevisiae*, (**e**) *S. paradoxus*, (**f**) *S. mikatae*, and (**g**) *S. kudriavzevii*. In all four species, genes associated with the “C” BSMV (blue) are more highly expressed than genes associated with the “T” BSMV (red) in starvation conditions (glycerol), and the opposite relationship is apparent during nitrogen starvation. The expression differences between the BSMVs are significantly condition-specific in panels a-f (p<0.005).

We also find that 56 out of 83 pairwise comparisons between functional BSMVs identified from an among-species comparative dataset (described below in the “conservation” discussion section) show condition specificity (Table S3). For example, the difference in regulation of genes with “G” or “A” at position 9 for the Reb1 binding sites is highest during growth in glycerol in all three *Saccharomyces* species examined, and therefore these experiments cluster together in Figure 3A–C.

As an independent line of evidence supporting the condition-specific action of functional BSMVs, genes associated with particular BSMVs often show enrichment for gene ontology (GO) processes consistent with their condition specific effects (Table S4). In the example of Mcm1, genes with an “A” variant of the binding site are induced during desiccation and rehydration (Fig. 2A), and these genes are also enriched for the protein modification GO process (p = 0.004). Genes with “T” BSMVs are upregulated in other conditions, and these genes are enriched for the DNA metabolism GO process (p = 0.02). In the example of Sum1, genes with an “A” variant at position 8 of the binding site are upregulated specifically during sporulation (Fig. 2B, 2C) and are also enriched for the sporulation GO process (p<0.001). Genes with a “T” BSMV at this position are upregulated in other conditions, and these genes are enriched for the protein biosynthesis GO process (p = 0.04).

### Reliability of predictions

**Figure 4:**
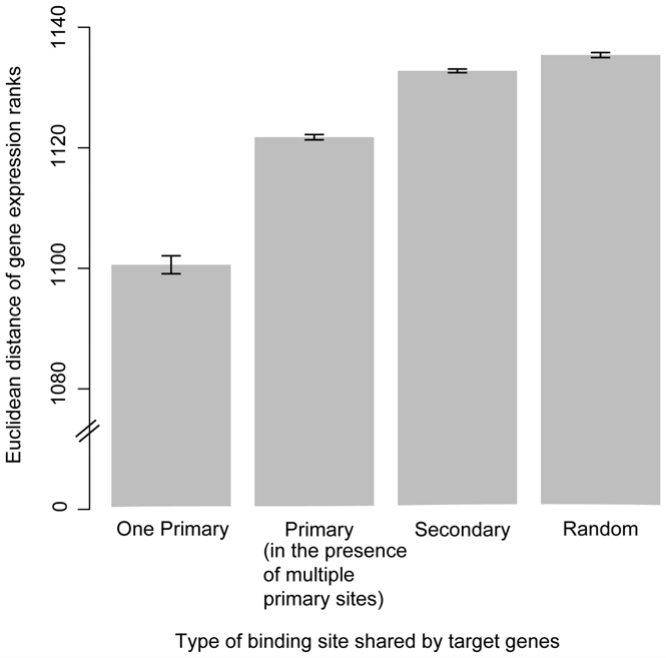
Pairwise expression profile distances (VDRE) between genes that have different types of binding sites in common. With “one primary” binding site in common, target genes have only a single primary binding site (posterior probability >0.7), and pairwise comparisons are between target genes that have a binding site with the same TF identity. With “primary (in the presence of multiple primary sites)” binding sites in common, target genes may have multiple primary binding sites, and pairwise comparisons are between target genes that have a binding site with the same TF identity. With “secondary” binding sites in common, pairwise comparisons are between target genes that have a secondary binding site (posterior probability <0.7 and >0.2) with the same TF identity. With “random” binding sites in common, pairwise comparisons are between random pairs of genes. Standard error bars are indicated.

The quality of the binding site annotations for a TF and the extent to which the TF's target genes are influenced by the TF are both important for our conclusions. To increase our power to detect functional BSMVs, in the analysis presented above we focused on target genes with simple regulatory control regions. As a proxy for regulatory simplicity, we selected only those target genes with a single primary binding site for the same TF (posterior probability >0.7). Pairs of such genes have significantly more similar expression profiles than pairs of genes that either have additional primary binding sites (p<2.2e-16) or than random gene pairs (p<2.2e-16), as is expected for genes participating in simpler regulatory circuits (Fig. 4). Target genes sharing secondary TF binding sites (posterior probability <0.7 and >0.2) have significantly less similar expression profiles than target genes that share a single primary binding site (p<2.2e-16), indicating that the posterior probabilities of the binding site predictions are reasonable. While we relied on genome-scale binding site annotations, a small number of RAP1 binding sites have been experimentally determined; these sites show the same pattern between BSMVs and target gene expression as predicted to be functional in this paper (Fig. S3). This suggests good concordance between the genome-scale annotations and experimentally validated sites for the binding sites considered in this study.

**Figure 5:**
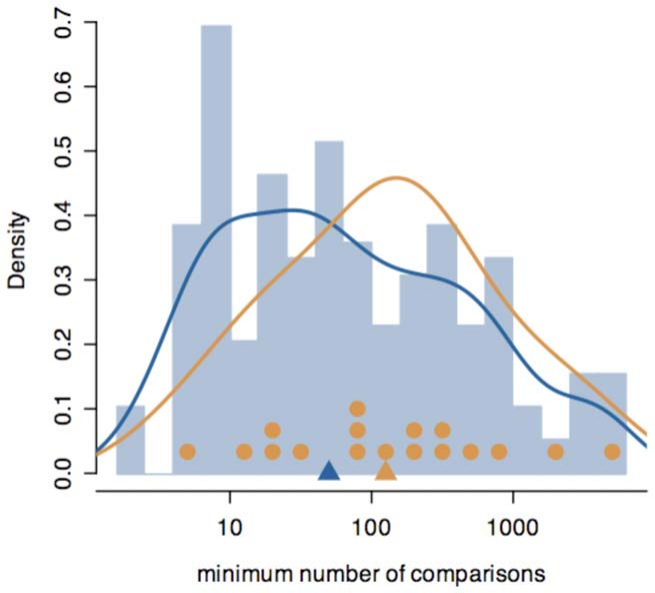
Distribution of the number of within and between variant comparisons between gene expression profiles of positions with binding site motif variants (BSMVs). For each motif position, the lower of either the number of within-BSMV comparisons or between BSMV expression comparisons was counted. The blue line and blue bars represent the distribution of all counts, while the orange line and orange dots represent the distribution of only the positions that are functionally variable. Triangles indicate the median of the two distributions. The distribution suggests that there are a reasonable number of comparisons available for most positions.

BSMVs considered in the analysis have, on average, ∼35 target genes, and functional BSMVs do not have a significantly different number of target genes than do non-functional BSMVs (p = 0.4; Fig. S4). However, power to identify functional BSMVs is a function of the number of within and between-BSMV comparisons, not simply the number of target genes. If BSMVs are distributed unevenly among genes or if genes are partitioned into too many BSMV groups, then there may be few between-BSMV comparisons or within-BSMV comparisons, reducing the power. We find fewer functionally variant binding site positions in our dataset that have a small number of either within or between comparisons than do all variable motif positions, indicating that our permutation test does not spuriously indicate sites for which there is too little information to reliably classify them as functional, and that most variable binding positions have a substantial number of both within and between comparisons (Fig. 5). The permutation test itself accounts for correlation structure due to multiple pairwise comparisons.

We selected genes with only a single primary TF binding site, yet it is possible that functional BSMVs we detect are due to co-occurrence between a particular BSMV and a TF binding site that falls below our primary stringency threshold or is not otherwise known or annotated. In this case, similar expression profiles may be caused by the presence of separately binding TFs [41]. The non-random presence of such secondary binding sites may either be biologically related to cooperation with the BSMV, or may occur by chance. To examine possible co-occurrences in our dataset, we searched for correlations between functional BSMVs and secondary (lower quality) binding sites. For at least 13 of the 17 motif positions with functional variants (and 44 of the 54 positions discovered across species, below), we can exclude the possibility that the association between BSMV and expression profile is due to a secondary TF binding site co-occurring with a particular BSMV (Table S5). We note that most secondary binding sites probably do not represent real binding sites, and we suspect that these correlations are due by chance to the extremely large number (∼18,000) of secondary binding sites genome-wide. Our estimate of the fraction of functional BSMVs that could potentially be explained by additional low probability binding sites is conservative, since we cannot consider TFs that do not yet have characterized binding sites.

### Many functional BSMVs are conserved among yeast species

We applied our method to each of four *Saccharomyces sensu stricto* species, using a published comparative data set of gene expression during stress conditions, assayed on a single cDNA microarray platform [42]. In this dataset, we found that ∼30–39% of TF binding motifs have functionally variant positions in *Saccharomyces sensu stricto* species (Table 1; Table S1; Fig. S5). This proportion is comparable to the ∼29% of motifs with functional variants identified in the Affymetrix dataset. Nine out of these 21 motifs have functional variants that are conserved in more than one species. These conserved functional BSMVs comprise about one fifth (9/42) of the positions identified as having functional variants (Table 2).

**Table 2:** Functional binding site motif variants conserved among *Saccharomyces sensu stricto* species.

Binding site motif family	Position	*S. cerevisiae* p-value	*S. paradoxus* p-value	*S. mikatae* p-value	*S. kudriavzevii* p-value	Mean information content (bits)^b^
Abf1	6	0.862	0.923	0.034*	0.035*	1.05
Cin5	9	0.502	0.000*	0.087	0.043*	1.08
2PAC^a^	11	0.713	0.023*	0.000*	0.047*	1.01
Rap1	10	0.005*	0.058	0.007*	0.031*	0.99
Reb1	9	0.035*	0.039*	0.037*	0.892	1.46
Rpn4	10	0.026*	0.041*	0.255	0.199	0.58
Spt15	2	0.010*	0.189	0.028*	0.178	1.15
Stb5	1	0.017*	0.000*	-	-	0.43
Thi2	3	0.045*	0.027*	0.388	0.821	0.97

a Two adjacent PAC motifs [69] which are bound by *Pbf1* and *Pbf2* [70].

b Maximum information content based on binding site motif nucleotide frequencies is 1.96.

**Figure 6:**
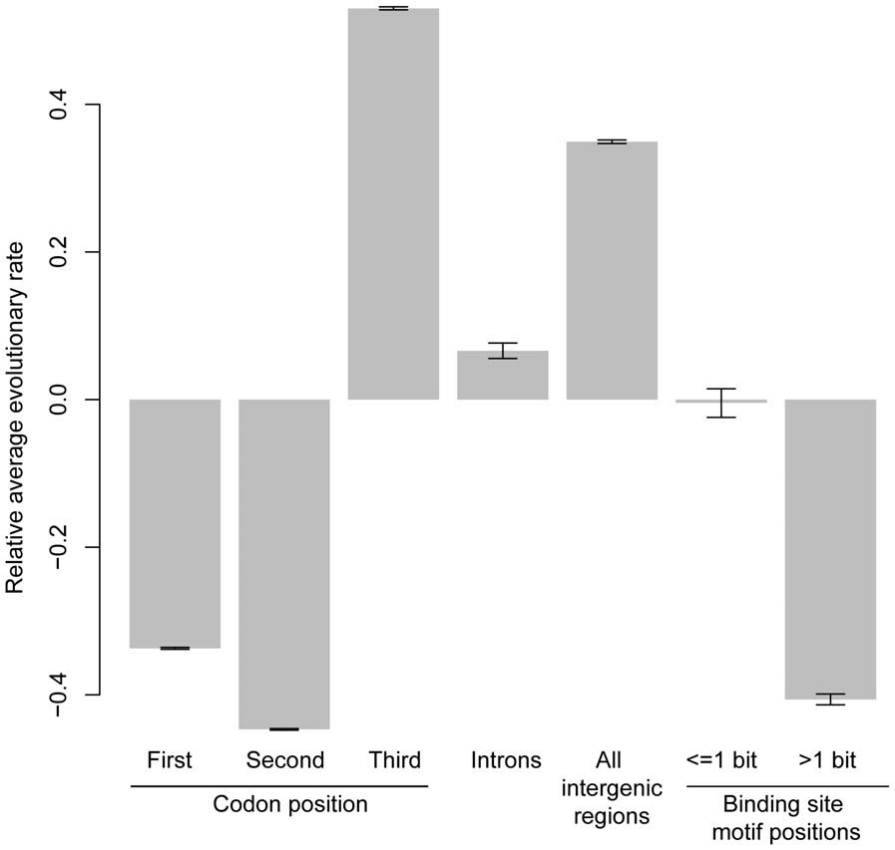
Variable and highly variable binding site motif positions are evolutionarily constrained. The relative evolutionary rate of binding site motif positions that are variable (>1 bit of information) and highly variable (≤bit of information) evolve more slowly than putatively neutral sites: third codon positons, introns, and intergenic regions. First and second positions, which are more functionally constrained, are also shown. Rates were calcualted from a whole-genome alignment of *Saccharomyces sensu stricto* species using emperical Bayesian estimation.

This conservation suggests that the BSMVs are under evolutionary constraint to preserve their function. Indeed we find that there is purifying selection acting both on variable and highly variable motif positions. We calculated the average evolutionary substitution rate of each site across the *Saccharomyces sensu stricto* phylogeny, and found that low information BSMV positions (≤1 bit of information) evolve significantly slower than sites that are expected to be evolving neutrally: the third position of codons, introns, or other intergenic regions (Fig. 6). These low information BSMV positions have nearly twice the number of evolutionarily invariant sites (65%) compared to third codon positions (37%), and more than intergenic regions (47%) and introns (62%; Table 3). These results suggest that even highly variable binding site motif positions are functionally constrained. This observation is in agreement with previous studies which showed that different rates of nucleotide substitution at binding sites are sometimes associated with functionally different classes of BSMVs, where classes sometimes differ by only a single nucleotide [43], [44].

**Table 3:** Nucleotide diversity of transcription factor binding site motif variants across *Saccharomyces sensu stricto* species in comparison to other sites.

Data type	Invariant positions	95% confidence interval		Average nucleotides per position
Second codon positions	88.12%	88.07%	88.18%	1.13
>1 bit binding site motif positions	86.28%	85.90%	86.65%	1.15
First codon positions	81.59%	81.53%	81.66%	1.2
All codon positions	68.95%	68.91%	69.00%	1.35
< = 1 bit (highly variable) binding site motif positions	65.84%	64.95%	66.73%	1.41
Introns	62.07%	61.59%	62.55%	1.46
All intergenic regions	47.79%	47.69%	47.89%	1.65
Third codon positions	37.17%	37.09%	37.25%	1.74

## Discussion

Considering the limited number of genes that meet our criteria for having a simple *cis*-regulatory promoter, and the finite number of conditions for which expression data is available, the proportion of functional BSMVs (9%) among all motif positions is remarkable.

We turned to the literature to assess the validity of a sample of the functionally variant binding positions we identified. We discuss what is predicted about each example from the VDRE approach alone, and then discuss each prediction in light of experimental evidence from the literature. Position 4 of the Mcm1 binding site, also called the middle sporulation element, is an example of a functionally variant binding site position identified in this analysis (Fig. 2A; p = 0.032). Under conditions where yeast is subjected to desiccation and rehydration, genes with an “A” at this position are induced, in comparison to genes with a “T” at this position. Under conditions where yeast is treated with methyl methanesulfonate (MMS), a DNA-damaging alkylating agent, the genes with an “A” at this position are repressed, in comparison to genes with a “T” at this position. A third category of genes has “C” at this position, and the VDRE scores of all three nucleotides (“A”,“T” and “C”) were considered when determining that the position is functional (Fig. S1). The Mcm1 protein is a member of the MADS box family and plays important roles in several diverse cellular processes; therefore, its binding site has been extensively characterized. When Mcm1 binding sites were selected from a pool of random sequence oligonucleotides, about three quarters of the selected sequences had “A” at position 4, ∼15% contained a “T” at this position, and Mcm1 had a higher affinity to “A” BSMVs than to “T” BSMVs [45]. Putative Mcm1 binding sites were cloned in a heterologous promoter in front of a reporter gene [46], and a Mcm1 binding site was subjected to saturation mutagenesis in front of a reporter [47], and in both cases, Mcm1 binding sites with “A” variants at position 4 showed higher (∼2×–3×) activation of the reporter than “T” (or “C”) variants.

Mcm1 acts as an activator alone, but as a repressor when co-bound with α2. The saturation mutagenesis of the Mcm1 binding site shows that BSMVs have different effects, depending on whether or not the α2 is co-bound [47]. An “A” nucleotide at position 4 of the binding site results in more than twice as much activation of the reporter gene than a “T”, but when α2 is present the high level of repression of reporter gene by the two BSMVs is almost identical–130× for the “A” BSMV and 126× for the “T” BSMV. One reason for this combinatorial effect may be that Mcm1 is known to induce sequence-specific DNA bending, which in turn regulates the formation of ternary complexes with other cofactors [47], [48]. Many of the single base pair changes in the binding site that alter its DNA bending and transcriptional regulation do not affect the affinity of the TF for the binding site [47]. Our finding that the “A” and “T” variants at position 4 of the Mcm4 binding site have different effects under different conditions makes sense because cofactors that act in a BSMV-specific way may be present in only a subset of these conditions. Although we have not determined which cofactor(s) are involved in our case, it is interesting that α2 is absent from the haploid a-mating type strain used in the MMS experiments [49], but present in the a/α diploid strain used in the desiccation/rehydration experiments [50].

Sum1 provides another example of how BSMVs may regulate target genes in a condition-specific manner through the participation of another factor, in this case, a competing transcription factor. During growth in rich media, we find that genes regulated by binding sites with a “T” at this position are induced, relative to genes with an “A” at position 8 (Fig. 2B; significance of functional BSMV p = 0.003). During sporulation, the opposite relationship is observed. (Sum1 binding sites with “C” at this position are also functional; Fig. S1). During vegetative growth, Sum1 induces expression of target genes, and the regulatory difference between genes with different variants at position 8 of the Sum1 binding site is small; indeed, while Sum1 has been shown experimentally through mutagenesis to bind sites with a “T” BSMV at position 8 at about 20% the rate of sites with an “A,” repression of reporter activity remained similar between the BSMVs in that study [51]. However, during sporulation, the repressor Ndt80 is also expressed, and competes with Sum1 for binding to the motif, dictating whether the site acts as a repressor or activator. The relative affinity of the BSMV for Ndt80 versus Sum1 acts as a molecular switch that induces only the genes required for the meiotic G_2_-to-M transition. For the “A” variant at position 8 of the binding site, Ndt80 out-competes Sum1 and causes induction of the target gene, while for the “T BSMV, Ndt80 does not out-compete Sum1, and the repressive effect of Sum1 on the target gene remains the same as it was for the “A” BSMV in the absence of Ndt80. This type of effect may explain why “A” and “T” functional variants at position 8 of the Sum1 binding site have different regulatory associations with target genes in sporulation media versus other conditions.

The functional BSMV at position 8 of the Sum1 binding motif remains significant when also considering target genes with multiple primary inputs using VDRE (p<0.001), and its effect on target genes in different conditions remains the same, even though the number of genes considered is greater (Fig. 2C). Although the method presented here does not explicitly accounts for the effects of both multiple regulatory inputs and BSMVs, such an approach is currently under development [52].

The functional BSMVs revealed using the two different platforms (cDNA vs. Affymetrix) were largely non-overlapping. This is the expected result since the regulatory function of BSMVs we detect is condition specific, and the conditions investigated in these sets of experiments are different.

A proportion of the functional BSMVs were identified in multiple species, suggesting that the BSMVs are under evolutionary constraint to preserve their function. For example, position 9 of the Reb1 binding site was identified as having functional BSMVs in *S. cerevisae, S. paradoxus* and *S. mikatae*. Genes regulated by binding sites with a “G” at this position are induced relative to genes with an “A” during growth in glycerol in all three species (Fig. 3A–C). In a small-scale affinity selection experiment, Reb1 had lower binding strength to sites with “G” at position 9 than to sites with “A” at position 9, and “G” BSMVs promoted lower transcriptional activity than “A” BSMVs when grown on 2% glucose plates [53].

Position 10 of the Rap1 binding site also has functional BSMVs identified in multiple species. During glucose starvation conditions (growth in glycerol), genes with “C” BSMVs are induced with respect to genes with “T” BSMVs (Fig. 3D–F). Differences in affinity of Rap1 binding sites have been shown to be specifically associated with expression in low glucose conditions, according to a precise set of experiments including ChIP-chip, protein binding microarrays, deletion mutants, and gene expression analysis [25], [27]. High affinity sites are constitutively bound by Rap1, while low affinity binding sites are protected by chromatin structure from Rap1, except during low glucose conditions, when chromatin conformational changes expose them, and Rap1 binds and induces expression. According to our method, such BSMV-by-condition patterns for Rap1 can be learned from accurate binding site predictions and expression patterns alone.

### Conclusions

Yeast has only around 200–300 TFs to regulate its complex regulatory function—from budding to the cell cycle to selectively metabolizing dozens of different energy sources. The fundamental question in regulatory biology is how a relatively small number of TFs orchestrate the regulation of thousands of genes to achieve innumerable phenotypic responses. The fine-tuning of TF binding motifs at non-consensus positions may provide an important source of control in coordinating these condition-specific expression patterns.

In this study, we found that a significant proportion of variable positions in TF binding motifs may have functional consequences. Several of these predictions are in agreement with available experimental evidence, and several are corroborated by conservation across species. We considered only a single variable position at a time and did not explicitly account for promoters with complex regulatory inputs. More functional BSMVs should be found if combinations of positions and/or binding sites are formally considered [52].

Functional BSMVs allow the same TF to have a broad range of regulatory effects simultaneously over different target genes. Our results, consistent with the molecular biology literature, show that these differential regulatory effects between BSMVs can change with the concentration of the TF and/or the concentration of cofactors across environmental or cellular conditions.

As the complexity of organisms increase, the complexity of their regulatory responses needs to also increase to accommodate differential expression across tissues and numerous developmental stages. We therefore expect that the contribution of functional BSMVs to the *cis*-regulatory code of higher eukaryotes may be even more pronounced, an idea supported by the observation of such BSMVs in the experimental literature in diverse organisms such as nematode [20], [23], fly [29], mouse [31], and human [24].

## Materials and Methods

### Binding site predictions

Binding site annotations were obtained from SwissRegulon [40], where position weight matrices (PWMs) from over-represented motifs in microarray bound DNA regions from high-throughput chromatin immunoprecipitation of 102 TFs [54] in *S. cerevisiae* were calculated by PhyloGibbs [55], and where these PWMs were inputted into MotEvo, which is a scanning algorithm that finds hits to a PWM, but also considers conservation in other species [56]. To obtain a set of genes under putatively simple forms of regulatory control, we included promoters and target genes with a single primary TF binding site, where primary binding sites have a posterior probability (according to MotEvo) of 0.7 or greater. This results in 1219 genes included among the target sets for the Affymetrix expression data set (see below) and between 648 and 664 genes, depending on the species, for the cDNA expression data sets. For comparison, we also examined sets of genes with multiple primary binding sites, or with secondary binding sites, which have a posterior probability less than 0.7 but greater than 0.2.

The lengths of the binding site motifs vary from 6 bp to 16 bp. The information, R*_ij_*, at position *j* of site *i*, was calculated according to [57], given the following base frequencies: f_A_ = 0.307, f_C_ = 0.188, f_G_ = 0.188, and f_T_ = 0.316.

### Comparative alignment of binding sites

To calculate the nucleotide substitution rate at each position in each binding site, we performed a whole-genome multiple sequence alignment and pairwise alignments using MAUVE [58] between *S. cerevisiae* and *S. bayanus*, *S. mikatae*, *S. paradoxus* and *S. kudriavzevii*, and discarded regions of sequence that are gapped in *S. cerevisiae*. We discarded the binding site if the alignment from the pairwise and multiple sequence alignments was different or if gaps existed in the sequence, except in the following cases: (1) no orthologous alignment of the region was produced by one method (pairwise or multiple) but an ungapped alignment was produced by the alternative method and (2) the alternative alignments of the binding sites are the same except for the introduction of gaps into the pairwise alignment, in which case we used the multiple alignment. We discarded positions that contain a gap or are unalignable.

Branch lengths for the five-species phylogeny [59] were calculated in PAML under a reversible model and a gamma distribution of rates with four categories [60]. Site-specific normalized average evolutionary rates were estimated based on this genome alignment and phylogeny using empirical Bayesian estimation in the program Rate4Site 2.01 [61]. A gamma distribution of rates with 35 rate categories was fit with an alpha parameter value of 0.62. The mean rate of sites from each of the following classes was calculated: coding regions (first, second and third positions), introns, intergenic regions, binding site positions with greater than one bit of information, and binding site positions with less than or equal to one bit of information. The 95% confidence interval for the mean rates of each class was calculated from 1000 nonparametric bootstrap replicates in the R package boot. We also calculated proportion of invariant sites for each of these classes. The 95% confidence interval for these proportions was calculated using the method of Wilson [62].

### Microarray expression analysis

We assembled a concatenated expression microarray dataset representing *S. cerevisiae* expression in 211 experimental conditions from S98 Affymetrix array data in the NCBI Gene Expression Omnibus. To maximize comparability we chose to use data from only one array-type, Affymetrix S98, which has the largest number of experiments available among all platforms and array designs. Expression values were normalized by the robust multi-chip average (rma) algorithm implemented in the R-affy package of Bioconductor [63].

The second expression dataset we used contains measurements under equivalent environmental stress conditions for four yeast species from the *Saccharomyces sensu stricto* complex: *S. cerevisiae*, *S. paradoxus*, *S. kudriavzevii* and *S. mikatae* measured on Y6.4kv6 cDNA arrays (GEO record GSE3406 [42]). cDNA hybridization after each stress condition for each species was measured relative to cDNA before application of the stress condition for that species, with either four biological replicates (two stress conditions) or across six time points (five stress conditions).

### Variant distance of ranked experiments (VDRE) and statistical analysis

In order to make between-gene comparisons of expression across conditions, we rank-ordered each gene's expression level across conditions, and used the ranks as a proxy for the expression levels in all analyses. To identify functional BSMVs, we selected a set of binding site positions that have at least two target genes for at least two BSMVs. For a given position in a motif, we compared two distributions of pairwise Euclidean distances between the ranked expression profiles (VDRE): D*_W_*, the distribution of distances between pairs of targets that have the same BSMV, and D*_B_* the distribution of distances between pairs of targets that have different BSMVs. The functionality score of the motif at one position is:
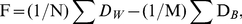
(1)where N is the number of within-BSMV pairwise distances in the summation and M is the number of between-BSMV pairwise distances, and the distances between target genes of all variants of a binding site motif are considered simultaneously for a single motif position. We stress that this measure for assessing the functionality of BSMVs incorporates expression distances between target genes of all the nucleotide variants of that binding site position, not only between genes associated with just two BSMVs. The significance of the association between BSMV and expression profile was calculated by permuting the assignment of target genes to BSMVs 1000 times and comparing observed values of F with the permuted null distribution. The full set of motif positions with functional BSMVs detected in each species is shown in Table S1. The false discovery rate was calculated using the p-values from the observed data [64].

For comparison, we also identified functional BSMVs for all genes, including genes with more than a single primary binding site in their promoter. The same methodology was followed as for genes with a single primary input. We checked to see whether the nucleotides defining the functional BSMVs are correlated with low probability, secondary binding sites (posterior probability <0.7 and >0.2).

We assessed the coincidence of secondary binding sites for all other TFs with each BSMV at each a functionally variant position. More specifically, among the target genes for a TF with functional BSMVs at a particular position, we tallied the number of targets with a particular BSMV, or a particular coincident low probability binding site, both or neither, resulting in a 2×2 contingency table that we tested for a significant correlation with Fisher's exact test at a level of 0.01.

Genes that are bound by the same transcription factor are often co-expressed [65]. To assess our assumption that genes with only a single primary binding site are subject to simpler regulatory control than genes with additional binding sites, we compared gene expression between the set of all genes with a single primary site for a TF versus the set also including genes that are bound by the TF but may also have additional primary binding sites. We used the Euclidean distance between ranked gene expression values as a comparative measure of gene expression value, a measure that is commonly used [66], [67], [68]. We performed a Welch two sample t-test on the two groups to assess whether genes with a single primary binding site (bound by the same TF) have significantly smaller expression distance between them than genes bound by the same TF, but which may also have additional primary binding sites. We similarly assessed our assumption that primary binding sites contain better predictions than secondary binding sites by comparing the expression distance between genes that share a primary binding site versus genes that share a secondary binding site. We compared these distances to expression distances between random pairs of genes.

### BSMV-specific partitioning of experimental conditions

We sought to quantify the biological consistency of the set of experimental conditions for which each functional BSMV explains target gene expression differences. For each pair of BSMVs, we sorted the experiments according to the difference of the mean expression value for the target sets (normalized by the variance in expression value), and then considered whether similar experimental conditions are clustered. We classified all experiments into groups of similar experimental types. For the comparative dataset, each stress condition was considered a single class including all time points and replicates. For the Affymetrix dataset, all rich media wild type/control experiments were classified together. Most other experimental types (e.g. growth in different media types, deletion strains, etc.) were classified into respective categories across time points. One dataset (replicates GSE1311–1314) which includes a large number of experiments was separated into the two stages of the experiment (desiccation and rehydration). The level of clustering of conditions according to differences in expression values of the BSMVs is defined by:
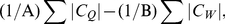
(2)where C*_Q_* the average distance between ranks of experiments of different types, C*_W_* is the average distance between ranks of experiments of the same type, A is the number of same-experimental-type pairwise differences in the summation, and B is the number of different-experimental-type pairwise differences. We compared the observed level of clustering to the distribution of clustering for 10,000 data sets, where the assignment of experimental types is permuted.

We also considered whether target genes associated with different functional BSMVs show an enrichment of particular Gene Ontology (GO) biological processes. For each set of target genes with a given BSMV, we permuted the biological processes between BSMVs to create 10,000 null data sets. We examined whether target genes associated with particular BSMVs show enrichment for biological processes compared to the null distribution for that process. A full list of significant GO enrichments for functional BSMVs is shown in Table S4.

## Supporting Information

Figure S1 **Comparison of average gene expression levels between genes with different functional transcription factor binding site motif variants (BSMVs) in** ***S. cerevisiae*** **(Affymetrix).** Mean expression levels for target genes of functional BSMVs of *S. cerevisiae* using expression data from 211 Affymetrix S98 arrays and a variety of experimental conditions. Even if more than two BSMVs exist at a position, only two are shown in each individual graph, and additional graphs show the pairwise comparison between each BSMV present at each position. The means are ordered across conditions according to the difference between mean expression of the two BSMVs. Vertical lines extending from each point indicate the standard deviation of the mean. Horizontal black bars indicate the difference between the mean ranks. The significance of the functional BSMVs was determined without reference to the segregation of experimental conditions, which are shown according to color along the x-axis. The number of targets for each BSMV graphed are shown at the bottom right hand of the graph.
(PDF)

Figure S2 **Mean expression levels of target genes with multiple primary inputs (posterior probability >0.7), for functional binding site motif variants (BSMVs) that were identified in both a dataset with multiple primary inputs, and limited to genes with a single primary input.** Expression data is from 211 Affymetrix S98 arrays and a variety of experimental conditions. Even if more than two BSMVs exist at a position, only two are shown in each individual graph, and additional graphs show the pairwise comparison between each BSMV present at each position. The means are ordered across conditions according to the difference between mean expression of the two BSMVs. Vertical lines extending from each point indicate the standard deviation of the mean. Horizontal black bars indicate the difference between the mean ranks. The significance of the functional BSMV was determined without reference to the segregation of experimental conditions, which are shown according to color along the x axis. The number of targets for each BSMV graphed is shown at the bottom right hand of the graph.
(PDF)

Figure S3 **Comparison of expression patterns in** ***S. cerevisiae*** **of genes associated with “A” and “T” binding site motif variants (BSMVs) at position 7 of the RAP1 transcription factor binding site based on binding site annotations from small-scale experimental mapping or the genome-wide annotations used in this study.** Of six RAP1 binding sites for single input genes according to SCPD, three identical (same position and sequence) binding sites are found in the genome-wide binding site annotations used in this study. A) Genes with only a single RAP1 binding site and no other TF binding site annotations collected from the experimental mapping literature in SCPD [71] were selected. Average expression values of 3 genes with an “A” nucleotide at position 7 of their associated RAP1 binding site are lower during growth in glycerol than the average expression values of 2 genes with a “T.” Expression values of genes with either BSMV are not different during growth in other stress conditions. B) Position 7 of the RAP1 binding site is functionally variant (p = 0.024) based on our analysis of genome-wide binding site annotations for RAP1 derived from genome-scanning with models based on ChIP-chip binding assays, conservation and motif overrepresentation [40], [54], [55], [56]. Similar to above, average expression of 27 genes with an “A” BSMV at position 7 of their associated RAP1 binding site are expressed at a lower level than 3 genes with a “T” BSMV.
(PDF)

Figure S4 **Number of target genes associated with functional and non-functional binding site motif variants (BSMVs).** Variable motif positions considered in the analysis have, on average, ∼35 target genes, and functional BSMVs do not have a significantly different number of target genes than do non-functional BSMVs (p = 0.4).
(PDF)

Figure S5 **Complete set of figures showing comparison of average gene expression levels between genes with different functional transcription factor binding site motif variants (BSMVs) in S. cerevisiae, S. kudriavzevii, S. mikatae and S. paradoxus based on expression data from Y6.4kv6 cDNA arrays.** Mean expression levels for target genes of functional BSMVs found at positions in TF binding sites using expression data from Y6.4kv6 cDNA arrays and stress conditions. Even if more than two BSMVs exist at a position, only two are shown in each individual graph, and additional graphs show the pairwise comparison between each BSMV present at each position. The means are ordered across conditions according to the difference between mean expression of genes regulated by the two BSMVs. Vertical lines extending from each point indicate the standard deviation of the mean. Horizontal black bars indicate the difference between the mean ranks. The significance of the functional BSMVs was determined without reference to the segregation of experimental conditions, which are shown according to color along the x-axis. The number of targets for each BSMV graphed is shown at the bottom right hand of the graph.
(PDF)

Table S1 **Transcription factor binding site motif positions that have functional variants inferred according to the variant distance of ranked experiments statistic (p<0.05).**
(PDF)

Table S2 **Transcription factor binding site motif positions that have functional variants inferred according to the variant distance of ranked experiments statistic (p<0.05) in both target genes with single primary inputs and multiple primary inputs.**
(PDF)

Table S3 **Condition specificity of functional binding site motif variants (BSMVs).** Significance of segregation of experimental conditions dependent upon upregulation of the major base or minor base. Cases are shown where the position has a functional BSMV and where there is clustering of similar experimental conditions when the experiments are sorted according to the difference in regulation of each-BSMV's target set.
(PDF)

Table S4 **Gene ontology enrichment among genes with alternative binding site motif variants at functionally variant binding site motif positions.**
(PDF)

Table S5 **Secondary transcription factor binding sites correlated with binding site motif variants at nearby functionally variant binding sites.** Secondary binding sites have a posterior probability of <0.7 and >0.2. Significant coincidence of secondary binding sites for each other TF with each nucleotide at each functional binding site motif variant position is given according to Fisher's exact test at a level of 0.01.
(PDF)
