# Supplementary material for: Contribution of Transcription Factor Binding Site Motif Variants to Condition-Specific Gene Expression Patterns in Budding Yeast
Source: PLoS One. 2012 Feb 23;7(2):e32274. doi: 10.1371/journal.pone.0032274 (PMC3285675; doi:10.1371/journal.pone.0032274)
Supplement: Figure S1 — Comparison of average gene expression levels between genes with different functional transcription factor binding site motif variants (BSMVs) in S. cerevisiae (Affymetrix). Mean expression levels for target genes of functional BSMVs of S. cerevisiae using expression data from 211 Affymetrix S98 arrays and a variety of experimental conditions. Even if more than two BSMVs exist at a position, only two are shown in each individual graph, and additional graphs show the pairwise comparison between each BSMV present at each position. The means are ordered across conditions according to the difference between mean expression of the two BSMVs. Vertical lines extending from each point indicate the standard deviation of the mean. Horizontal black bars indicate the difference between the mean ranks. The significance of the functional BSMVs was determined without reference to the segregation of experimental conditions, which are shown according to color along the x-axis. The number of targets for each BSMV graphed are shown at the bottom right hand of the graph. (PDF) [file pone.0032274.s001.pdf]

Figure S1. Comparison of average gene expression levels between genes with different functional variants of transcription factor binding site motifs in *S. cerevisiae* (Affymetrix)

Mean expression levels for target genes of functional variants found at positions in TF binding sites of *S. cerevisiae* using expression data from 211 Affymetrix S98 arrays and a variety of experimental conditions. Even if more than two variants exist at a position, only two are shown in each individual graph, and additional graphs show the pairwise comparison between each variant present at each position. The means are ordered across conditions according to the difference between mean expression of the two variants. Vertical lines extending from each point indicate the standard deviation of the mean. Horizontal black bars indicate the difference between the mean ranks. The significance of the functional heterogeneity was determined without reference to the segregation of experimental conditions, which are shown according to color along the x-axis. The number of targets for each variant graphed are shown at the bottom right hand of the graph.

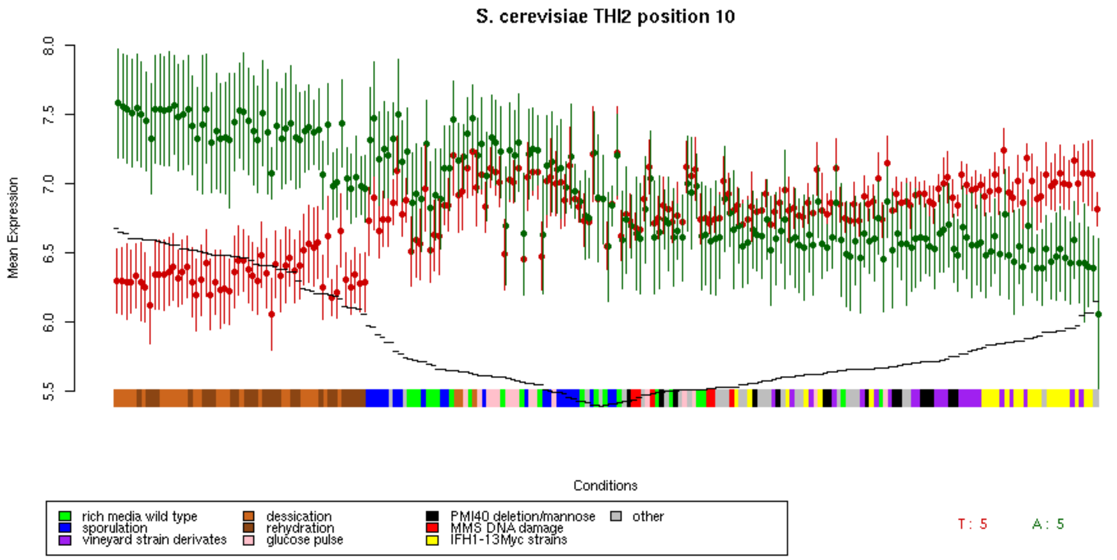

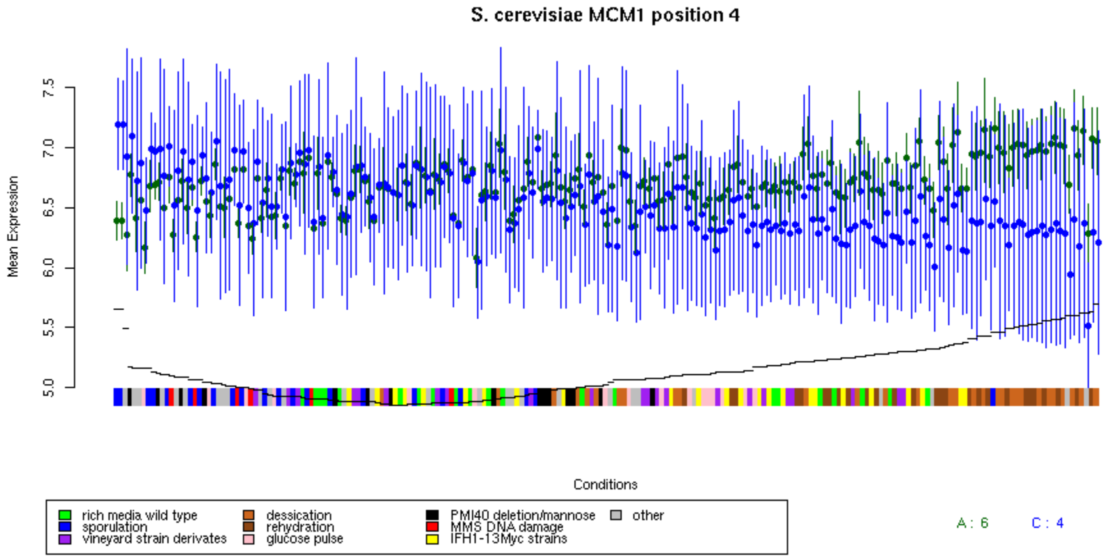

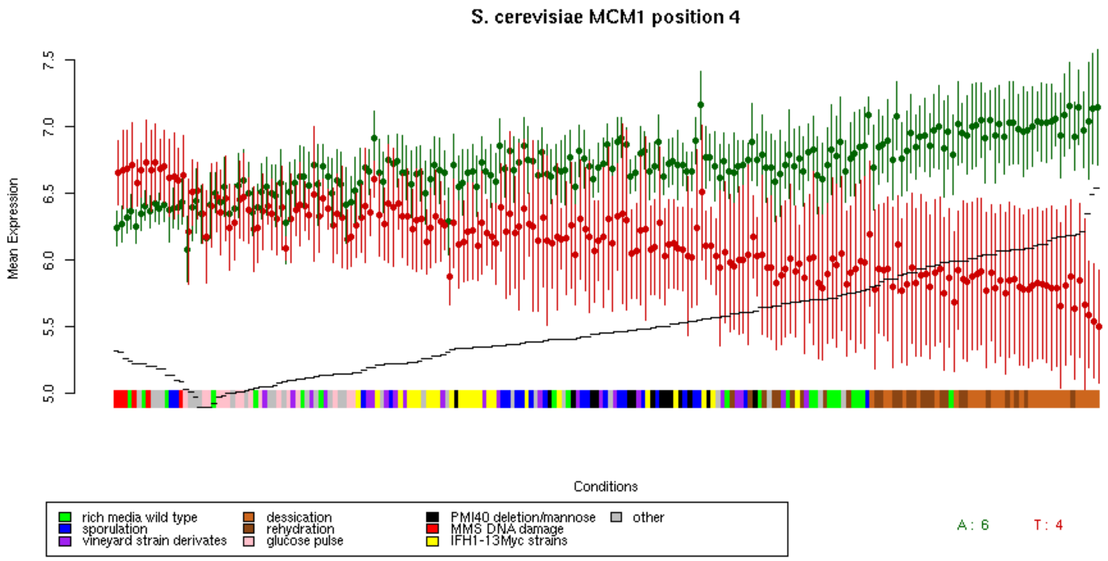
S. cerevisiae MCM1 position 4
Mean Expression
7.5
7.0
6.5
6.0
5.5
5.0
Conditions
rich media wild type
sporulation
vineyard strain derivates
dessication
rehydration
glucose pulse
PMI40 deletion/mannose
MMS DNA damage
IFH1-13Myc strains
other
A : 6
T : 4

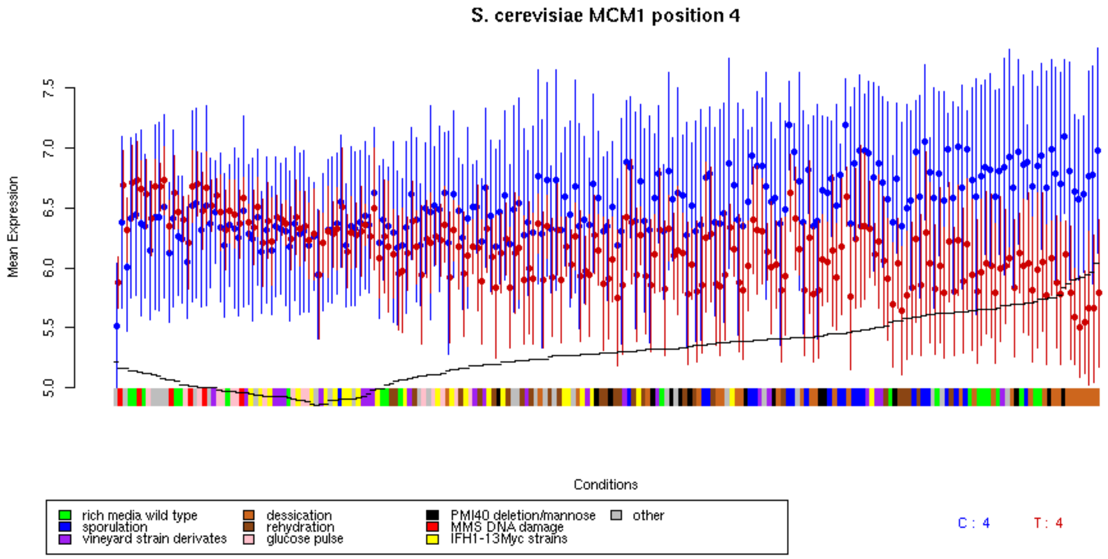
S. cerevisiae MCM1 position 4
Mean Expression
7.5
7.0
6.5
6.0
5.5
5.0
Conditions
rich media wild type
sporulation
vineyard strain derivates
dessication
rehydration
glucose pulse
PMI40 deletion/mannose
MMS DNA damage
IFH1-13Myc strains
other
C : 4
T : 4

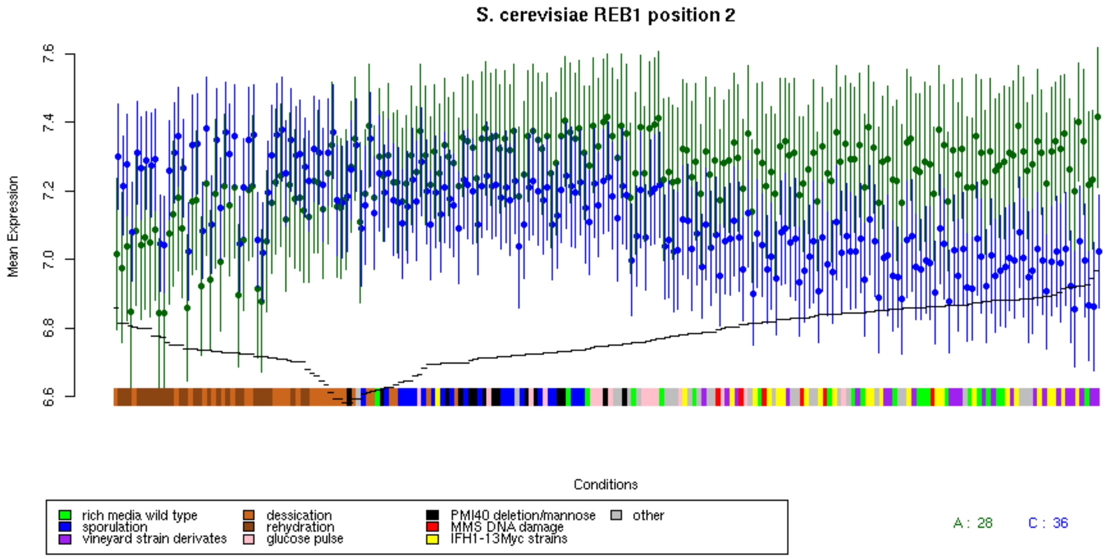
S. cerevisiae REB1 position 2
Mean Expression
7.6
7.4
7.2
7.0
6.8
6.6
Conditions
rich media wild type
sporulation
vineyard strain derivates
dessication
rehydration
glucose pulse
PMI40 deletion/mannose
MMS DNA damage
IFH1-13Myc strains
other
A : 28
C : 36

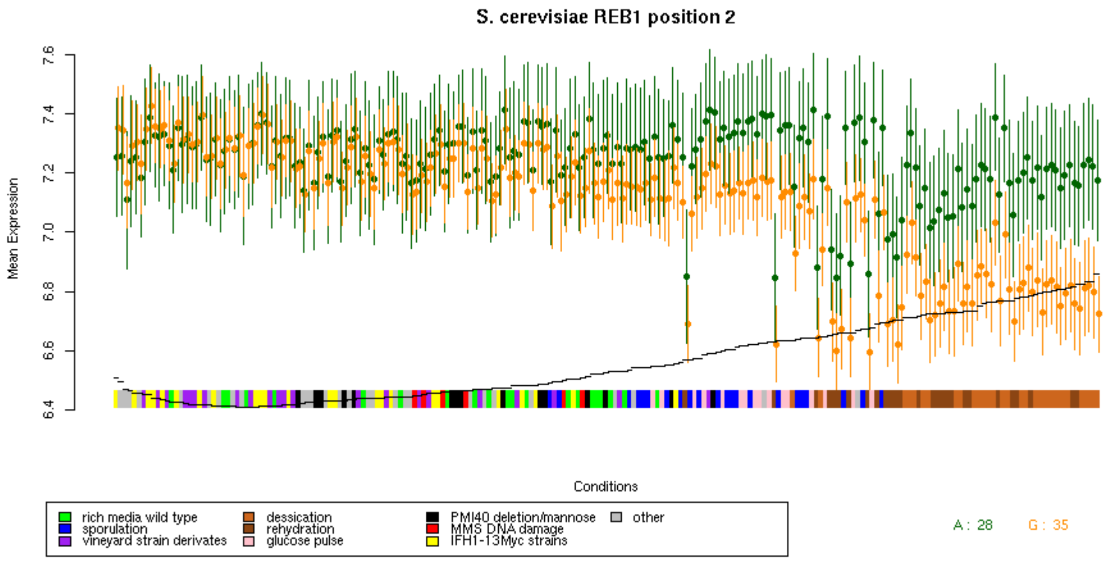
S. cerevisiae REB1 position 2
Mean Expression
Conditions
rich media wild type
sporulation
vineyard strain derivates
dessication
rehydration
glucose pulse
PMI40 deletion/mannose
MMS DNA damage
IFH1-13Myc strains
other
A : 28
G : 35

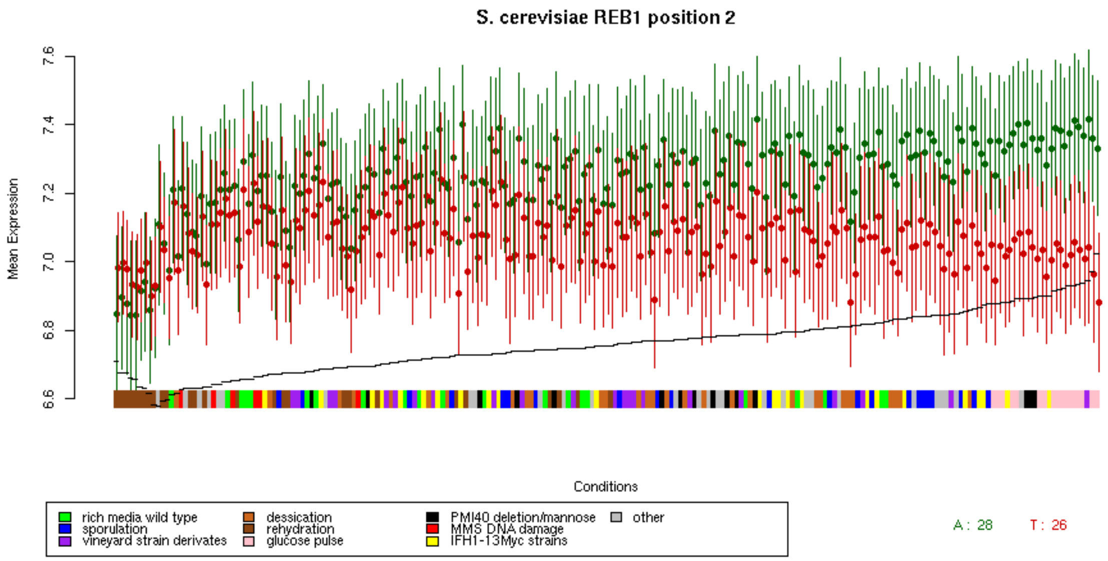
S. cerevisiae REB1 position 2
Mean Expression
Conditions
rich media wild type
sporulation
vineyard strain derivates
dessication
rehydration
glucose pulse
PMI40 deletion/mannose
MMS DNA damage
IFH1-13Myc strains
other
A : 28
T : 26

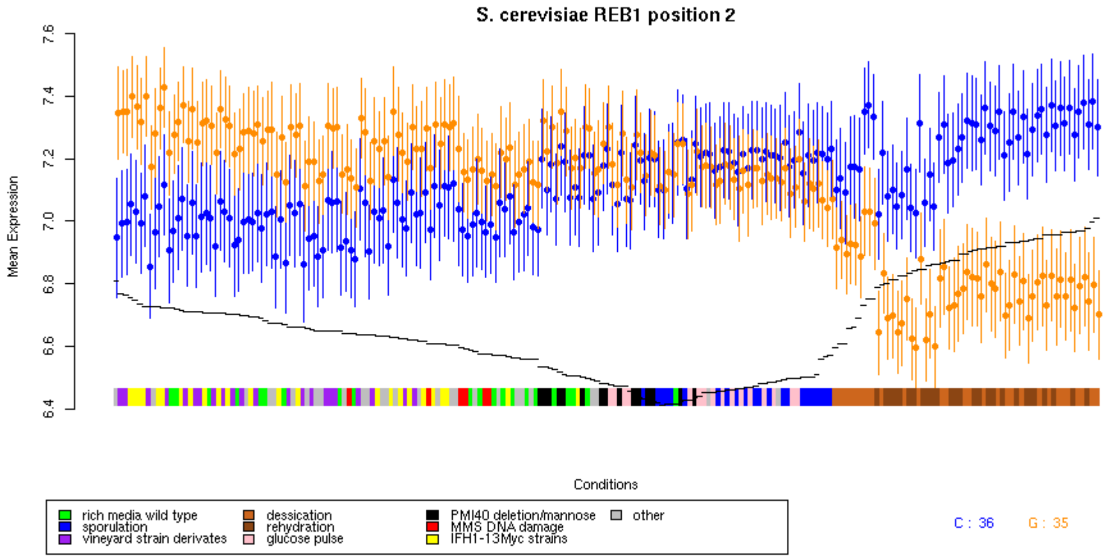
S. cerevisiae REB1 position 2
Mean Expression
Conditions
rich media wild type
sporulation
vineyard strain derivates
dessication
rehydration
glucose pulse
PMI40 deletion/mannose
MMS DNA damage
IFH1-13Myc strains
other
C : 36
G : 35

S. cerevisiae REB1 position 2

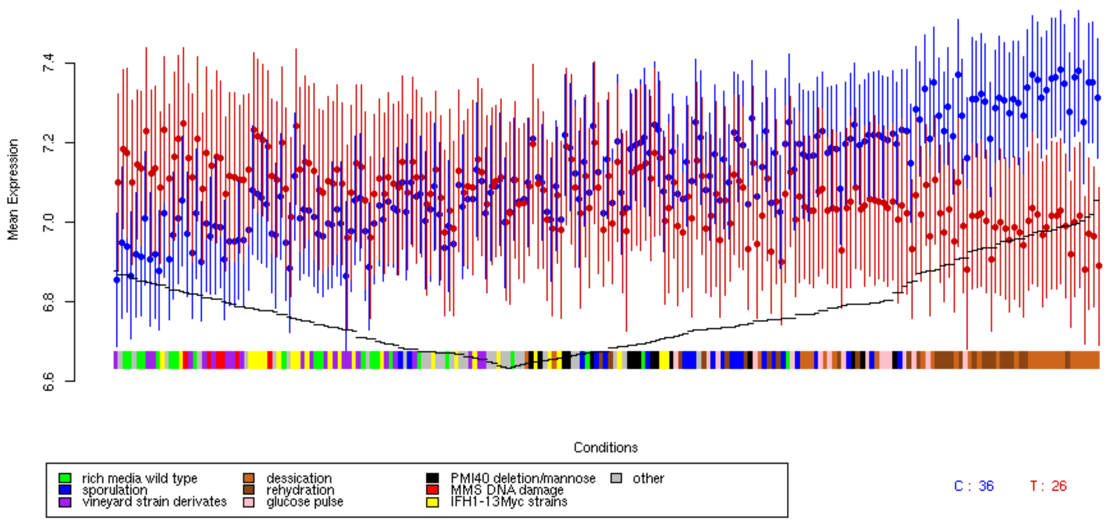

S. cerevisiae REB1 position 2

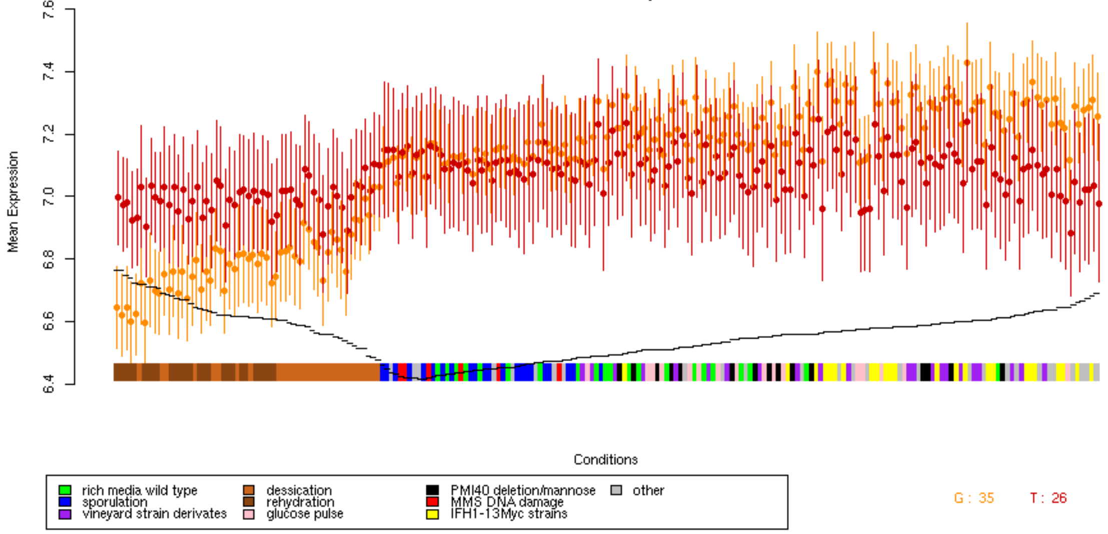

S. cerevisiae STE12DIG1 position 7

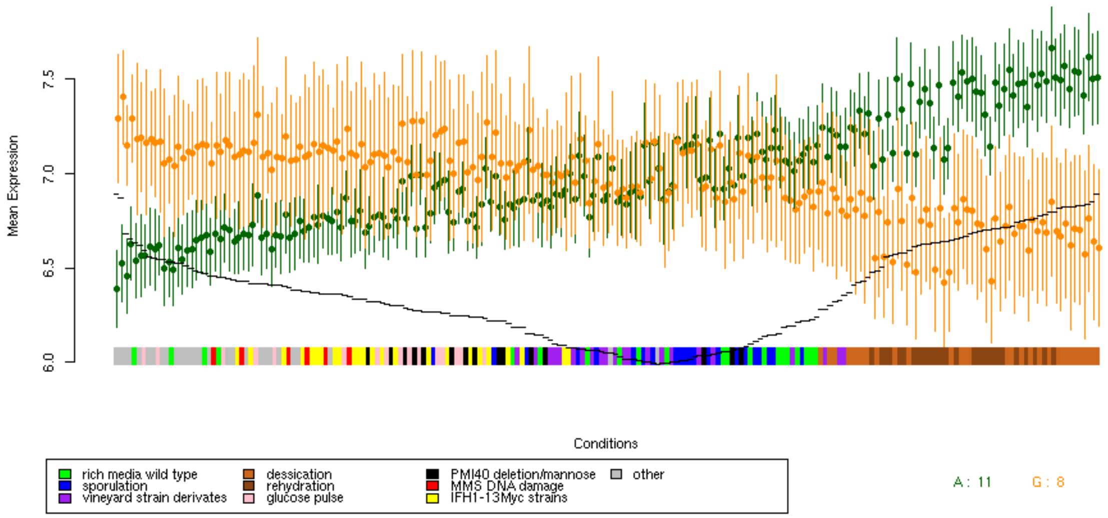

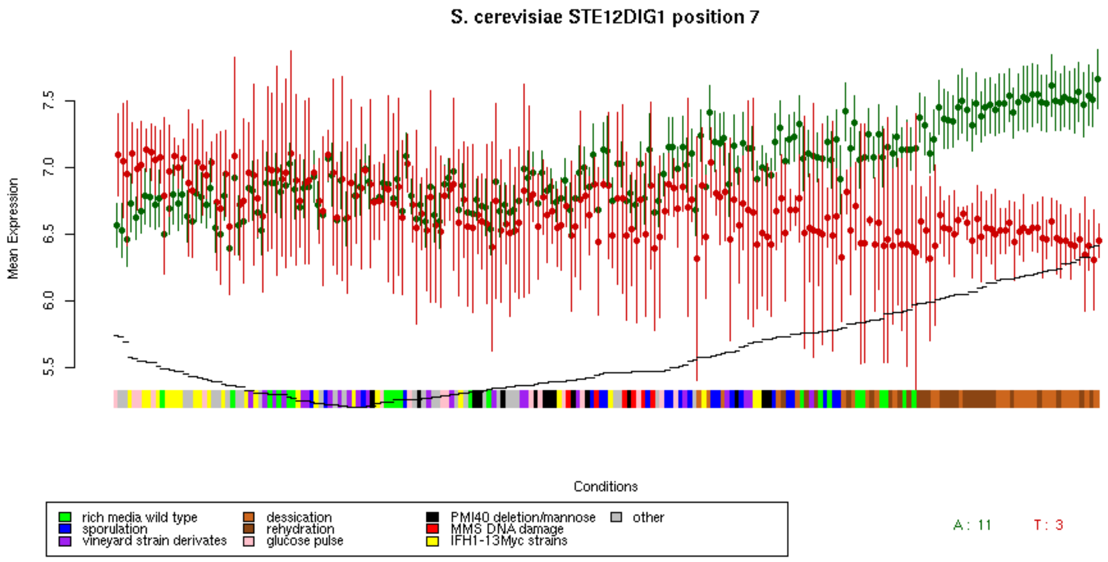
S. cerevisiae STE12DIG1 position 7
Mean Expression
5.5
6.0
6.5
7.0
7.5
Conditions
rich media wild type
sporulation
vineyard strain derivates
dessication
rehydration
glucose pulse
PMI40 deletion/mannose
MMS DNA damage
IFH1-13Myc strains
other
A : 11
T : 3

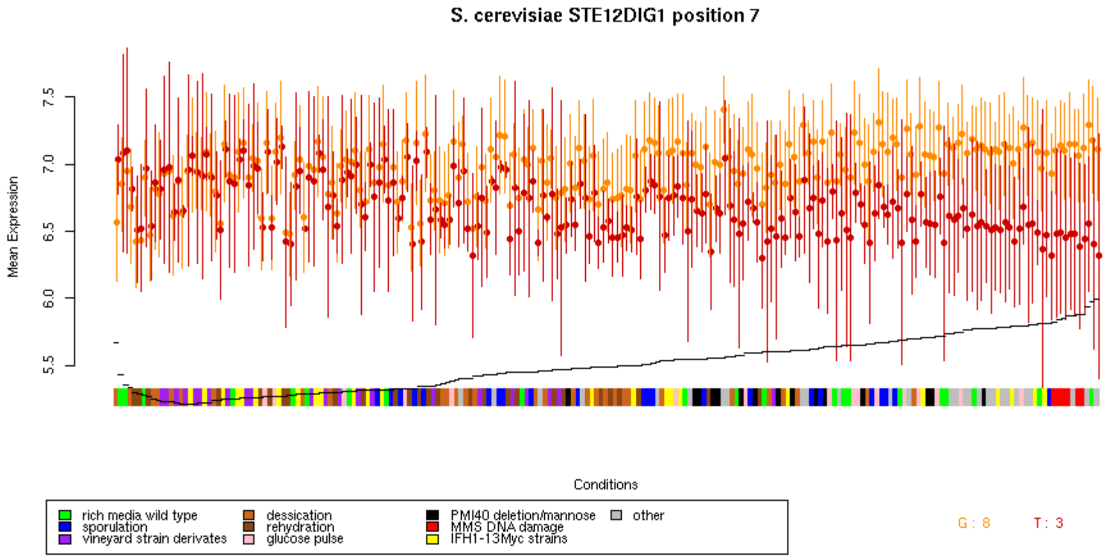
S. cerevisiae STE12DIG1 position 7
Mean Expression
5.5
6.0
6.5
7.0
7.5
Conditions
rich media wild type
sporulation
vineyard strain derivates
dessication
rehydration
glucose pulse
PMI40 deletion/mannose
MMS DNA damage
IFH1-13Myc strains
other
G : 8
T : 3

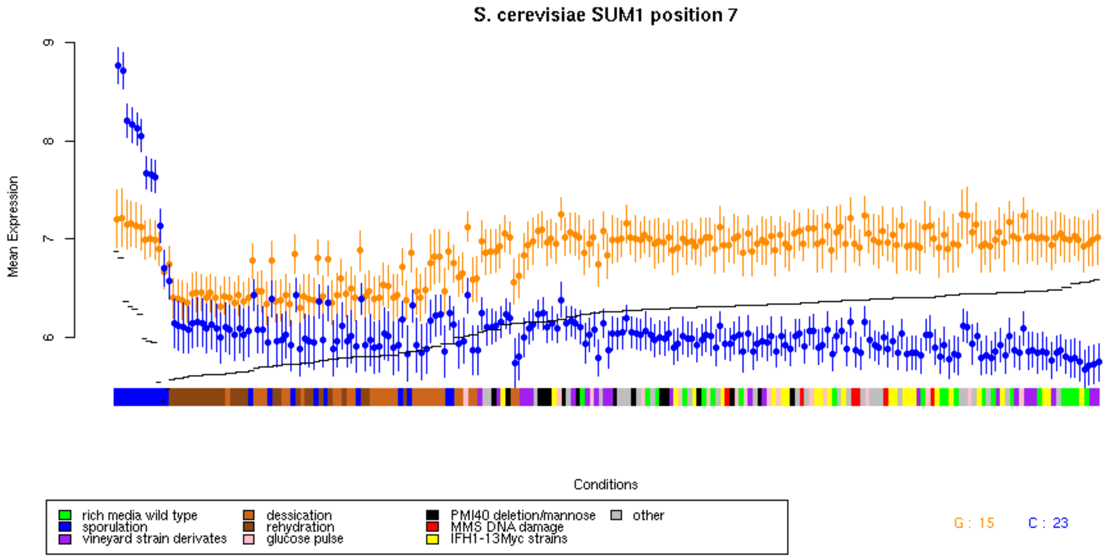
S. cerevisiae SUM1 position 7
Mean Expression
6
7
8
9
Conditions
rich media wild type
sporulation
vineyard strain derivates
dessication
rehydration
glucose pulse
PMI40 deletion/mannose
MMS DNA damage
IFH1-13Myc strains
other
G : 15
C : 23

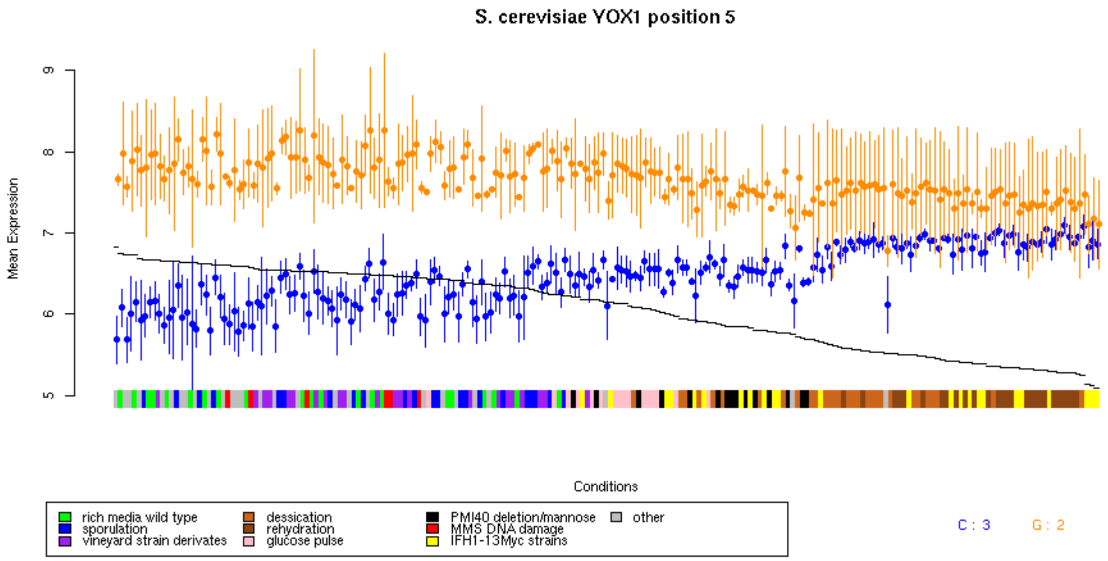
S. cerevisiae YOX1 position 5
Mean Expression
9
8
7
6
5
Conditions
rich media wild type
sporulation
vineyard strain derivates
dessication
rehydration
glucose pulse
PMI40 deletion/mannose
MMS DNA damage
IFH1-13Myc strains
other
C : 3
G : 2

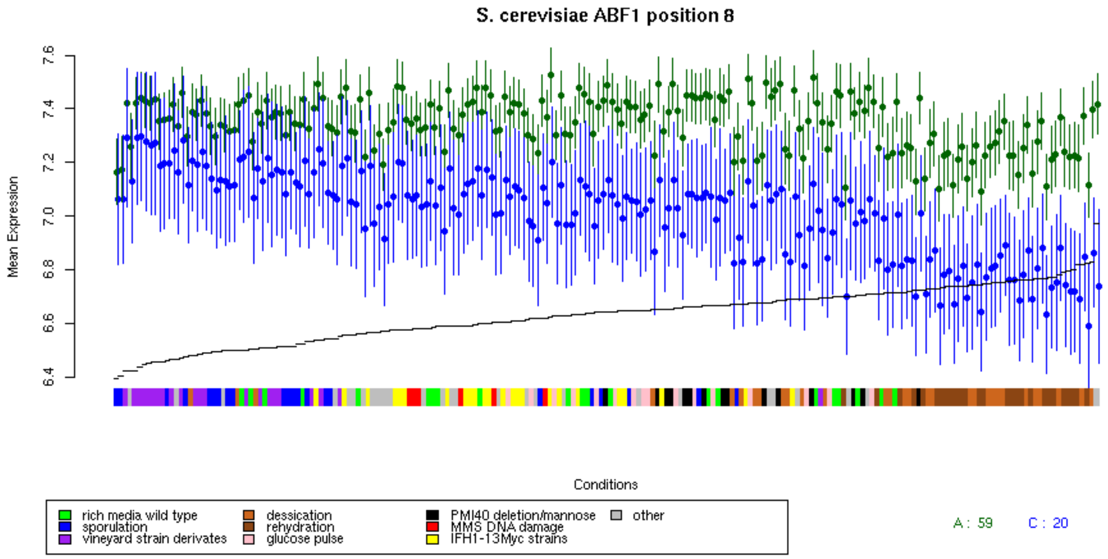
S. cerevisiae ABF1 position 8
Mean Expression
7.6
7.4
7.2
7.0
6.8
6.6
6.4
Conditions
rich media wild type
sporulation
vineyard strain derivates
dessication
rehydration
glucose pulse
PMI40 deletion/mannose
MMS DNA damage
IFH1-13Myc strains
other
A : 59
C : 20

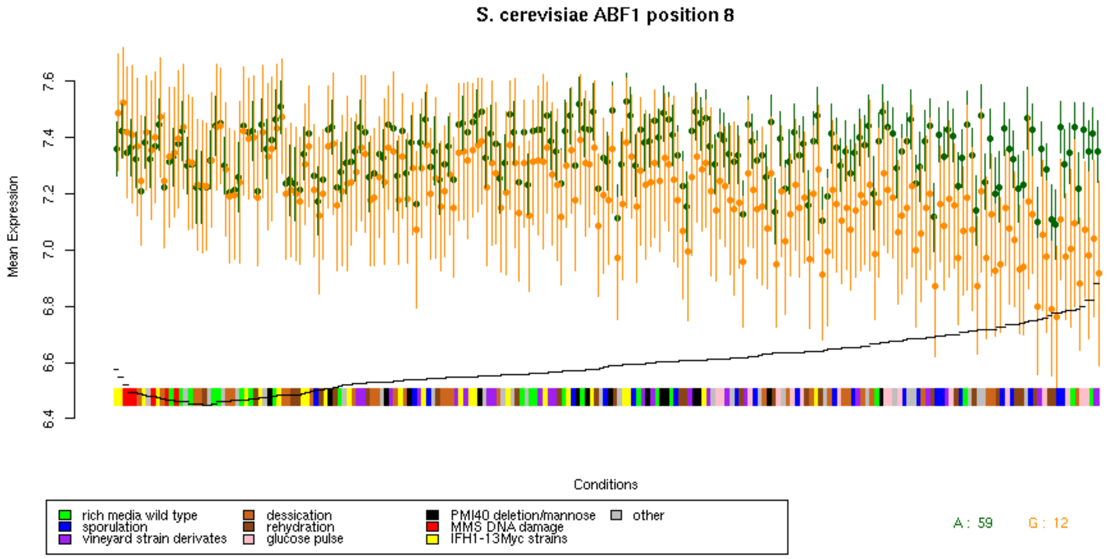
S. cerevisiae ABF1 position 8
Mean Expression
7.6
7.4
7.2
7.0
6.8
6.6
6.4
Conditions
rich media wild type
sporulation
vineyard strain derivates
dessication
rehydration
glucose pulse
PMI40 deletion/mannose
MMS DNA damage
IFH1-13Myc strains
other
A : 59
G : 12

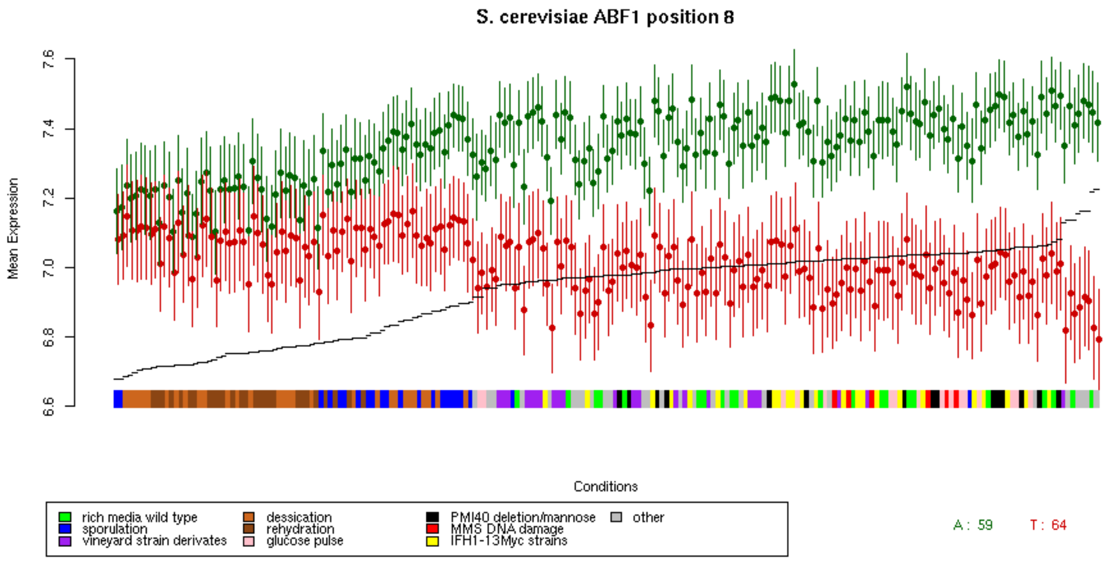
S. cerevisiae ABF1 position 8
Mean Expression
7.6
7.4
7.2
7.0
6.8
6.6
Conditions
rich media wild type
sporulation
vineyard strain derivates
dessication
rehydration
glucose pulse
PMI40 deletion/mannose
MMS DNA damage
IFH1-13Myc strains
other
A : 59
T : 64

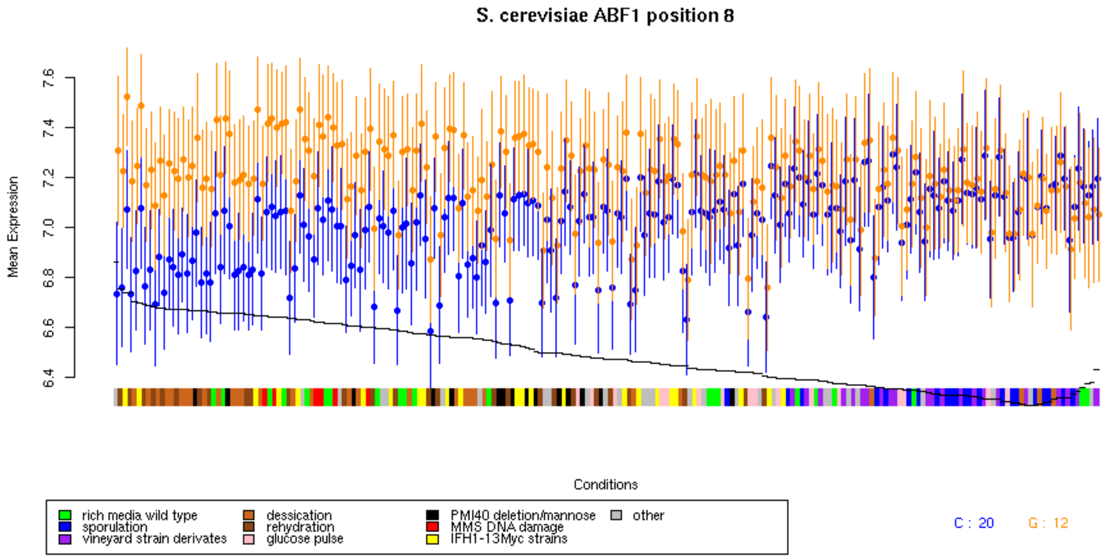
S. cerevisiae ABF1 position 8
Mean Expression
7.6
7.4
7.2
7.0
6.8
6.6
6.4
Conditions
rich media wild type
sporulation
vineyard strain derivates
dessication
rehydration
glucose pulse
PMI40 deletion/mannose
MMS DNA damage
IFH1-13Myc strains
other
C : 20
G : 12

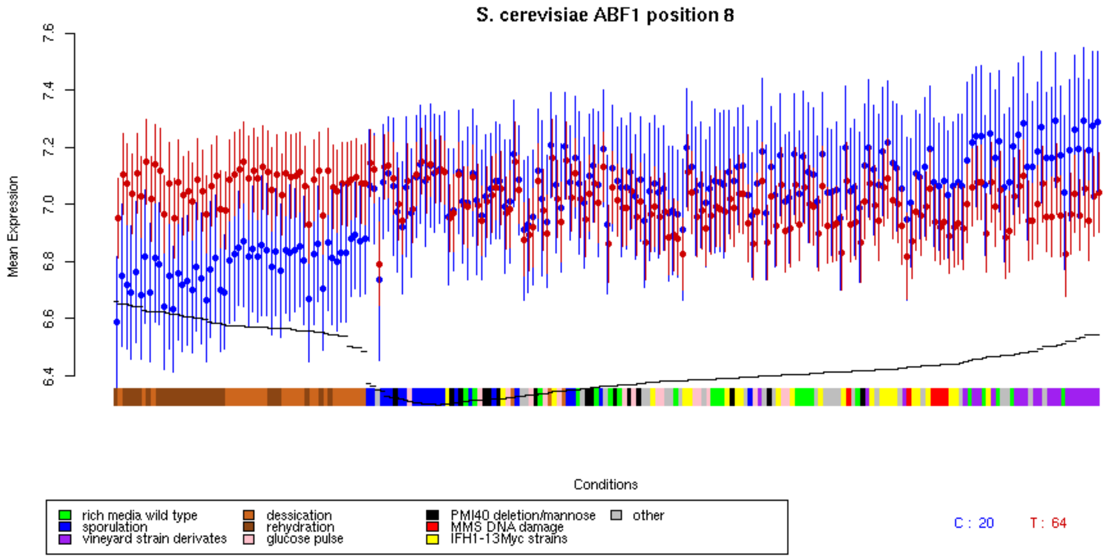
S. cerevisiae ABF1 position 8
Mean Expression
7.6
7.4
7.2
7.0
6.8
6.6
6.4
Conditions
rich media wild type
sporulation
vineyard strain derivates
dessication
rehydration
glucose pulse
PMI40 deletion/mannose
MMS DNA damage
IFH1-13Myc strains
other
C : 20
T : 64

S. cerevisiae ABF1 position 8

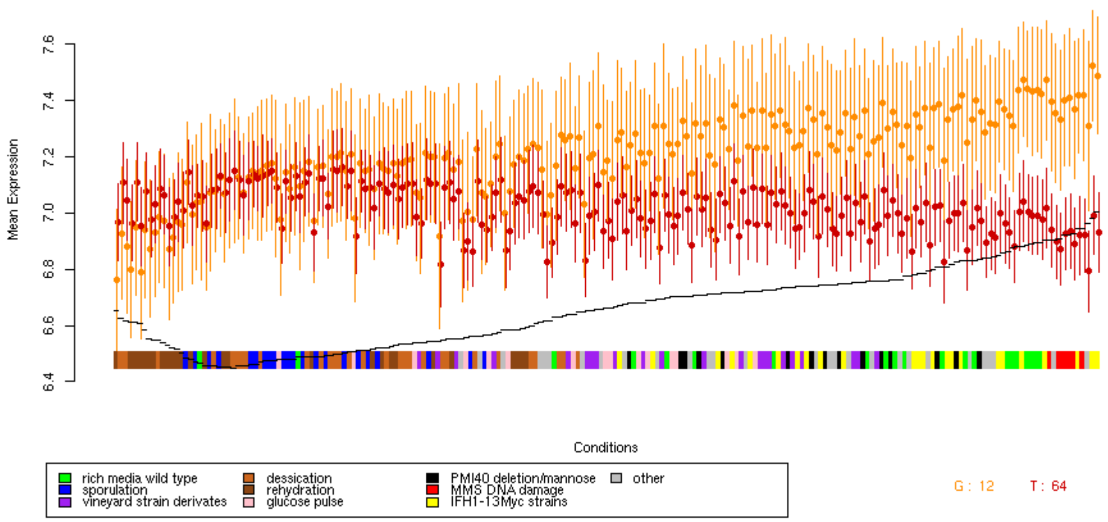

S. cerevisiae doublePAC position 1

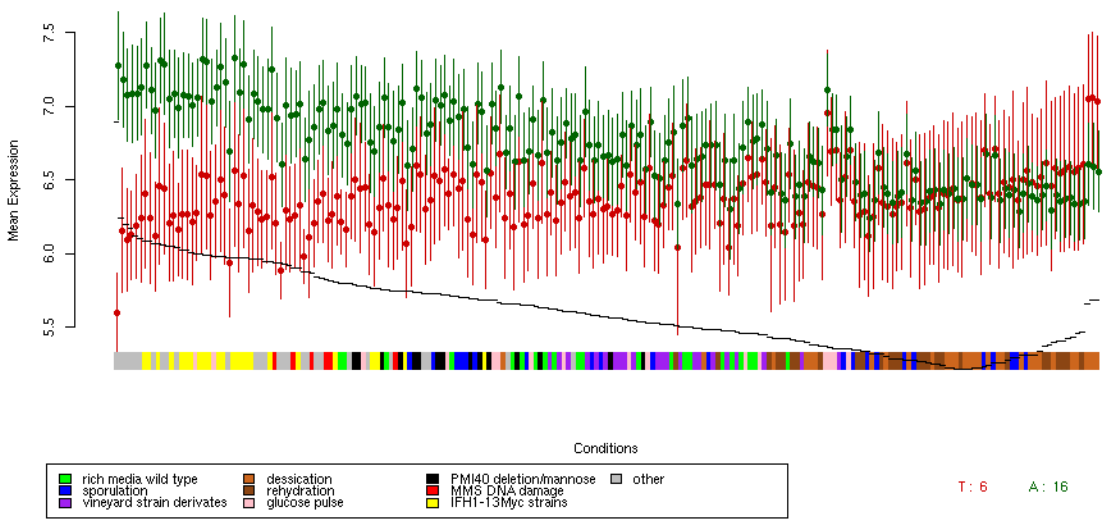

S. cerevisiae doublePAC position 11

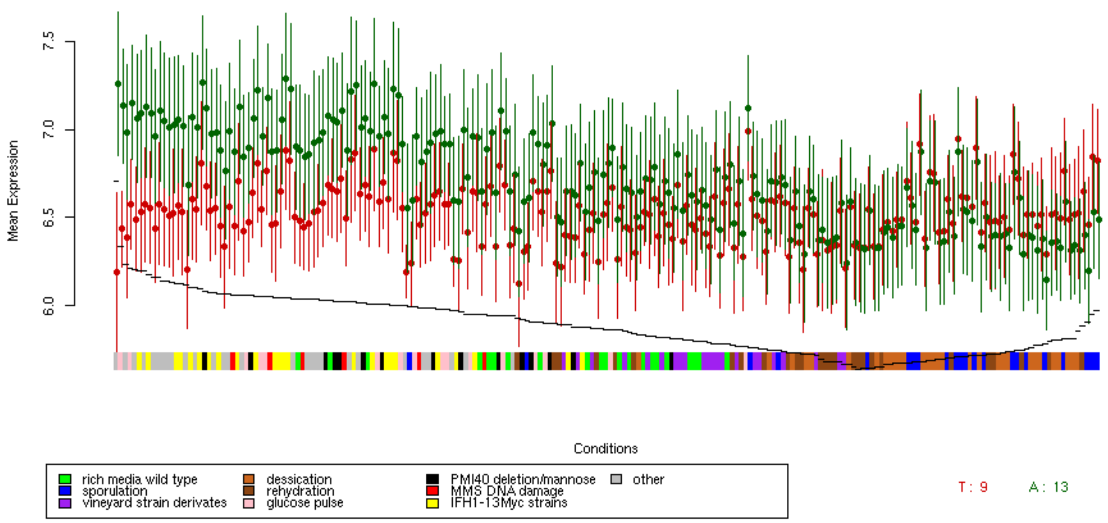

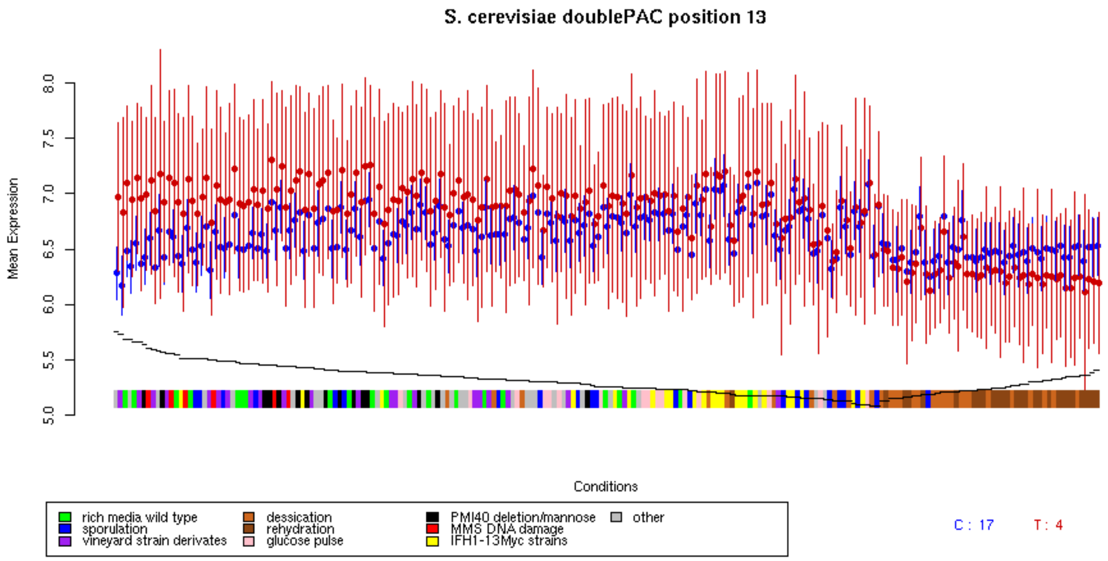
S. cerevisiae doublePAC position 13
Mean Expression
Conditions
rich media wild type
sporulation
vineyard strain derivates
dessication
rehydration
glucose pulse
PMI40 deletion/mannose
MMS DNA damage
IFH1-13Myc strains
other
C : 17
T : 4

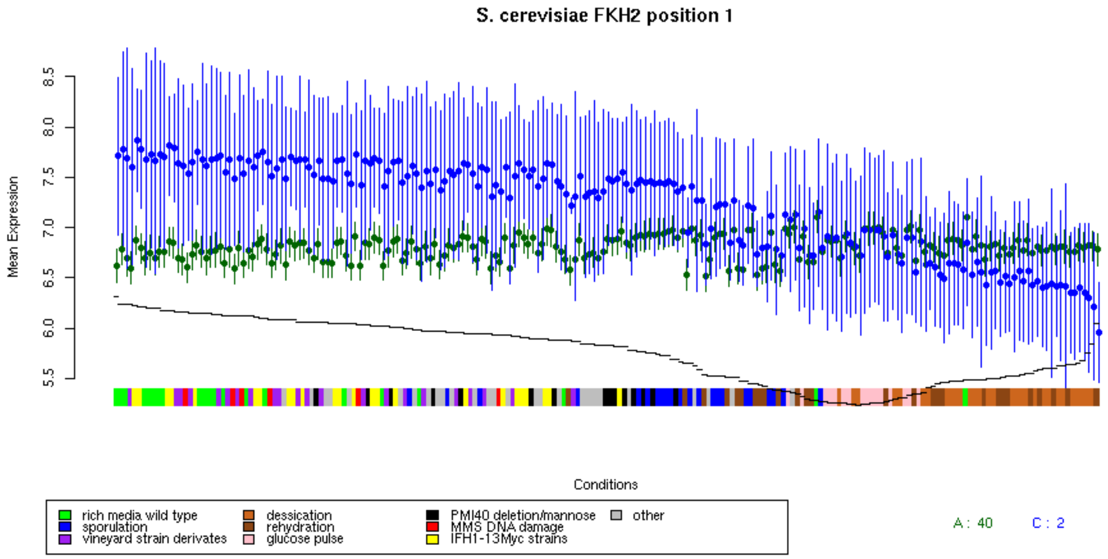
S. cerevisiae FKH2 position 1
Mean Expression
Conditions
rich media wild type
sporulation
vineyard strain derivates
dessication
rehydration
glucose pulse
PMI40 deletion/mannose
MMS DNA damage
IFH1-13Myc strains
other
A : 40
C : 2

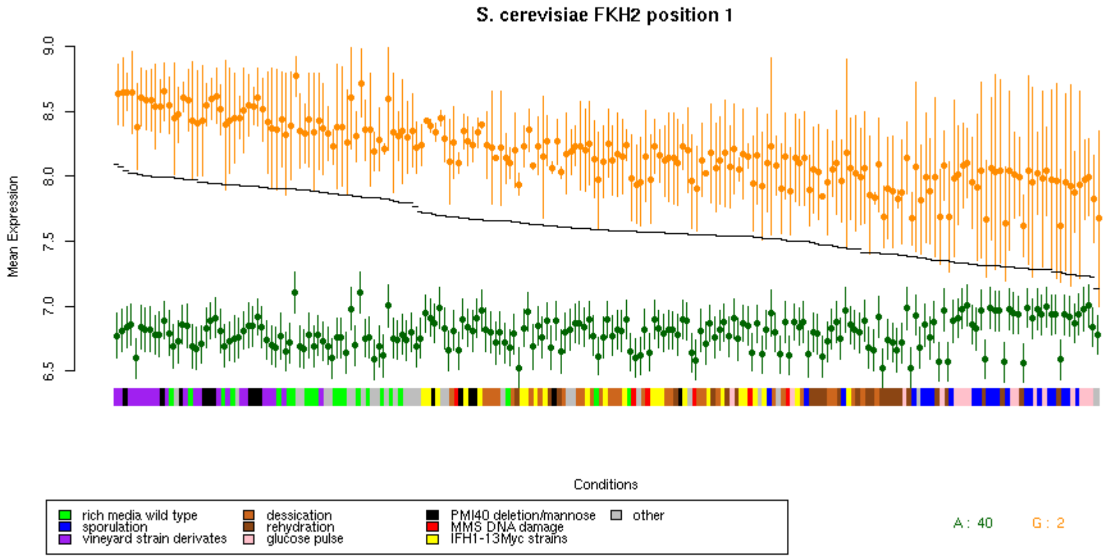
S. cerevisiae FKH2 position 1
Mean Expression
Conditions
rich media wild type
sporulation
vineyard strain derivates
dessication
rehydration
glucose pulse
PMI40 deletion/mannose
MMS DNA damage
IFH1-13Myc strains
other
A : 40
G : 2

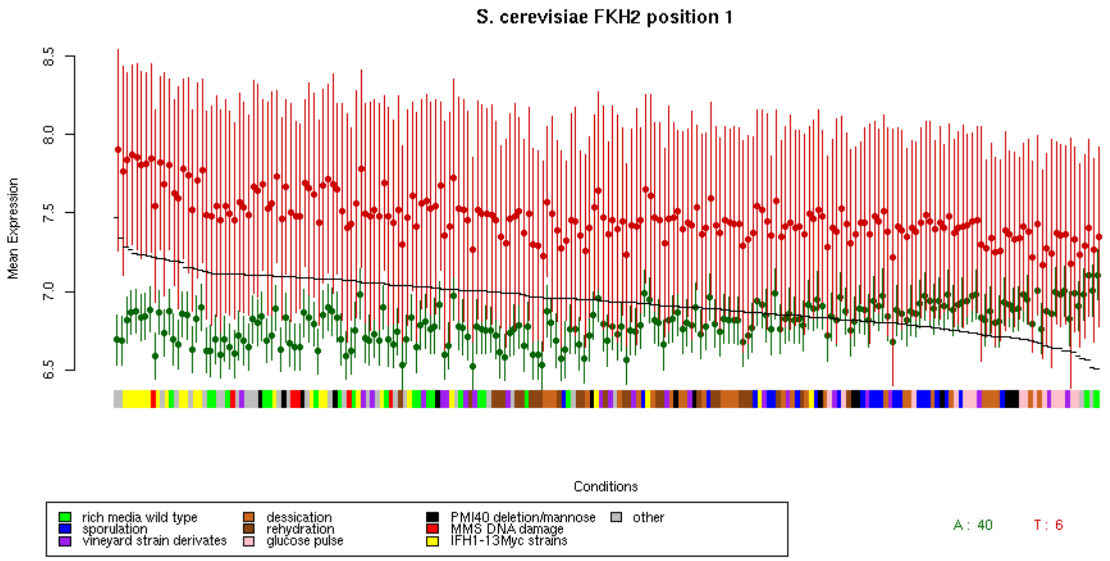
S. cerevisiae FKH2 position 1
Mean Expression
6.5
7.0
7.5
8.0
8.5
Conditions
rich media wild type
sporulation
vineyard strain derivates
dessication
rehydration
glucose pulse
PMI40 deletion/mannose
MMS DNA damage
IFH1-13Myc strains
other
A : 40
T : 6

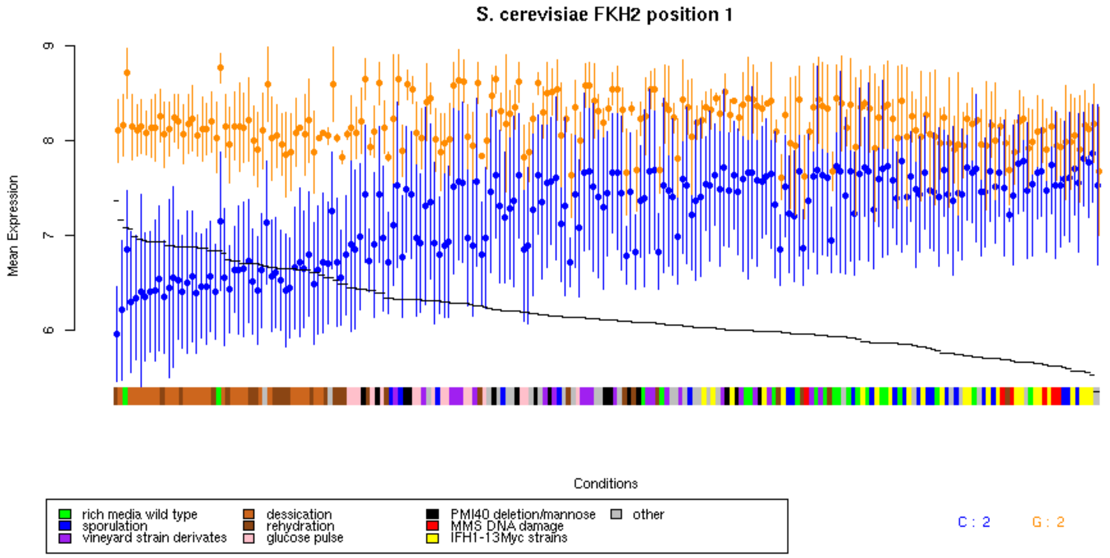
S. cerevisiae FKH2 position 1
Mean Expression
6
7
8
9
Conditions
rich media wild type
sporulation
vineyard strain derivates
dessication
rehydration
glucose pulse
PMI40 deletion/mannose
MMS DNA damage
IFH1-13Myc strains
other
C : 2
G : 2

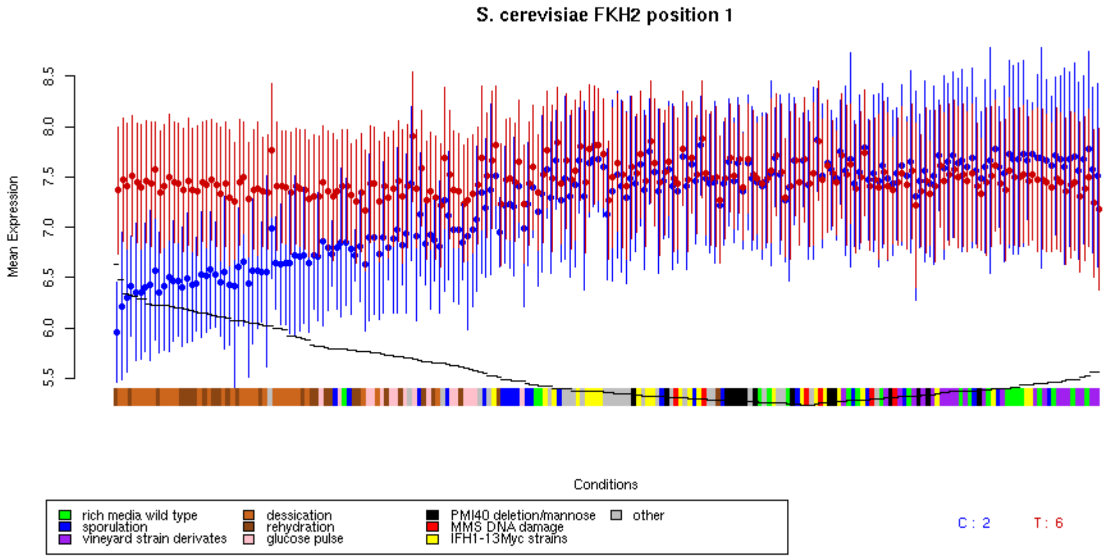
S. cerevisiae FKH2 position 1
Mean Expression
5.5
6.0
6.5
7.0
7.5
8.0
8.5
Conditions
rich media wild type
sporulation
vineyard strain derivates
dessication
rehydration
glucose pulse
PMI40 deletion/mannose
MMS DNA damage
IFH1-13Myc strains
other
C : 2
T : 6

S. cerevisiae FKH2 position 1

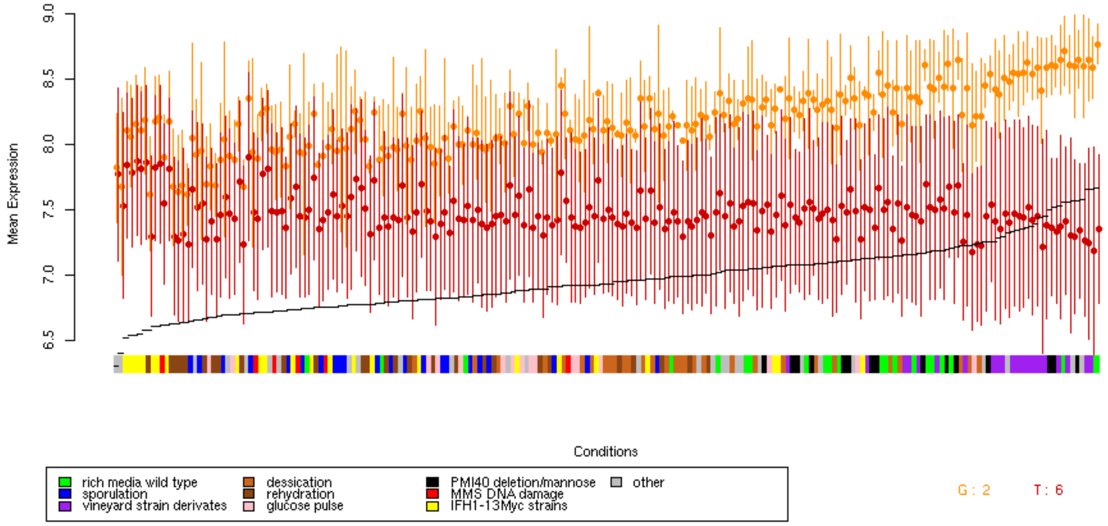

S. cerevisiae FKH2 position 6

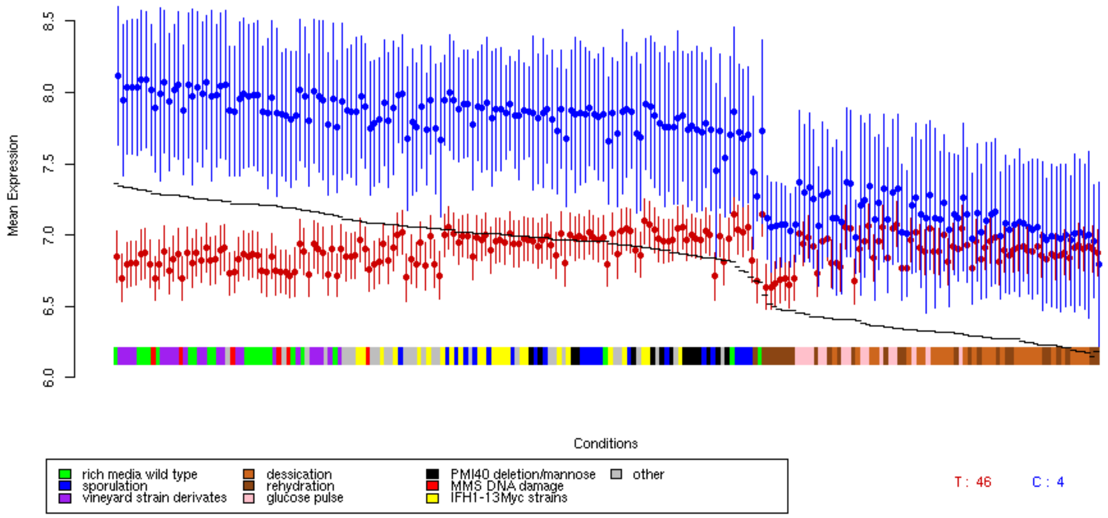

S. cerevisiae FKH2 position 10

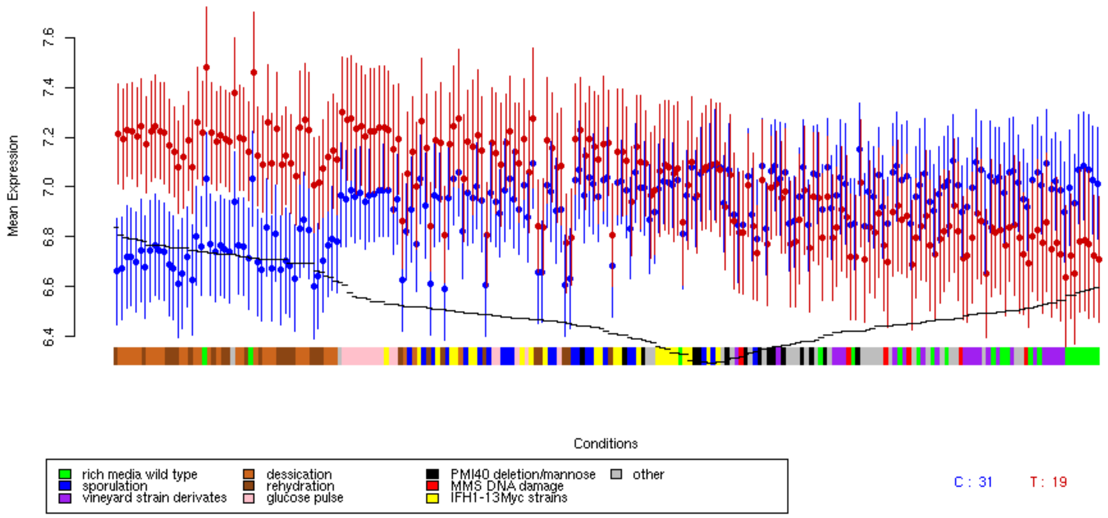

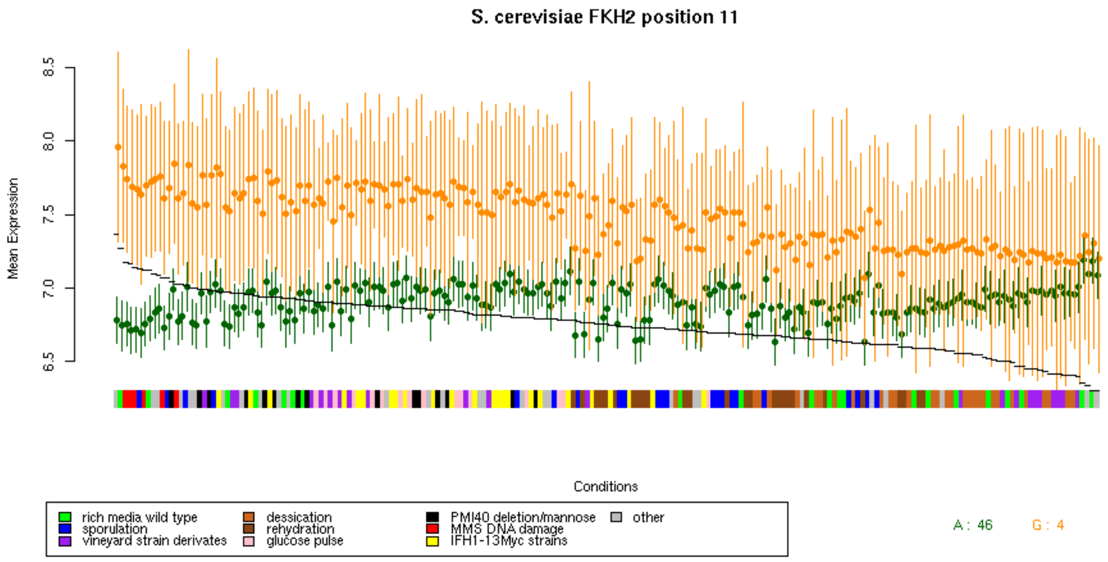
S. cerevisiae FKH2 position 11
Mean Expression
6.5
7.0
7.5
8.0
8.5
Conditions
rich media wild type
sporulation
vineyard strain derivates
dessication
rehydration
glucose pulse
PMI40 deletion/mannose
MMS DNA damage
IFH1-13Myc strains
other
A : 46
G : 4

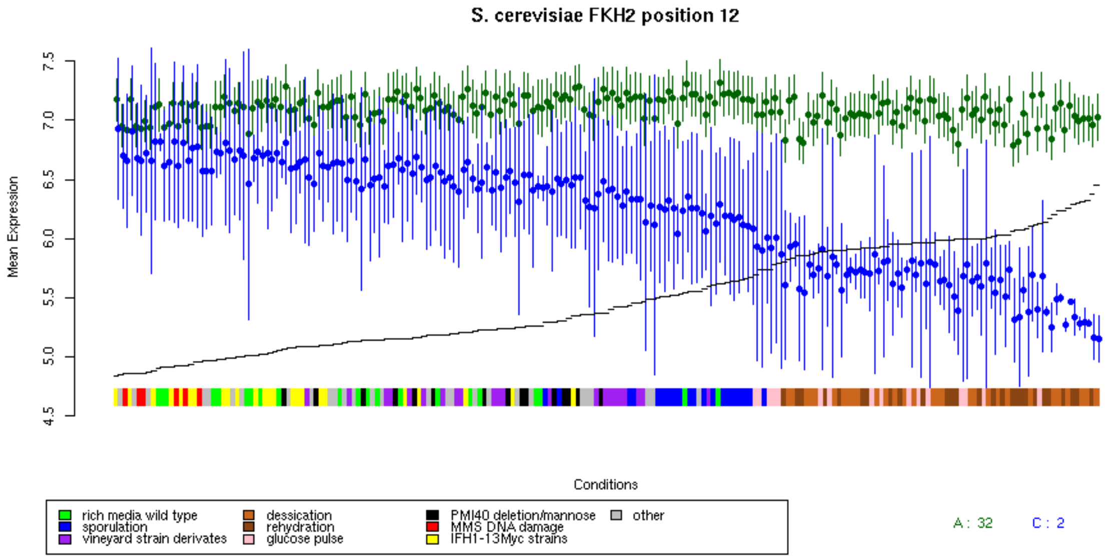
S. cerevisiae FKH2 position 12
Mean Expression
4.5
5.0
5.5
6.0
6.5
7.0
7.5
Conditions
rich media wild type
sporulation
vineyard strain derivates
dessication
rehydration
glucose pulse
PMI40 deletion/mannose
MMS DNA damage
IFH1-13Myc strains
other
A : 32
C : 2

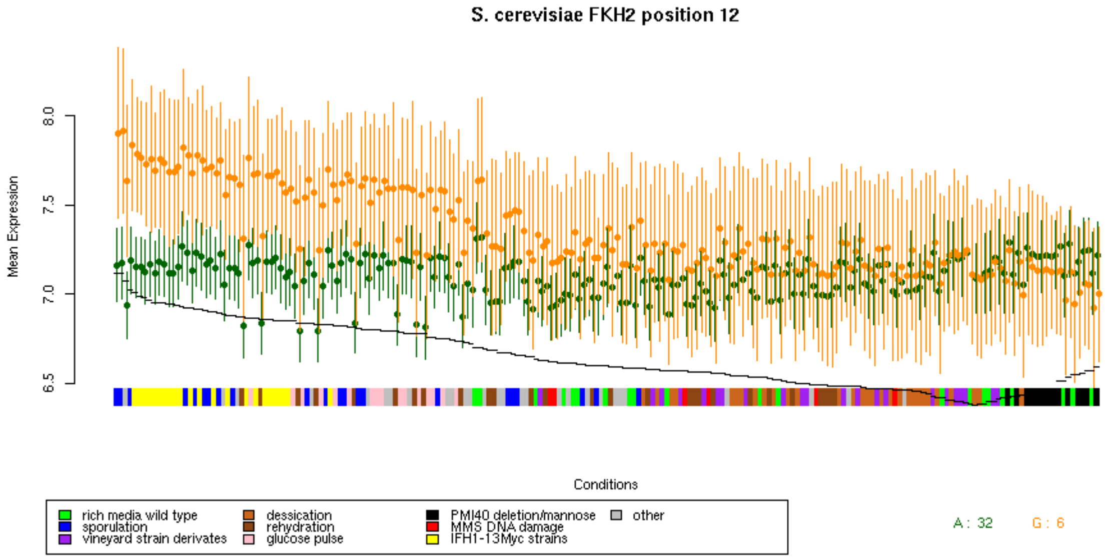
S. cerevisiae FKH2 position 12
Mean Expression
6.5
7.0
7.5
8.0
Conditions
rich media wild type
sporulation
vineyard strain derivates
dessication
rehydration
glucose pulse
PMI40 deletion/mannose
MMS DNA damage
IFH1-13Myc strains
other
A : 32
G : 6

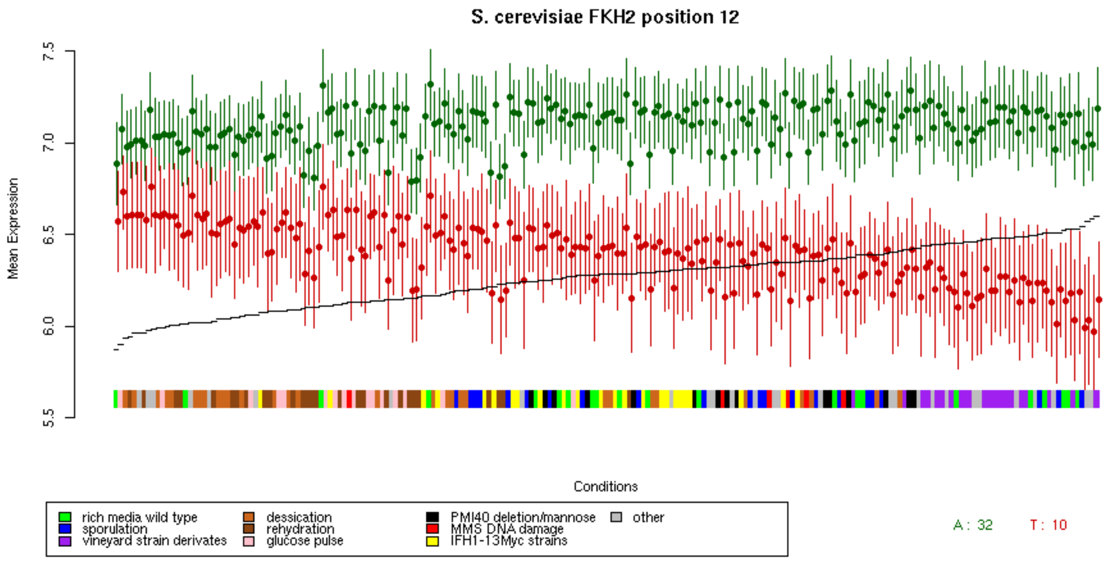
S. cerevisiae FKH2 position 12
Mean Expression
7.5
7.0
6.5
6.0
5.5
Conditions
rich media wild type
sporulation
vineyard strain derivates
dessication
rehydration
glucose pulse
PMI40 deletion/mannose
MMS DNA damage
IFH1-13Myc strains
other
A : 32
T : 10

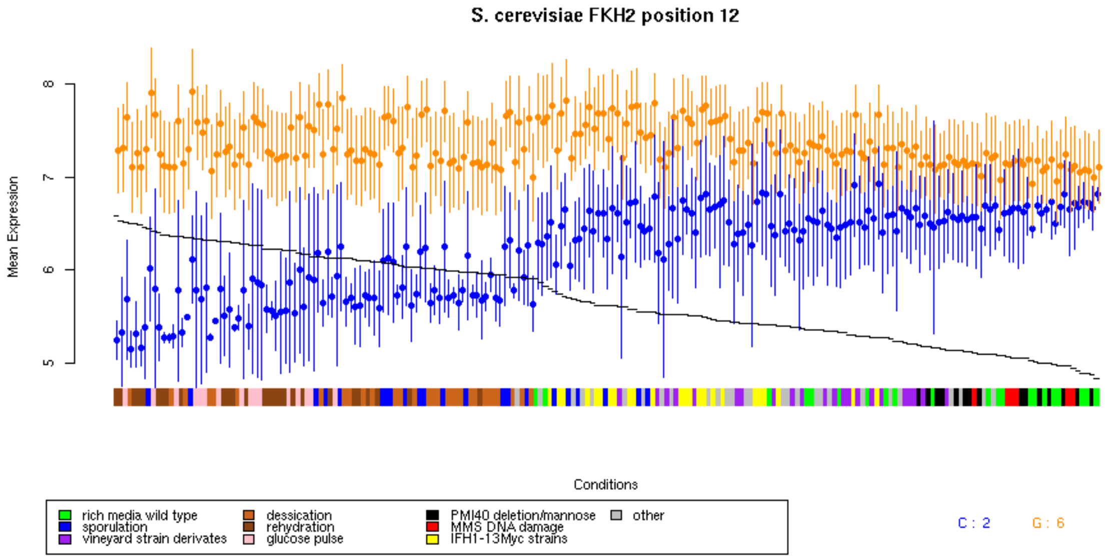
S. cerevisiae FKH2 position 12
Mean Expression
8
7
6
5
Conditions
rich media wild type
sporulation
vineyard strain derivates
dessication
rehydration
glucose pulse
PMI40 deletion/mannose
MMS DNA damage
IFH1-13Myc strains
other
C : 2
G : 6

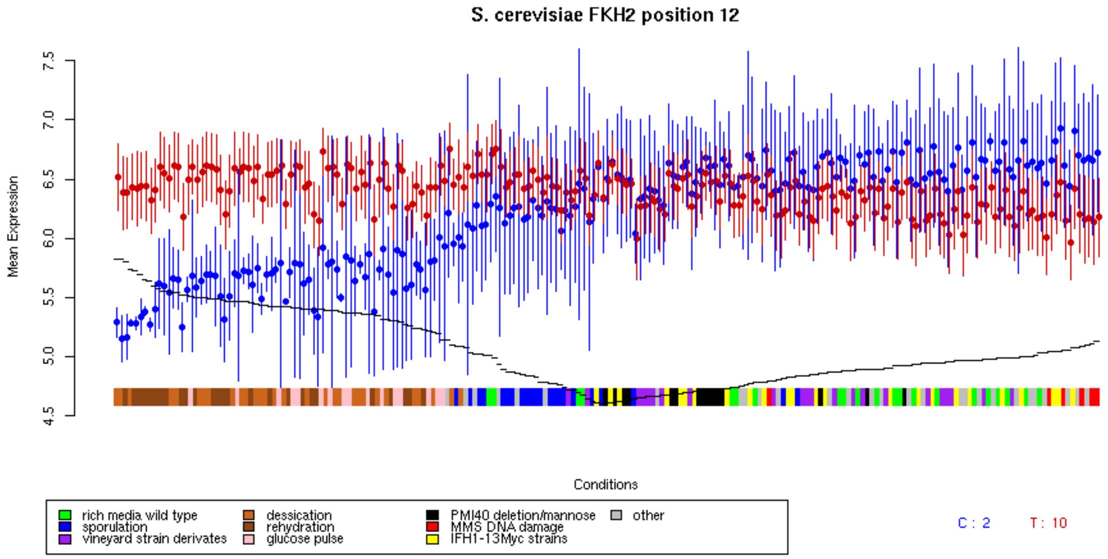
S. cerevisiae FKH2 position 12
Mean Expression
7.5
7.0
6.5
6.0
5.5
5.0
4.5
Conditions
rich media wild type
sporulation
vineyard strain derivates
dessication
rehydration
glucose pulse
PMI40 deletion/mannose
MMS DNA damage
IFH1-13Myc strains
other
C : 2
T : 10

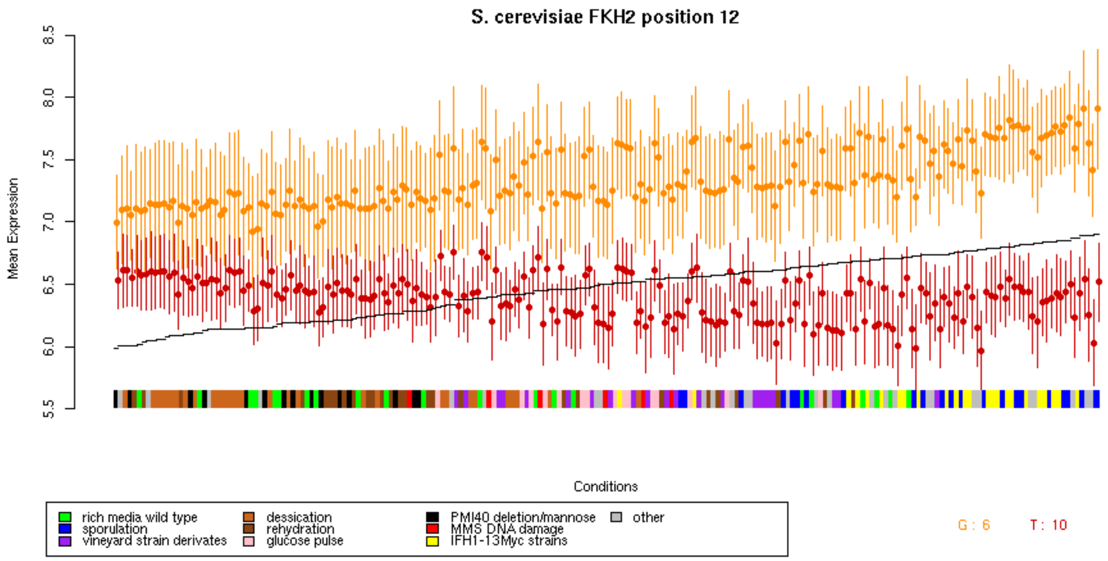
S. cerevisiae FKH2 position 12
Mean Expression
Conditions
rich media wild type
sporulation
vineyard strain derivates
dessication
rehydration
glucose pulse
PMI40 deletion/mannose
MMS DNA damage
IFH1-13Myc strains
other
G : 6
T : 10

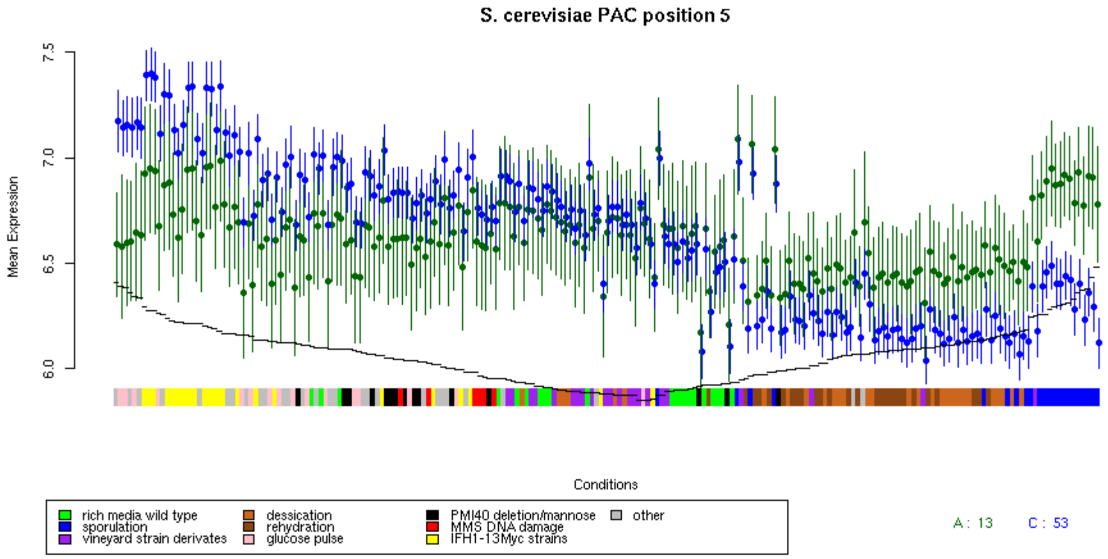
S. cerevisiae PAC position 5
Mean Expression
Conditions
rich media wild type
sporulation
vineyard strain derivates
dessication
rehydration
glucose pulse
PMI40 deletion/mannose
MMS DNA damage
IFH1-13Myc strains
other
A : 13
C : 53

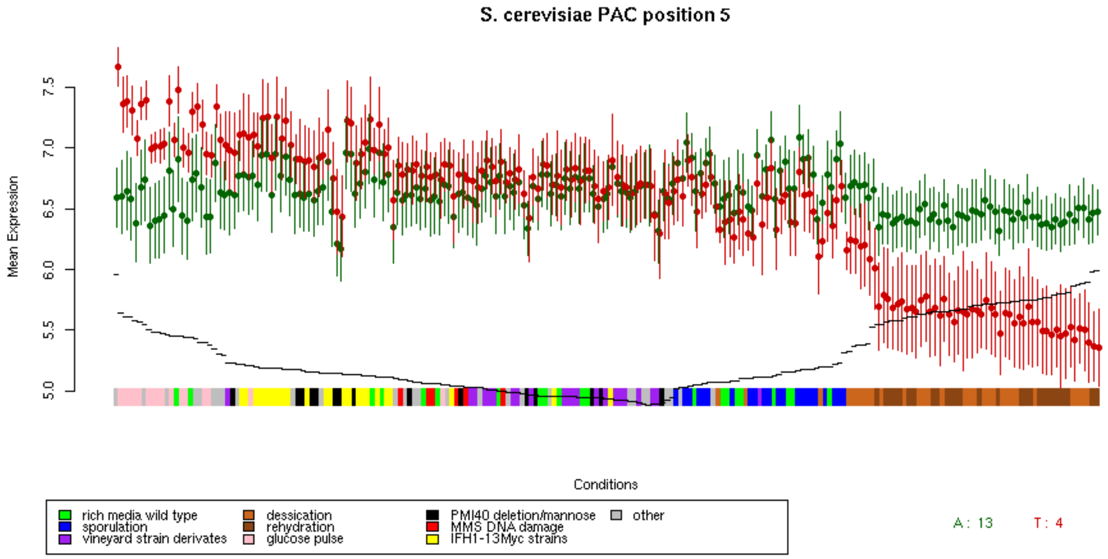
S. cerevisiae PAC position 5
Mean Expression
Conditions
rich media wild type
sporulation
vineyard strain derivates
dessication
rehydration
glucose pulse
PMI40 deletion/mannose
MMS DNA damage
IFH1-13Myc strains
other
A : 13
T : 4

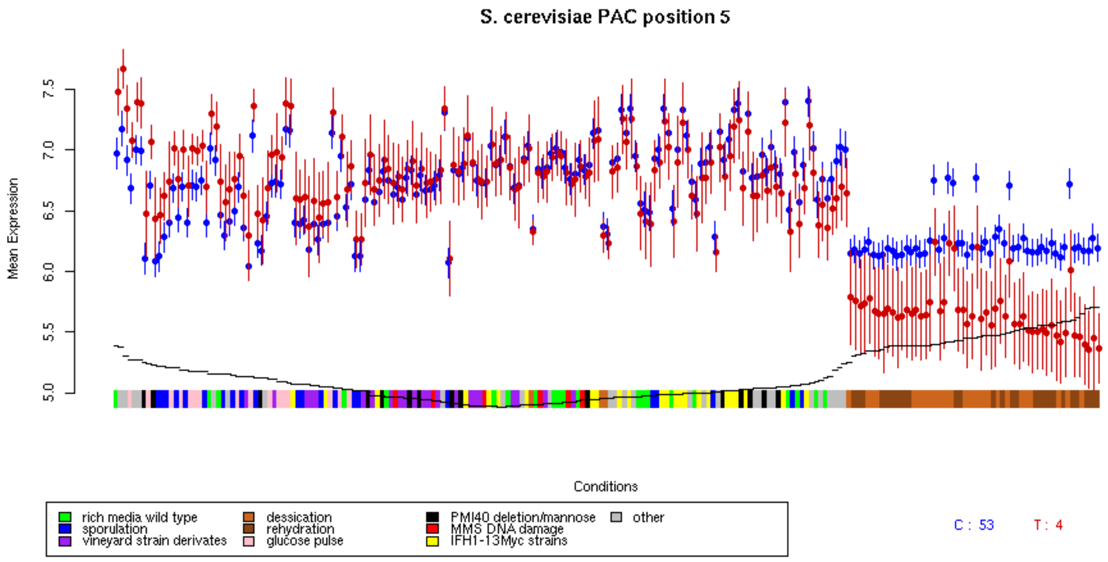
S. cerevisiae PAC position 5
Mean Expression
Conditions
rich media wild type
sporulation
vineyard strain derivates
dessication
rehydration
glucose pulse
PMI40 deletion/mannose
MMS DNA damage
IFH1-13Myc strains
other
C : 53
T : 4

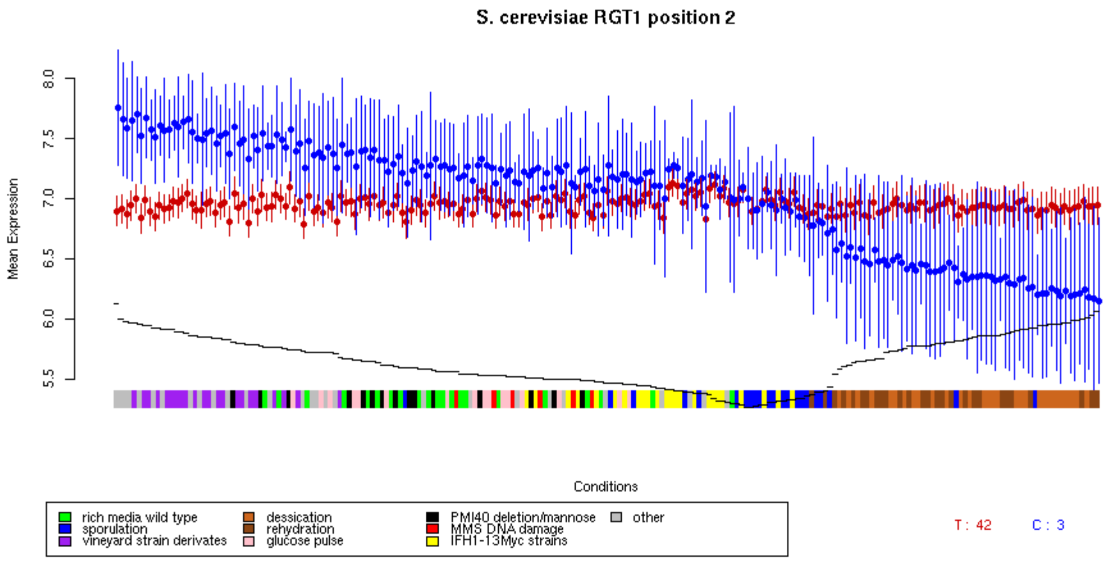
S. cerevisiae RGT1 position 2
Mean Expression
Conditions
rich media wild type
sporulation
vineyard strain derivates
dessication
rehydration
glucose pulse
PMI40 deletion/mannose
MMS DNA damage
IFH1-13Myc strains
other
T : 42
C : 3

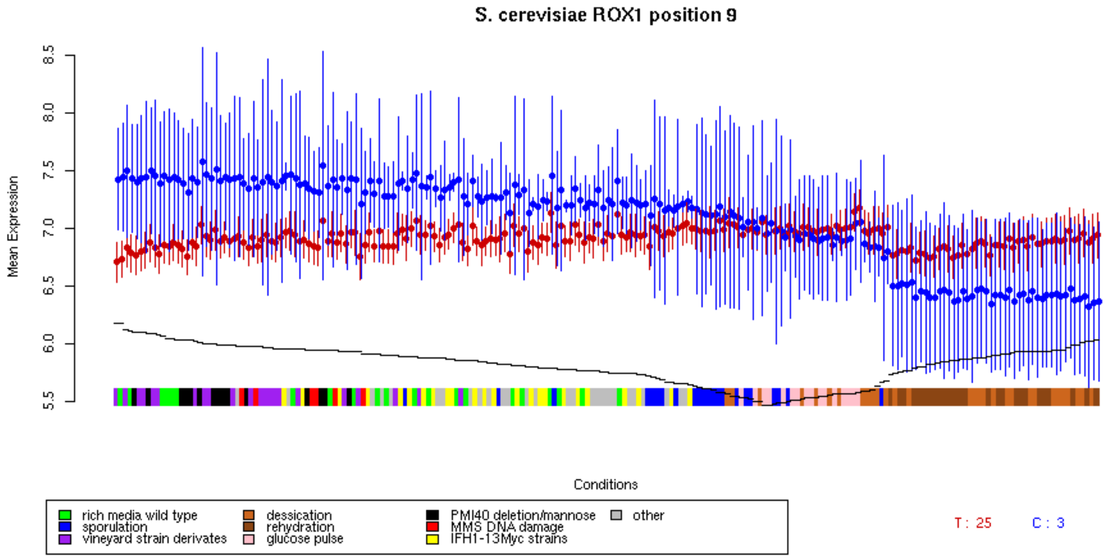
S. cerevisiae ROX1 position 9
Mean Expression
Conditions
rich media wild type
sporulation
vineyard strain derivates
dessication
rehydration
glucose pulse
PMI40 deletion/mannose
MMS DNA damage
IFH1-13Myc strains
other
T : 25
C : 3

S. cerevisiae RPN4 position 10

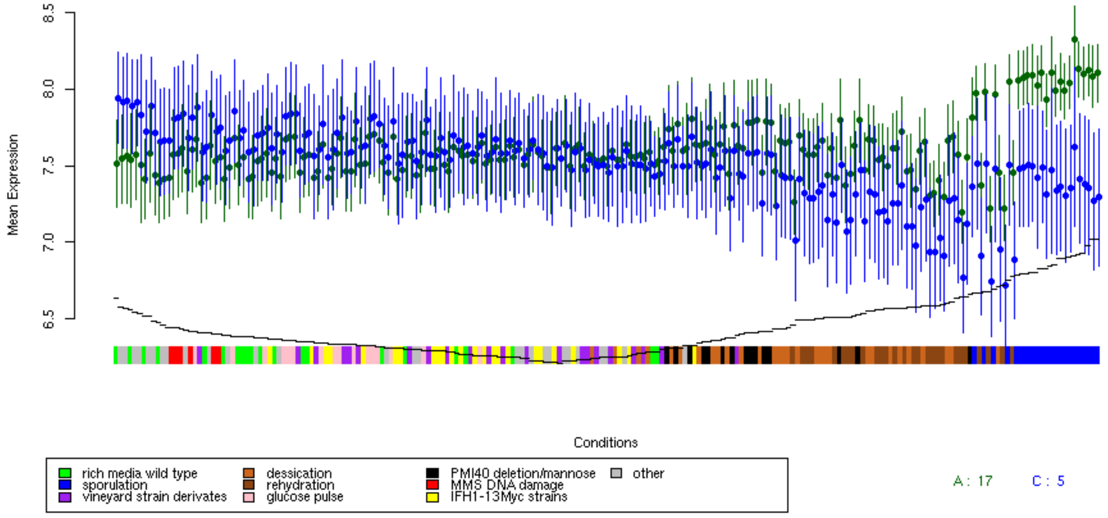

S. cerevisiae RPN4 position 10

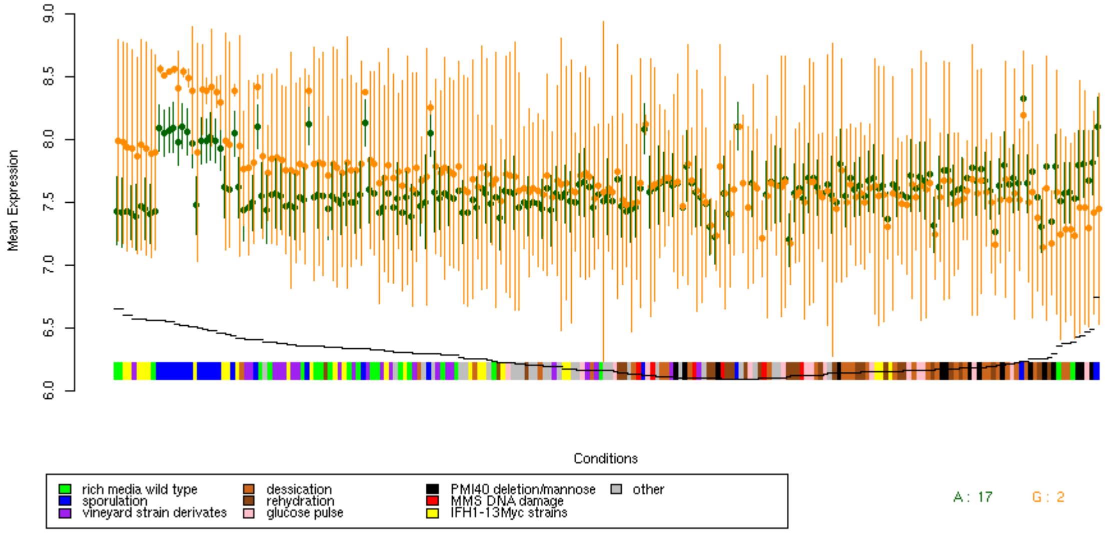

S. cerevisiae RPN4 position 10

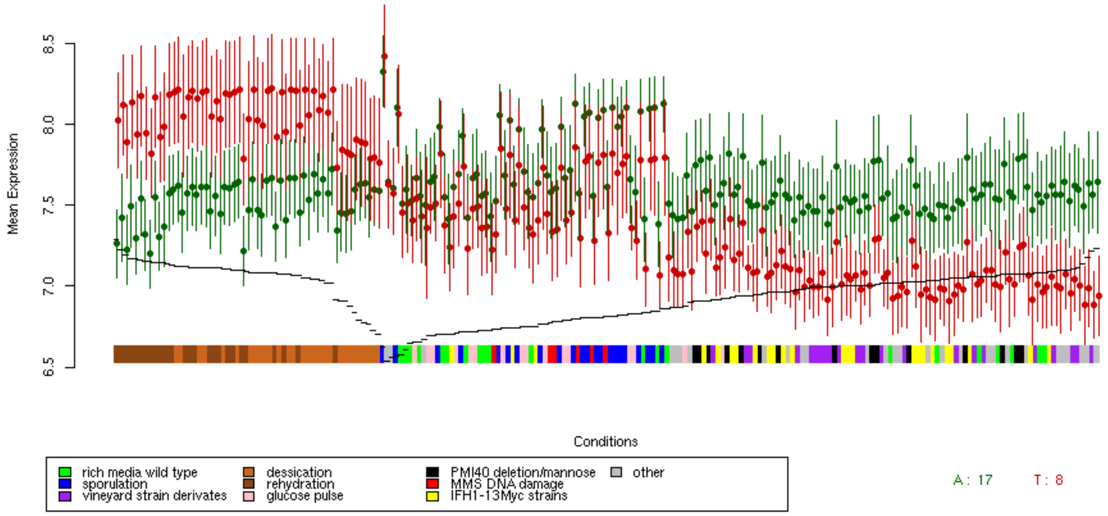

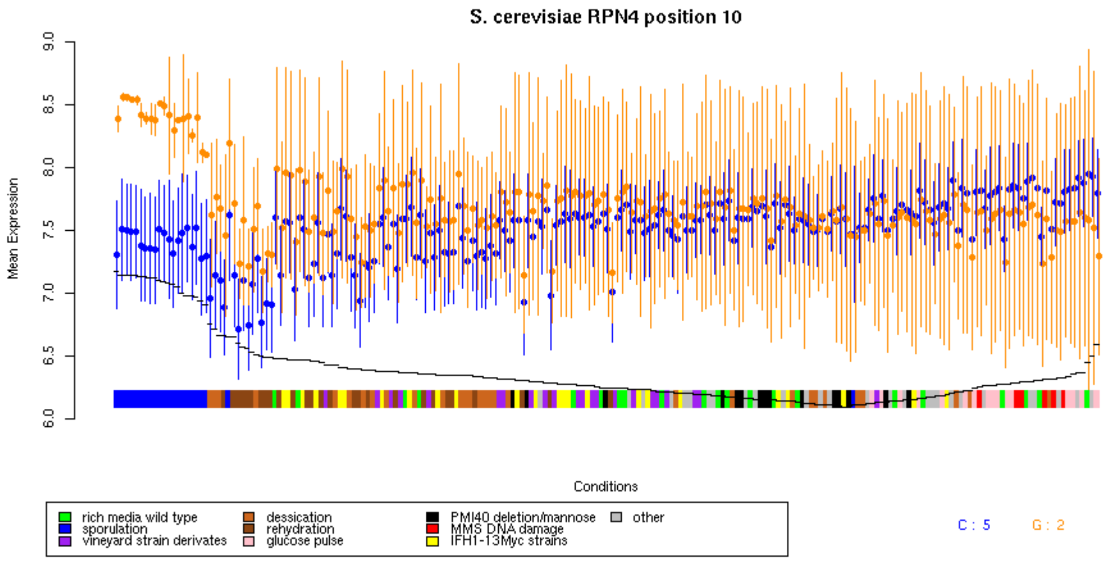
S. cerevisiae RPN4 position 10
Mean Expression
9.0
8.5
8.0
7.5
7.0
6.5
6.0
Conditions
rich media wild type
sporulation
vineyard strain derivates
dessication
rehydration
glucose pulse
PMI40 deletion/mannose
MMS DNA damage
IFH1-13Myc strains
other
C : 5
G : 2

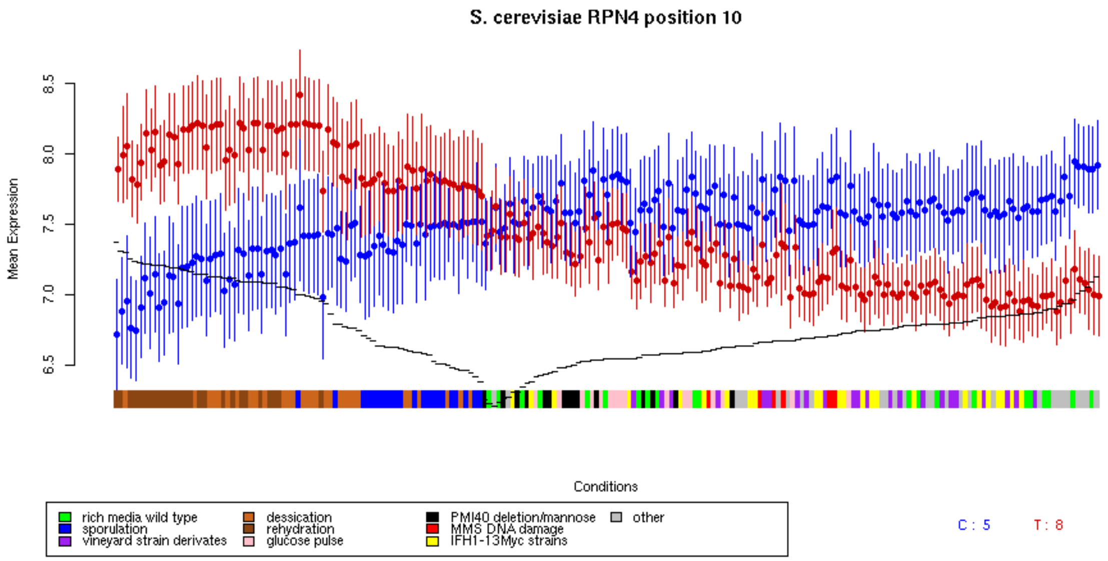
S. cerevisiae RPN4 position 10
Mean Expression
8.5
8.0
7.5
7.0
6.5
Conditions
rich media wild type
sporulation
vineyard strain derivates
dessication
rehydration
glucose pulse
PMI40 deletion/mannose
MMS DNA damage
IFH1-13Myc strains
other
C : 5
T : 8

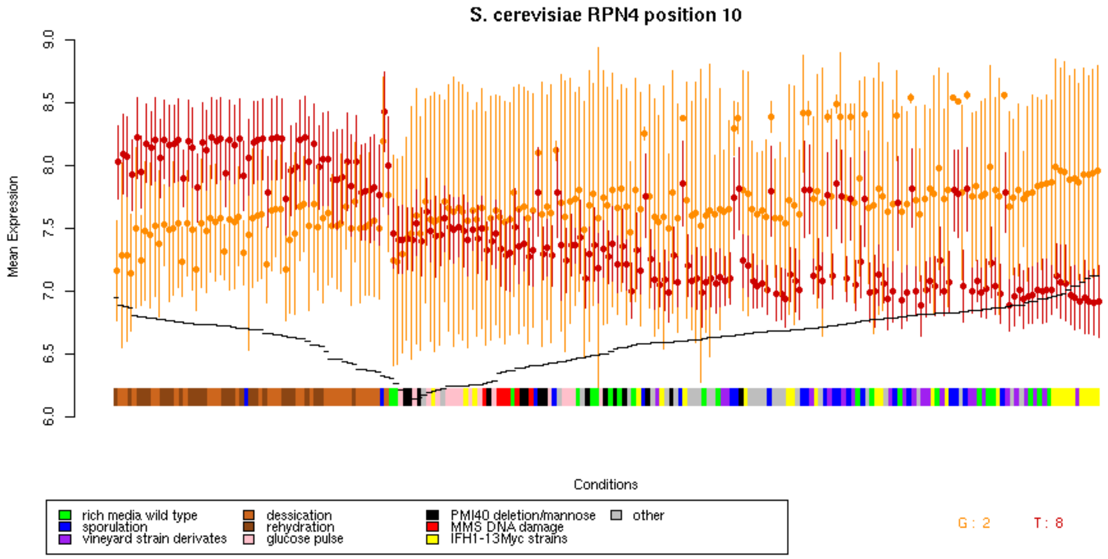
S. cerevisiae RPN4 position 10
Mean Expression
9.0
8.5
8.0
7.5
7.0
6.5
6.0
Conditions
rich media wild type
sporulation
vineyard strain derivates
dessication
rehydration
glucose pulse
PMI40 deletion/mannose
MMS DNA damage
IFH1-13Myc strains
other
G : 2
T : 8

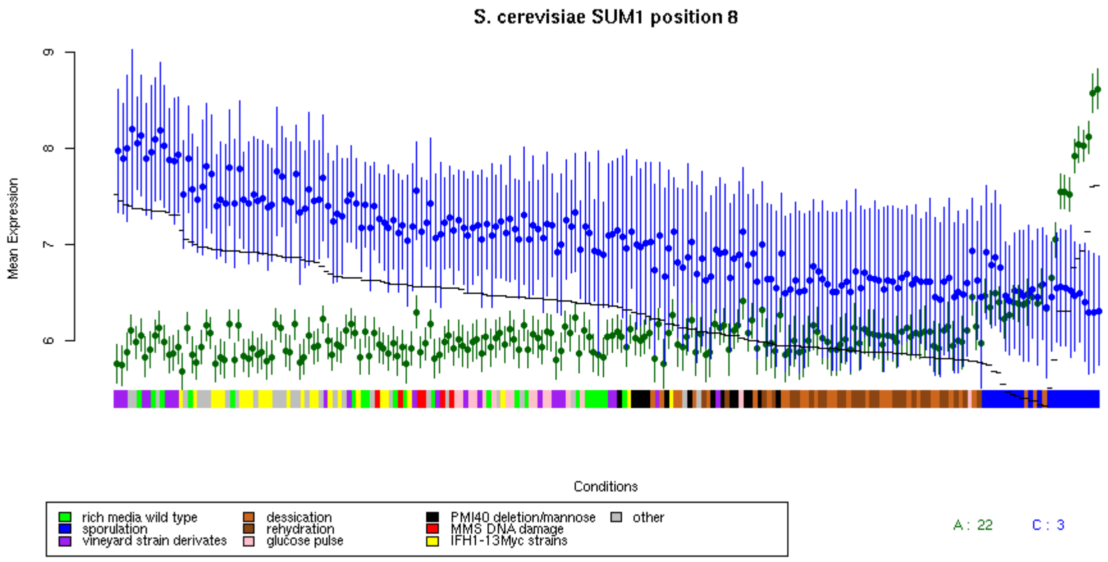
S. cerevisiae SUM1 position 8
Mean Expression
Conditions
rich media wild type
sporulation
vineyard strain derivates
dessication
rehydration
glucose pulse
PMI40 deletion/mannose
MMS DNA damage
IFH1-13Myc strains
other
A : 22
C : 3

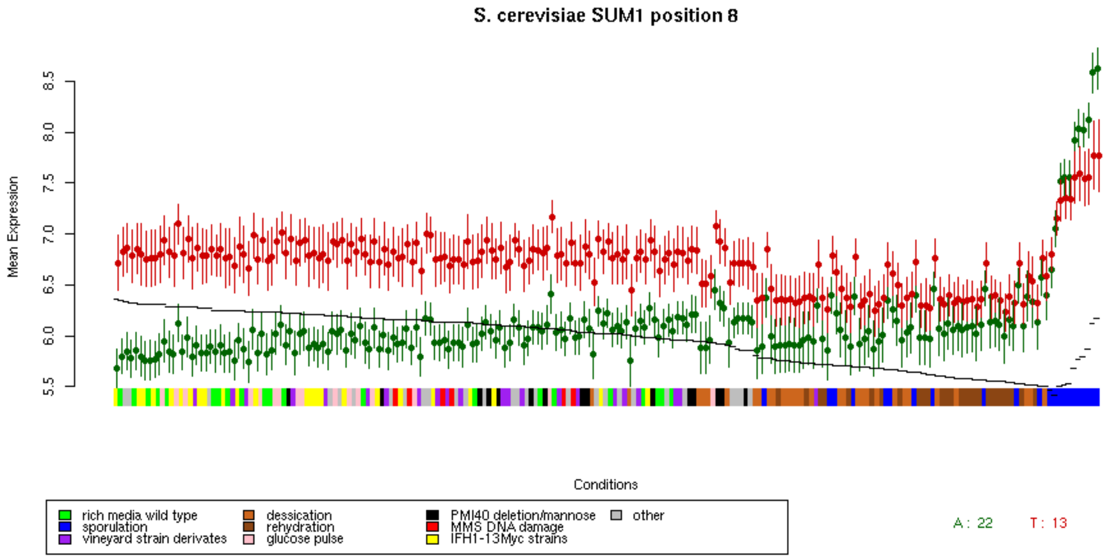
S. cerevisiae SUM1 position 8
Mean Expression
Conditions
rich media wild type
sporulation
vineyard strain derivates
dessication
rehydration
glucose pulse
PMI40 deletion/mannose
MMS DNA damage
IFH1-13Myc strains
other
A : 22
T : 13

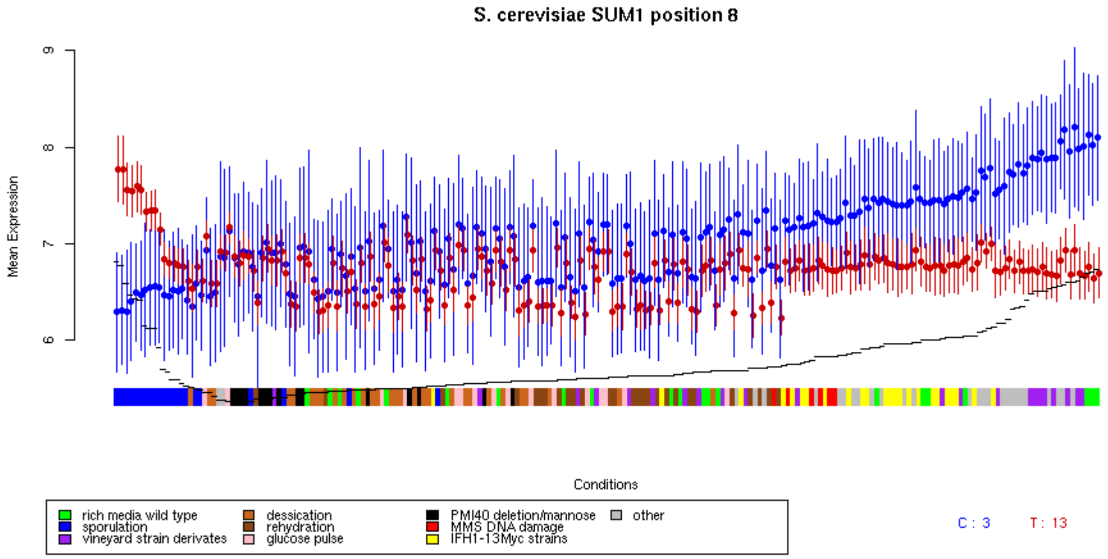
S. cerevisiae SUM1 position 8
Mean Expression
Conditions
rich media wild type
sporulation
vineyard strain derivates
dessication
rehydration
glucose pulse
PMI40 deletion/mannose
MMS DNA damage
IFH1-13Myc strains
other
C : 3
T : 13

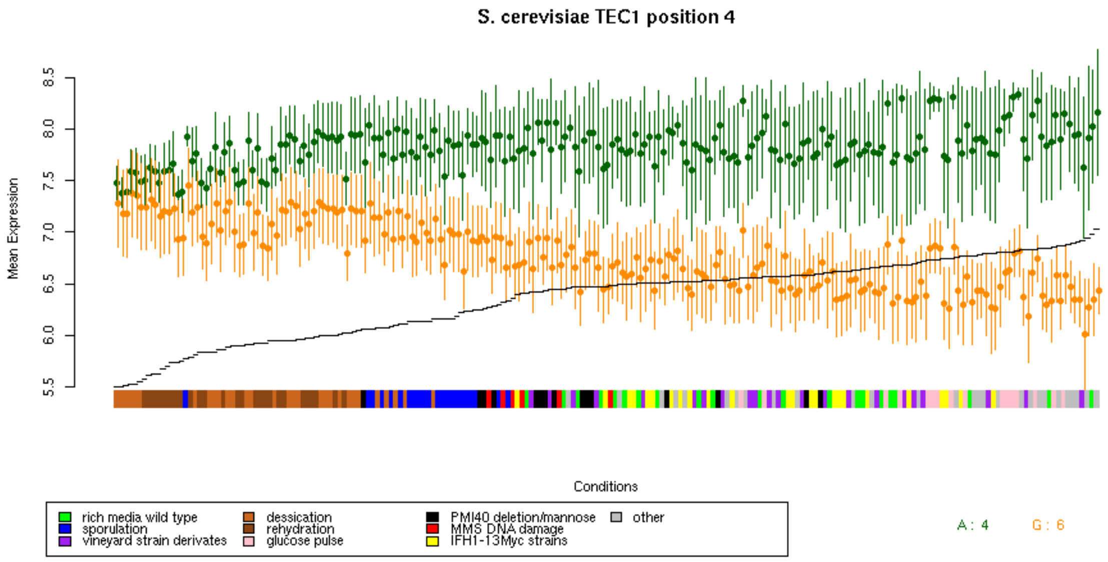
S. cerevisiae TEC1 position 4
Mean Expression
8.5
8.0
7.5
7.0
6.5
6.0
5.5
Conditions
rich media wild type
sporulation
vineyard strain derivates
dessication
rehydration
glucose pulse
PMI40 deletion/mannose
MMS DNA damage
IFH1-13Myc strains
other
A : 4
G : 6

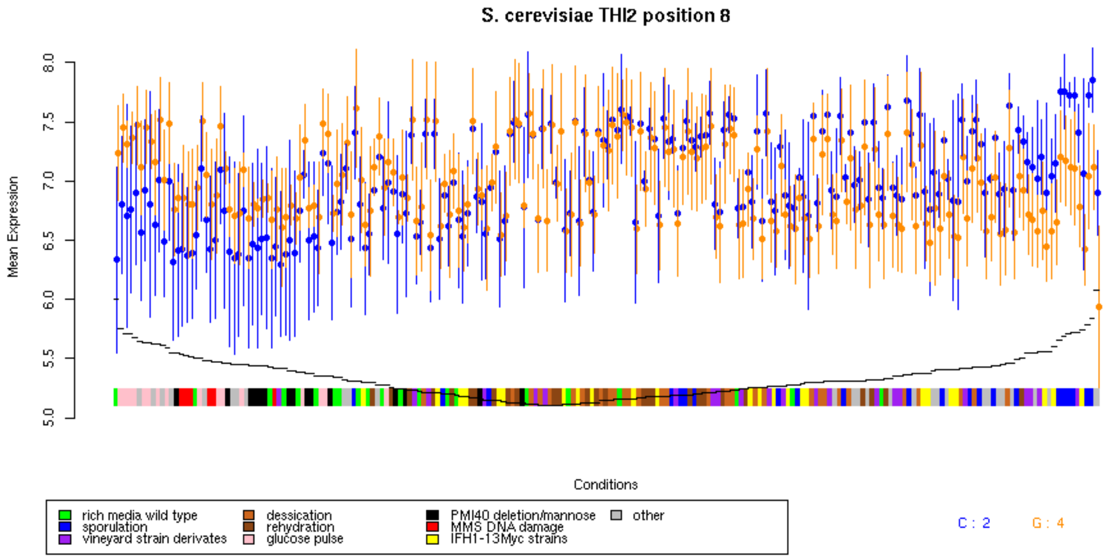
S. cerevisiae THI2 position 8
Mean Expression
8.0
7.5
7.0
6.5
6.0
5.5
5.0
Conditions
rich media wild type
sporulation
vineyard strain derivates
dessication
rehydration
glucose pulse
PMI40 deletion/mannose
MMS DNA damage
IFH1-13Myc strains
other
C : 2
G : 4

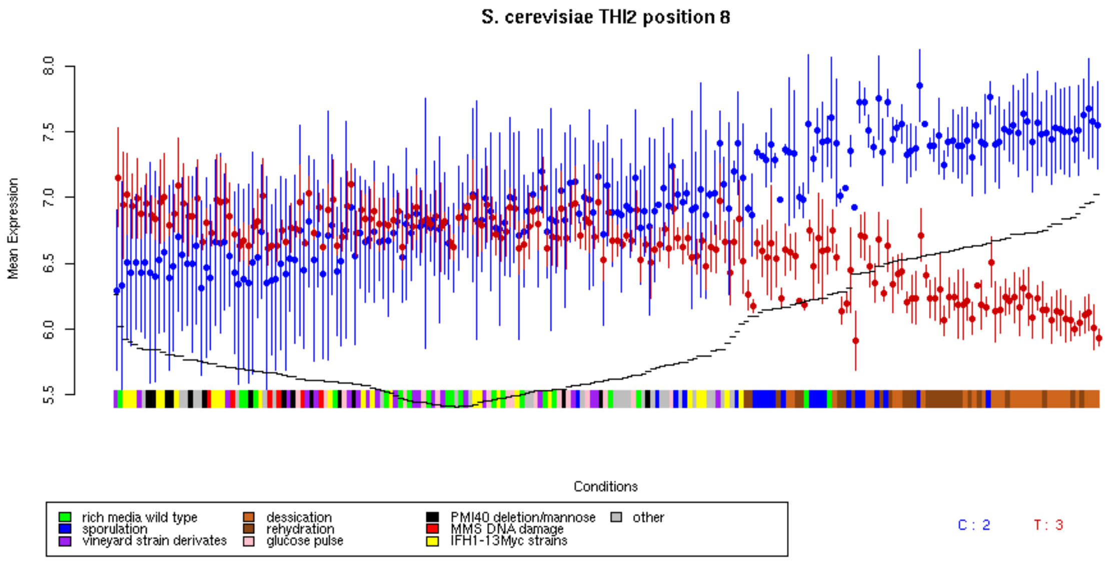
S. cerevisiae THI2 position 8
Mean Expression
8.0
7.5
7.0
6.5
6.0
5.5
Conditions
rich media wild type
sporulation
vineyard strain derivates
dessication
rehydration
glucose pulse
PMI40 deletion/mannose
MMS DNA damage
IFH1-13Myc strains
other
C : 2
T : 3

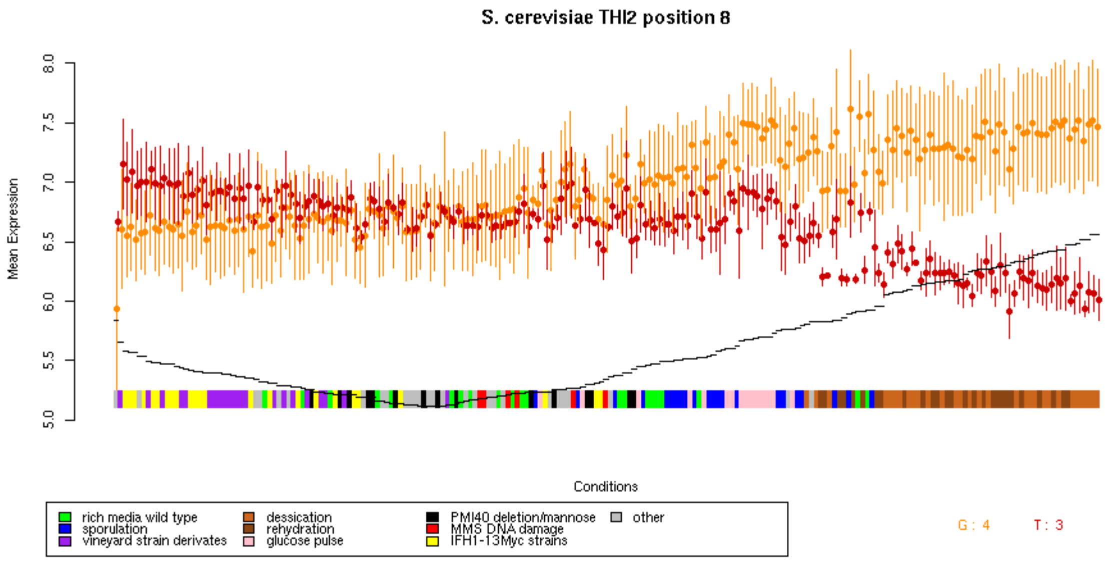
S. cerevisiae THI2 position 8
Mean Expression
8.0
7.5
7.0
6.5
6.0
5.5
5.0
Conditions
rich media wild type
sporulation
vineyard strain derivates
dessication
rehydration
glucose pulse
PMI40 deletion/mannose
MMS DNA damage
IFH1-13Myc strains
other
G : 4
T : 3
