# Supplementary material for: Contribution of Transcription Factor Binding Site Motif Variants to Condition-Specific Gene Expression Patterns in Budding Yeast
Source: PLoS One. 2012 Feb 23;7(2):e32274. doi: 10.1371/journal.pone.0032274 (PMC3285675; doi:10.1371/journal.pone.0032274)
Supplement: Figure S2 — Mean expression levels of target genes with multiple primary inputs (posterior probability >0.7), for functional binding site motif variants (BSMVs) that were identified in both a dataset with multiple primary inputs, and limited to genes with a single primary input. Expression data is from 211 Affymetrix S98 arrays and a variety of experimental conditions. Even if more than two BSMVs exist at a position, only two are shown in each individual graph, and additional graphs show the pairwise comparison between each BSMV present at each position. The means are ordered across conditions according to the difference between mean expression of the two BSMVs. Vertical lines extending from each point indicate the standard deviation of the mean. Horizontal black bars indicate the difference between the mean ranks. The significance of the functional BSMV was determined without reference to the segregation of experimental conditions, which are shown according to color along the x axis. The number of targets for each BSMV graphed is shown at the bottom right hand of the graph. (PDF) [file pone.0032274.s002.pdf]

Figure S2. Mean expression levels of target genes with multiple primary inputs (posterior probability >0.7), for functional variants that were identified in both a dataset with multiple primary inputs, and limited to genes with a single primary input. Expression data is from 211 Affymetrix S98 arrays and a variety of experimental conditions. Even if more than two variants exist at a position, only two are shown in each individual graph, and additional graphs show the pairwise comparison between each variant present at each position. The means are ordered across conditions according to the difference between mean expression of the two variants. Vertical lines extending from each point indicate the standard deviation of the mean. Horizontal black bars indicate the difference between the mean ranks. The significance of the functional heterogeneity was determined without reference to the segregation of experimental conditions, which are shown according to color along the xaxis. The number of targets for each variant graphed are shown at the bottom right hand of the graph.

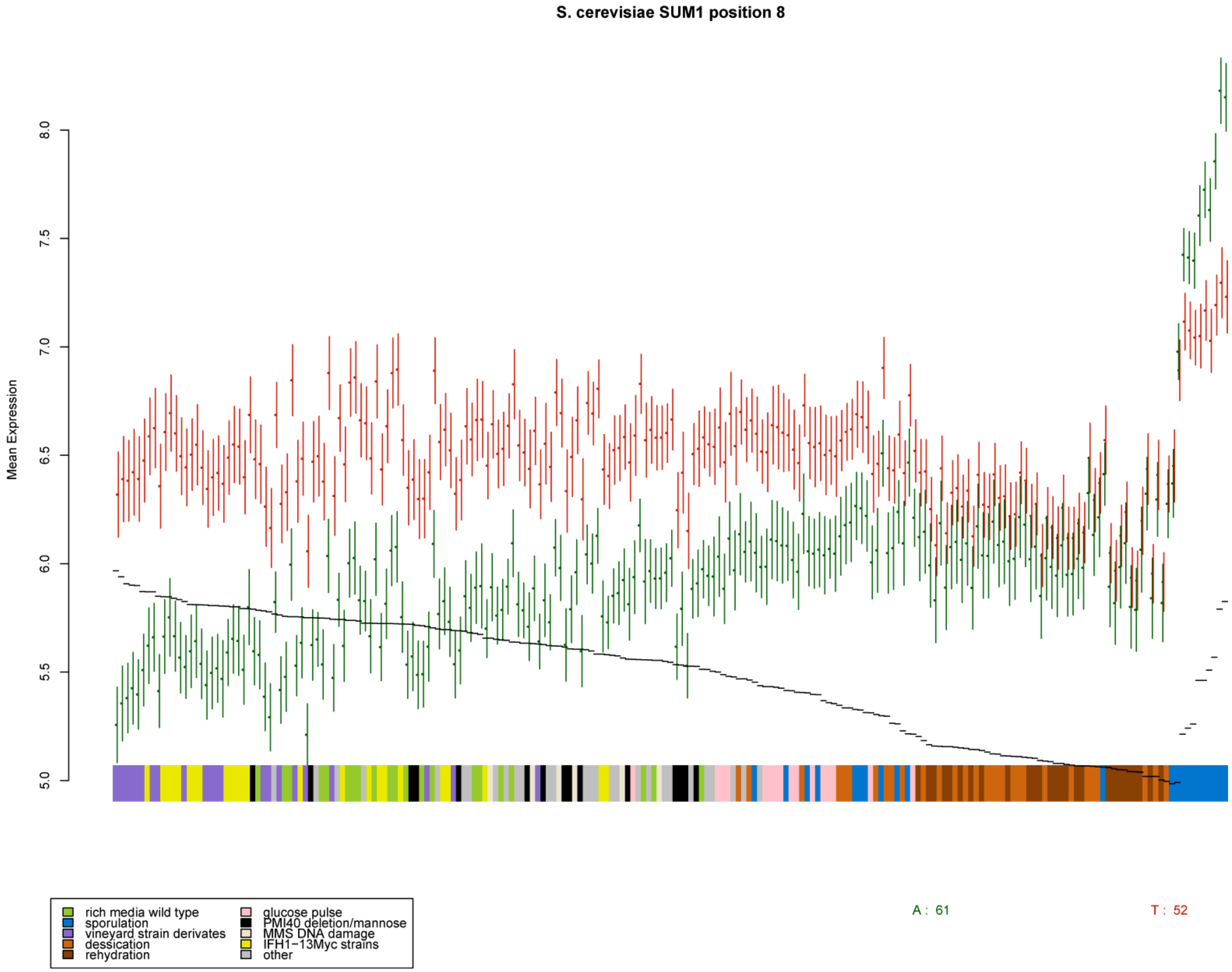

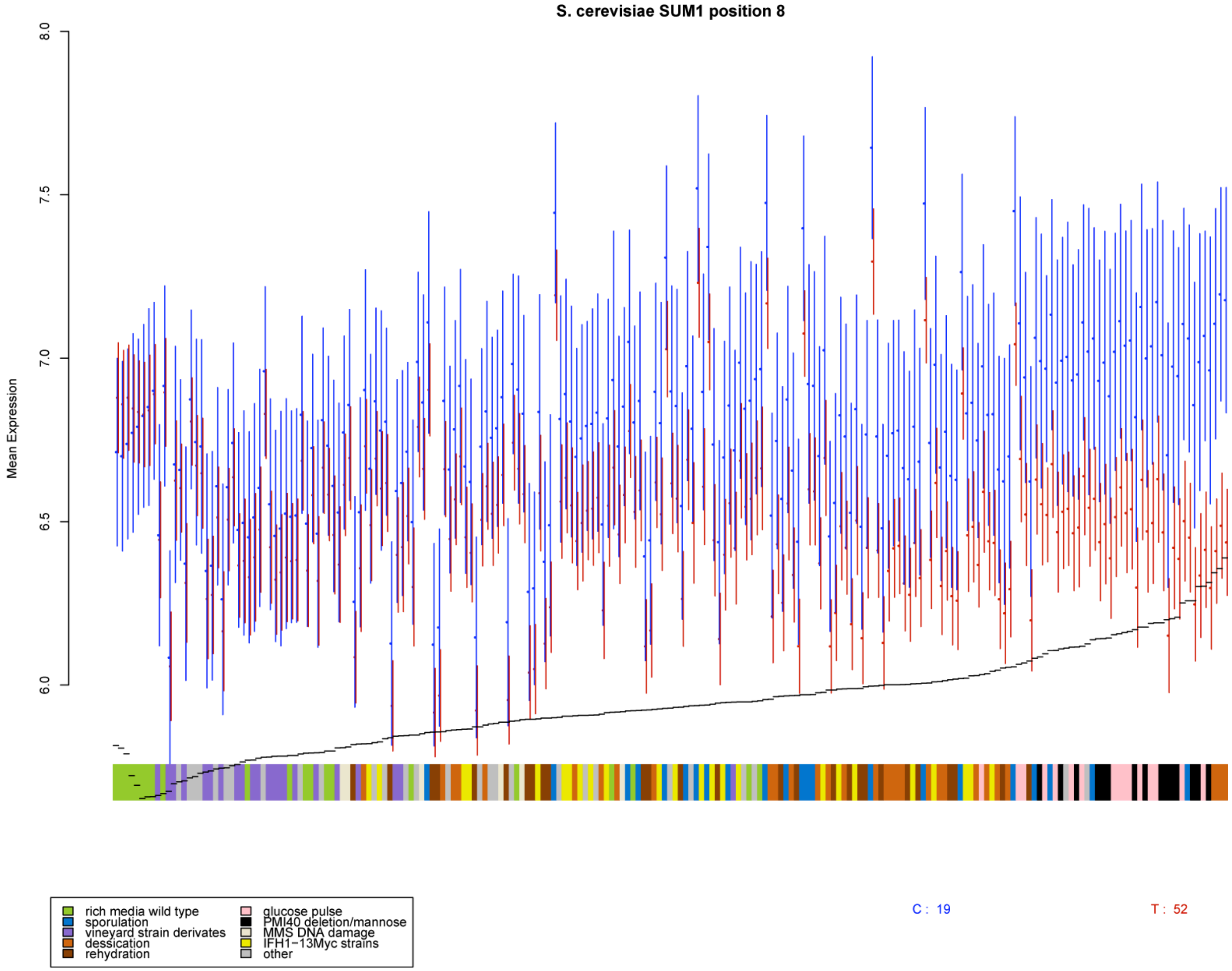

S. cerevisiae SUM1 position 8
Mean Expression
8.0
7.5
7.0
6.5
6.0
rich media wild type
sporulation
vineyard strain derivates
dessication
rehydration
glucose pulse
PMI40 deletion/mannose
MMS DNA damage
IFH1−13Myc strains
other
C : 19
T : 52

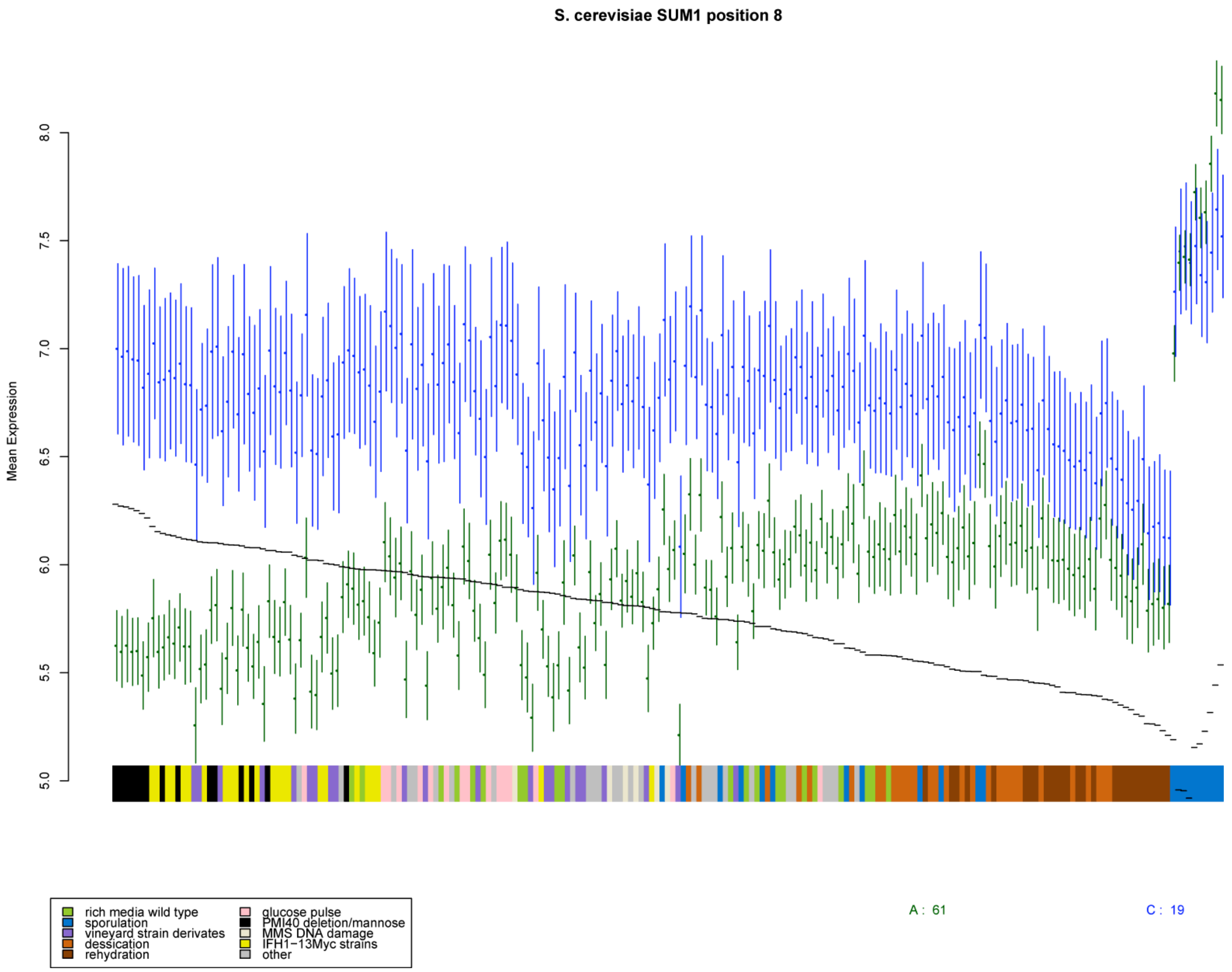
S. cerevisiae SUM1 position 8
Mean Expression
8.0
7.5
7.0
6.5
6.0
5.5
5.0
rich media wild type
sporulation
vineyard strain derivates
dessication
rehydration
glucose pulse
PMI40 deletion/mannose
MMS DNA damage
IFH1−13Myc strains
other
A : 61
C : 19

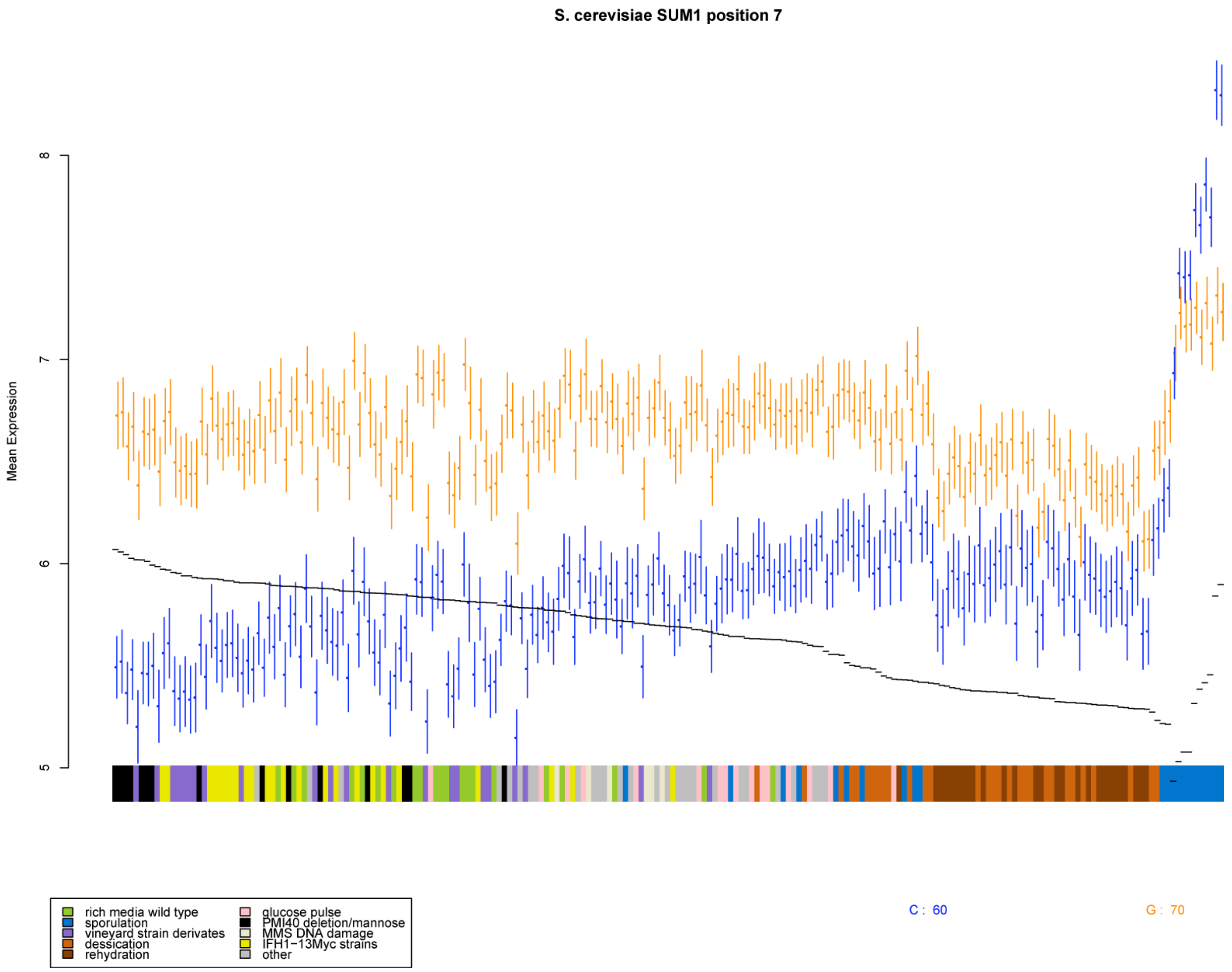

S. cerevisiae SUM1 position 7
Mean Expression
8
7
6
5
rich media wild type
sporulation
vineyard strain derivates
dessication
rehydration
glucose pulse
PMI40 deletion/mannose
MMS DNA damage
IFH1−13Myc strains
other
C : 60
G : 70

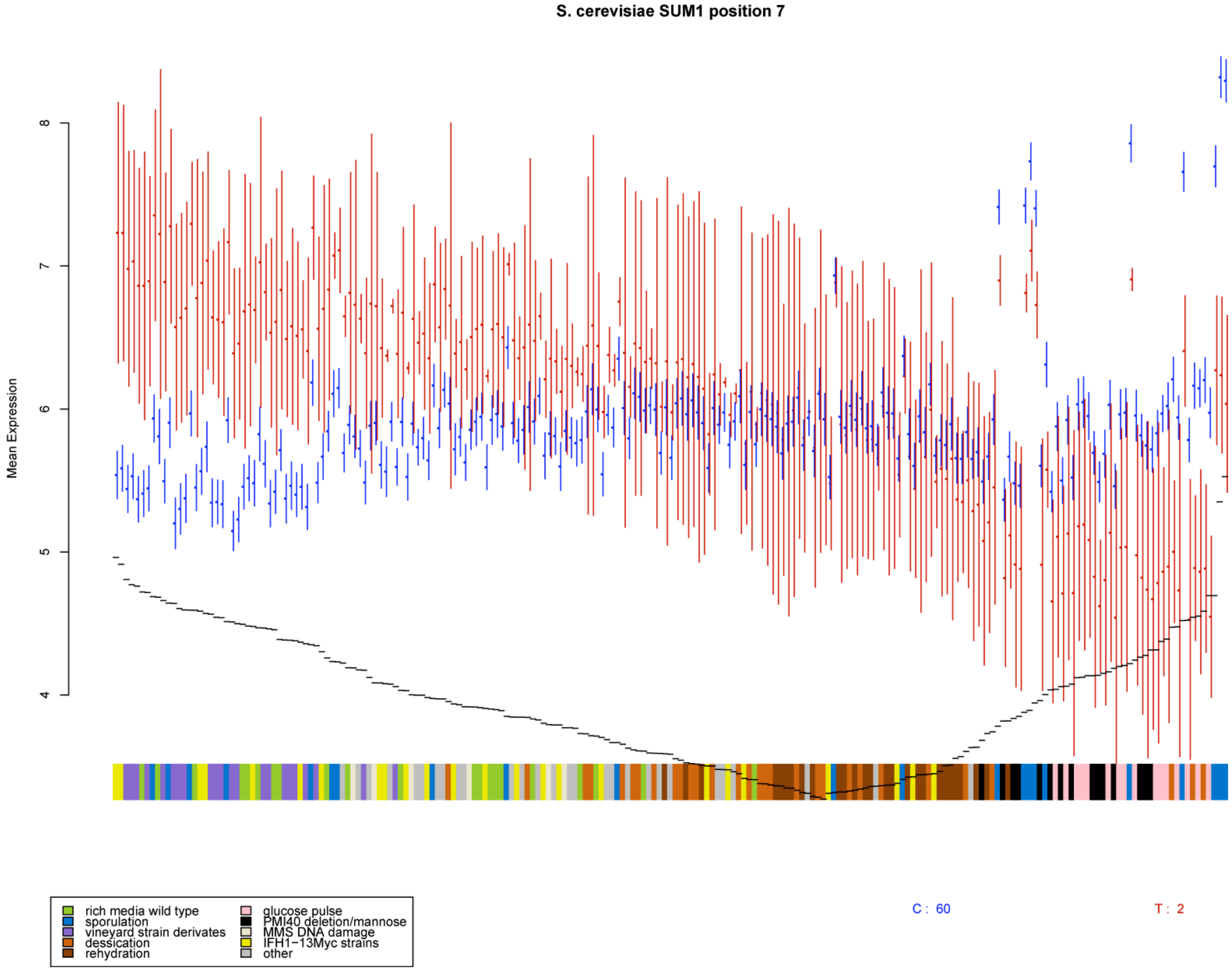

S. cerevisiae SUM1 position 7
Mean Expression
8
7
6
5
4
rich media wild type
sporulation
vineyard strain derivates
dessication
rehydration
glucose pulse
PMI40 deletion/mannose
MMS DNA damage
IFH1−13Myc strains
other
C : 60
T : 2

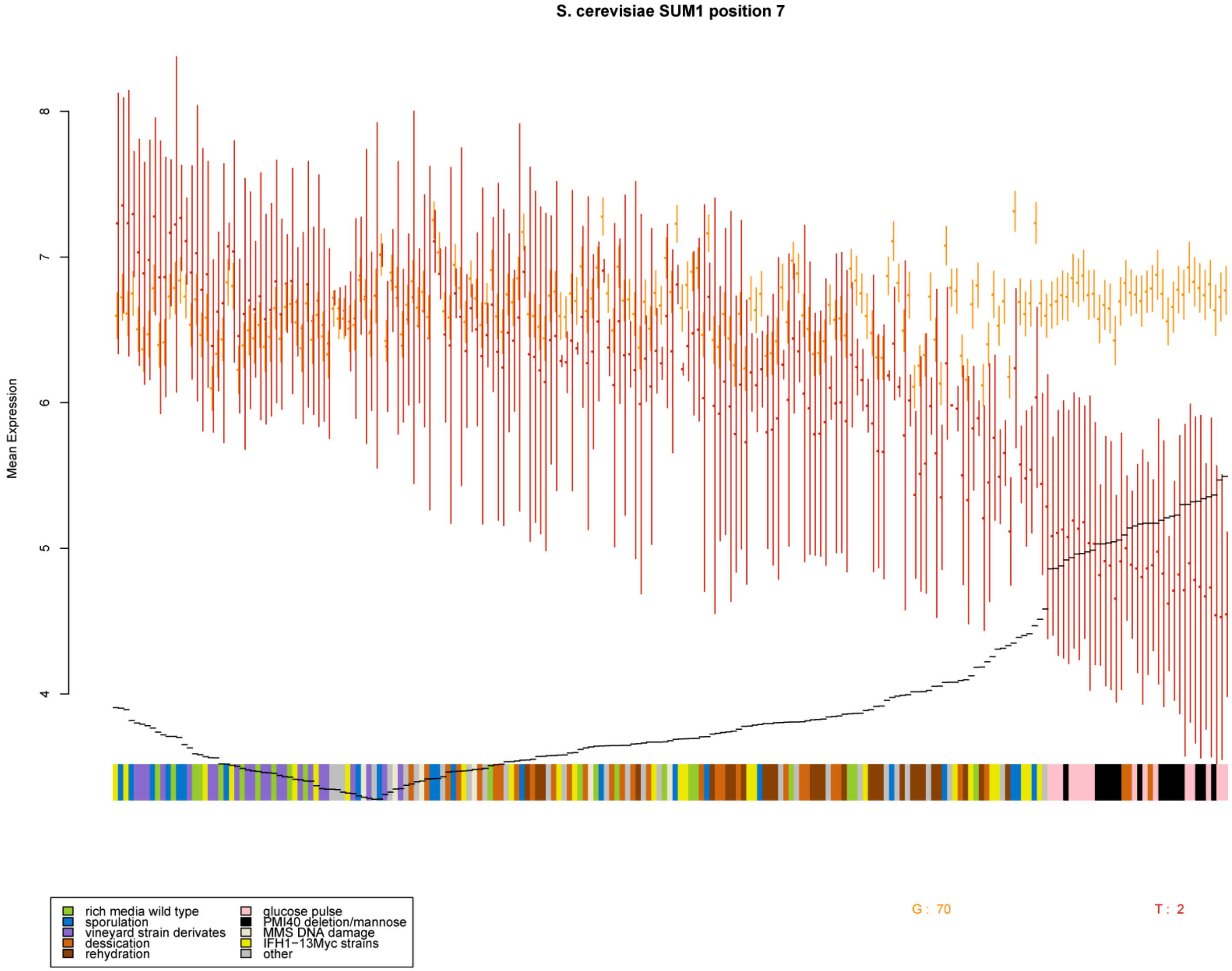

S. cerevisiae SUM1 position 7
Mean Expression
8
7
6
5
4
rich media wild type
sporulation
vineyard strain derivates
dessication
rehydration
glucose pulse
PMI40 deletion/mannose
MMS DNA damage
IFH1−13Myc strains
other
G : 70
T : 2

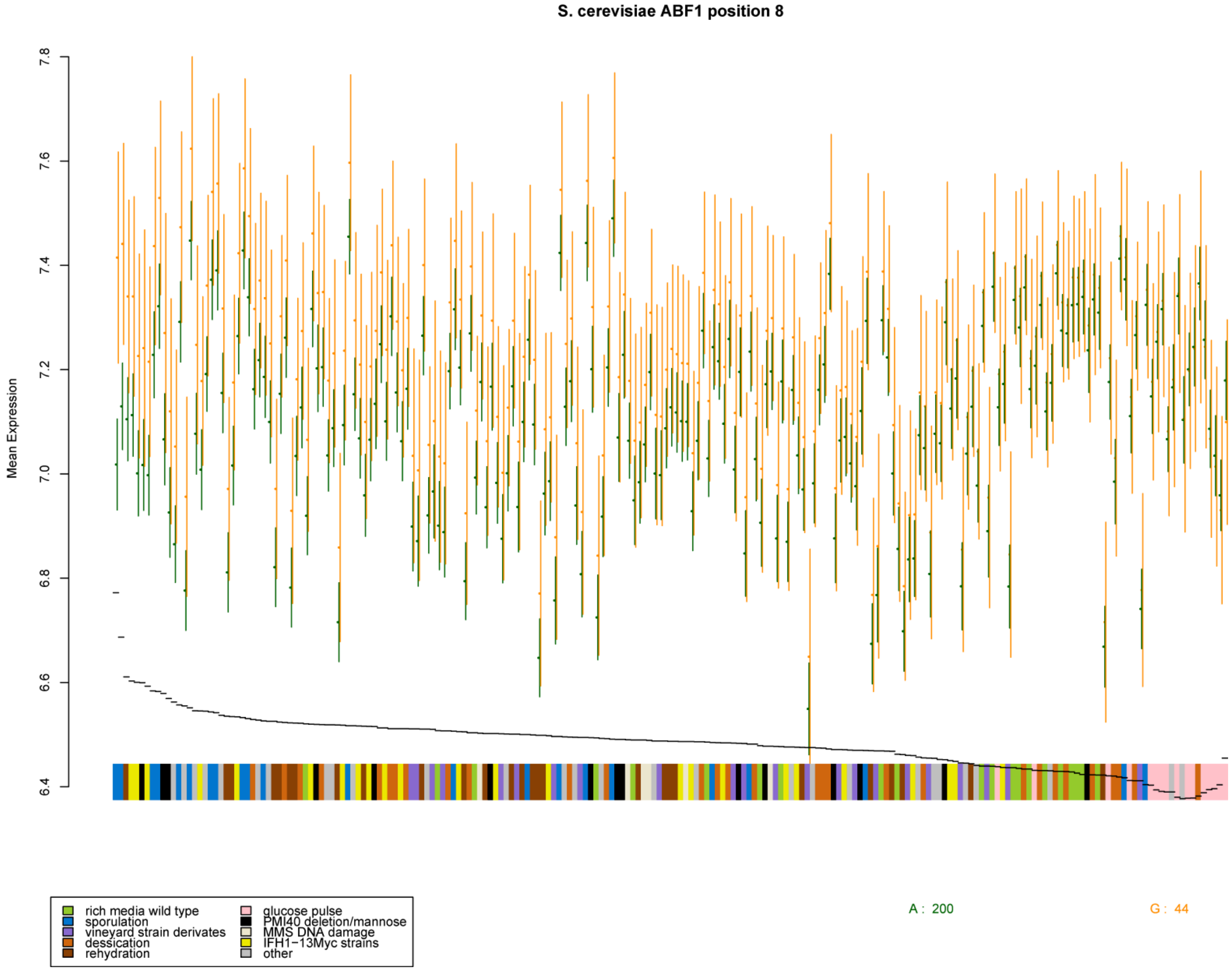

S. cerevisiae ABF1 position 8
Mean Expression
7.8
7.6
7.4
7.2
7.0
6.8
6.6
6.4
rich media wild type
sporulation
vineyard strain derivates
dessication
rehydration
glucose pulse
PMI40 deletion/mannose
MMS DNA damage
IFH1−13Myc strains
other
A : 200
G : 44

S. cerevisiae ABF1 position 8

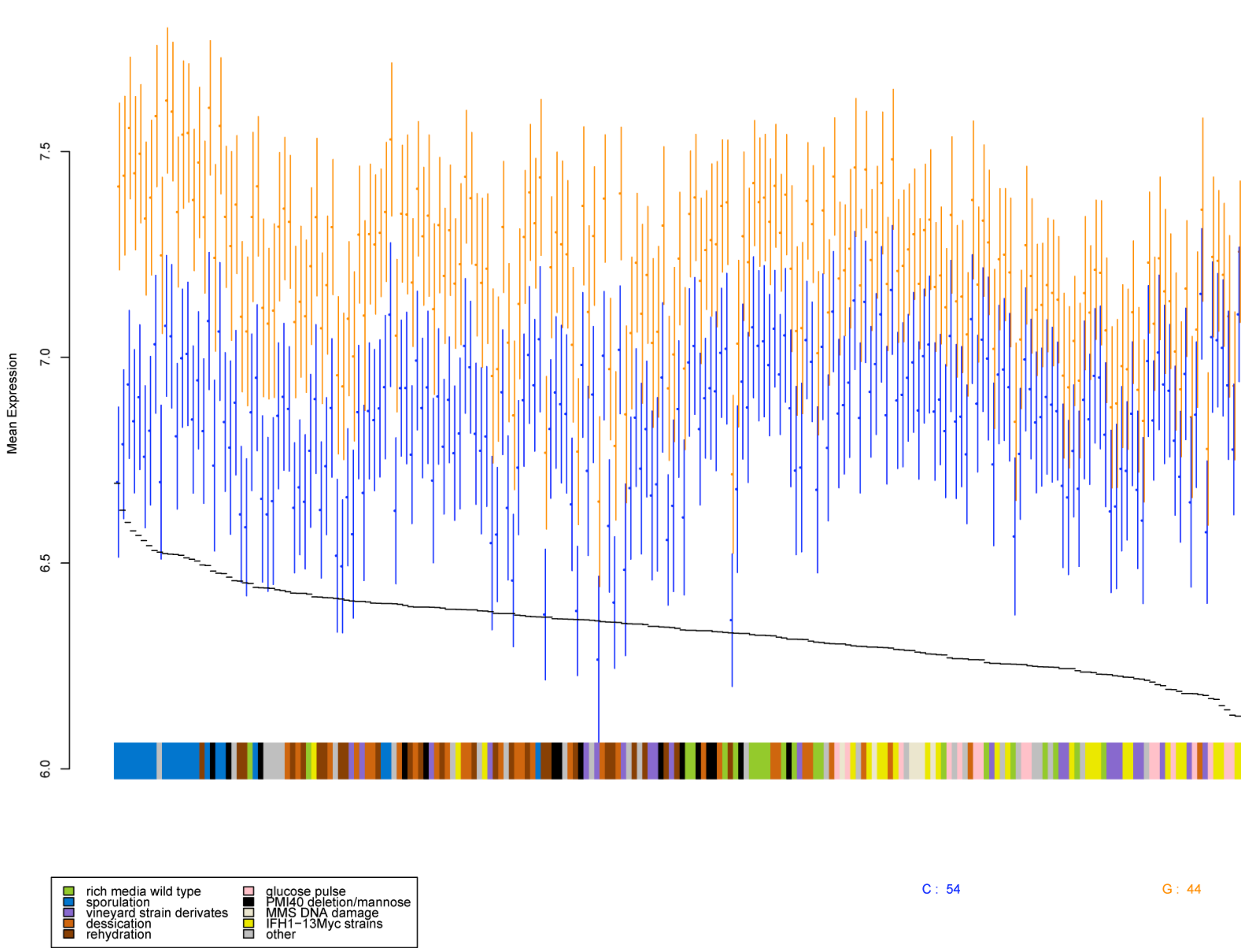

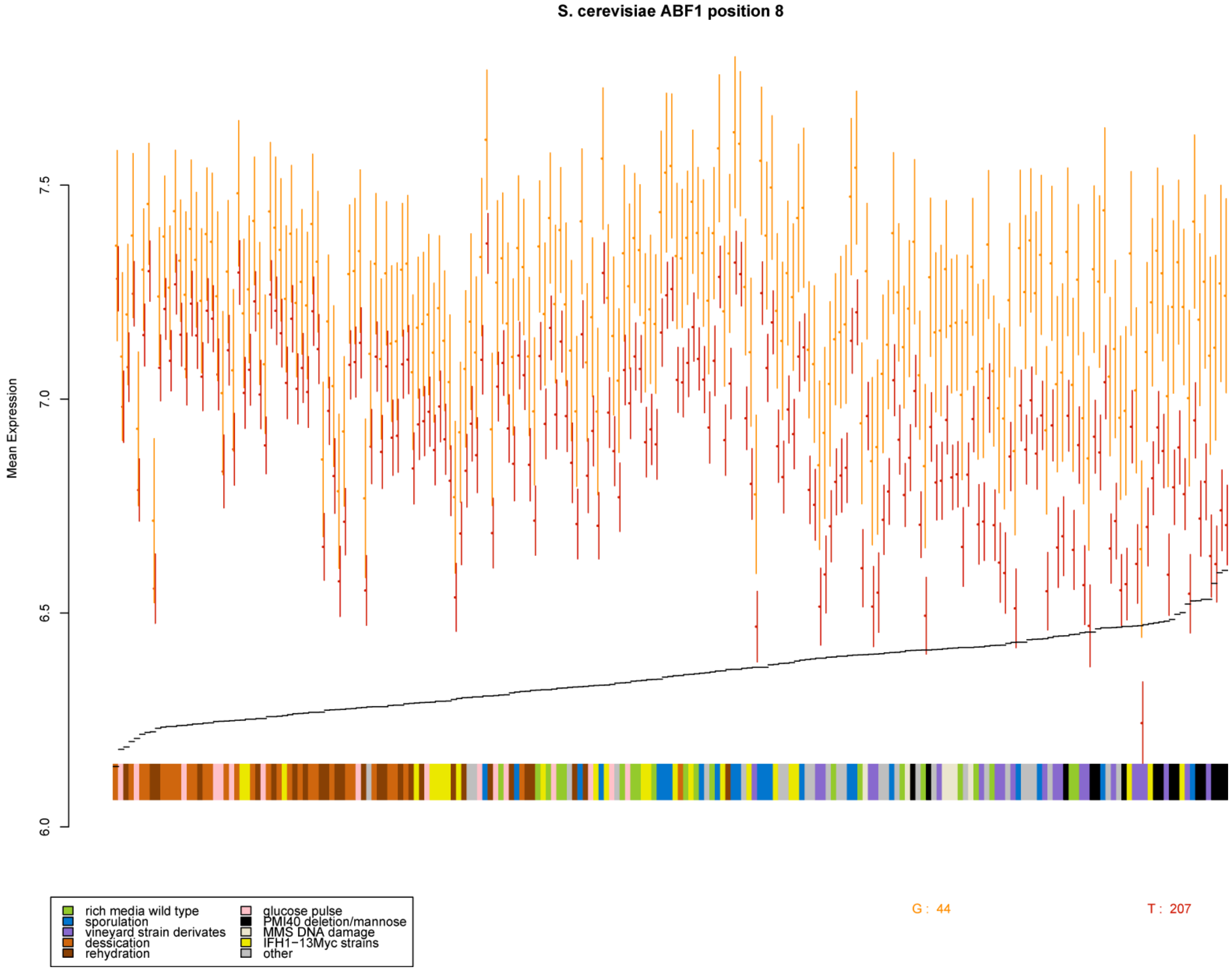
S. cerevisiae ABF1 position 8
Mean Expression
7.5
7.0
6.5
6.0
rich media wild type
sporulation
vineyard strain derivates
dessication
rehydration
glucose pulse
PMI40 deletion/mannose
MMS DNA damage
IFH1−13Myc strains
other
G : 44
T : 207

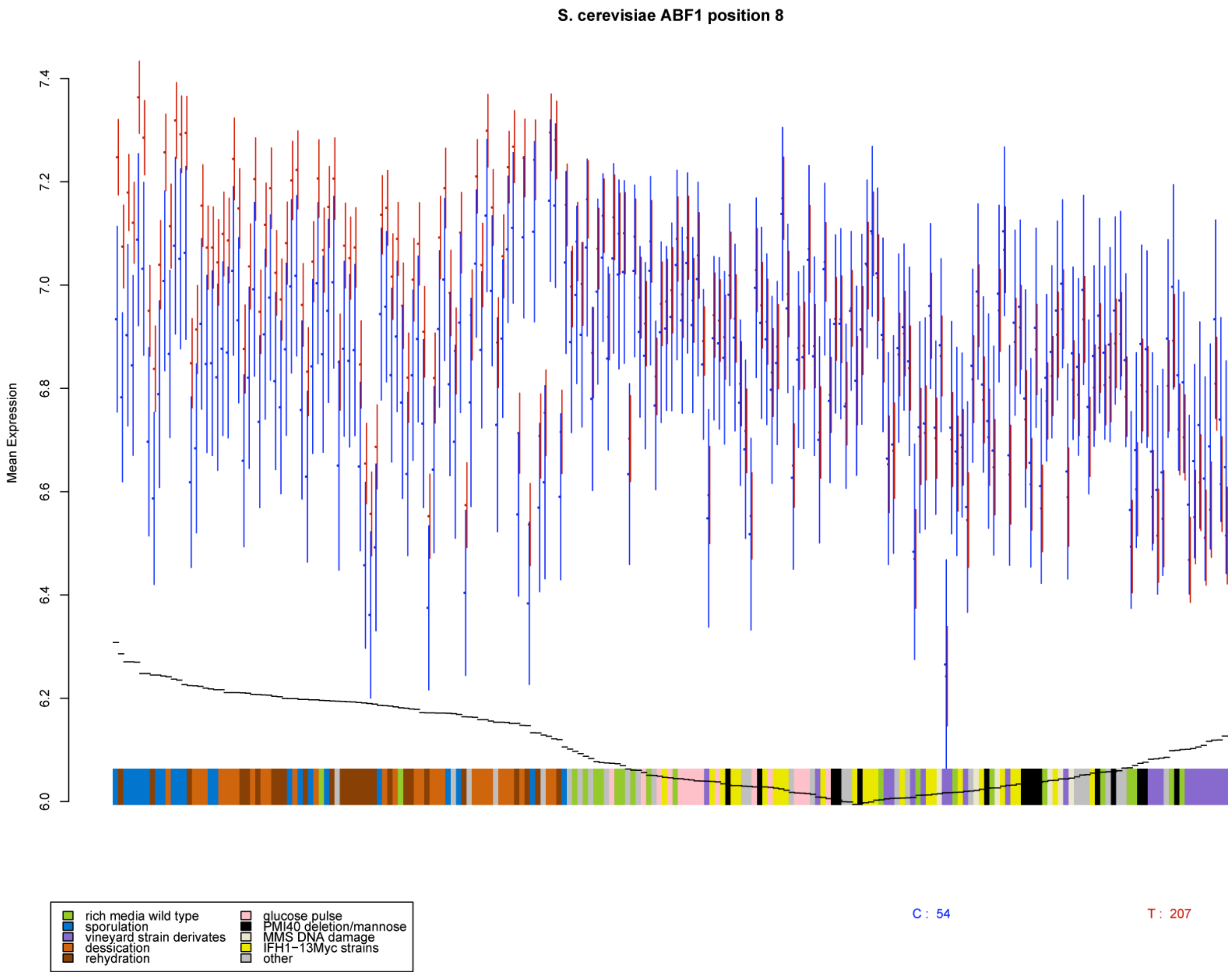
S. cerevisiae ABF1 position 8
Mean Expression
7.4
7.2
7.0
6.8
6.6
6.4
6.2
6.0
rich media wild type
sporulation
vineyard strain derivates
dessication
rehydration
glucose pulse
PMI40 deletion/mannose
MMS DNA damage
IFH1−13Myc strains
other
C : 54
T : 207

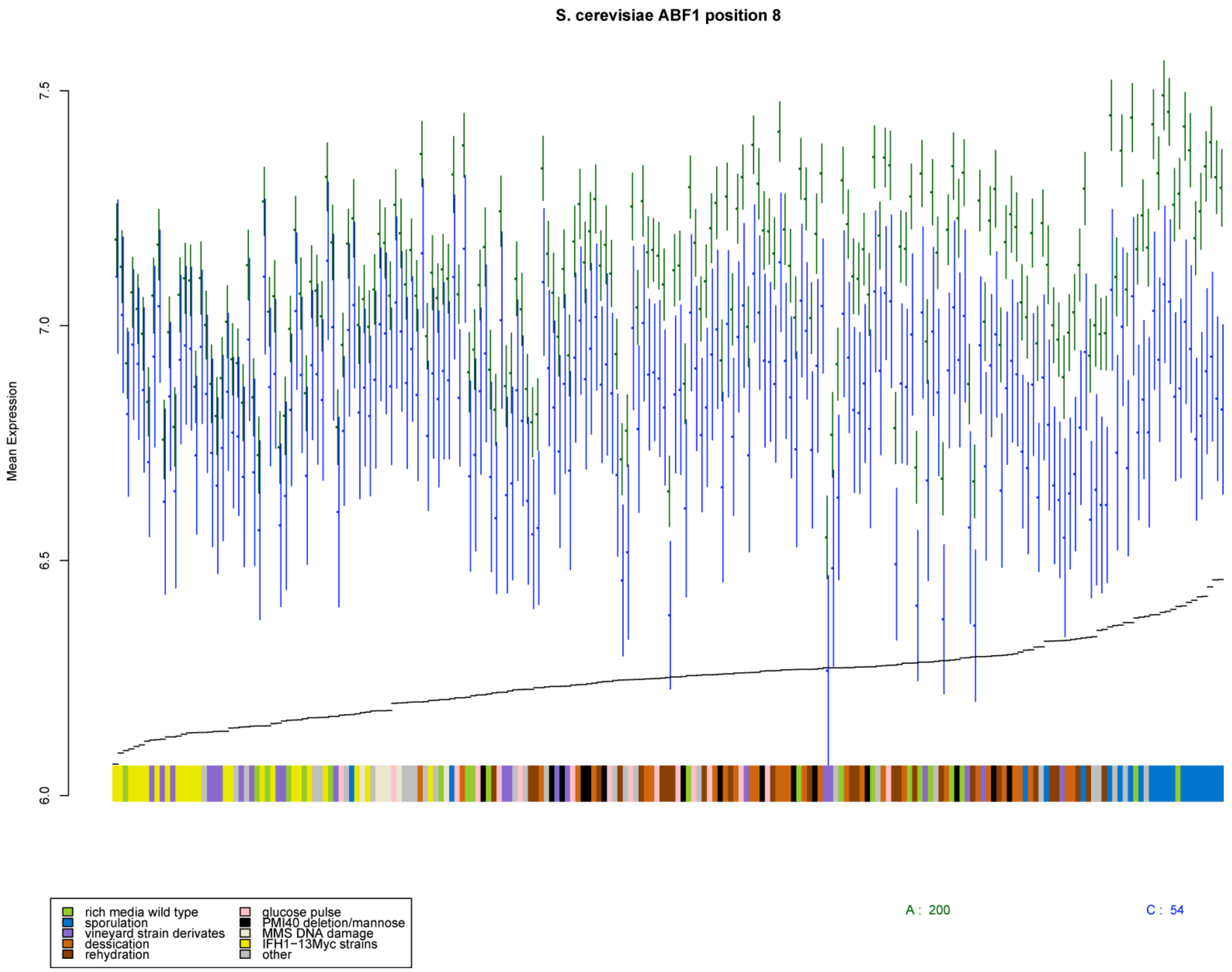
S. cerevisiae ABF1 position 8
Mean Expression
7.5
7.0
6.5
6.0
rich media wild type
sporulation
vineyard strain derivates
dessication
rehydration
glucose pulse
PMI40 deletion/mannose
MMS DNA damage
IFH1−13Myc strains
other
A : 200
C : 54
