# Supplementary material for: Contribution of Transcription Factor Binding Site Motif Variants to Condition-Specific Gene Expression Patterns in Budding Yeast
Source: PLoS One. 2012 Feb 23;7(2):e32274. doi: 10.1371/journal.pone.0032274 (PMC3285675; doi:10.1371/journal.pone.0032274)

Figure S3. Comparison of expression patterns in *S. cerevisiae* of genes associated with "A" and "T" variants at position 7 of the RAP1 transcription factor binding site based on A) binding site annotations from small-scale experimental mapping or B) the genome-wide annotations used in this study. Of six RAP1 binding sites for single input genes according to SCPD, three identical (same position and sequence) binding sites are found in the genome-wide binding site annotations used in this study. A) Genes with only a single RAP1 binding site and no other TF binding site annotations collected from the experimental mapping literature in SCPD[62] were selected. Average expression values of 3 genes with an "A" nucleotide at position 7 of their associated RAP1 binding site are lower during growth in glycerol than the average expression values of 2 genes with a "T." Expression values of genes with either binding variant are not different during growth in other stress conditions. B) Position 7 of the RAP1 binding site is functionally variant (p=0.024) based on our analysis of genome-wide binding site annotations for RAP1 derived from genome-scanning with models based on ChIP-chip binding assays, conservation and motif overrepresentation [36,50,51,52]. Similar to above, average expression of 27 genes with an "A" variant at position 7 of their associated RAP1 binding site are expressed at a lower level than 3 genes with a "T".

A

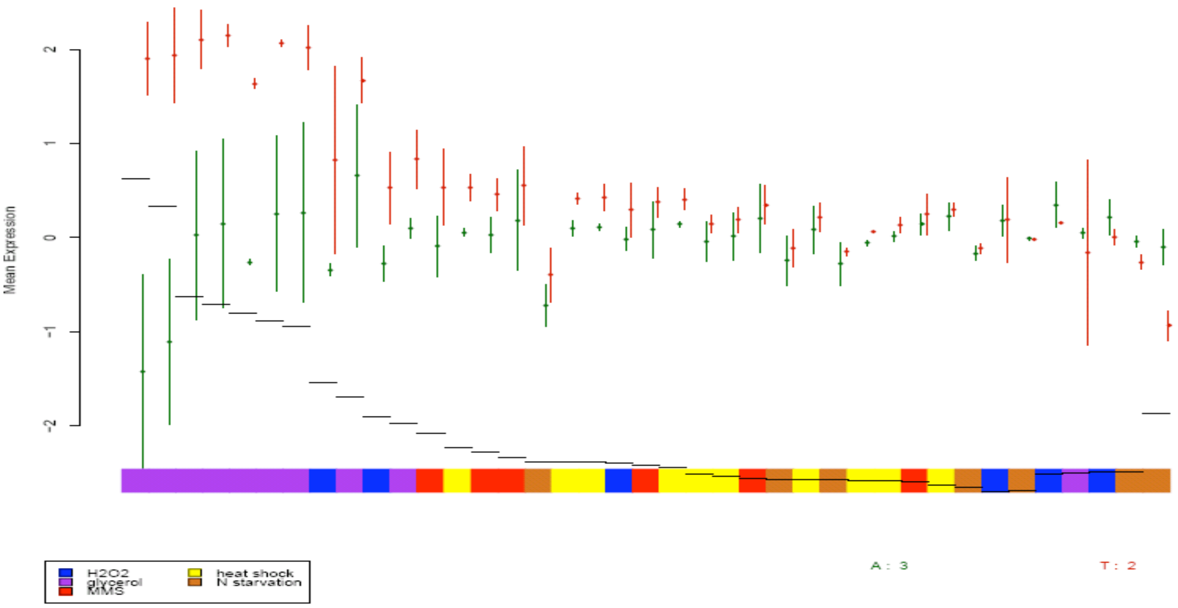

B

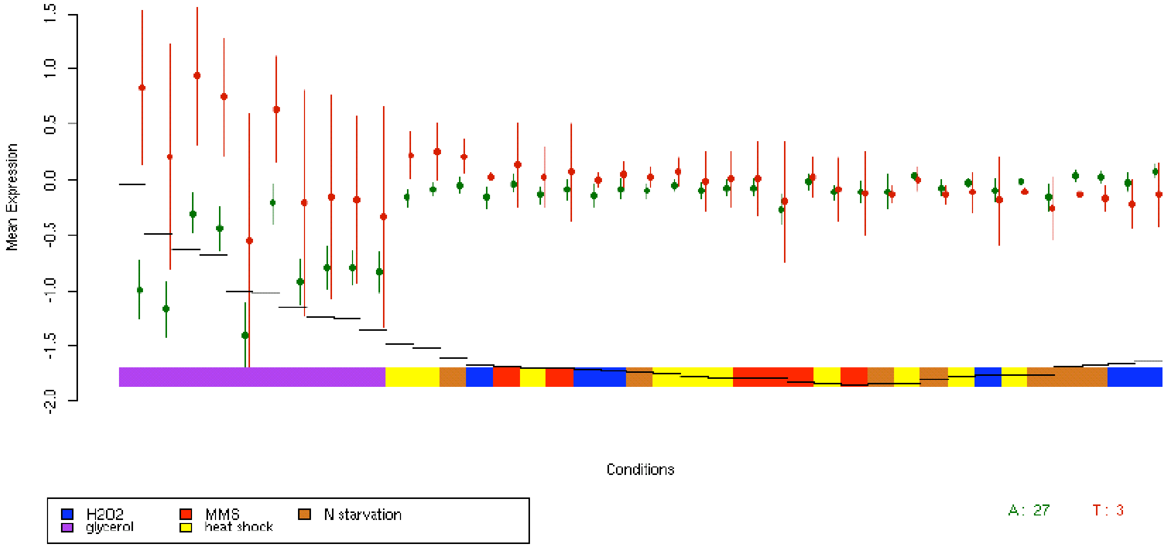

Supplement: Figure S3 — Comparison of expression patterns in S. cerevisiae of genes associated with “A” and “T” binding site motif variants (BSMVs) at position 7 of the RAP1 transcription factor binding site based on binding site annotations from small-scale experimental mapping or the genome-wide annotations used in this study. Of six RAP1 binding sites for single input genes according to SCPD, three identical (same position and sequence) binding sites are found in the genome-wide binding site annotations used in this study. A) Genes with only a single RAP1 binding site and no other TF binding site annotations collected from the experimental mapping literature in SCPD [71] were selected. Average expression values of 3 genes with an “A” nucleotide at position 7 of their associated RAP1 binding site are lower during growth in glycerol than the average expression values of 2 genes with a “T.” Expression values of genes with either BSMV are not different during growth in other stress conditions. B) Position 7 of the RAP1 binding site is functionally variant (p = 0.024) based on our analysis of genome-wide binding site annotations for RAP1 derived from genome-scanning with models based on ChIP-chip binding assays, conservation and motif overrepresentation [40], [54], [55], [56]. Similar to above, average expression of 27 genes with an “A” BSMV at position 7 of their associated RAP1 binding site are expressed at a lower level than 3 genes with a “T” BSMV. (PDF) [file pone.0032274.s003.pdf]
