# Supplementary material for: Contribution of Transcription Factor Binding Site Motif Variants to Condition-Specific Gene Expression Patterns in Budding Yeast
Source: PLoS One. 2012 Feb 23;7(2):e32274. doi: 10.1371/journal.pone.0032274 (PMC3285675; doi:10.1371/journal.pone.0032274)

**Figure S4**. **Number of target genes associated with functional and non-functional motif positions.** Variable motif positions considered in the analysis have, on average, ~35 target genes, and functional variants do not have a significantly different number of target genes than do non-functional variants (p=0.4).

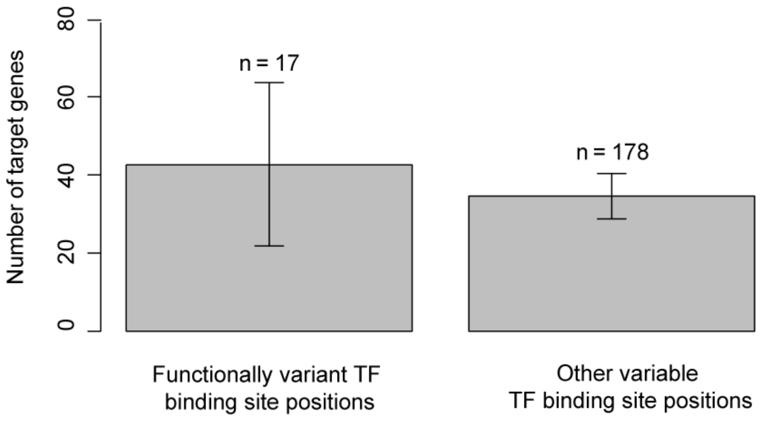

Supplement: Figure S4 — Number of target genes associated with functional and non-functional binding site motif variants (BSMVs). Variable motif positions considered in the analysis have, on average, ∼35 target genes, and functional BSMVs do not have a significantly different number of target genes than do non-functional BSMVs (p = 0.4). (PDF) [file pone.0032274.s004.pdf]
