# Supplementary material for: Contribution of Transcription Factor Binding Site Motif Variants to Condition-Specific Gene Expression Patterns in Budding Yeast
Source: PLoS One. 2012 Feb 23;7(2):e32274. doi: 10.1371/journal.pone.0032274 (PMC3285675; doi:10.1371/journal.pone.0032274)
Supplement: Figure S5 — Complete set of figures showing comparison of average gene expression levels between genes with different functional transcription factor binding site motif variants (BSMVs) in S. cerevisiae, S. kudriavzevii, S. mikatae and S. paradoxus based on expression data from Y6.4kv6 cDNA arrays. Mean expression levels for target genes of functional BSMVs found at positions in TF binding sites using expression data from Y6.4kv6 cDNA arrays and stress conditions. Even if more than two BSMVs exist at a position, only two are shown in each individual graph, and additional graphs show the pairwise comparison between each BSMV present at each position. The means are ordered across conditions according to the difference between mean expression of genes regulated by the two BSMVs. Vertical lines extending from each point indicate the standard deviation of the mean. Horizontal black bars indicate the difference between the mean ranks. The significance of the functional BSMVs was determined without reference to the segregation of experimental conditions, which are shown according to color along the x-axis. The number of targets for each BSMV graphed is shown at the bottom right hand of the graph. (PDF) [file pone.0032274.s005.pdf]

Figure S5. Complete set of figures showing comparison of average gene expression levels between genes with different functional variants of transcription factor binding site motifs in *S. cerevisiae, S. kudriavzevii, S. mikatae* and *S. paradoxus* based on expression data from Y6.4kv6 cDNA arrays. Mean expression levels for target genes of functional variants found at positions in TF binding sites using expression data from Y6.4kv6 cDNA arrays and stress conditions. Even if more than two variants exist at a position, only two are shown in each individual graph, and additional graphs show the pairwise comparison between each variant present at each position. The means are ordered across conditions according to the difference between mean expression of the two variants. Vertical lines extending from each point indicate the standard deviation of the mean. Horizontal black bars indicate the difference between the mean ranks. The significance of the functional heterogeneity was determined without reference to the segregation of experimental conditions, which are shown according to color along the x-axis. The number of targets for each variant graphed are shown at the bottom right hand of the graph.

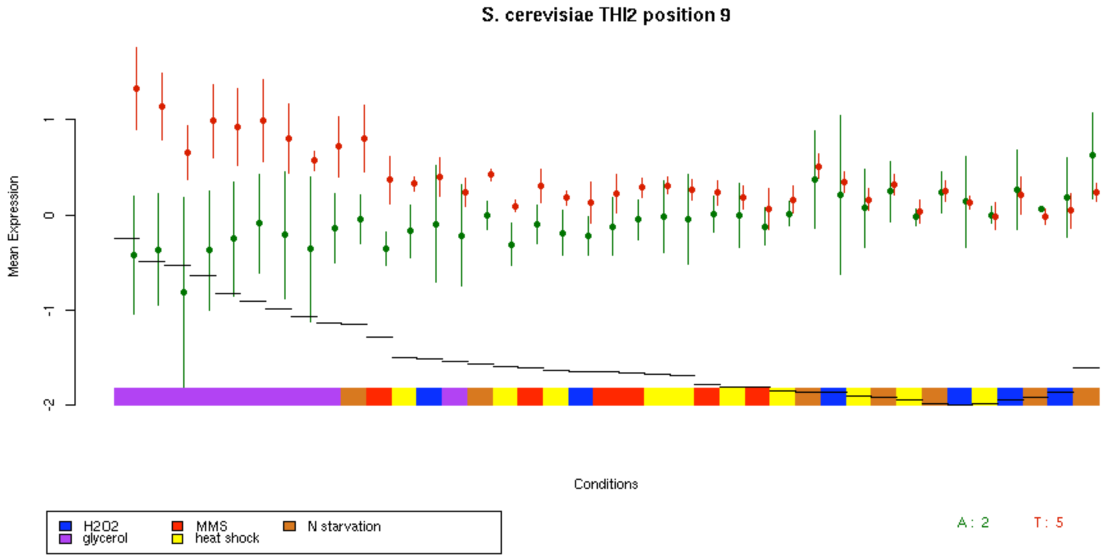

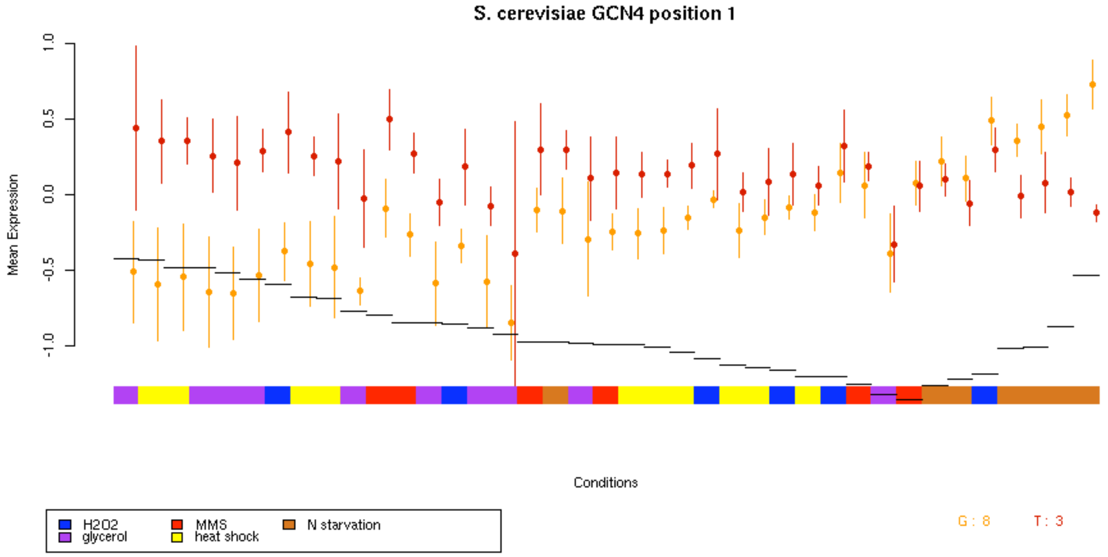

S. cerevisiae HSF1 position 7

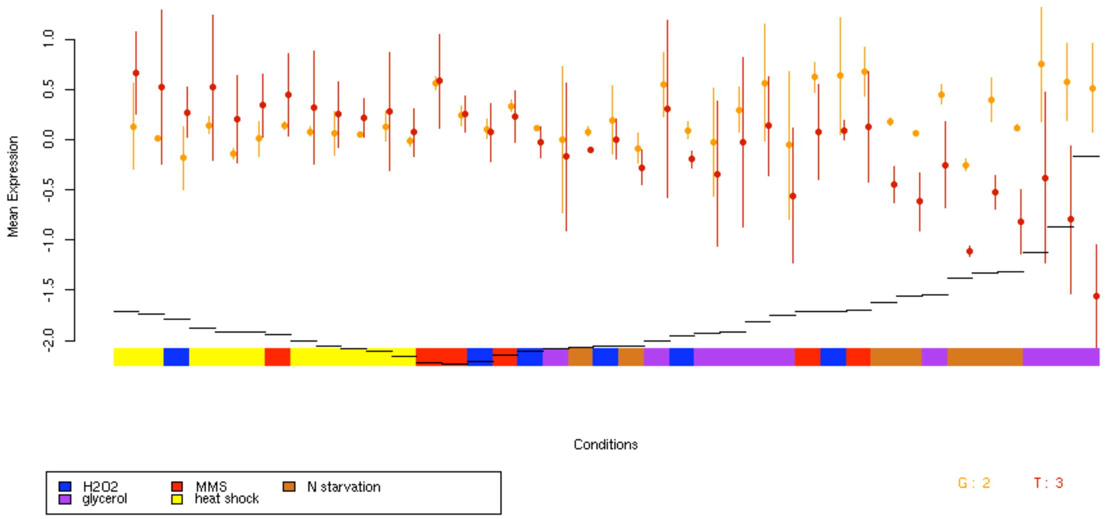

S. cerevisiae MATalpha2 position 9

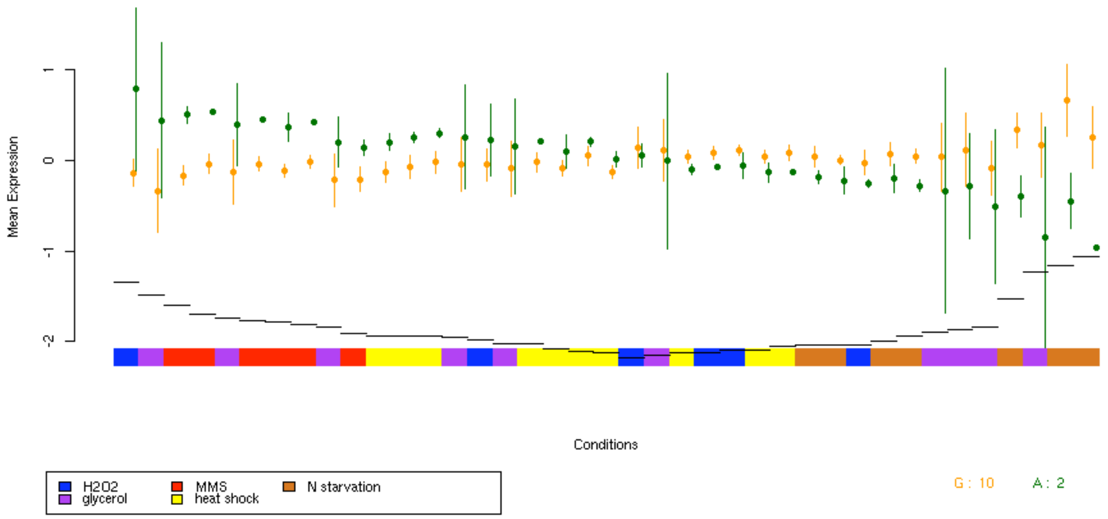

S. cerevisiae NRG1 position 2

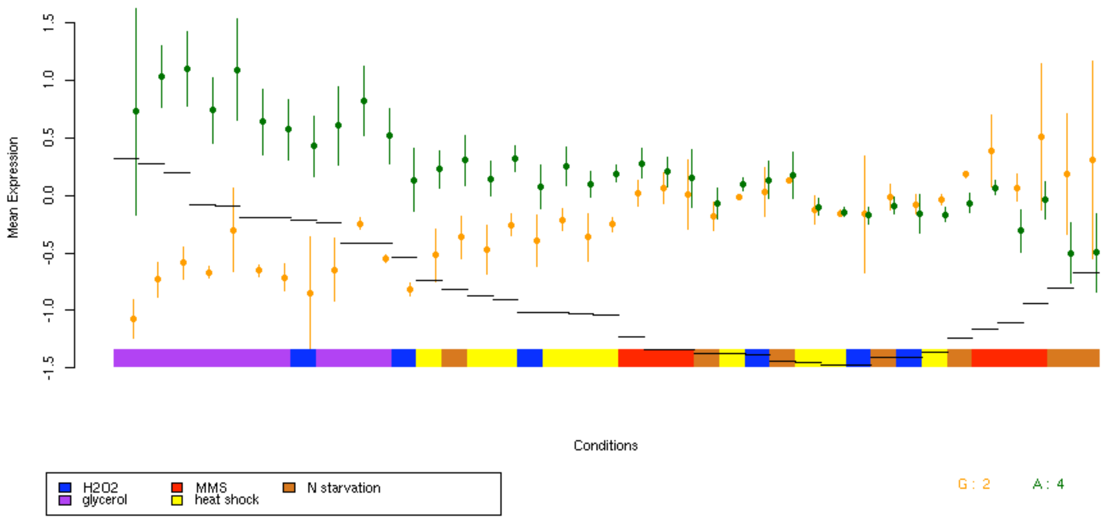

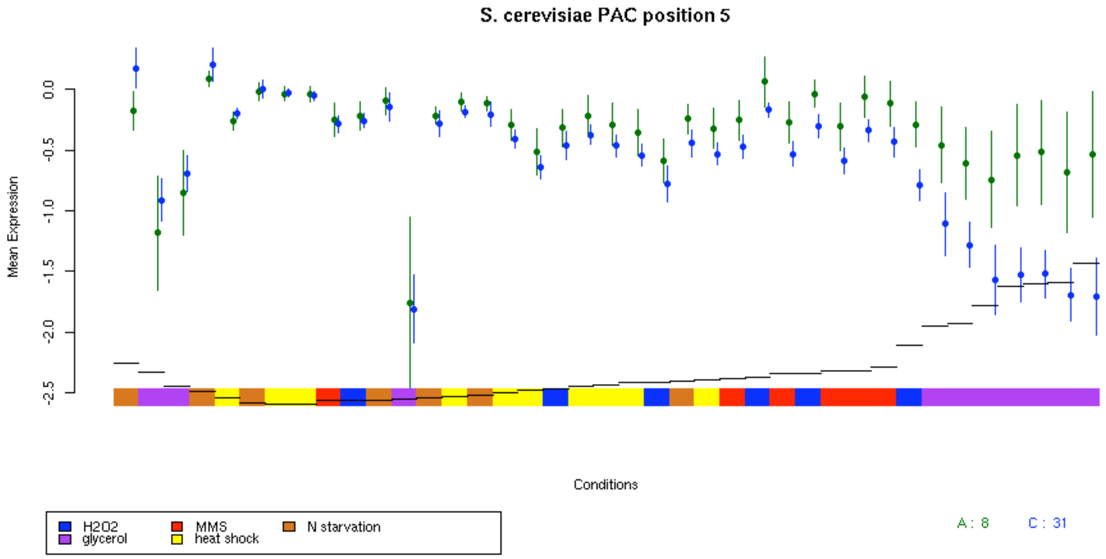
S. cerevisiae PAC position 5
Mean Expression
0.0
-0.5
-1.0
-1.5
-2.0
-2.5
Conditions
H2O2
glycerol
MMS
heat shock
N starvation
A : 8
C : 31

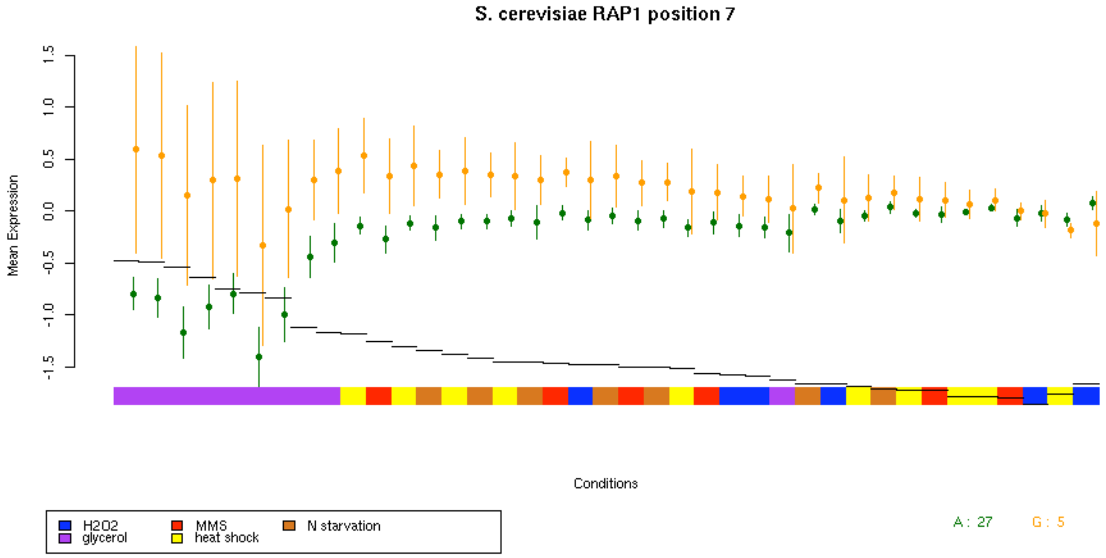
S. cerevisiae RAP1 position 7
Mean Expression
1.5
1.0
0.5
0.0
-0.5
-1.0
-1.5
Conditions
H2O2
glycerol
MMS
heat shock
N starvation
A : 27
G : 5

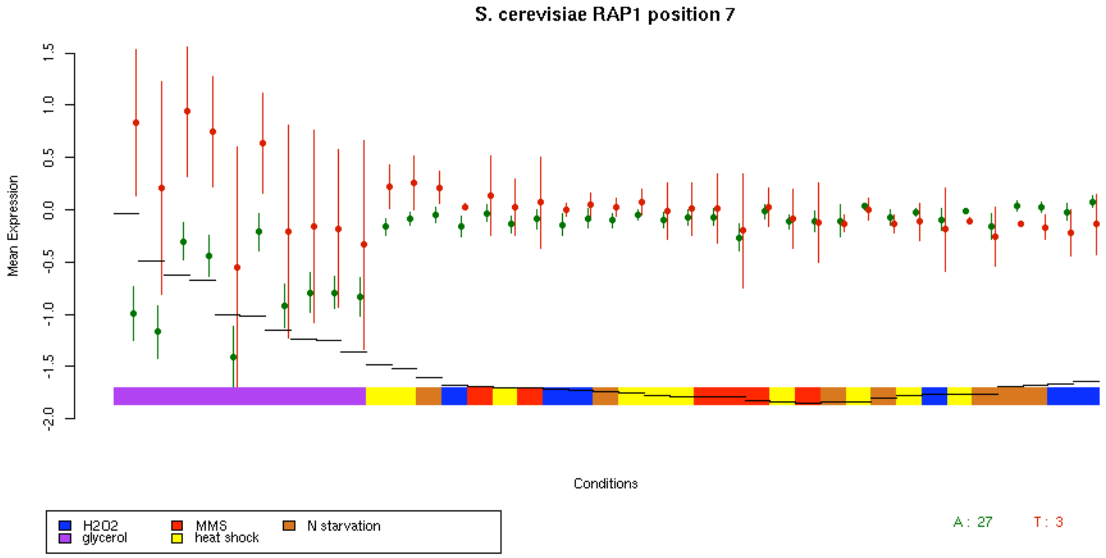
S. cerevisiae RAP1 position 7
Mean Expression
1.5
1.0
0.5
0.0
-0.5
-1.0
-1.5
-2.0
Conditions
H2O2
glycerol
MMS
heat shock
N starvation
A : 27
T : 3

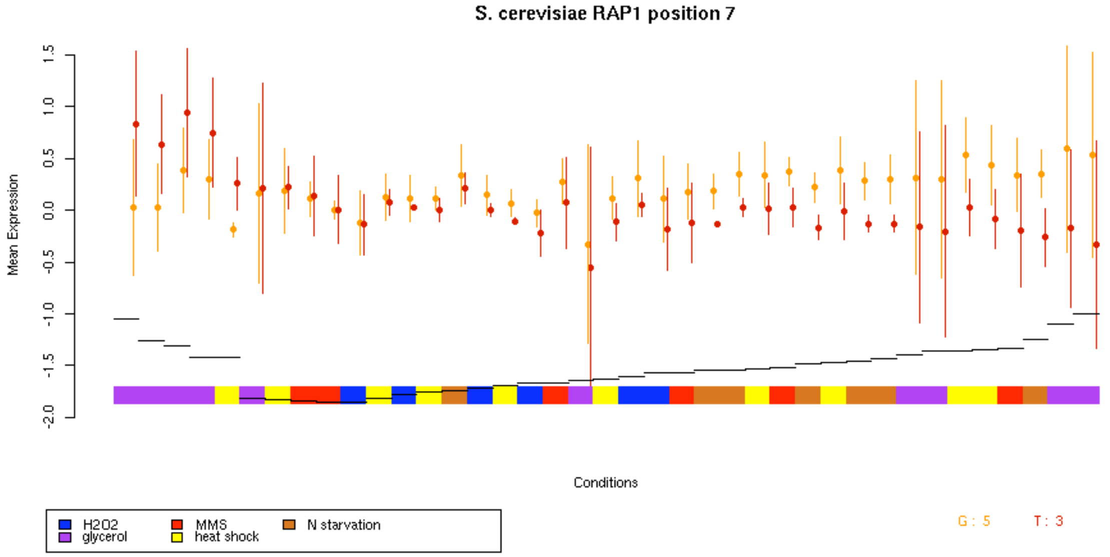
S. cerevisiae RAP1 position 7
Mean Expression
-2.0
-1.5
-1.0
-0.5
0.0
0.5
1.0
1.5
Conditions
H2O2
glycerol
MMS
heat shock
N starvation
G : 5
T : 3

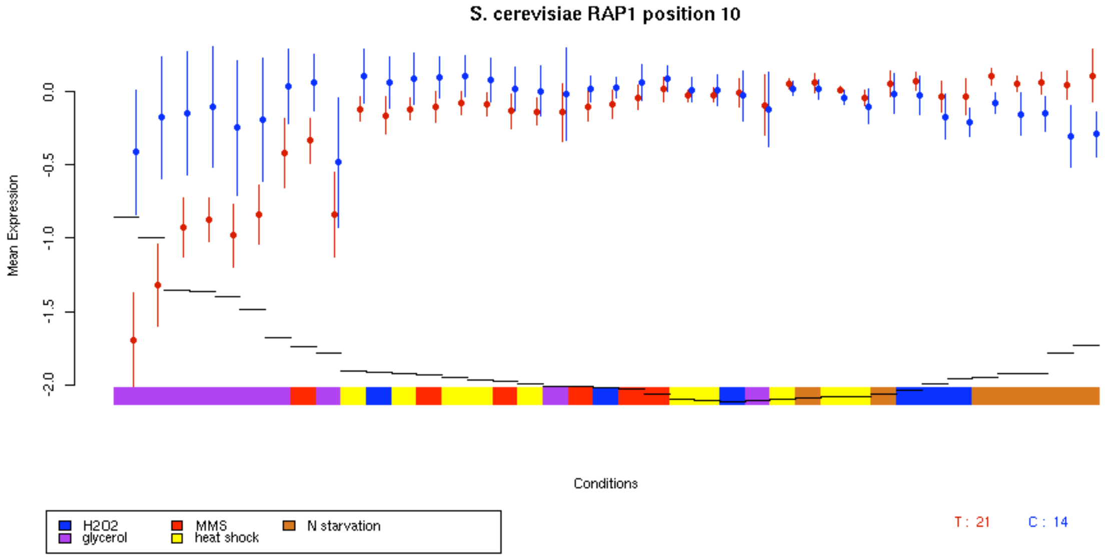
S. cerevisiae RAP1 position 10
Mean Expression
-2.0
-1.5
-1.0
-0.5
0.0
Conditions
H2O2
glycerol
MMS
heat shock
N starvation
T : 21
C : 14

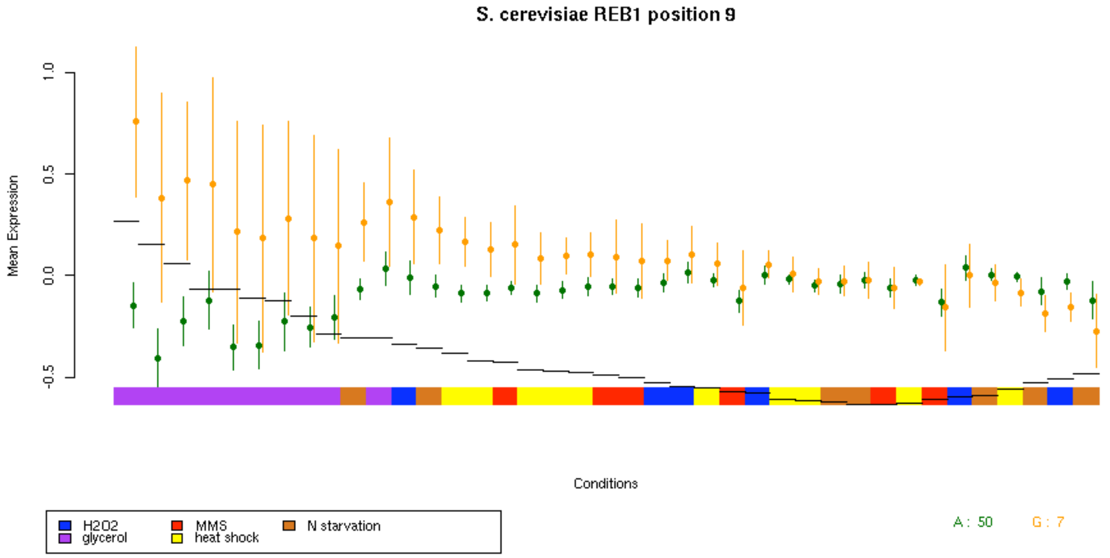
S. cerevisiae REB1 position 9
Mean Expression
-0.5
0.0
0.5
1.0
Conditions
H2O2
glycerol
MMS
heat shock
N starvation
A : 50
G : 7

S. cerevisiae RPN4 position 10

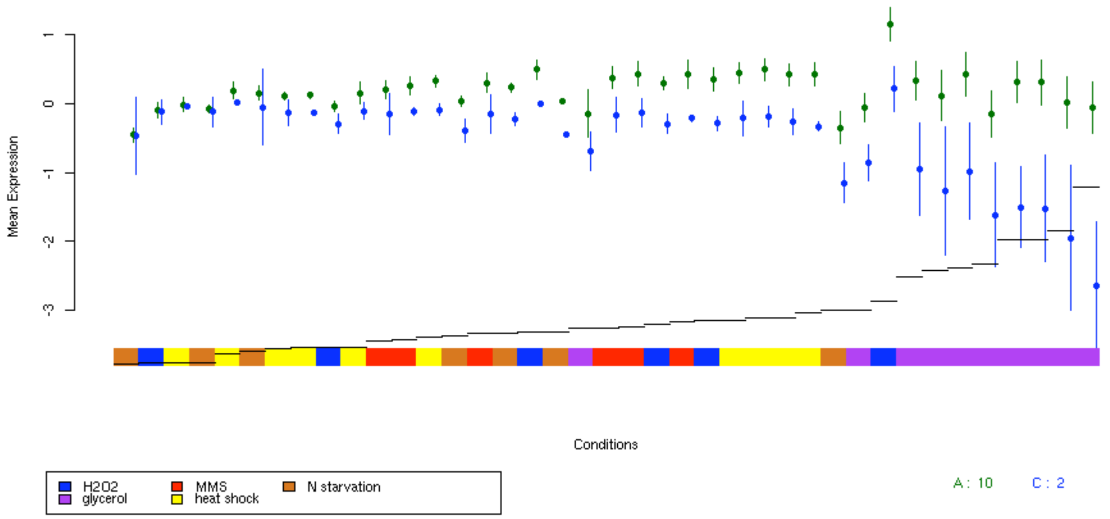

S. cerevisiae RPN4 position 10

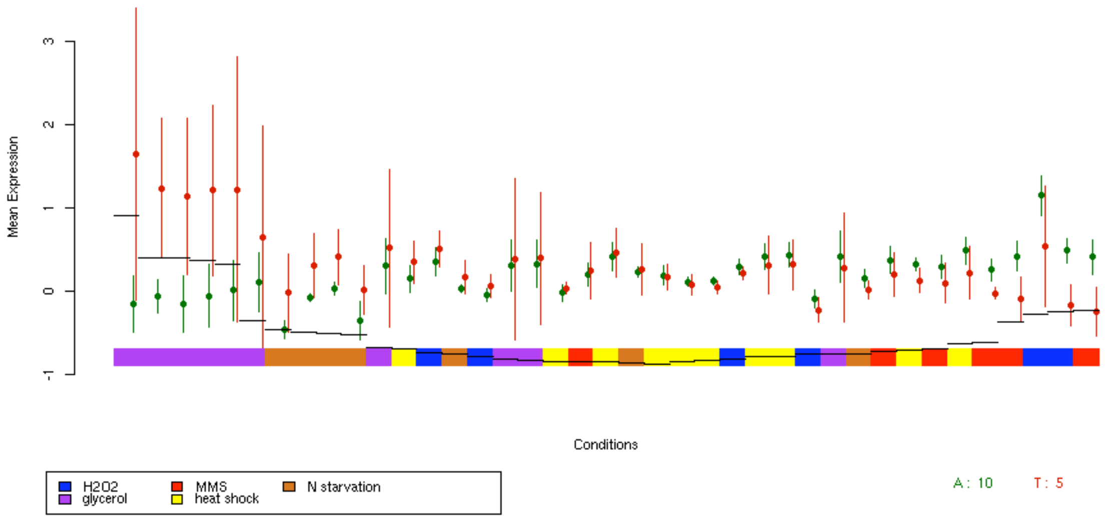

S. cerevisiae RPN4 position 10

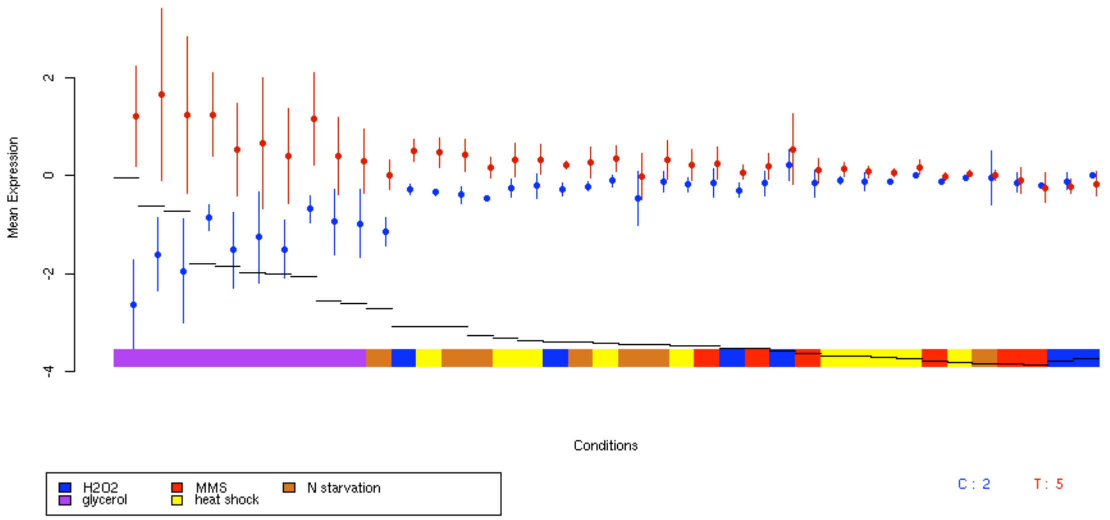

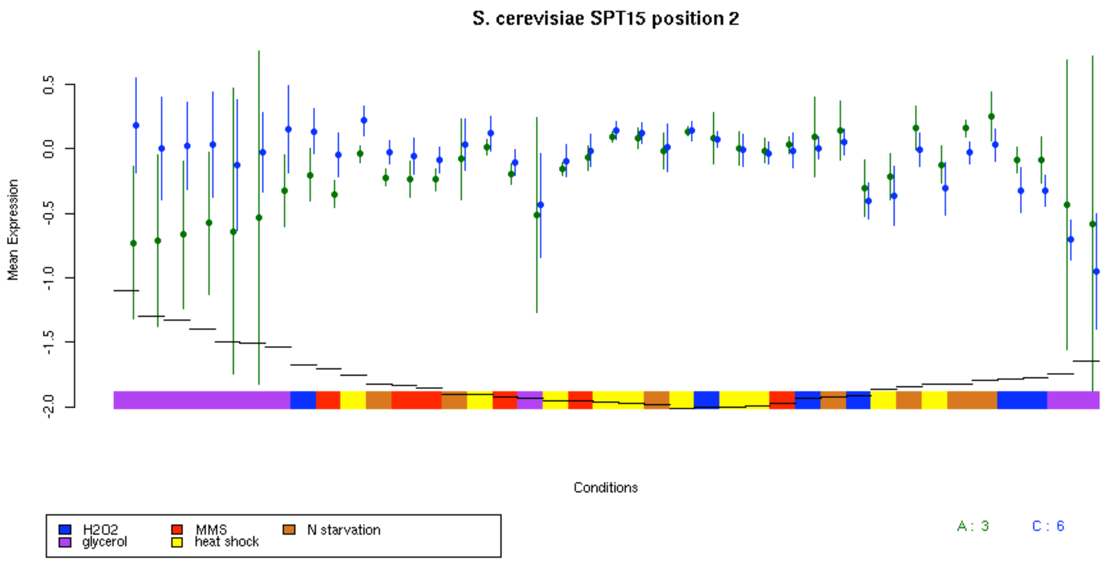
S. cerevisiae SPT15 position 2
Mean Expression
0.5
0.0
-0.5
-1.0
-1.5
-2.0
Conditions
H2O2
glycerol
MMS
heat shock
N starvation
A : 3
C : 6

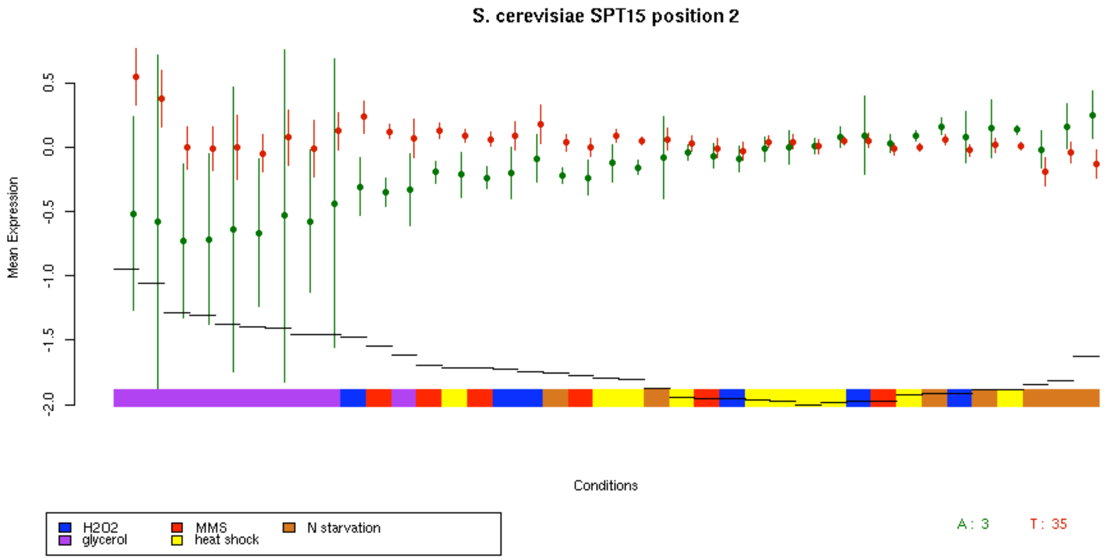
S. cerevisiae SPT15 position 2
Mean Expression
0.5
0.0
-0.5
-1.0
-1.5
-2.0
Conditions
H2O2
glycerol
MMS
heat shock
N starvation
A : 3
T : 35

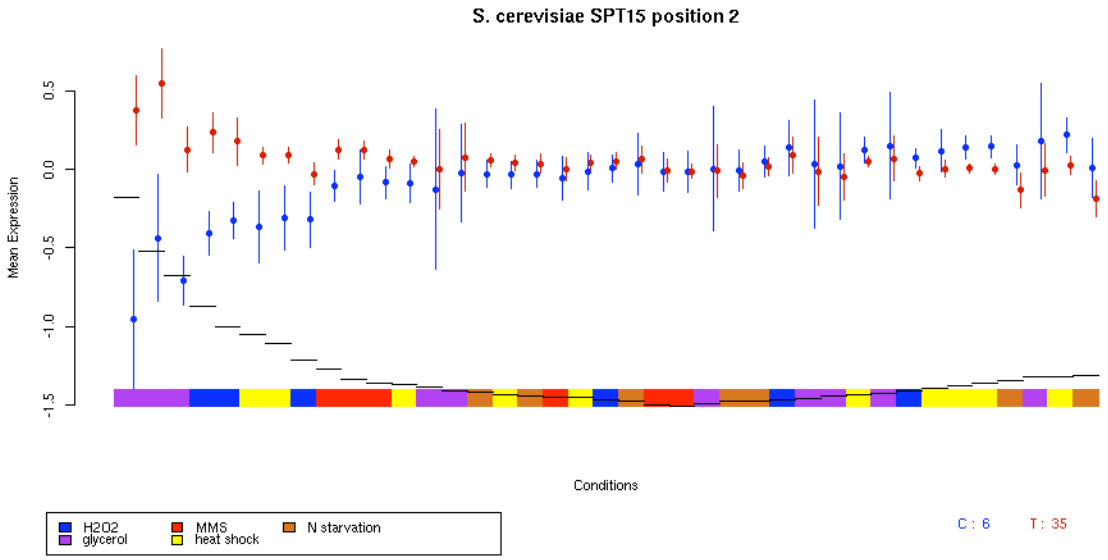
S. cerevisiae SPT15 position 2
Mean Expression
0.5
0.0
-0.5
-1.0
-1.5
Conditions
H2O2
glycerol
MMS
heat shock
N starvation
C : 6
T : 35

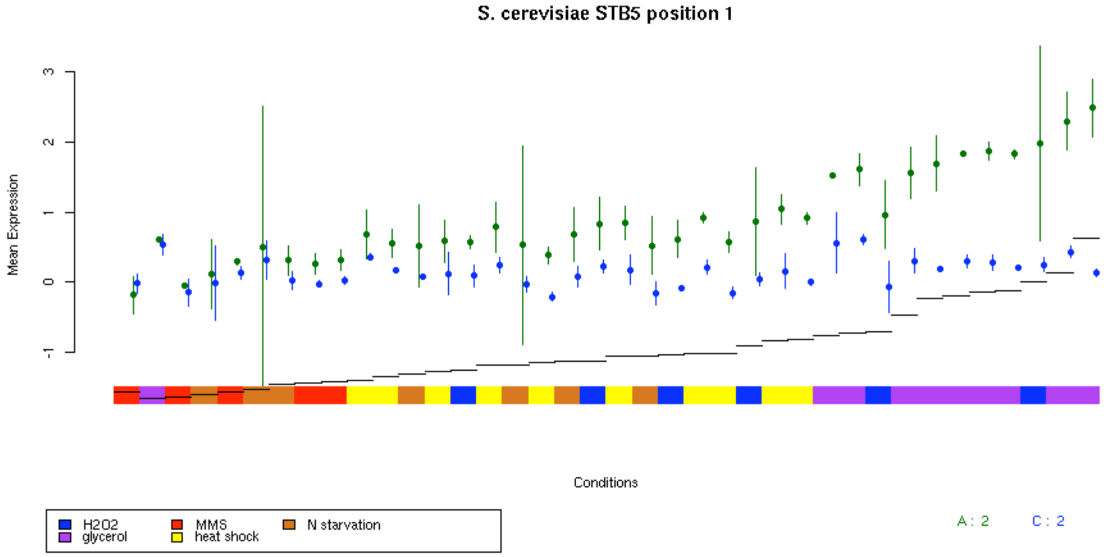

S. cerevisiae STB5 position 1
Mean Expression
Conditions
H2O2
glycerol
MMS
heat shock
N starvation
A : 2
C : 2

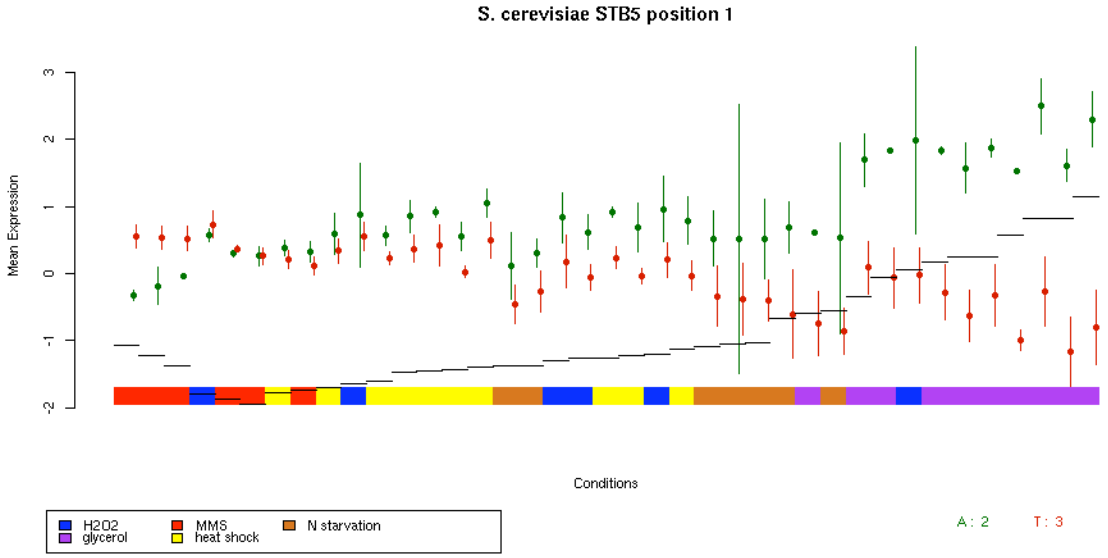

S. cerevisiae STB5 position 1
Mean Expression
Conditions
H2O2
glycerol
MMS
heat shock
N starvation
A : 2
T : 3

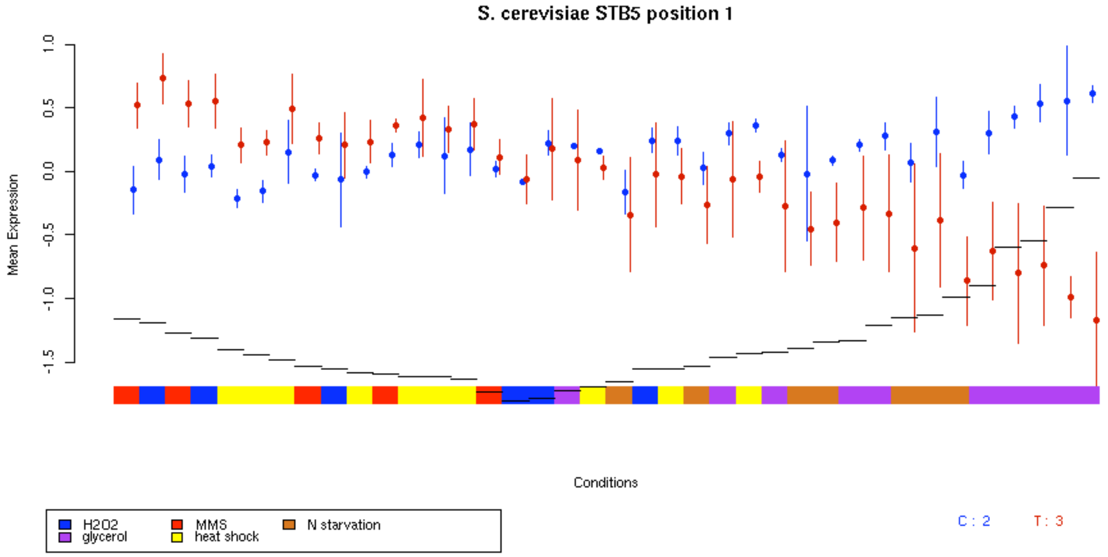
S. cerevisiae STB5 position 1
Mean Expression
1.0
0.5
0.0
-0.5
-1.0
-1.5
Conditions
H2O2
glycerol
MMS
heat shock
N starvation
C : 2
T : 3

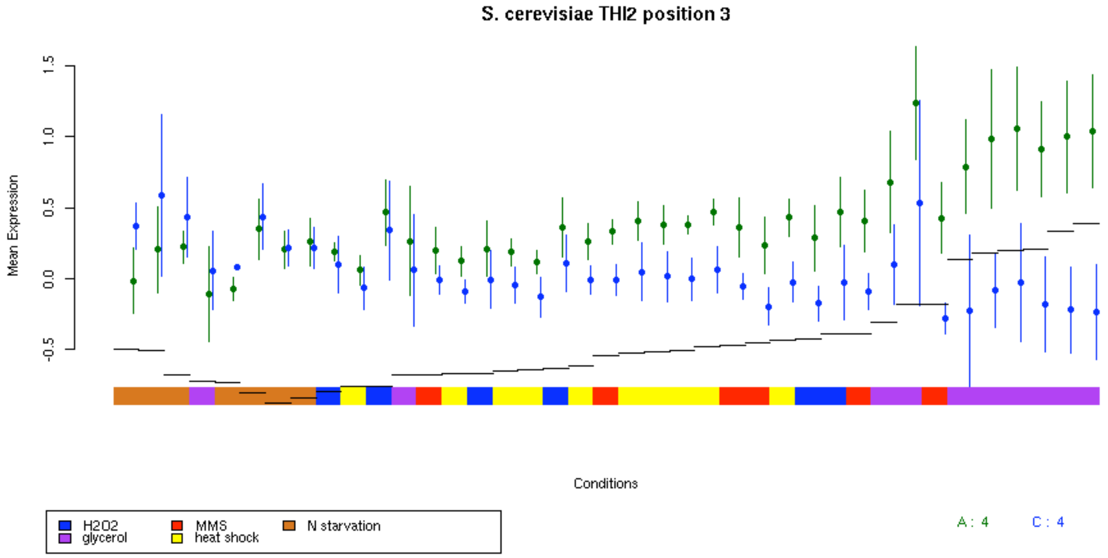
S. cerevisiae THI2 position 3
Mean Expression
1.5
1.0
0.5
0.0
-0.5
Conditions
H2O2
glycerol
MMS
heat shock
N starvation
A : 4
C : 4

S. kudriavzevii RAP1 position 10

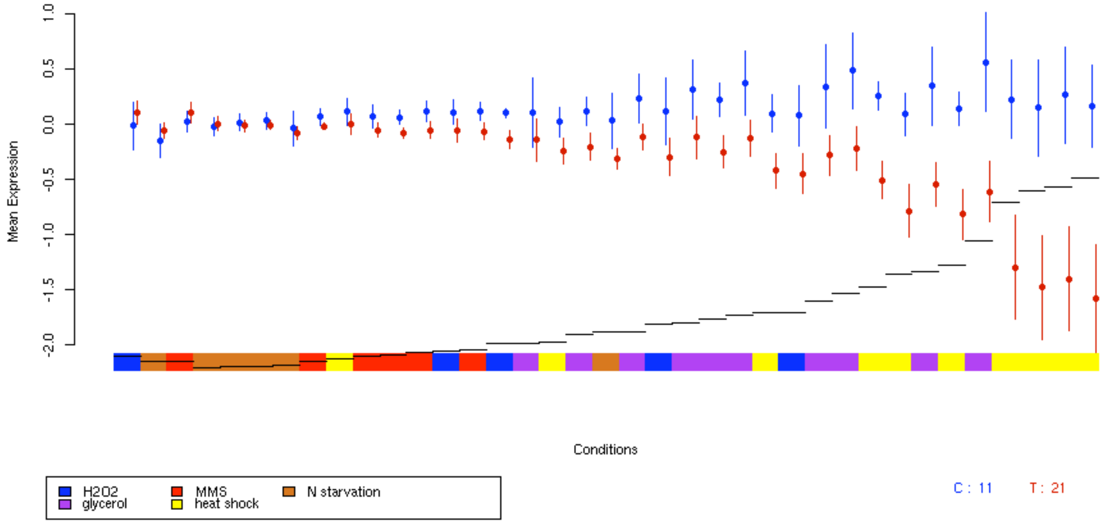

S. kudriavzevii ABF1 position 6

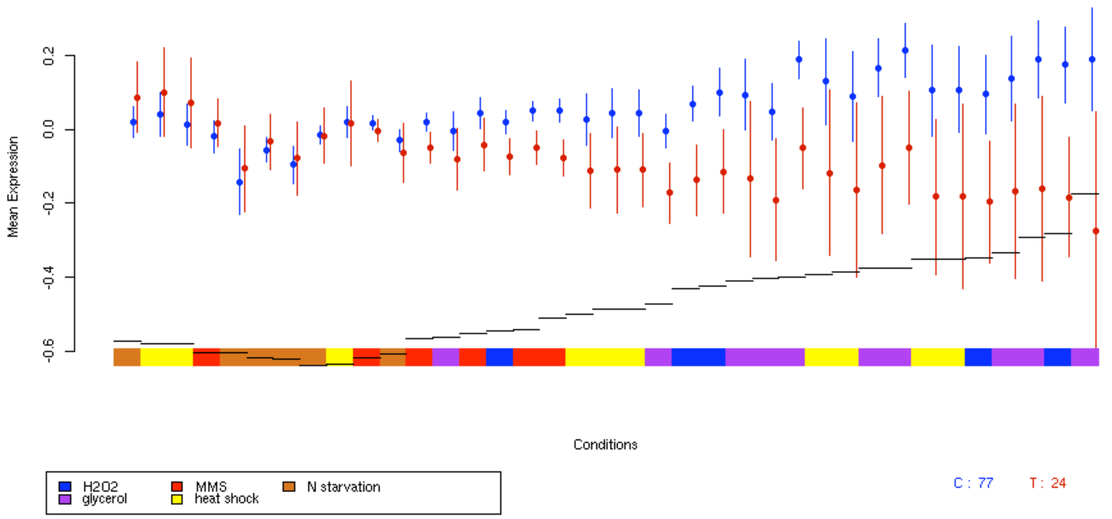

S. kudriavzevii doublePAC position 7

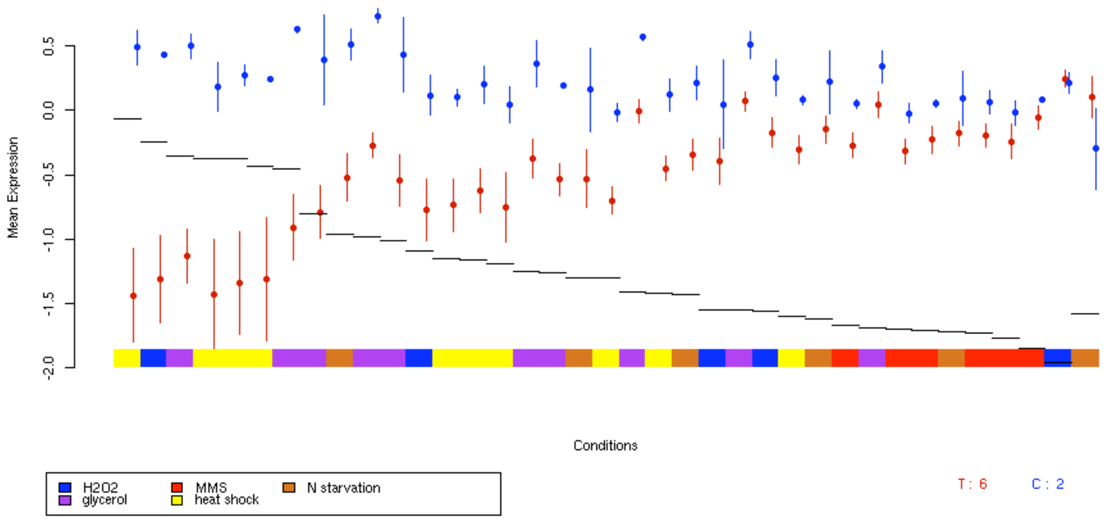

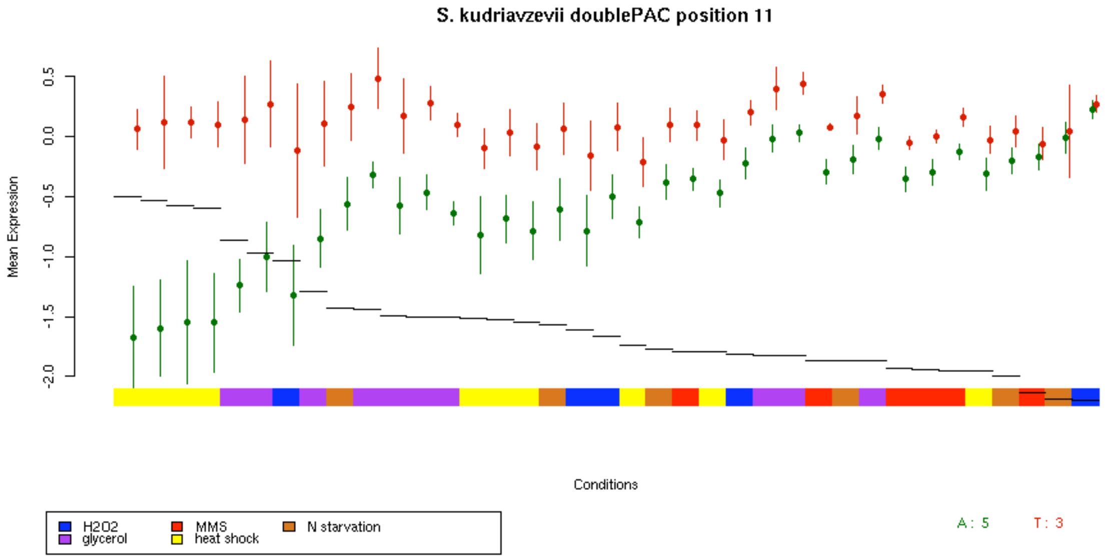
S. kudriavzevii doublePAC position 11
Mean Expression
0.5
0.0
-0.5
-1.0
-1.5
-2.0
Conditions
H2O2
glycerol
MMS
heat shock
N starvation
A : 5
T : 3

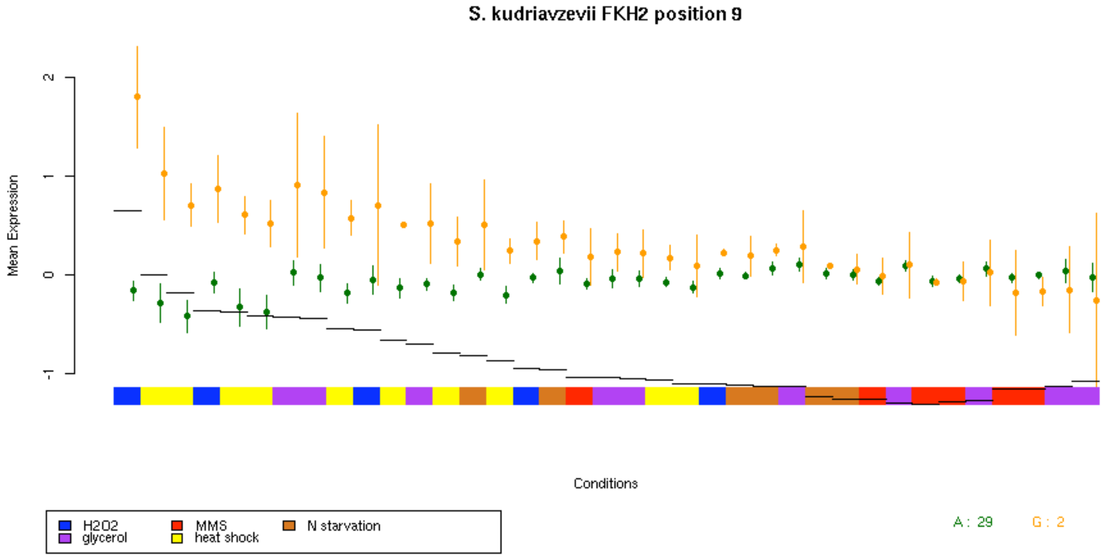
S. kudriavzevii FKH2 position 9
Mean Expression
2
1
0
-1
Conditions
H2O2
glycerol
MMS
heat shock
N starvation
A : 29
G : 2

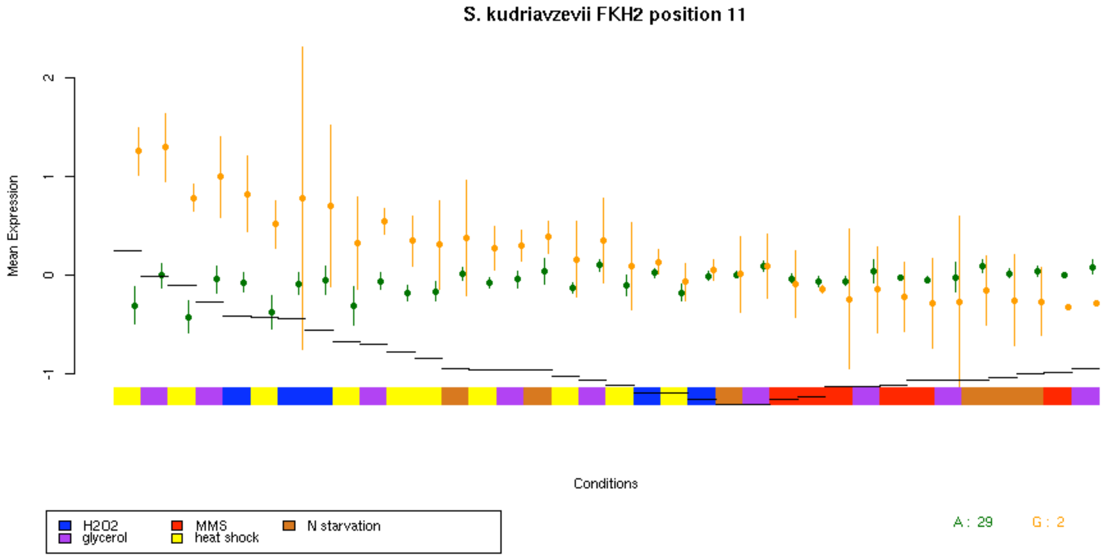
S. kudriavzevii FKH2 position 11
Mean Expression
2
1
0
-1
Conditions
H2O2
glycerol
MMS
heat shock
N starvation
A : 29
G : 2

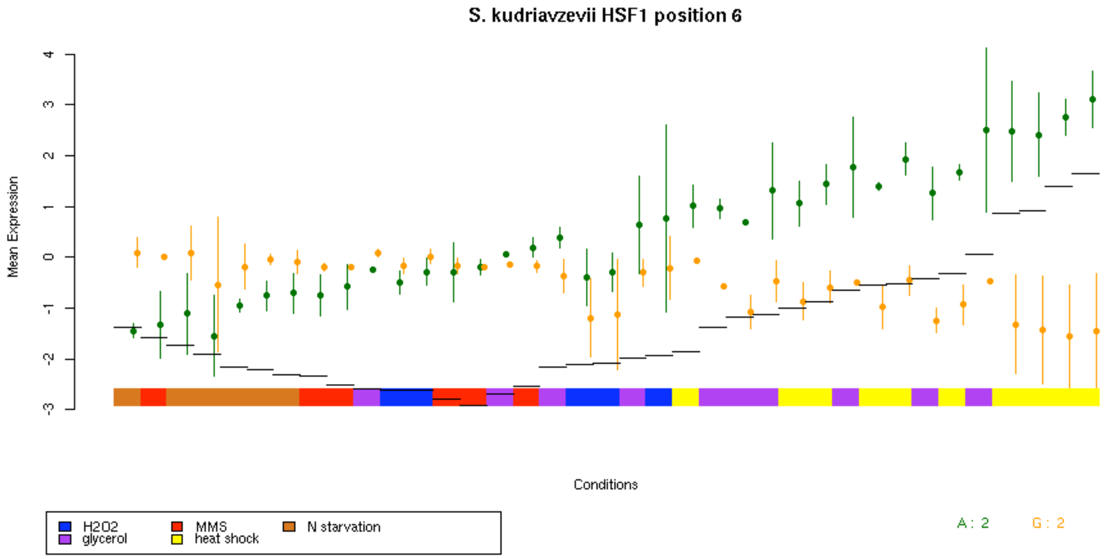
S. kudriavzevii HSF1 position 6
Mean Expression
4
3
2
1
0
-1
-2
-3
Conditions
H2O2
glycerol
MMS
heat shock
N starvation
A : 2
G : 2

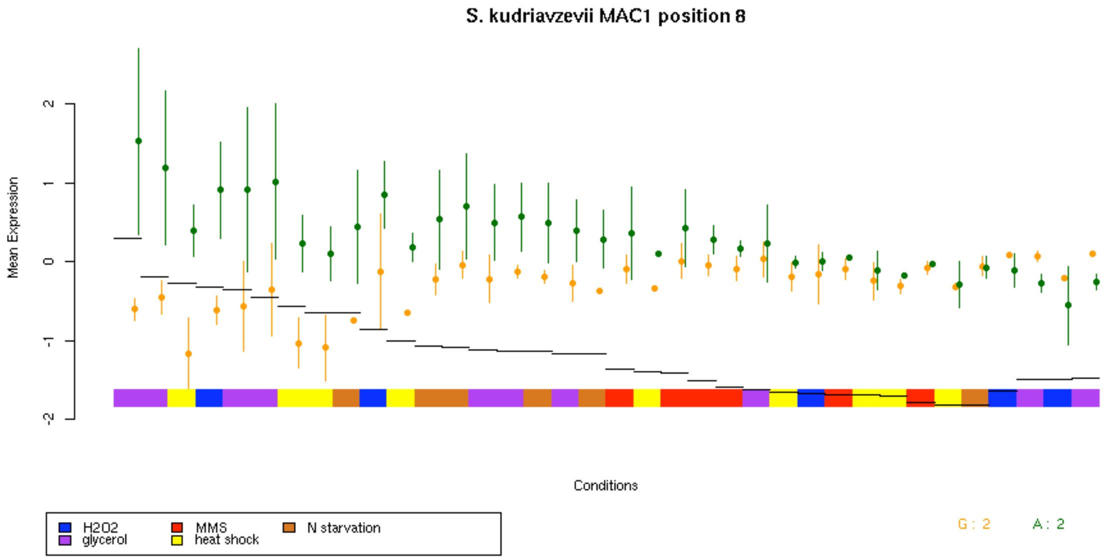
S. kudriavzevii MAC1 position 8
Mean Expression
2
1
0
-1
-2
Conditions
H2O2
glycerol
MMS
heat shock
N starvation
G : 2
A : 2

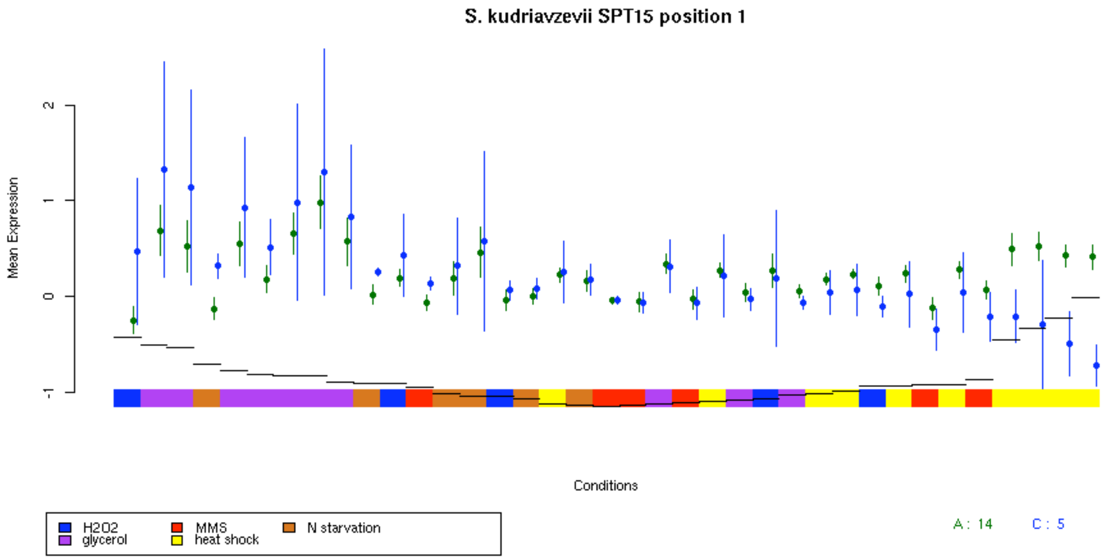
S. kudriavzevii SPT15 position 1
Mean Expression
2
1
0
-1
Conditions
H2O2
glycerol
MMS
heat shock
N starvation
A : 14
C : 5

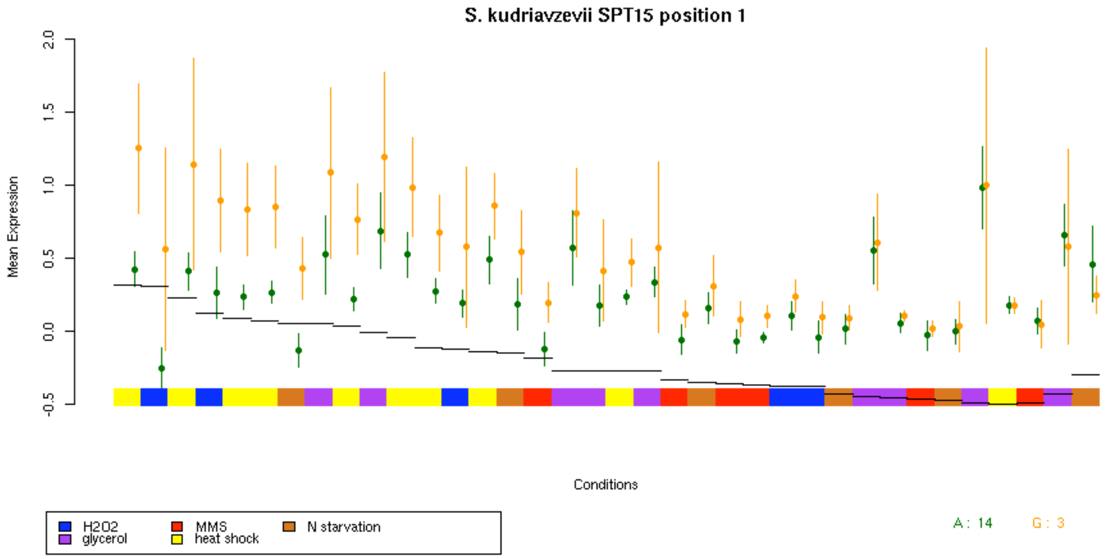
S. kudriavzevii SPT15 position 1
Mean Expression
-0.5
0.0
0.5
1.0
1.5
2.0
Conditions
H2O2
glycerol
MMS
heat shock
N starvation
A : 14
G : 3

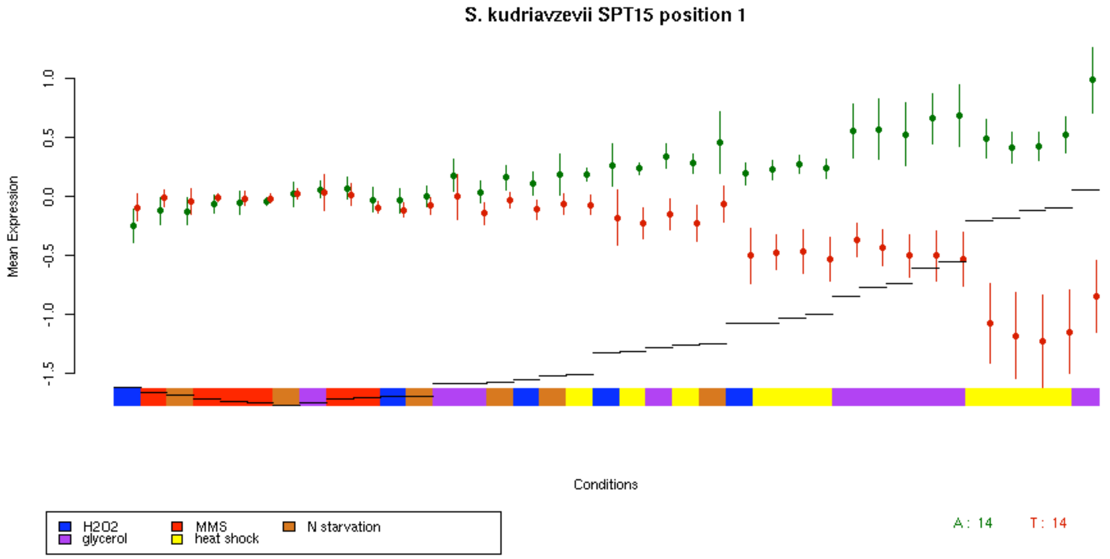
S. kudriavzevii SPT15 position 1
Mean Expression
-1.5
-1.0
-0.5
0.0
0.5
1.0
Conditions
H2O2
glycerol
MMS
heat shock
N starvation
A : 14
T : 14

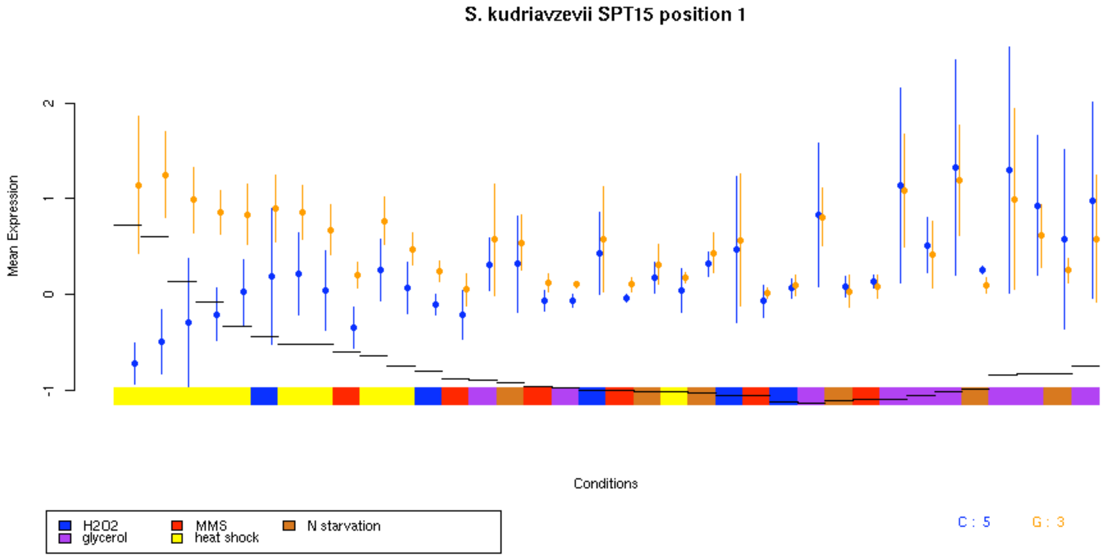
S. kudriavzevii SPT15 position 1
Mean Expression
-1
0
1
2
Conditions
H2O2
glycerol
MMS
heat shock
N starvation
C : 5
G : 3

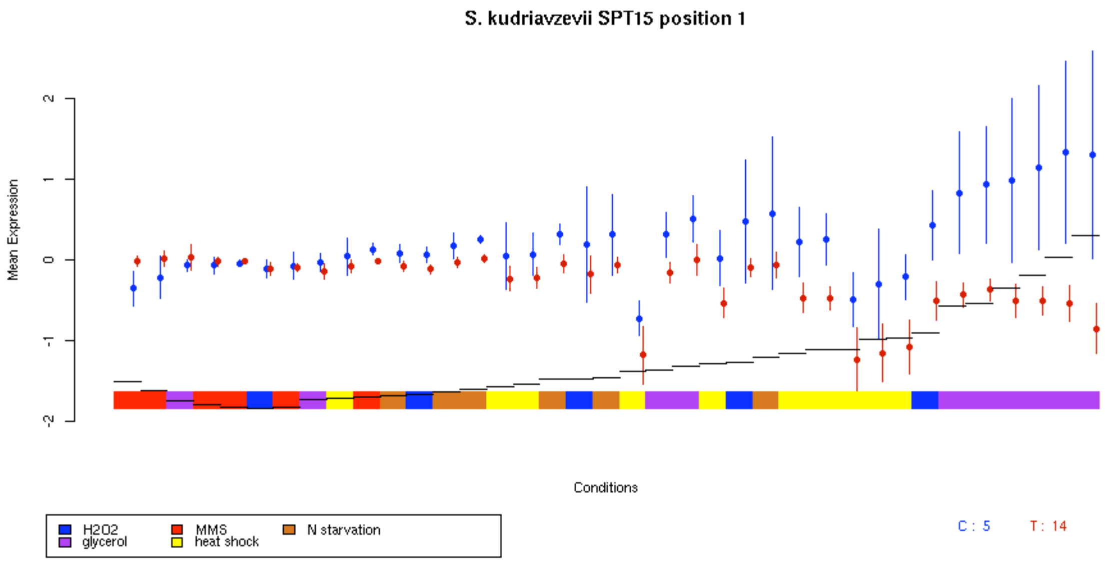
S. kudriavzevii SPT15 position 1
Mean Expression
2
1
0
-1
-2
Conditions
H2O2
glycerol
MMS
heat shock
N starvation
C : 5
T : 14

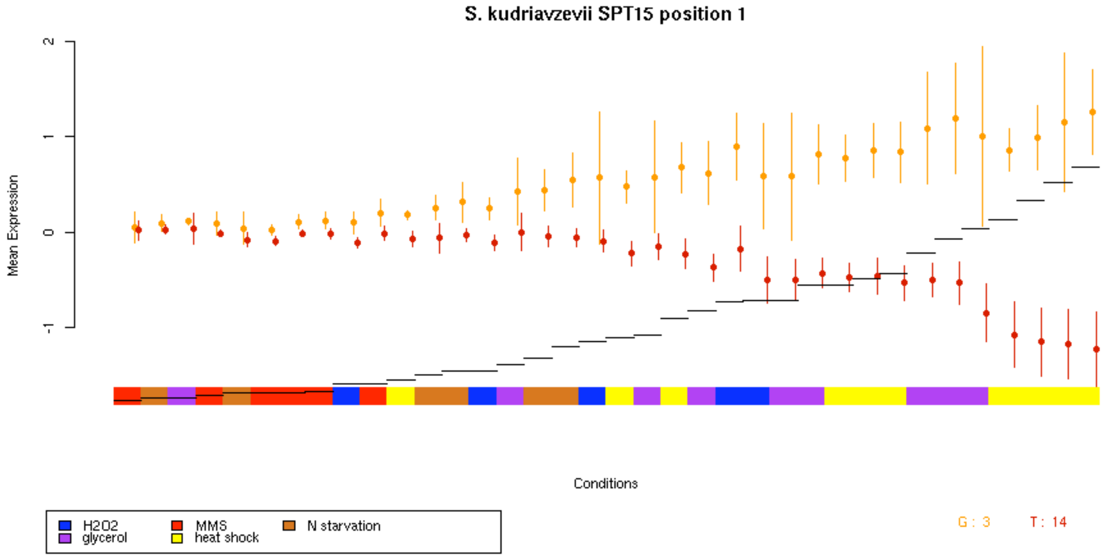
S. kudriavzevii SPT15 position 1
Mean Expression
2
1
0
-1
Conditions
H2O2
glycerol
MMS
heat shock
N starvation
G : 3
T : 14

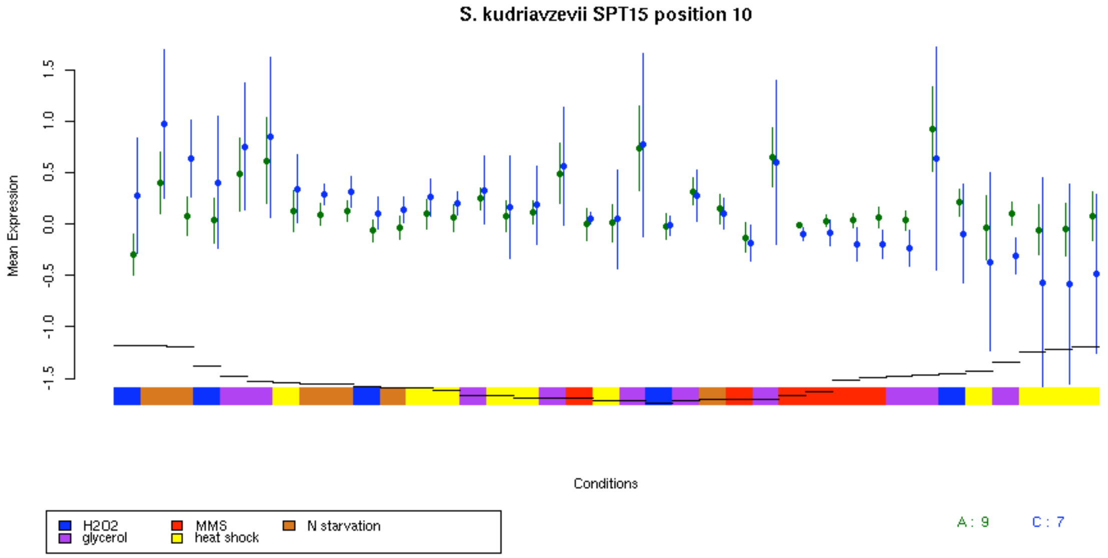
S. kudriavzevii SPT15 position 10
Mean Expression
1.5
1.0
0.5
0.0
-0.5
-1.0
-1.5
Conditions
H2O2
glycerol
MMS
heat shock
N starvation
A : 9
C : 7

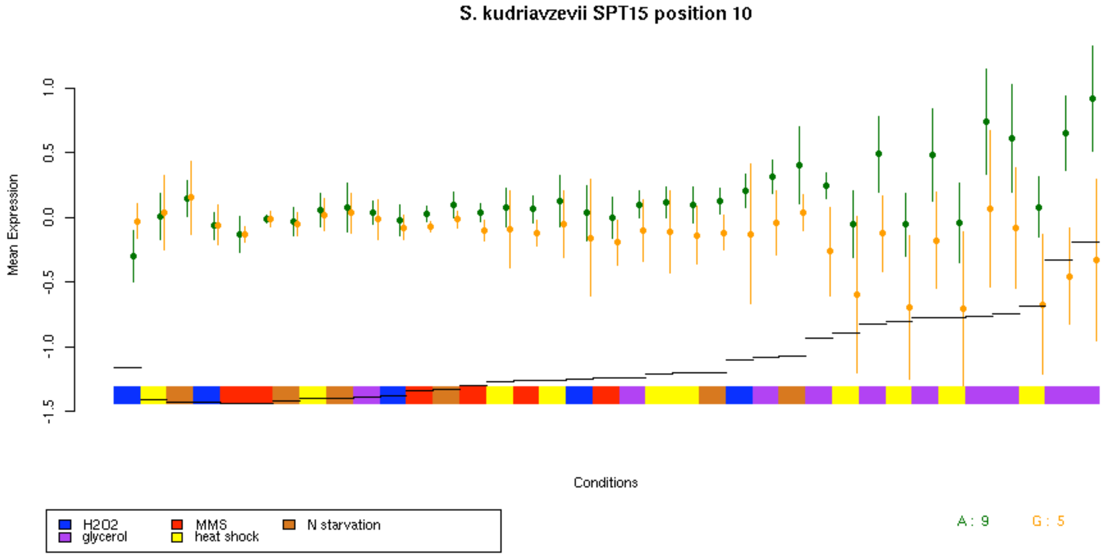
S. kudriavzevii SPT15 position 10
Mean Expression
Conditions
H2O2
glycerol
MMS
heat shock
N starvation
A : 9
G : 5

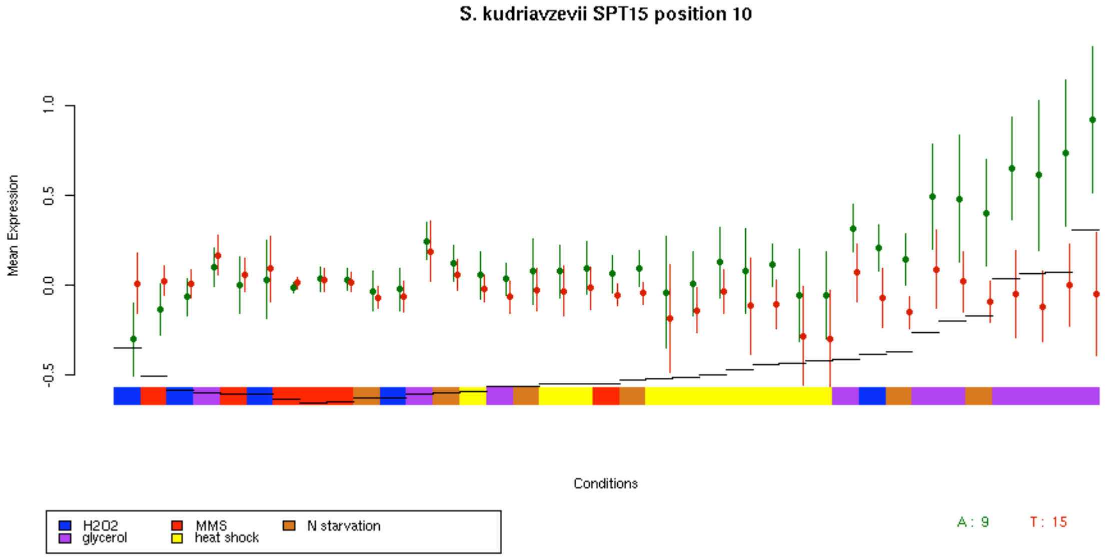
S. kudriavzevii SPT15 position 10
Mean Expression
Conditions
H2O2
glycerol
MMS
heat shock
N starvation
A : 9
T : 15

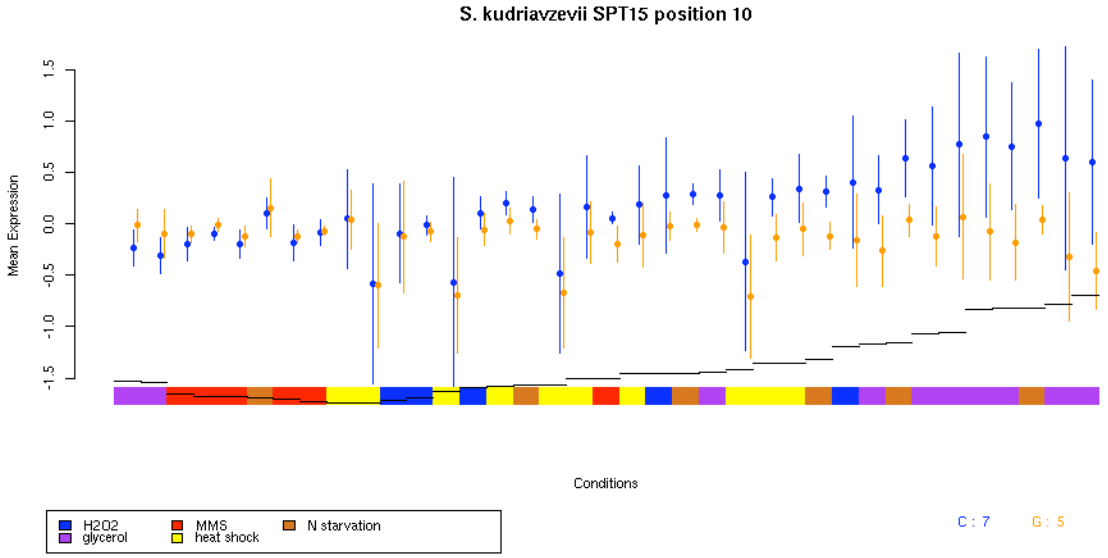
S. kudriavzevii SPT15 position 10
Mean Expression
Conditions
H2O2
glycerol
MMS
heat shock
N starvation
C : 7
G : 5

S. kudriavzevii SPT15 position 10

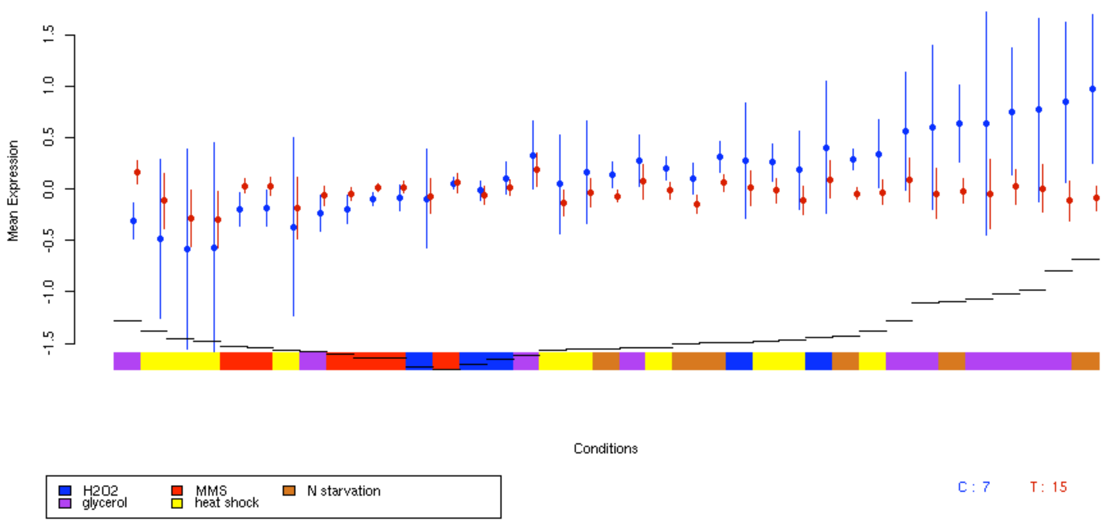

S. kudriavzevii SPT15 position 10

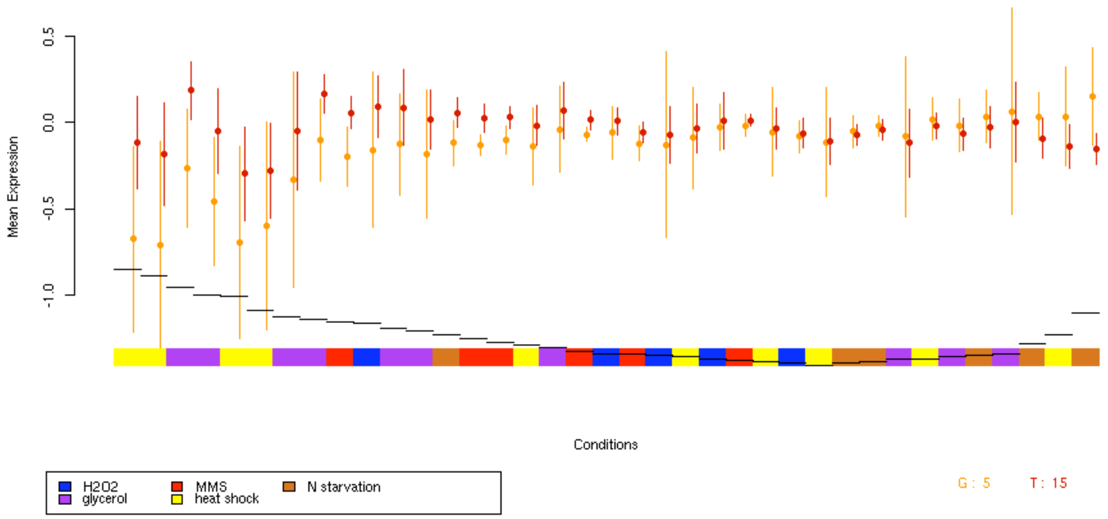

S. kudriavzevii SPT15 position 11

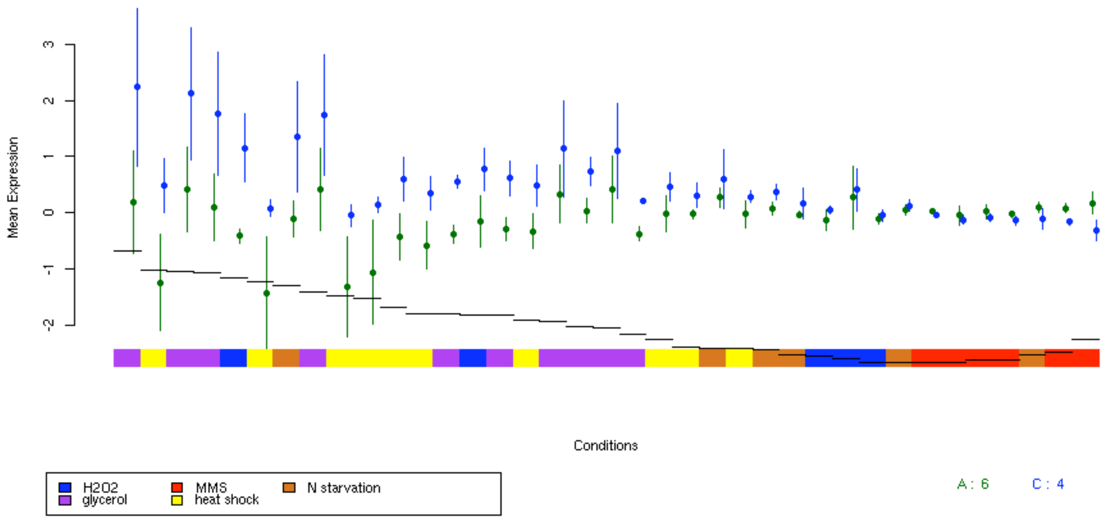

S. kudriavzevii SPT15 position 11

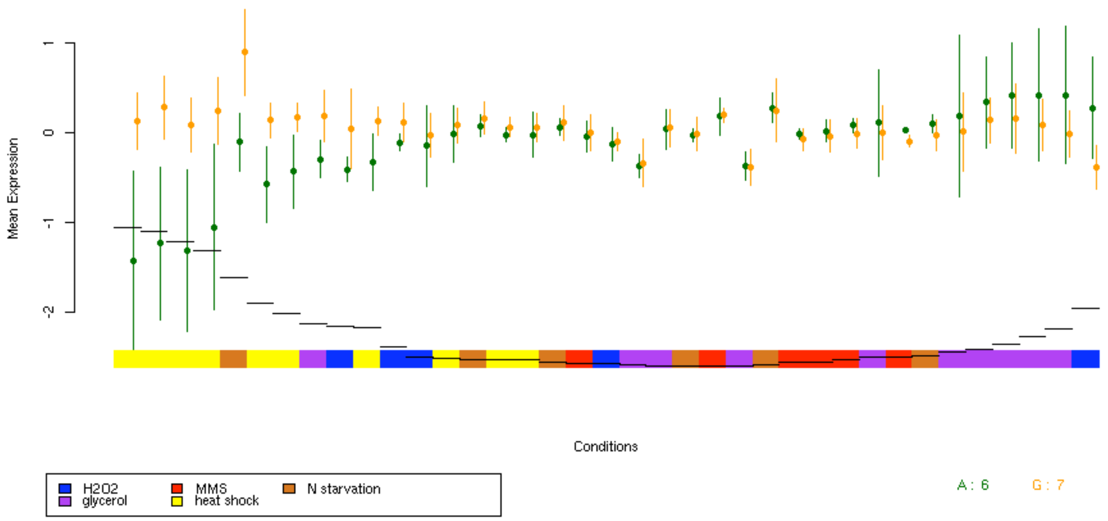

S. kudriavzevii SPT15 position 11

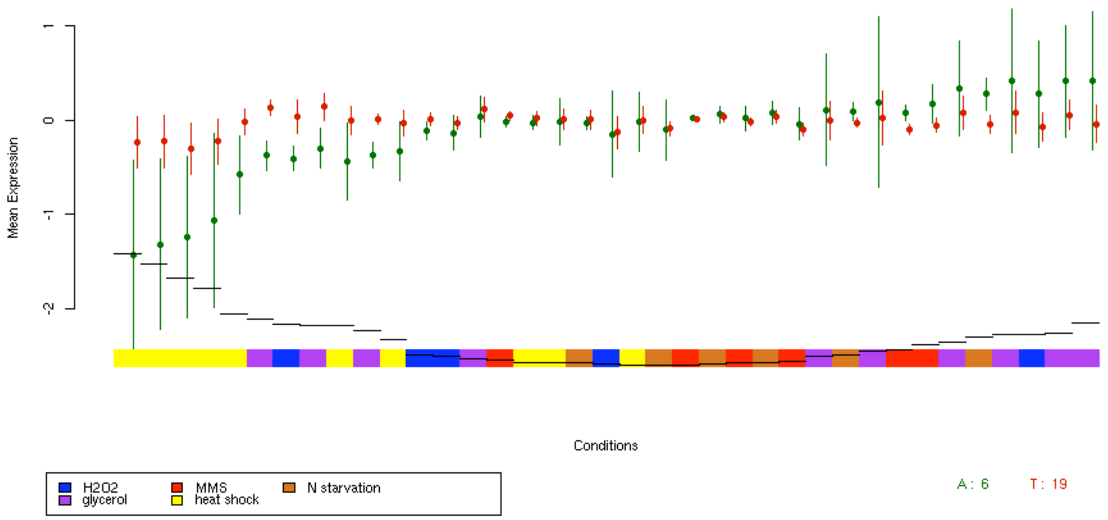

S. kudriavzevii SPT15 position 11

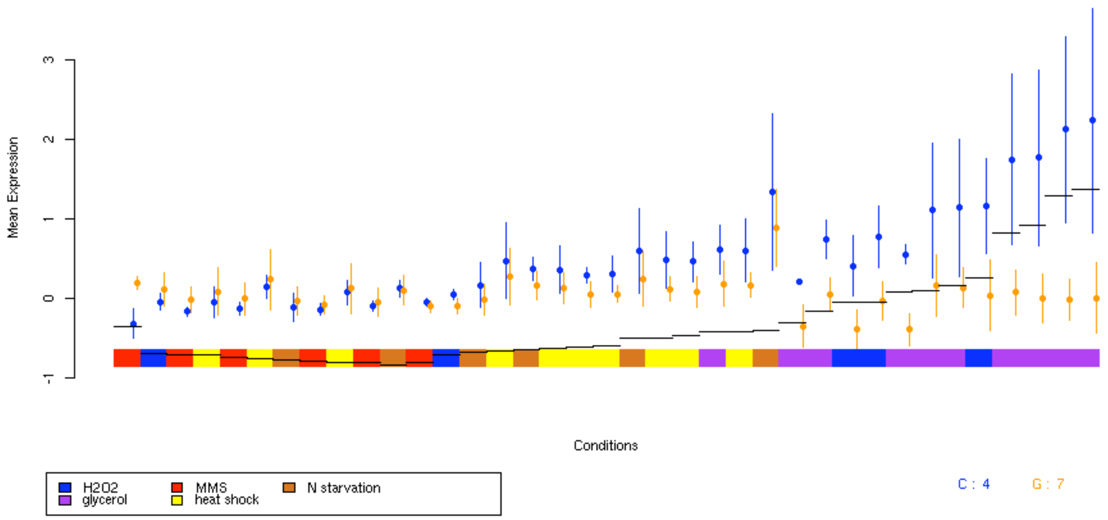

S. kudriavzevii SPT15 position 11

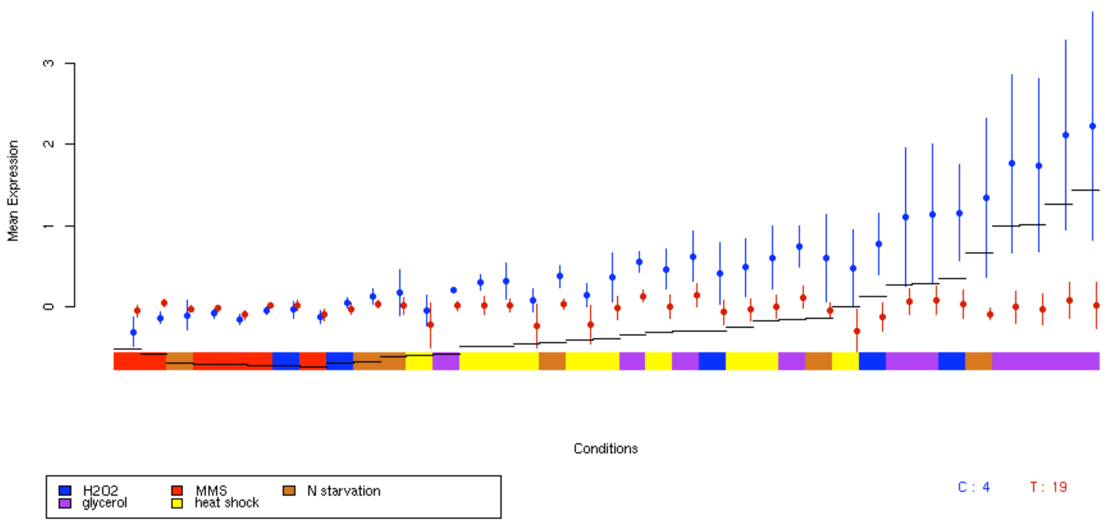

S. kudriavzevii SPT15 position 11

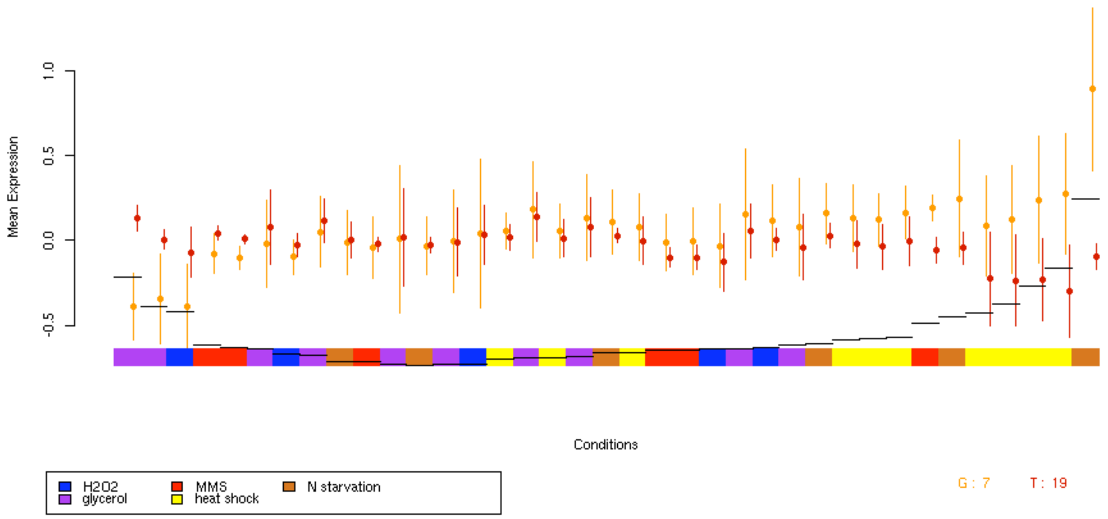

S. kudriavzevii STB5 position 6

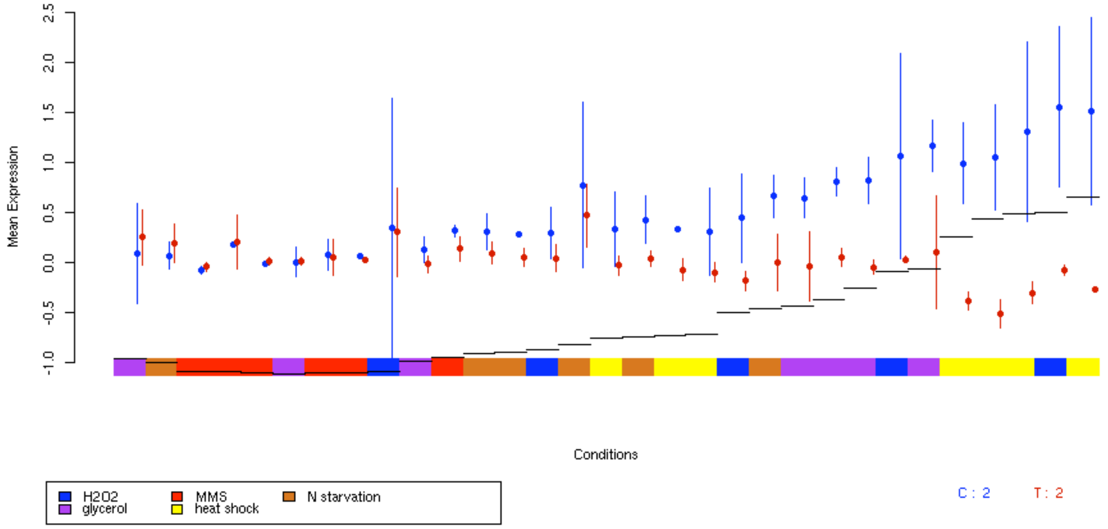

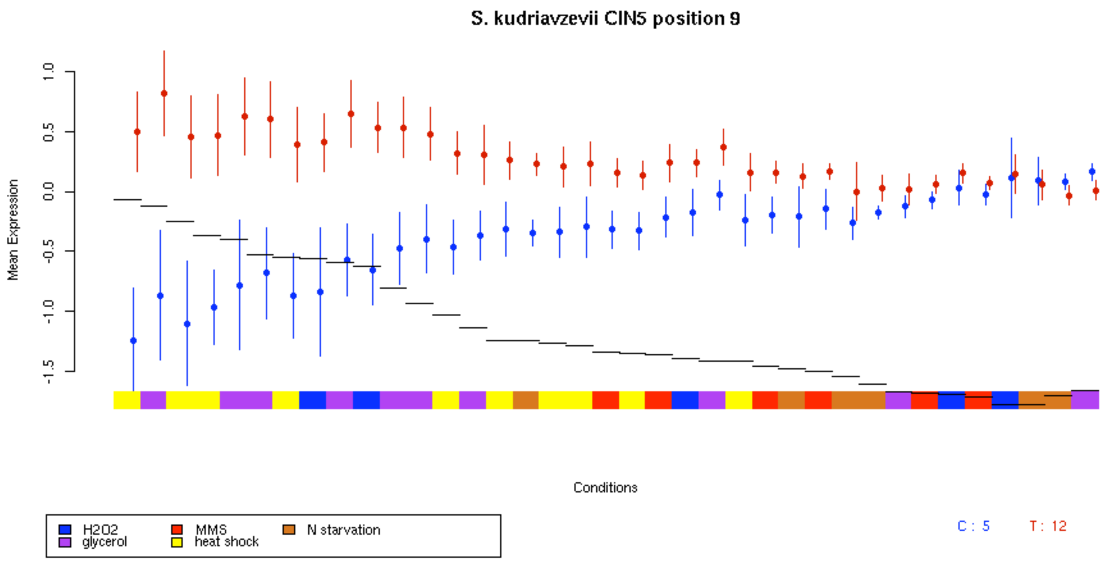
S. kudriavzevii CIN5 position 9
Mean Expression
Conditions
H2O2
glycerol
MMS
heat shock
N starvation
C : 5
T : 12

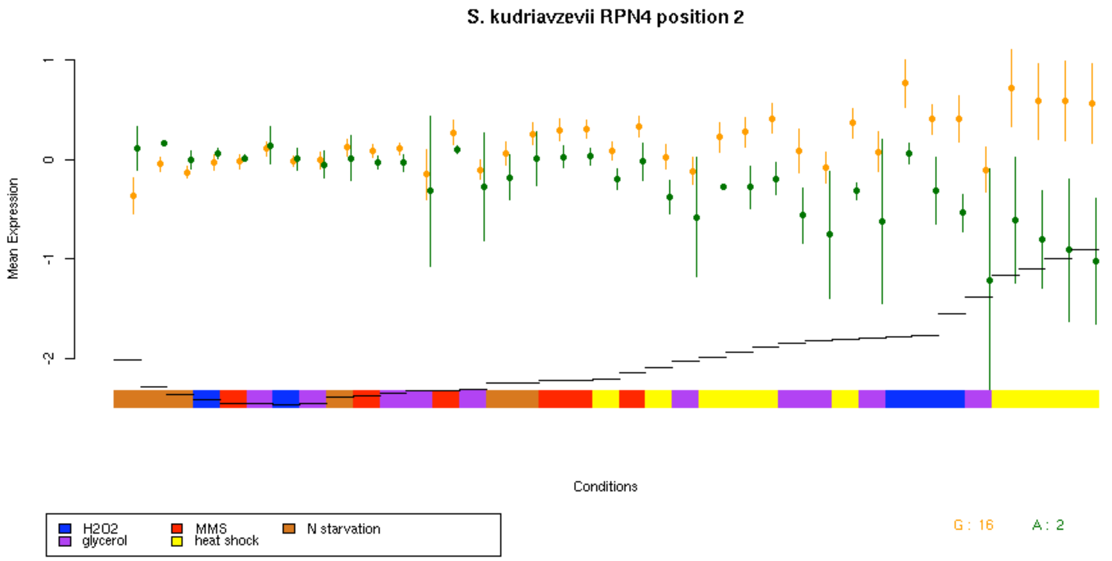
S. kudriavzevii RPN4 position 2
Mean Expression
Conditions
H2O2
glycerol
MMS
heat shock
N starvation
G : 16
A : 2

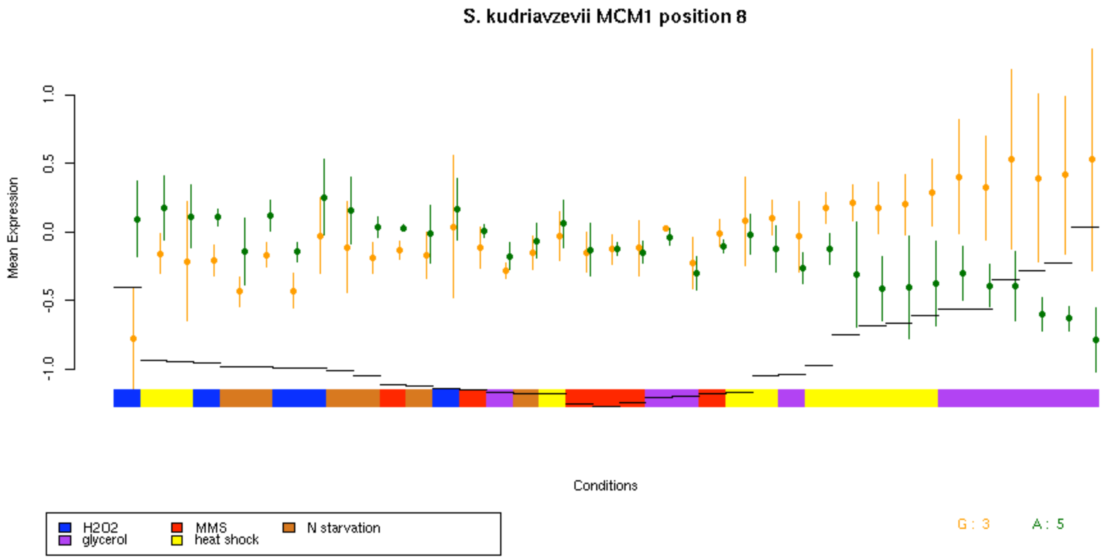
S. kudriavzevii MCM1 position 8
Mean Expression
Conditions
H2O2
glycerol
MMS
heat shock
N starvation
G : 3
A : 5

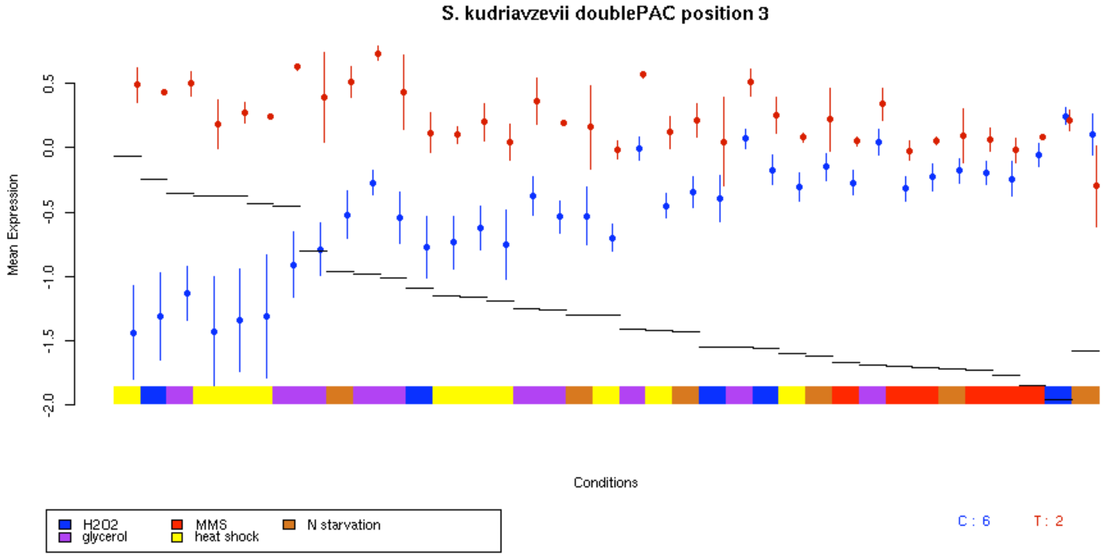
S. kudriavzevii doublePAC position 3
Mean Expression
0.5
0.0
-0.5
-1.0
-1.5
-2.0
Conditions
H2O2
glycerol
MMS
heat shock
N starvation
C : 6
T : 2

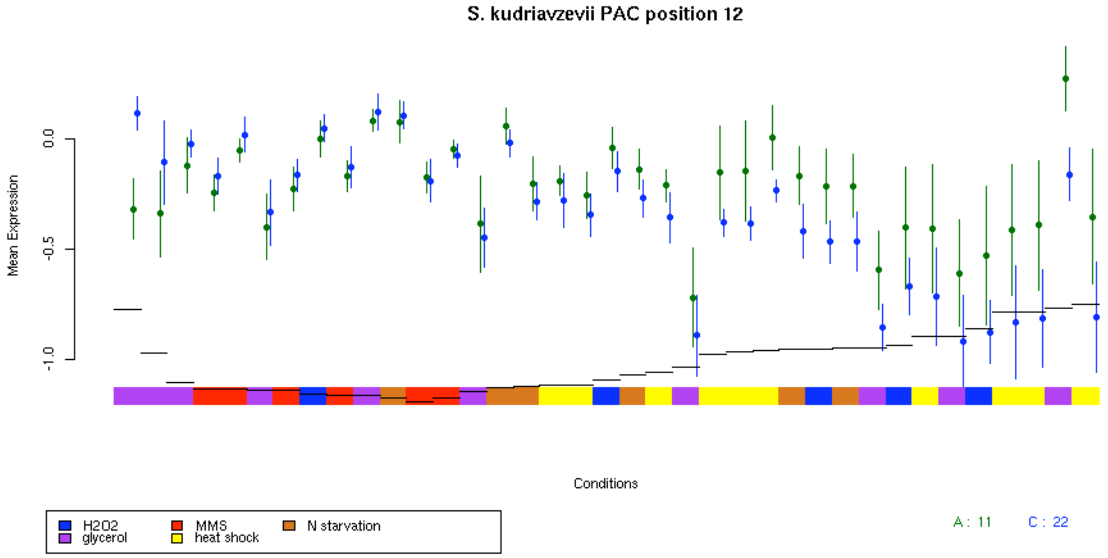
S. kudriavzevii PAC position 12
Mean Expression
0.0
-0.5
-1.0
Conditions
H2O2
glycerol
MMS
heat shock
N starvation
A : 11
C : 22

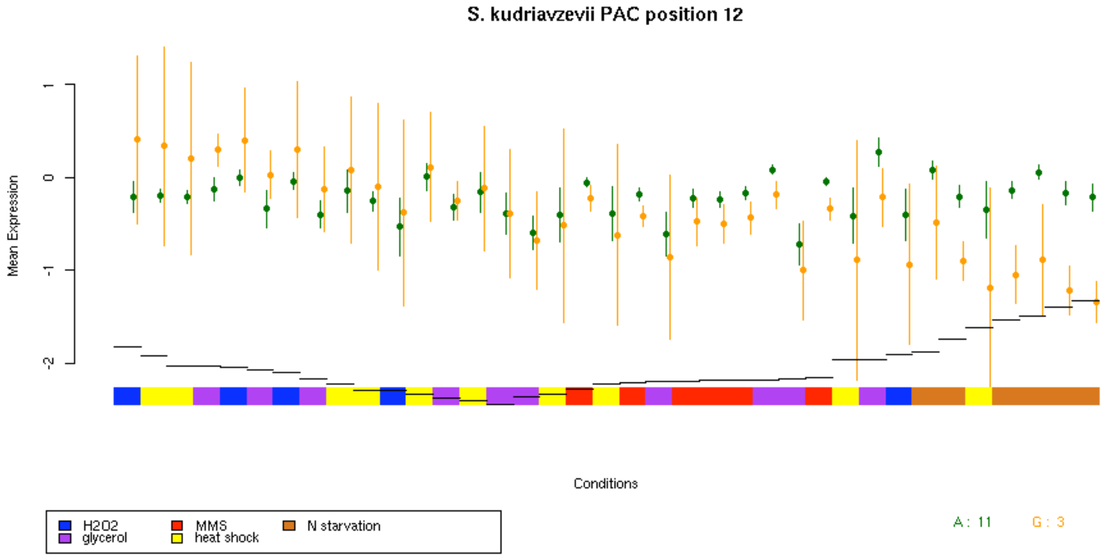
S. kudriavzevii PAC position 12
Mean Expression
1
0
-1
-2
Conditions
H2O2
glycerol
MMS
heat shock
N starvation
A : 11
G : 3

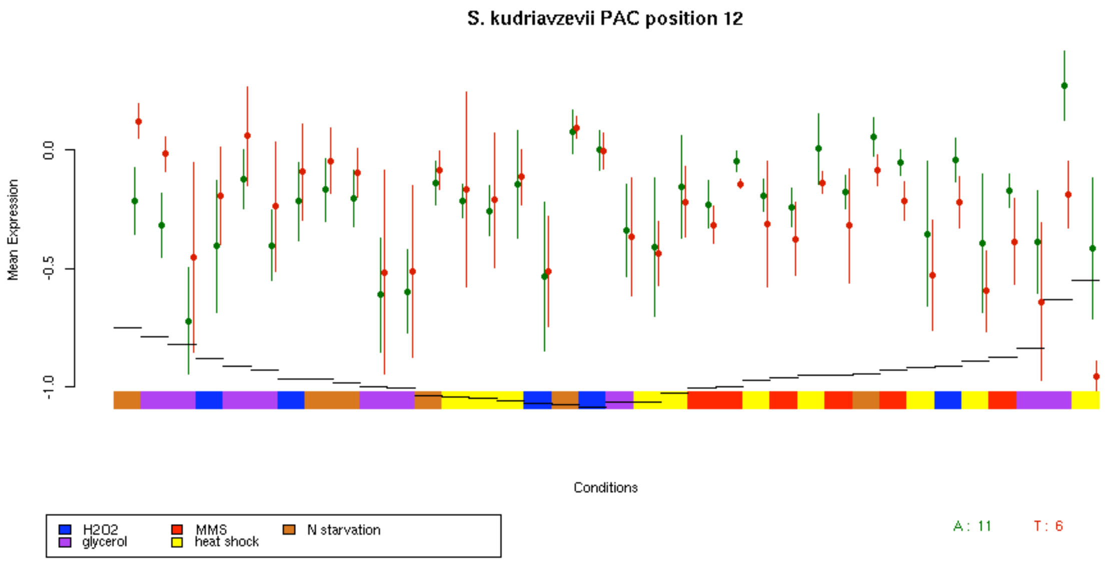
S. kudriavzevii PAC position 12
Mean Expression
0.0
-0.5
-1.0
Conditions
H2O2
glycerol
MMS
heat shock
N starvation
A : 11
T : 6

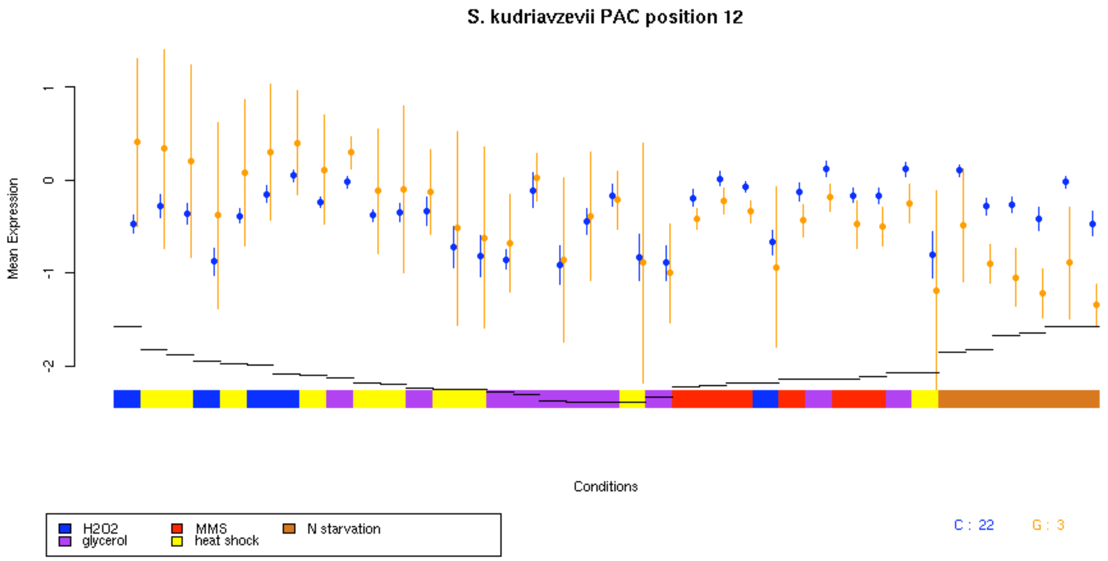
S. kudriavzevii PAC position 12
Mean Expression
1
0
-1
-2
Conditions
H2O2
glycerol
MMS
heat shock
N starvation
C : 22
G : 3

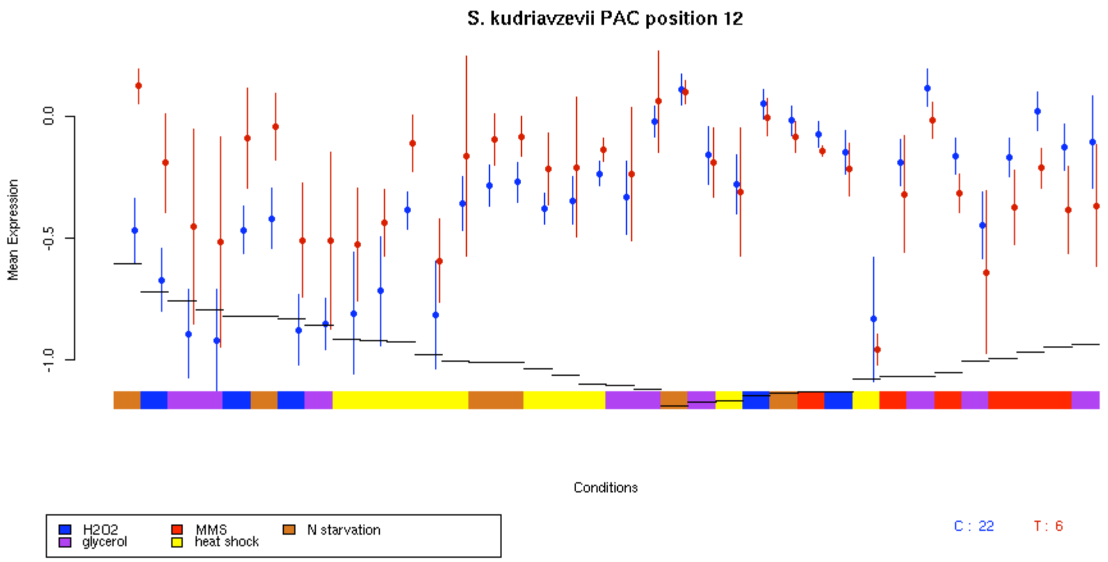

S. kudriavzevii PAC position 12
Mean Expression
0.0
-0.5
-1.0
Conditions
H2O2
glycerol
MMS
heat shock
N starvation
C : 22
T : 6

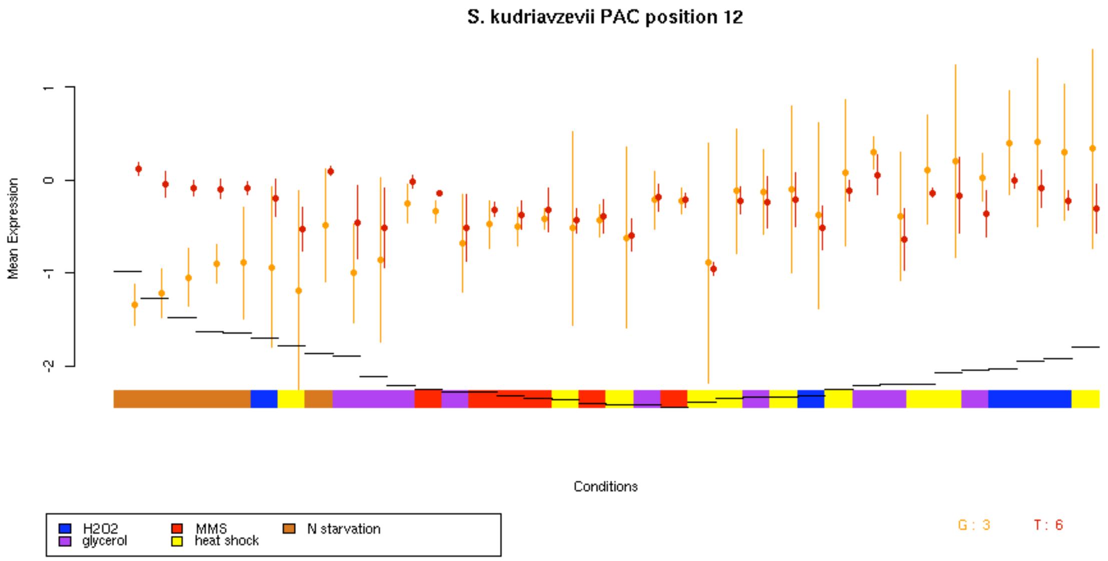

S. kudriavzevii PAC position 12
Mean Expression
1
0
-1
-2
Conditions
H2O2
glycerol
MMS
heat shock
N starvation
G : 3
T : 6

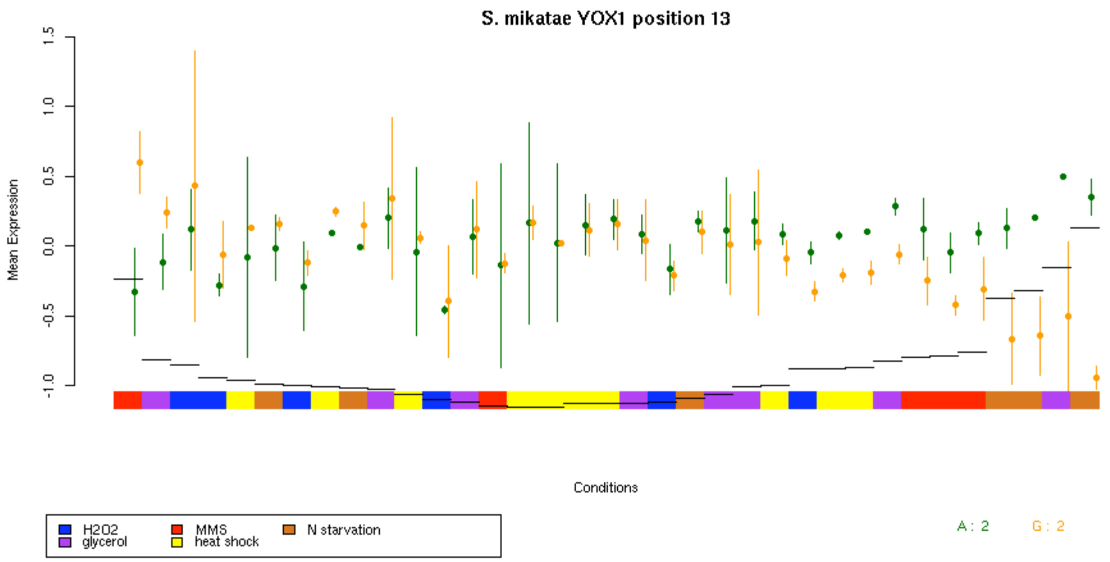
S. mikatae YOX1 position 13
Mean Expression
1.5
1.0
0.5
0.0
-0.5
-1.0
Conditions
H2O2
glycerol
MMS
heat shock
N starvation
A : 2
G : 2

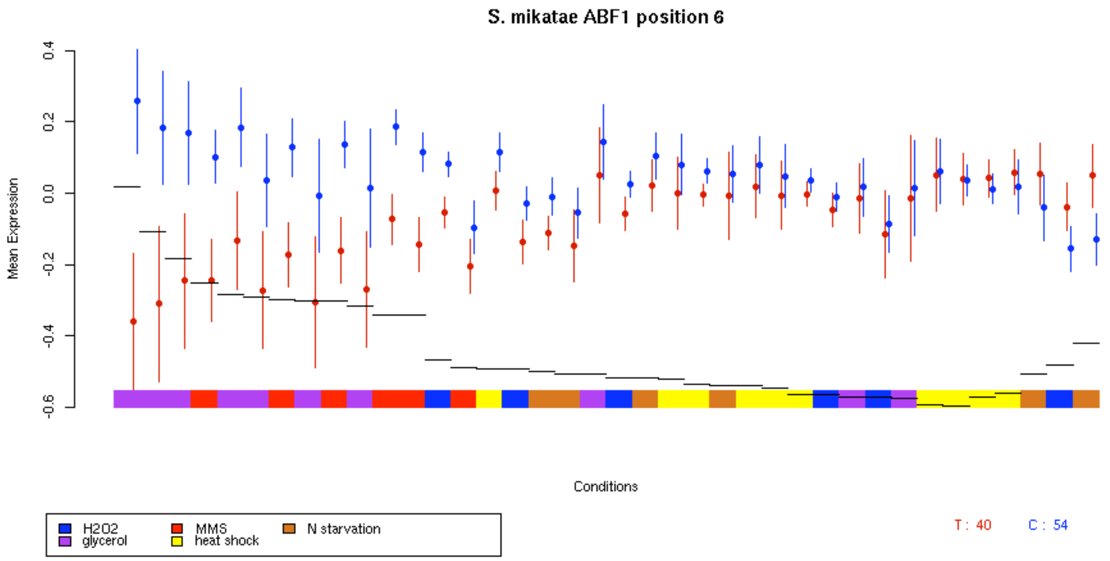
S. mikatae ABF1 position 6
Mean Expression
0.4
0.2
0.0
-0.2
-0.4
-0.6
Conditions
H2O2
glycerol
MMS
heat shock
N starvation
T : 40
C : 54

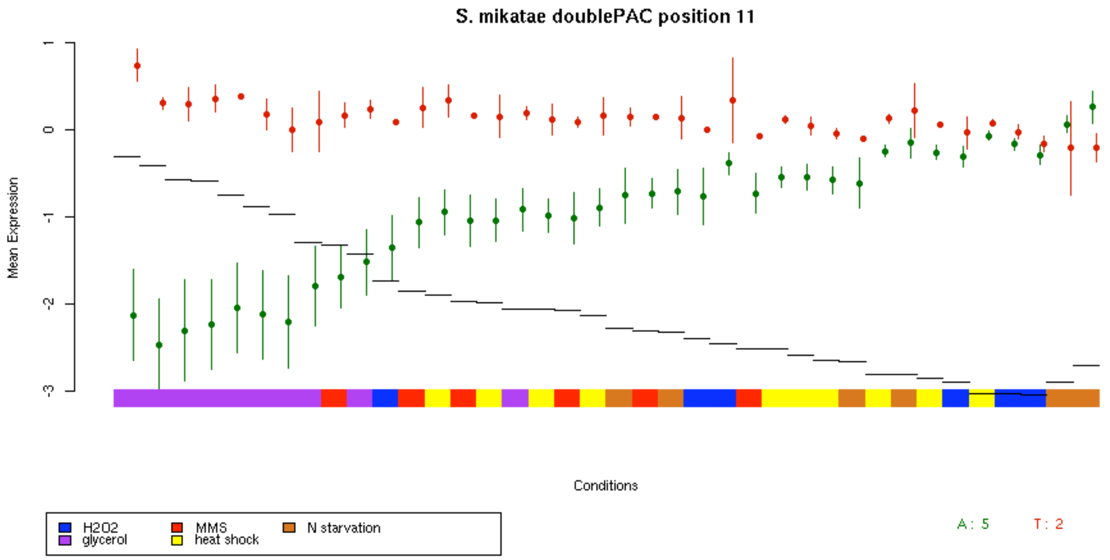
S. mikatae doublePAC position 11
Mean Expression
1
0
-1
-2
-3
Conditions
H2O2
glycerol
MMS
heat shock
N starvation
A : 5
T : 2

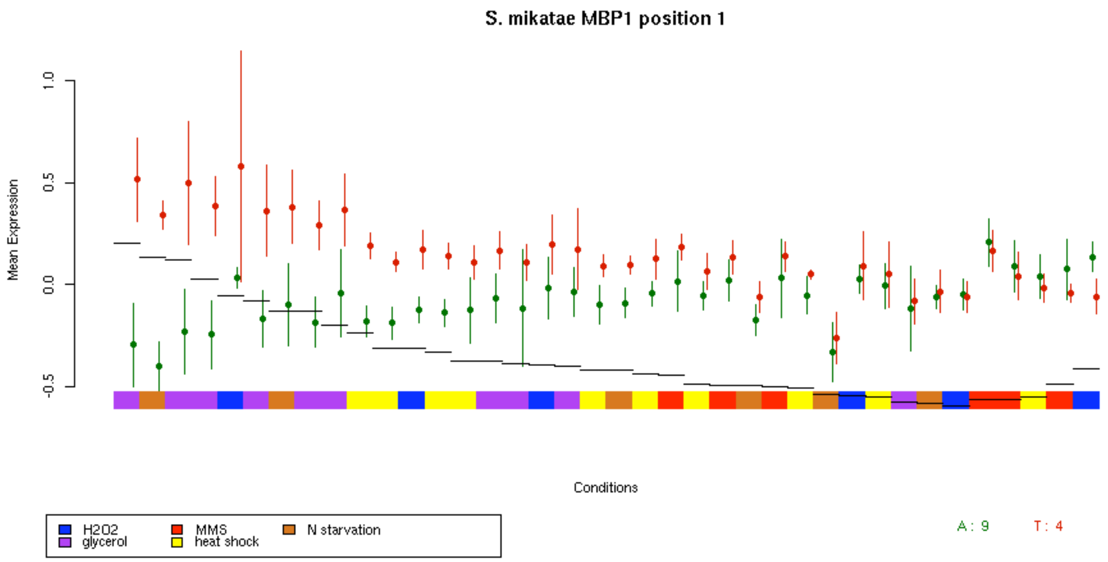
S. mikatae MBP1 position 1
Mean Expression
1.0
0.5
0.0
-0.5
Conditions
H2O2
glycerol
MMS
heat shock
N starvation
A : 9
T : 4

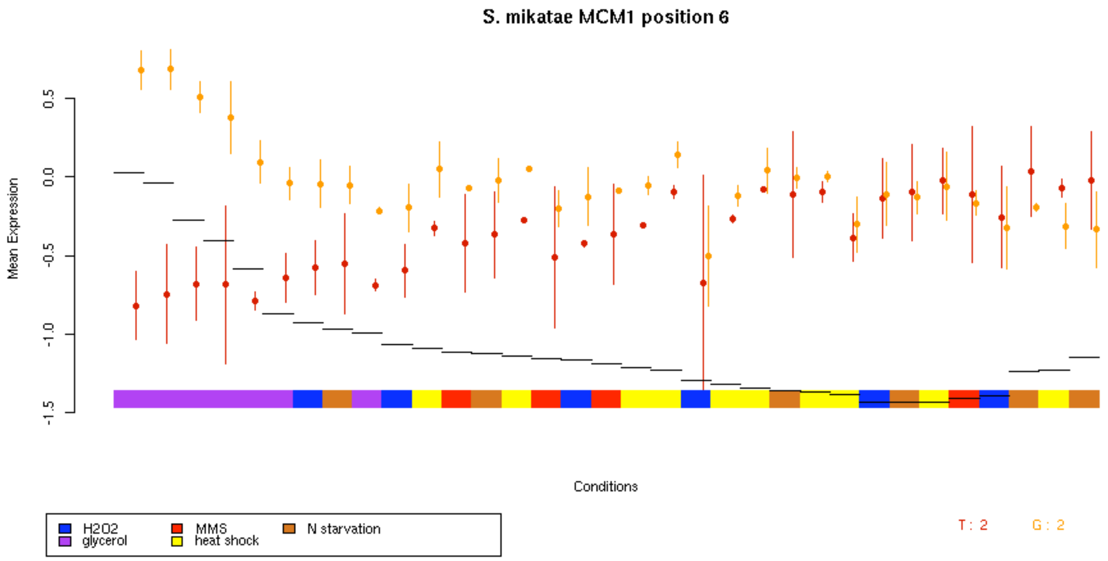
S. mikatae MCM1 position 6
Mean Expression
0.5
0.0
-0.5
-1.0
-1.5
Conditions
H2O2
glycerol
MMS
heat shock
N starvation
T : 2
G : 2

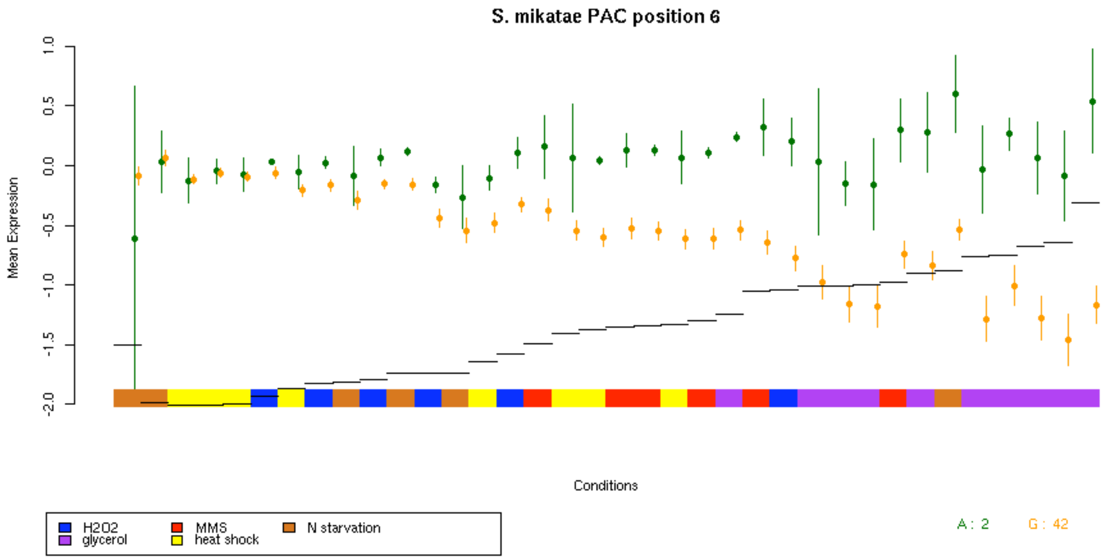
S. mikatae PAC position 6
Mean Expression
1.0
0.5
0.0
-0.5
-1.0
-1.5
-2.0
Conditions
H2O2
glycerol
MMS
heat shock
N starvation
A : 2
G : 42

S. mikatae RAP1 position 10

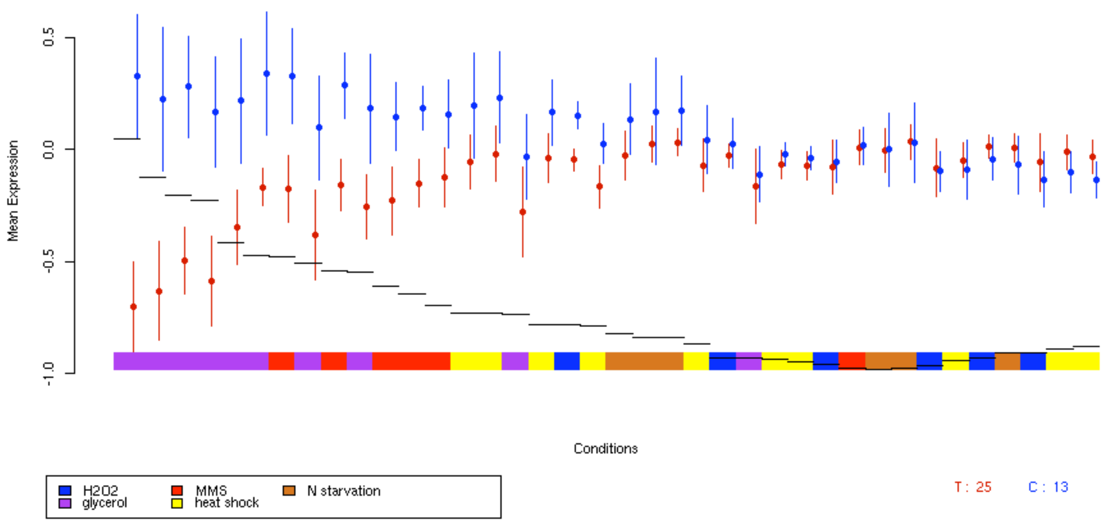

S. mikatae RCS1 position 8

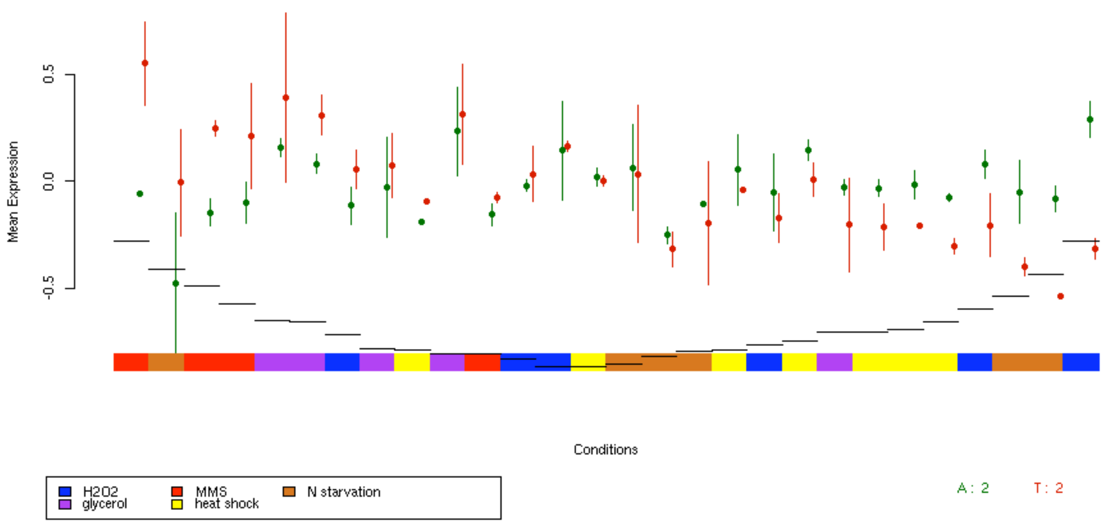

S. mikatae REB1 position 9

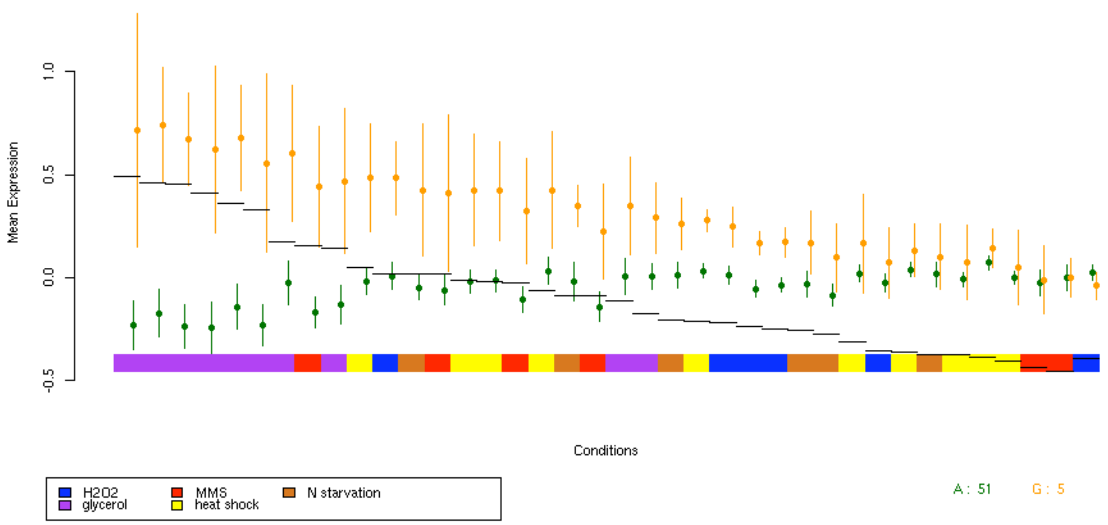

S. mikatae SPT15 position 2

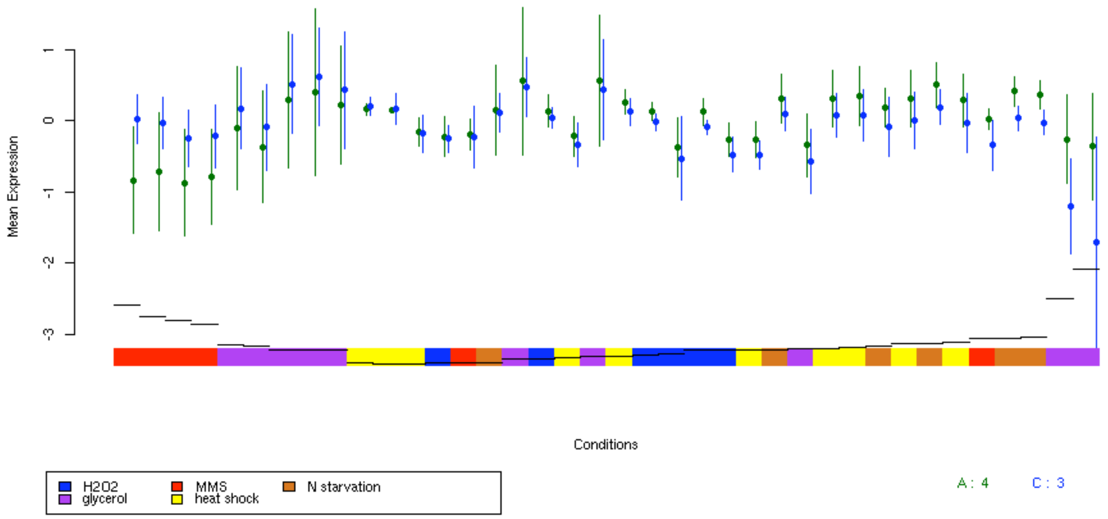

S. mikatae SPT15 position 2

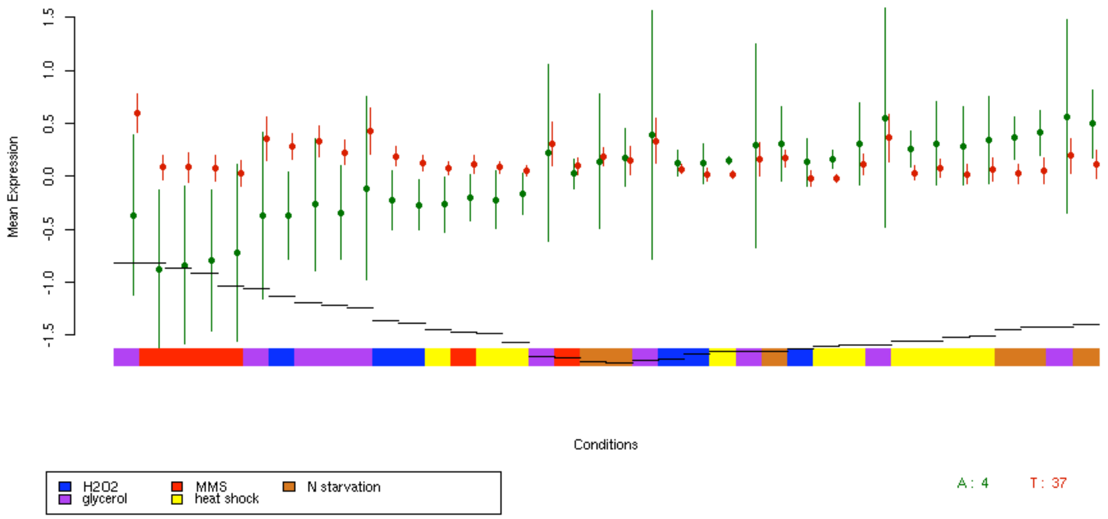

S. mikatae SPT15 position 2

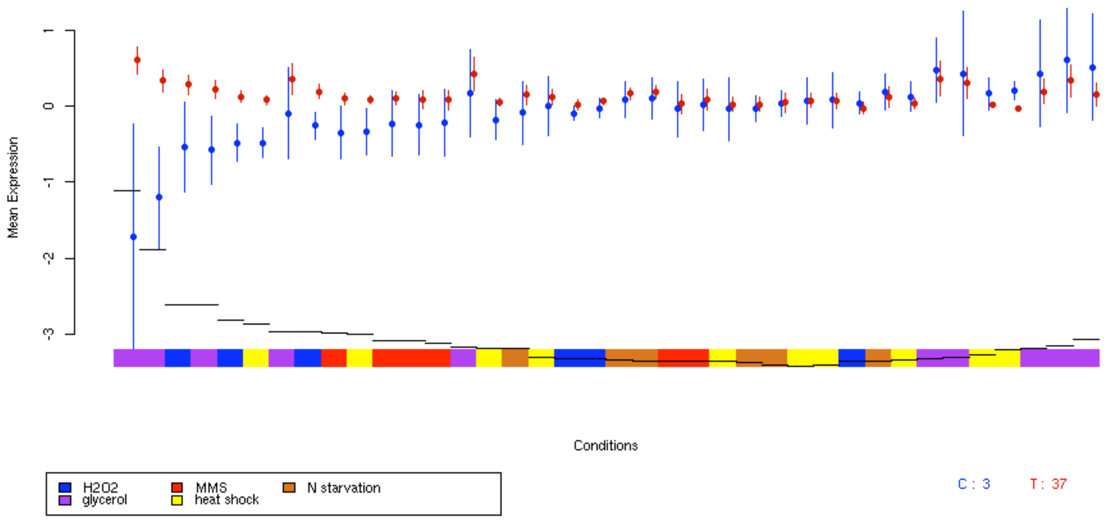

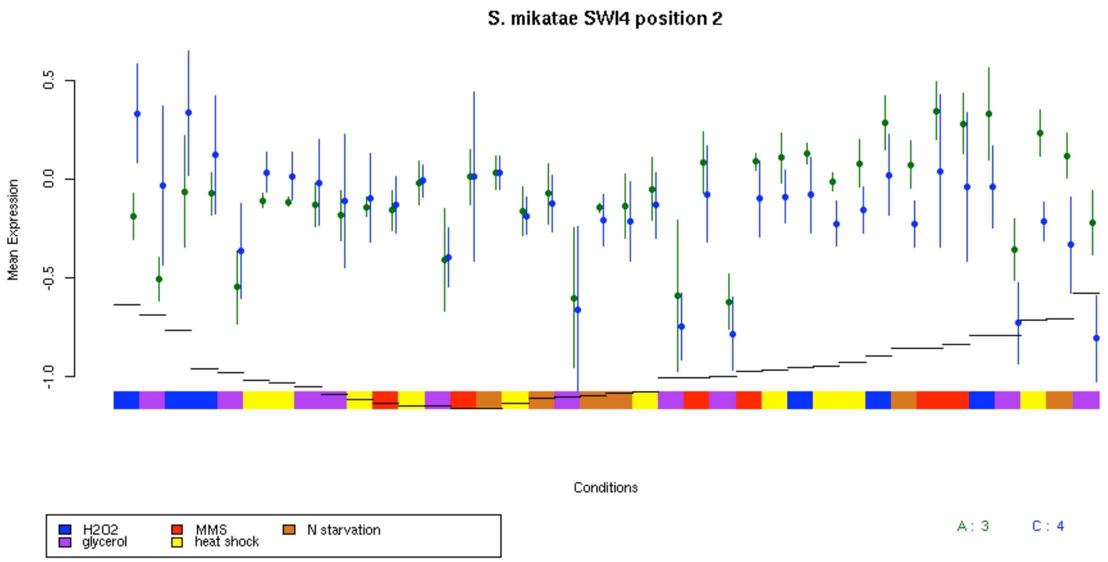

S. mikatae SWI4 position 2
Mean Expression
0.5
0.0
-0.5
-1.0
Conditions
H2O2
glycerol
MMS
heat shock
N starvation
A : 3
C : 4

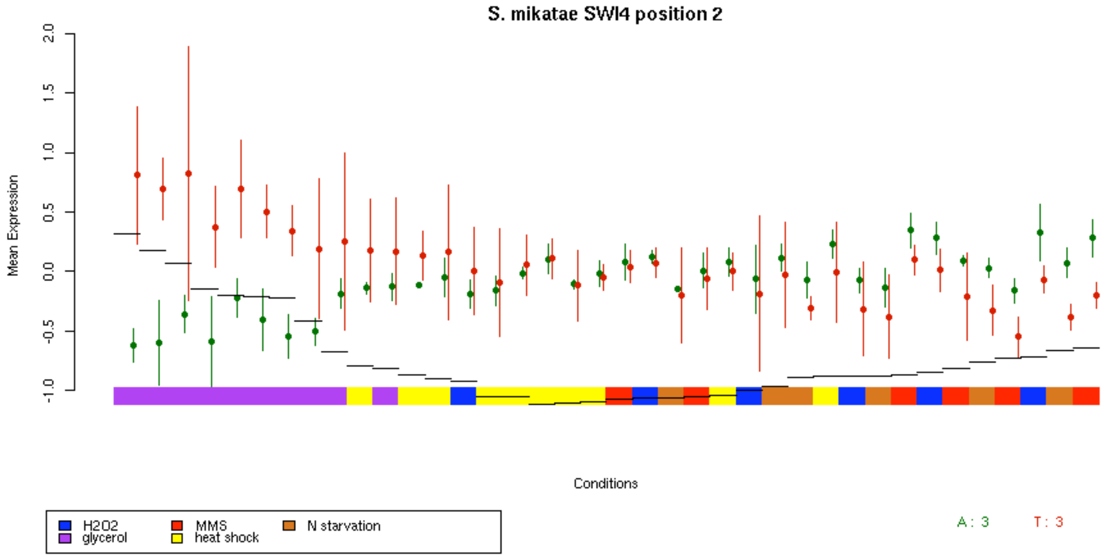

S. mikatae SWI4 position 2
Mean Expression
2.0
1.5
1.0
0.5
0.0
-0.5
-1.0
Conditions
H2O2
glycerol
MMS
heat shock
N starvation
A : 3
T : 3

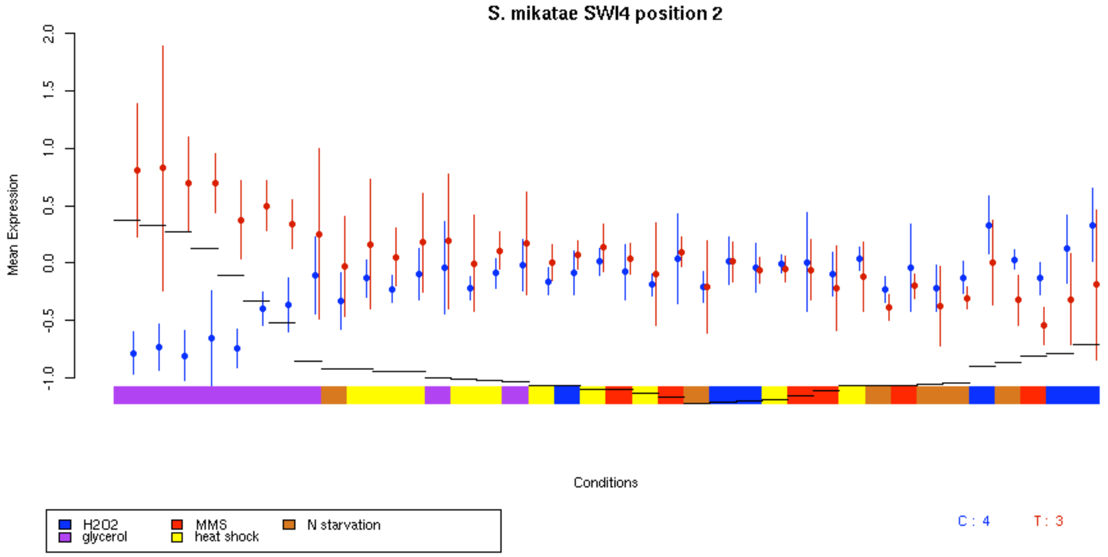
S. mikatae SWI4 position 2
Mean Expression
2.0
1.5
1.0
0.5
0.0
-0.5
-1.0
Conditions
H2O2
glycerol
MMS
heat shock
N starvation
C : 4
T : 3

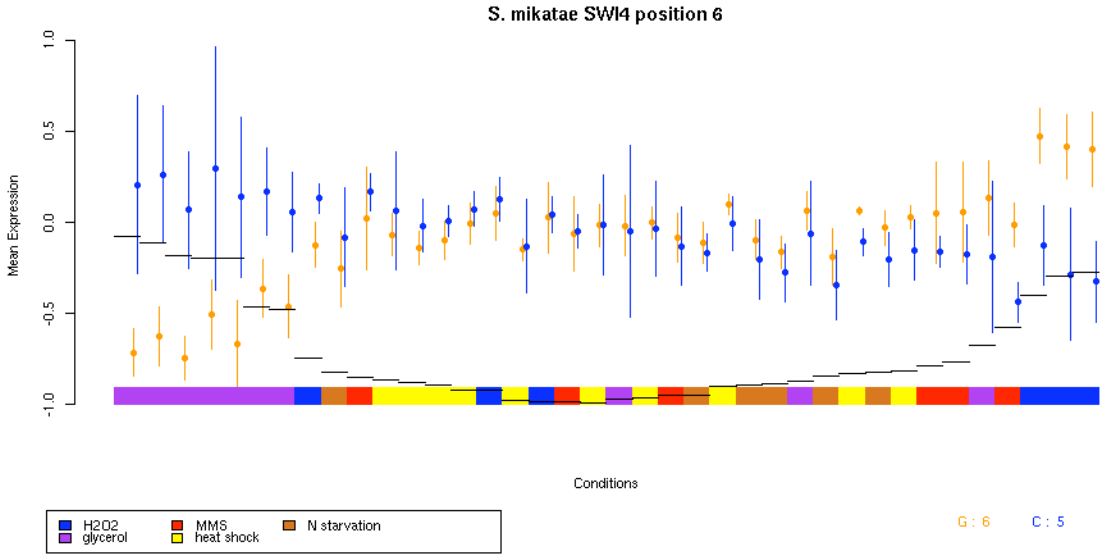
S. mikatae SWI4 position 6
Mean Expression
1.0
0.5
0.0
-0.5
-1.0
Conditions
H2O2
glycerol
MMS
heat shock
N starvation
G : 6
C : 5

S. paradoxus ABF1 position 15

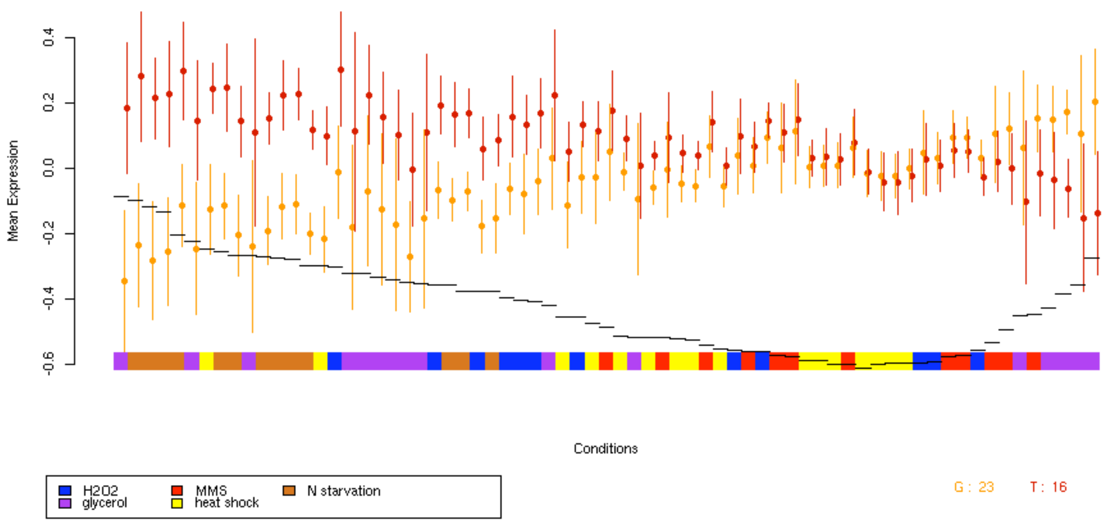

S. paradoxus ABF1 position 9

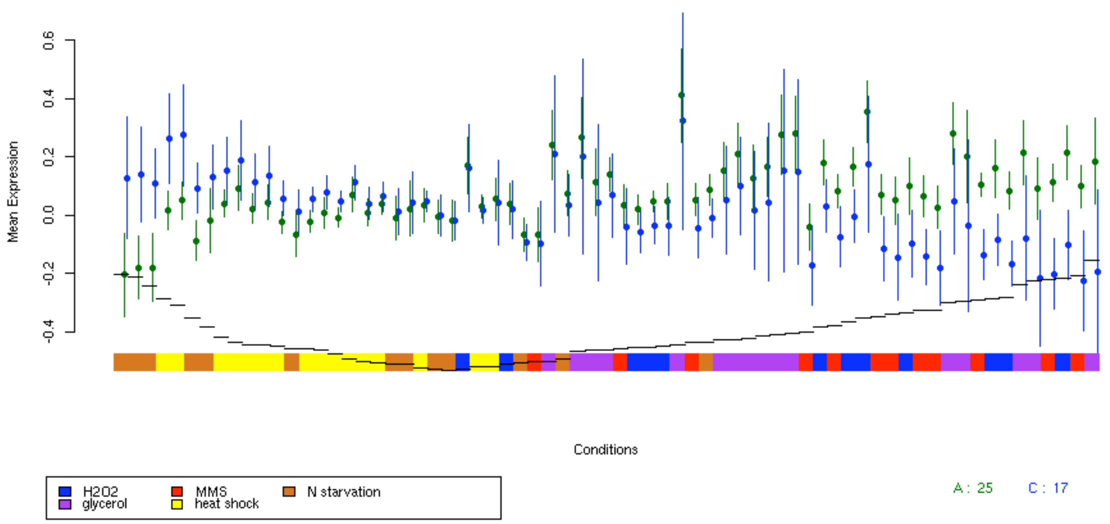

S. paradoxus ABF1 position 9

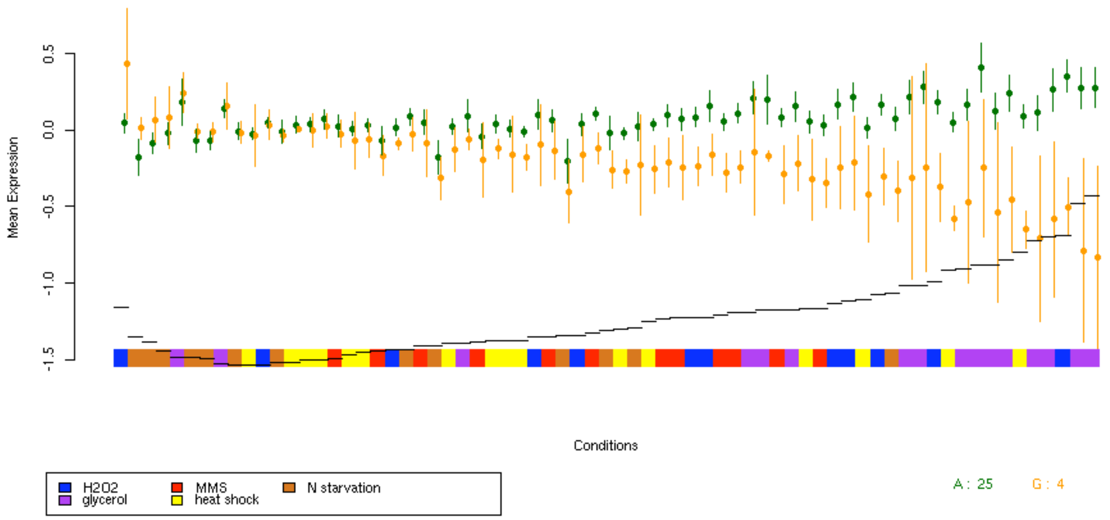

S. paradoxus ABF1 position 9

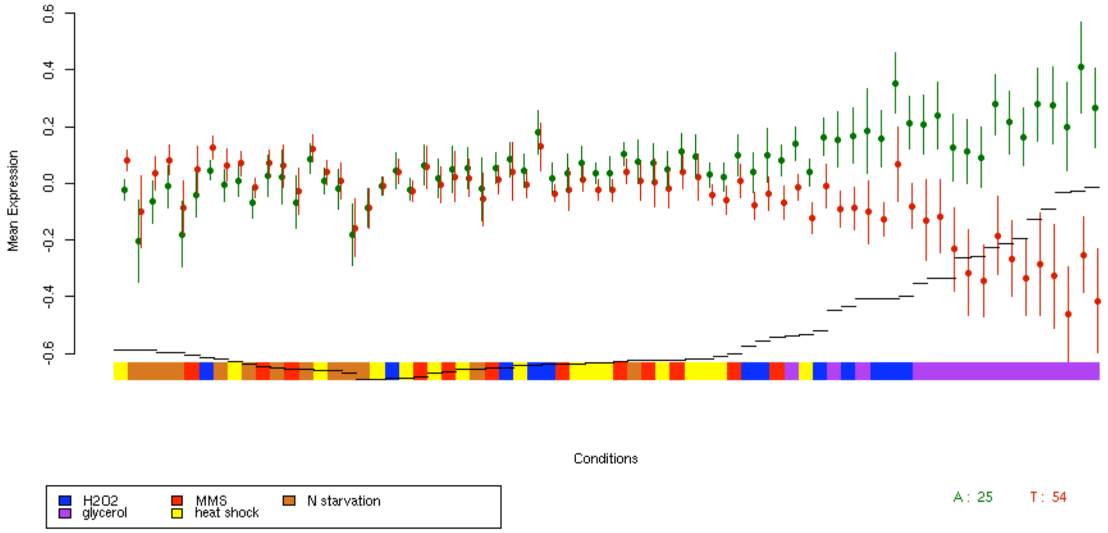

S. paradoxus ABF1 position 9

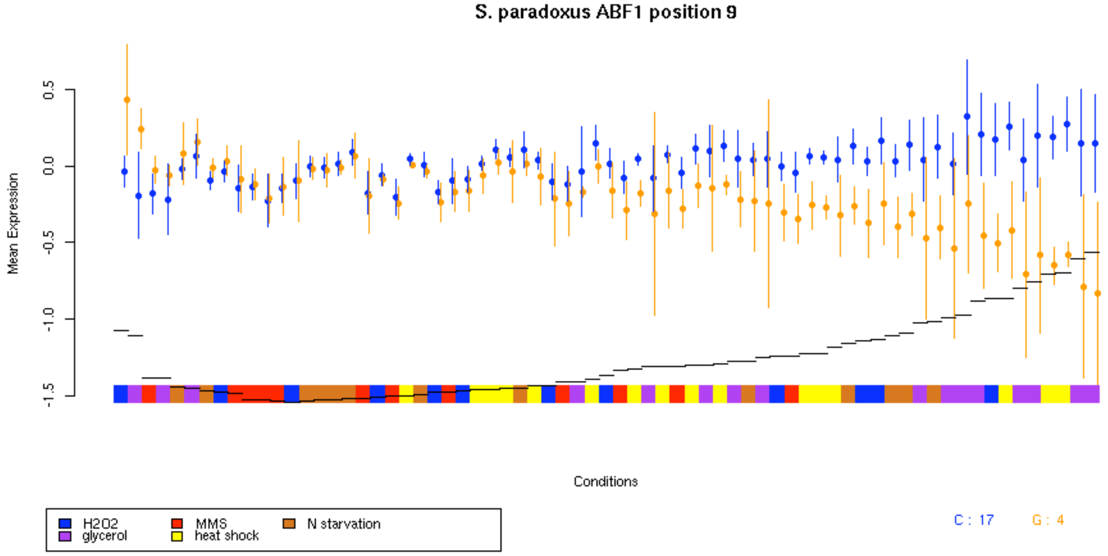

S. paradoxus ABF1 position 9

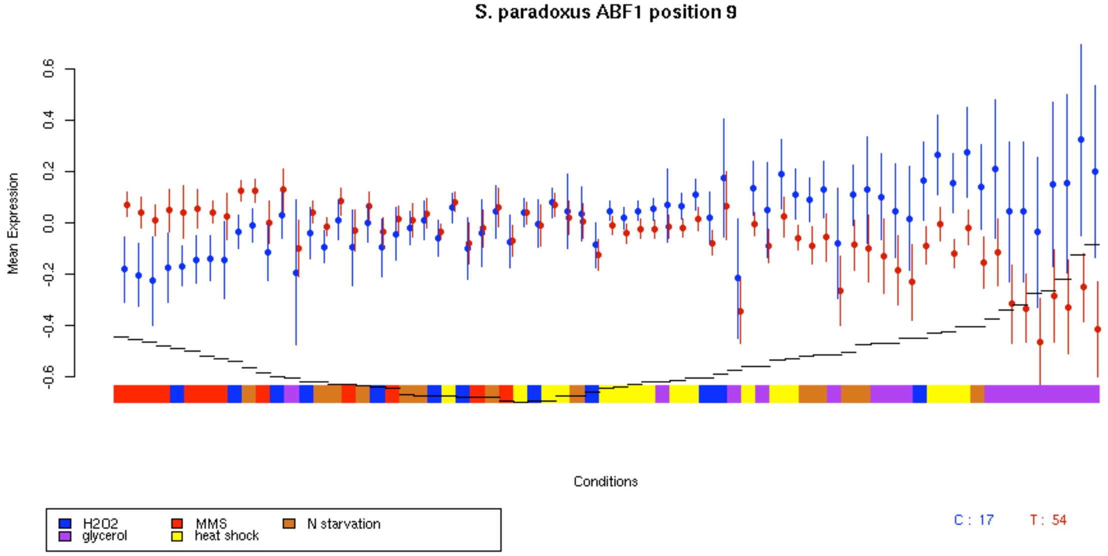

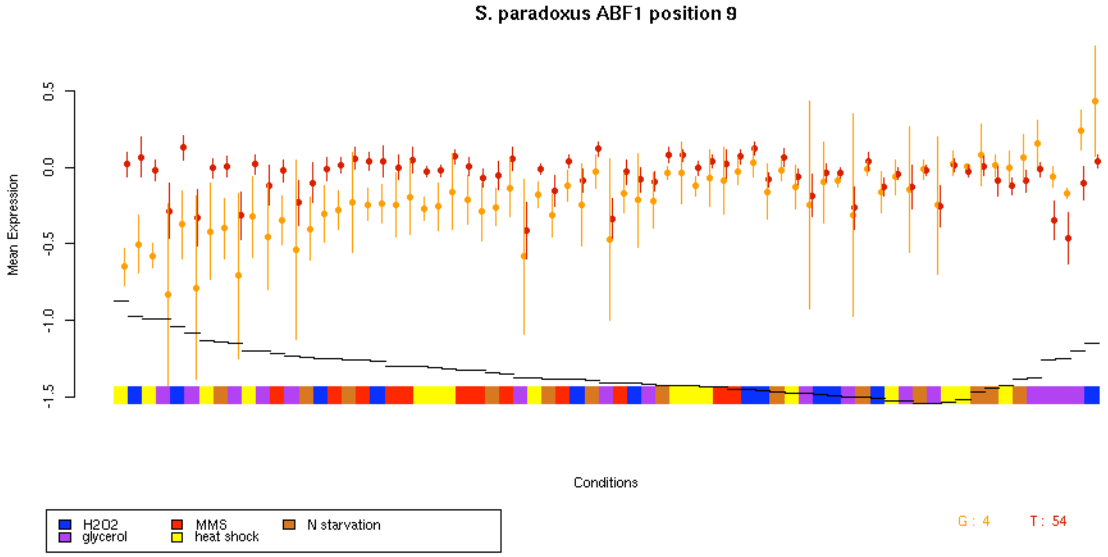
S. paradoxus ABF1 position 9
Mean Expression
0.5
0.0
-0.5
-1.0
-1.5
Conditions
H2O2
glycerol
MMS
heat shock
N starvation
G : 4
T : 54

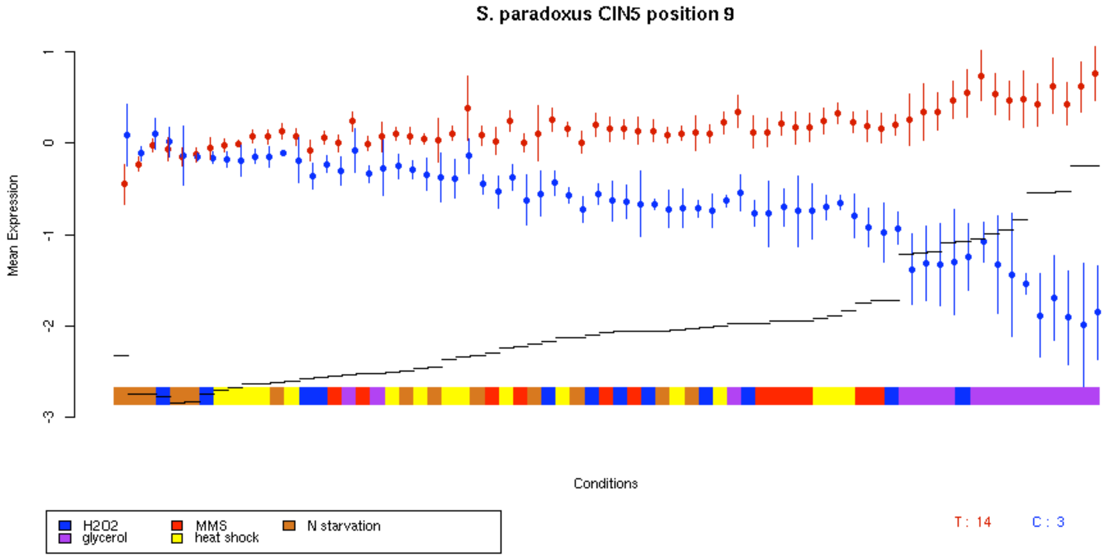
S. paradoxus CIN5 position 9
Mean Expression
1
0
-1
-2
-3
Conditions
H2O2
glycerol
MMS
heat shock
N starvation
T : 14
C : 3

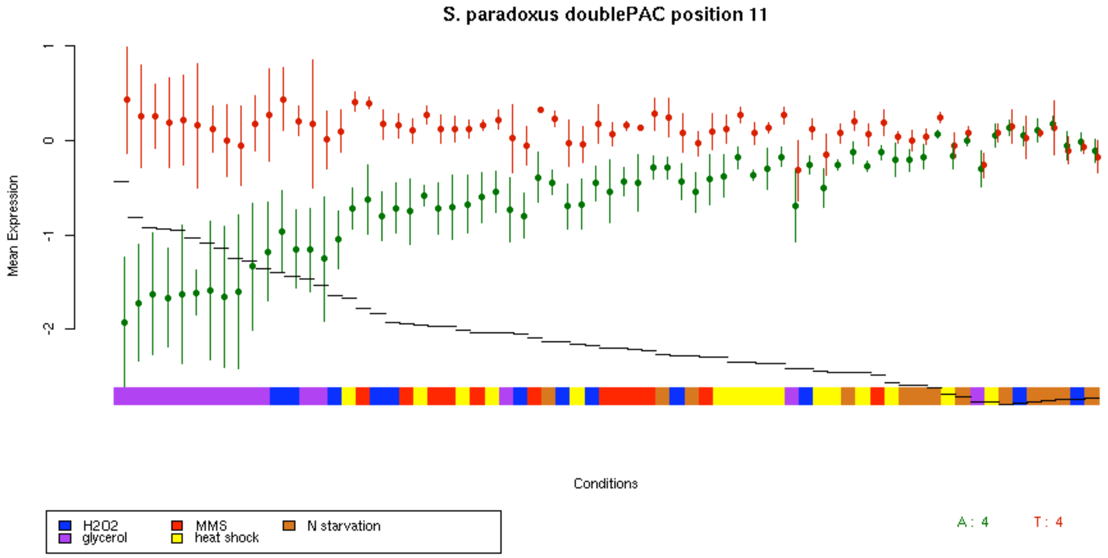
S. paradoxus doublePAC position 11
Mean Expression
1
0
-1
-2
Conditions
H2O2
glycerol
MMS
heat shock
N starvation
A : 4
T : 4

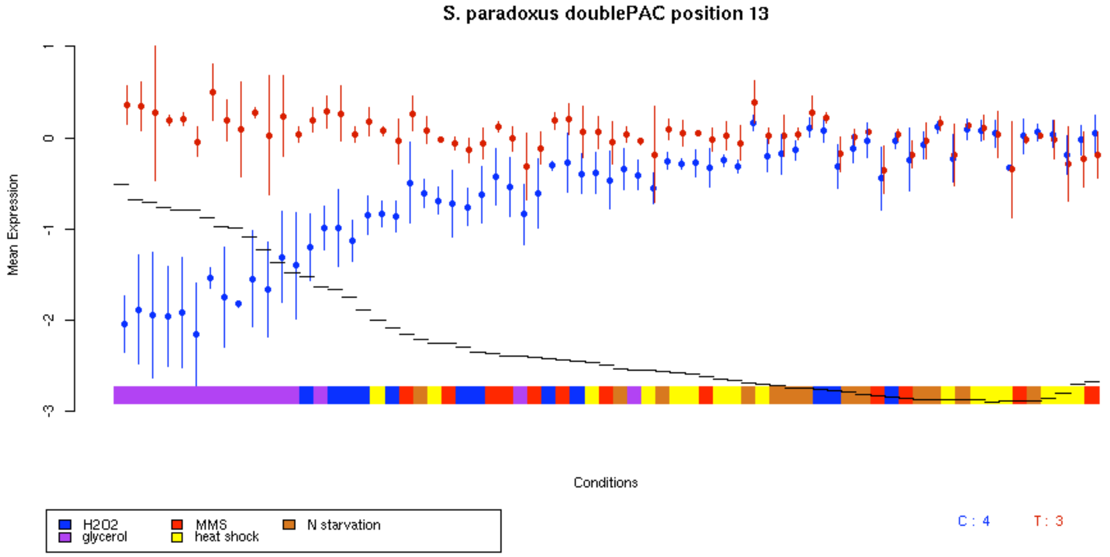
S. paradoxus doublePAC position 13
Mean Expression
1
0
-1
-2
-3
Conditions
H2O2
glycerol
MMS
heat shock
N starvation
C : 4
T : 3

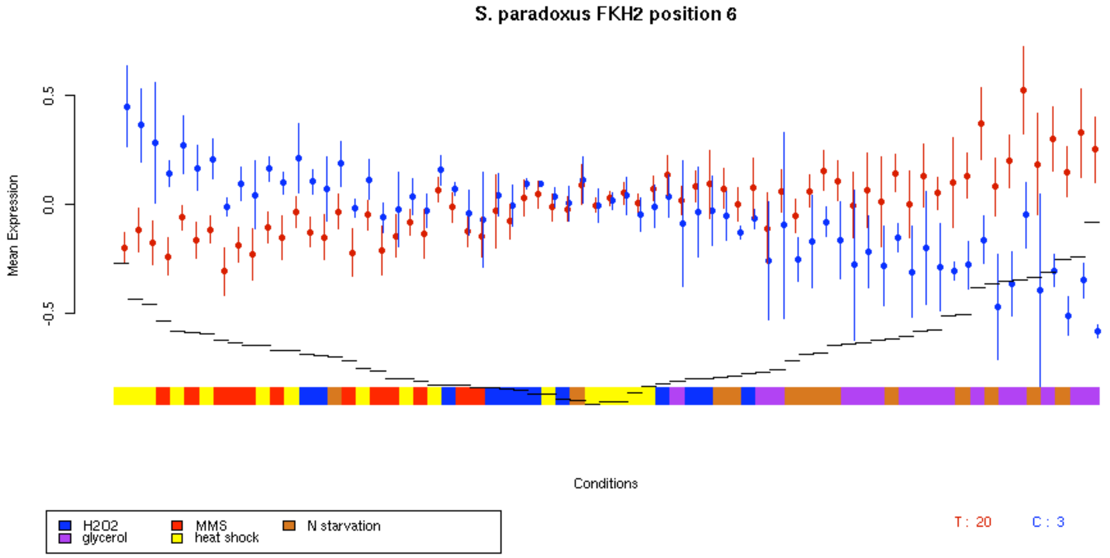
S. paradoxus FKH2 position 6
Mean Expression
0.5
0.0
-0.5
Conditions
H2O2
glycerol
MMS
heat shock
N starvation
T : 20
C : 3

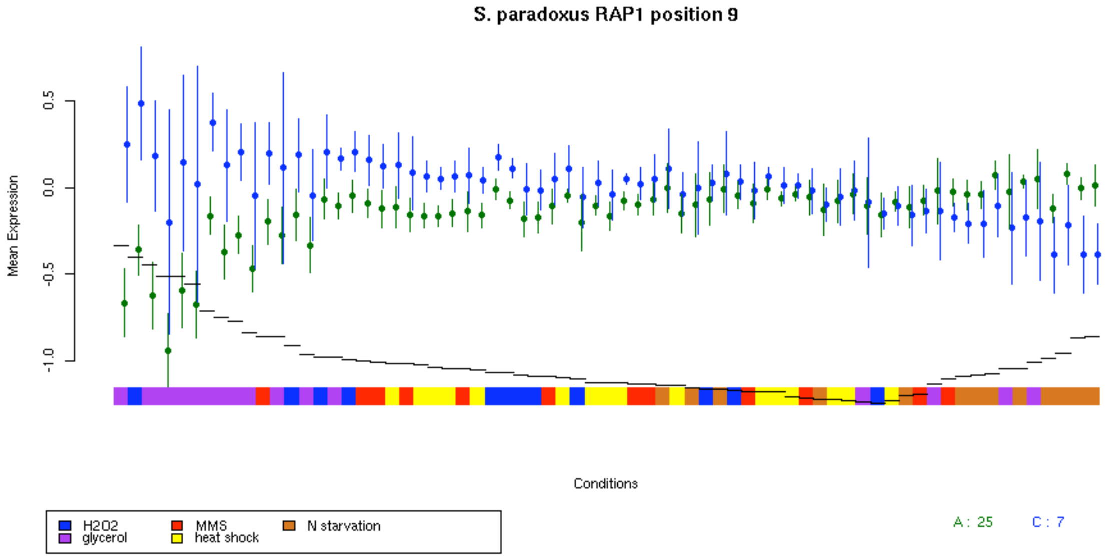
S. paradoxus RAP1 position 9
Mean Expression
0.5
0.0
-0.5
-1.0
Conditions
H2O2
glycerol
MMS
heat shock
N starvation
A : 25
C : 7

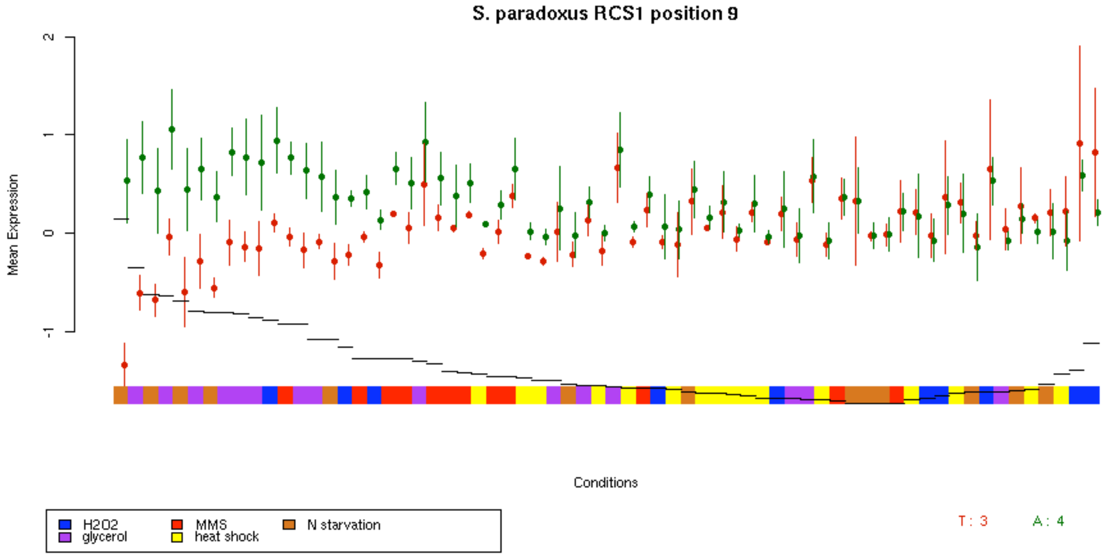
S. paradoxus RCS1 position 9
Mean Expression
Conditions
H2O2
glycerol
MMS
heat shock
N starvation
T : 3
A : 4

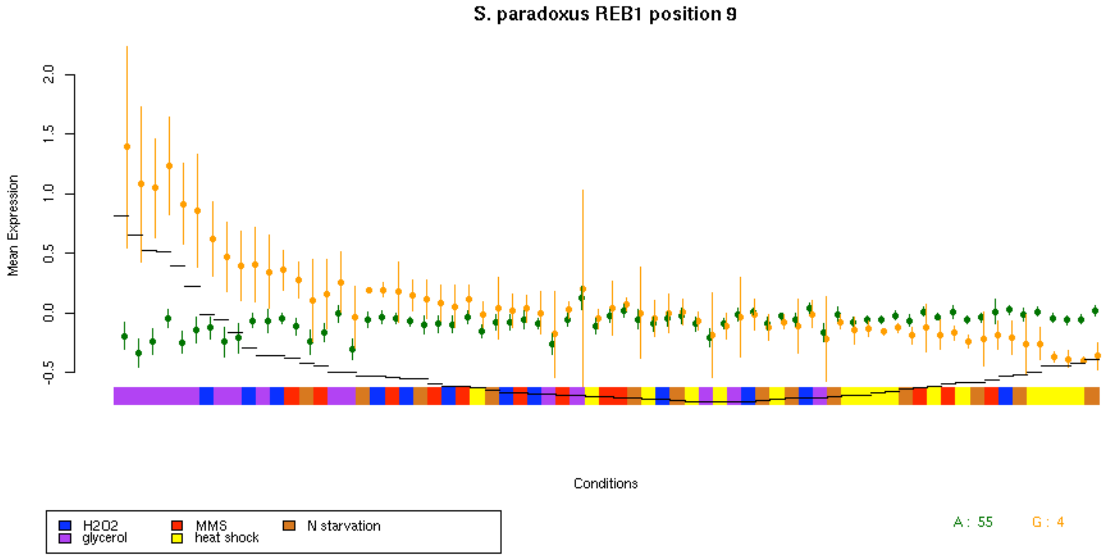
S. paradoxus REB1 position 9
Mean Expression
Conditions
H2O2
glycerol
MMS
heat shock
N starvation
A : 55
G : 4

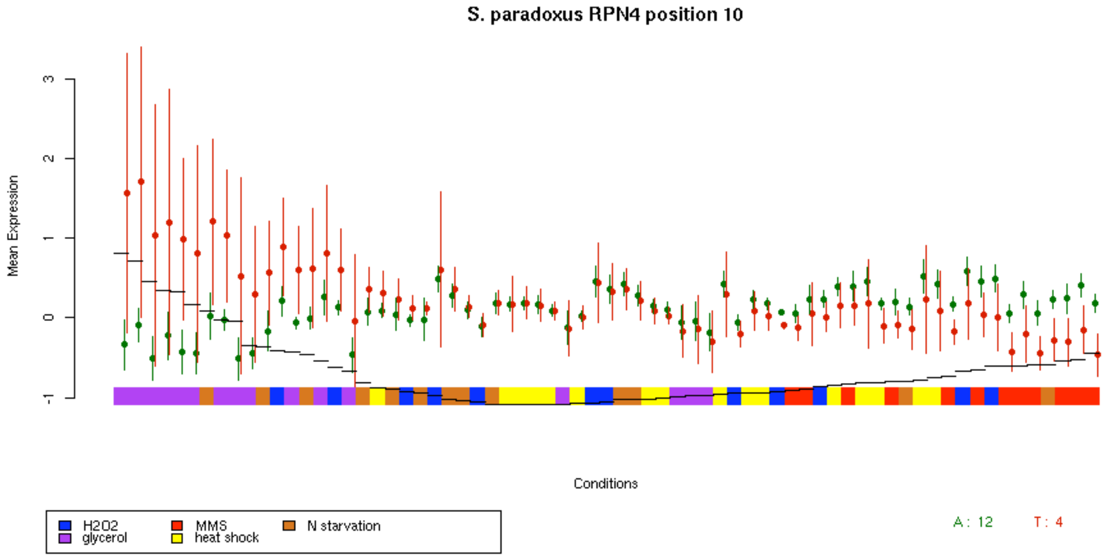
S. paradoxus RPN4 position 10
Mean Expression
Conditions
H2O2
glycerol
MMS
heat shock
N starvation
A : 12
T : 4

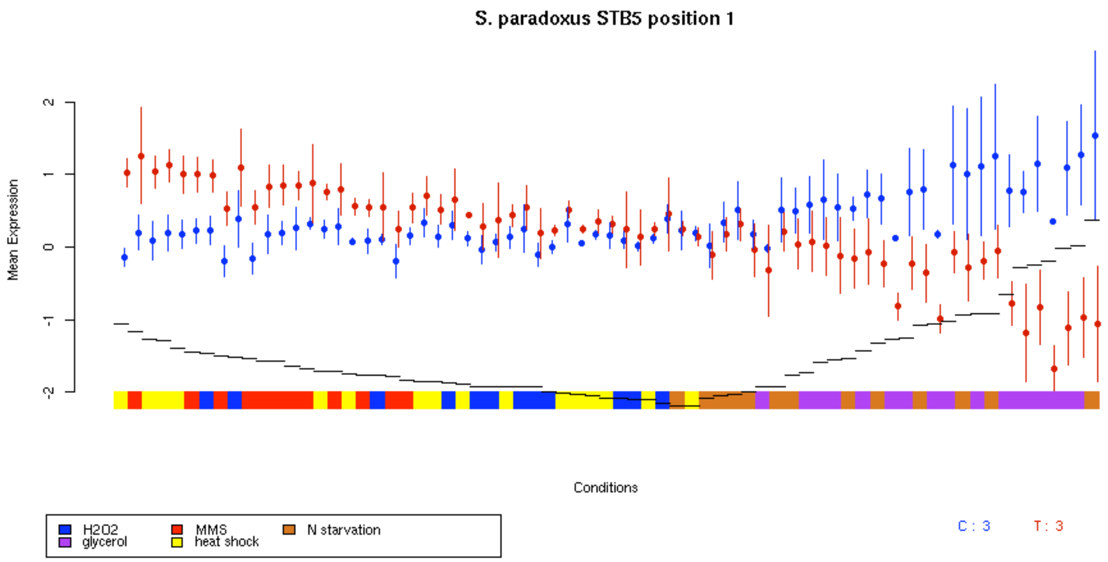
S. paradoxus STB5 position 1
Mean Expression
2
1
0
-1
-2
Conditions
H2O2
glycerol
MMS
heat shock
N starvation
C : 3
T : 3

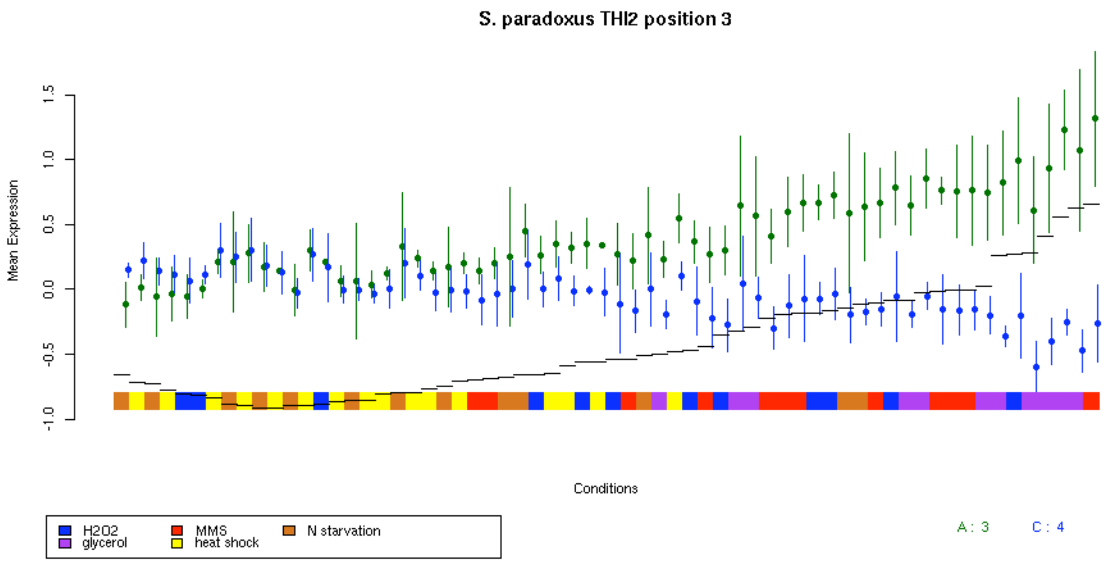
S. paradoxus THI2 position 3
Mean Expression
1.5
1.0
0.5
0.0
-0.5
-1.0
Conditions
H2O2
glycerol
MMS
heat shock
N starvation
A : 3
C : 4

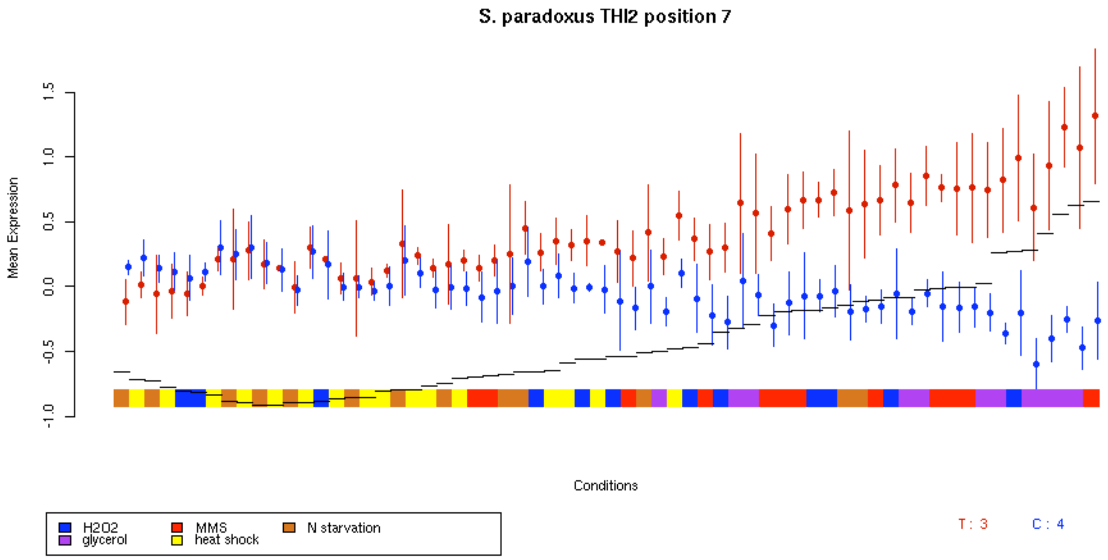
S. paradoxus THI2 position 7
Mean Expression
1.5
1.0
0.5
0.0
-0.5
-1.0
Conditions
H2O2
glycerol
MMS
heat shock
N starvation
T : 3
C : 4

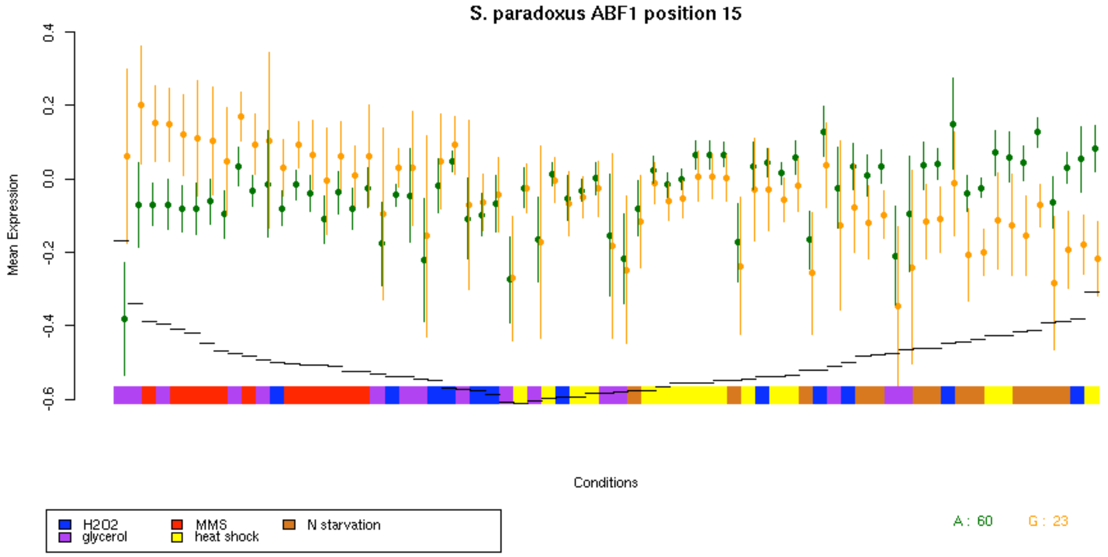

S. paradoxus ABF1 position 15
Mean Expression
Conditions
H2O2
glycerol
MMS
heat shock
N starvation
A : 60
G : 23

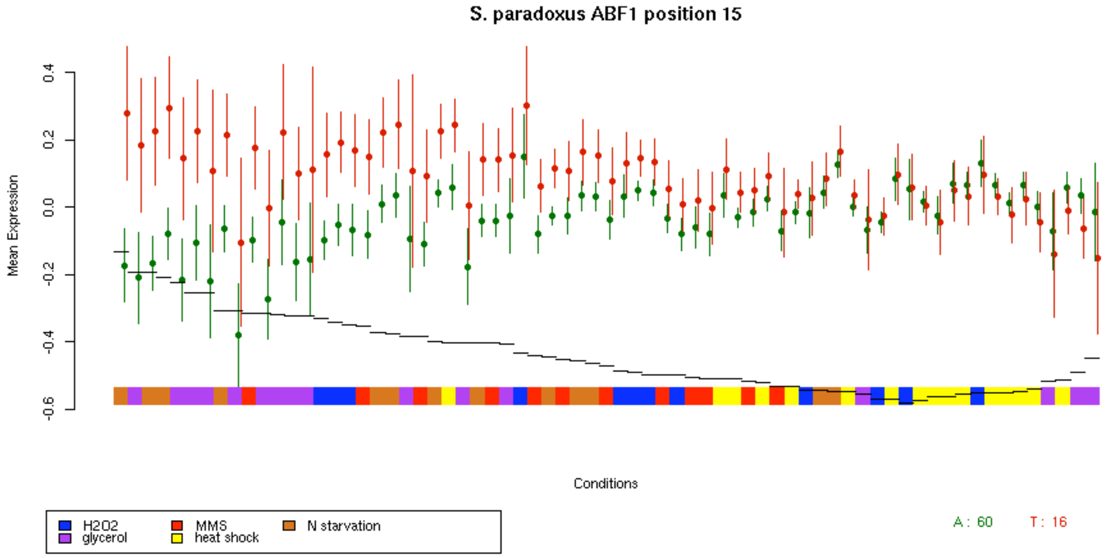

S. paradoxus ABF1 position 15
Mean Expression
Conditions
H2O2
glycerol
MMS
heat shock
N starvation
A : 60
T : 16
