# Supplementary material for: Contribution of Transcription Factor Binding Site Motif Variants to Condition-Specific Gene Expression Patterns in Budding Yeast
Source: PLoS One. 2012 Feb 23;7(2):e32274. doi: 10.1371/journal.pone.0032274 (PMC3285675; doi:10.1371/journal.pone.0032274)
Supplement: Table S1 — Transcription factor binding site motif positions that have functional variants inferred according to the variant distance of ranked experiments statistic (p<0.05). (PDF) [file pone.0032274.s006.pdf]

Table S1. Transcription factor binding motif positions identified with functionally variant (p<0.05) positions.

Transcription Factor Binding Site Family	Position	Score (*F*)	p-value	Information Content (bits)	Species	Platform
MCM1	4	122.526	0.032	0.15	*S. cerevisae*	Affymetrix S98
STE12DIG1	7	143.644	0.002	0.53	*S. cerevisae*	Affymetrix S98
REB1	2	27.4023	0.005	0.03	*S. cerevisae*	Affymetrix S98
YOX1	5	366.652	0.034	0.50	*S. cerevisae*	Affymetrix S98
SUM1	7	92.4822	0.002	0.99	*S. cerevisae*	Affymetrix S98
RGT1	2	158.442	0.011	1.60	*S. cerevisae*	Affymetrix S98
SUM1	8	89.3686	0.003	0.68	*S. cerevisae*	Affymetrix S98
RPN4	10	93.9509	0.018	0.30	*S. cerevisae*	Affymetrix S98
PAC	5	76.2545	0.022	0.96	*S. cerevisae*	Affymetrix S98
THI2	8	265.722	0.012	0.11	*S. cerevisae*	Affymetrix S98
THI2	10	330.062	0	0.96	*S. cerevisae*	Affymetrix S98
FKH2	1	44.191	0.031	0.96	*S. cerevisae*	Affymetrix S98
ROX1	9	142.987	0.008	1.46	*S. cerevisae*	Affymetrix S98
TEC1	4	233.906	0.044	0.98	*S. cerevisae*	Affymetrix S98
FKH2	6	79.4414	0.006	1.55	*S. cerevisae*	Affymetrix S98
ABF1	8	16.7334	0.034	0.23	*S. cerevisae*	Affymetrix S98
DOUBLEPAC	1	81.1273	0.031	1.11	*S. cerevisae*	Affymetrix S98
SPT15	2	2.57282	0.01	1.03	*S. cerevisae*	Y6.4kv6 cDNA
RAP1	7	9.00021	0.024	0.96	*S. cerevisae*	Y6.4kv6 cDNA
NRG1	2	41.9398	0	1.04	*S. cerevisae*	Y6.4kv6 cDNA
GCN4	1	10.6391	0.039	1.11	*S. cerevisae*	Y6.4kv6 cDNA
RAP1	10	8.11308	0.005	0.98	*S. cerevisae*	Y6.4kv6 cDNA
THI2	3	7.81477	0.045	0.96	*S. cerevisae*	Y6.4kv6 cDNA
STB5	1	28.6462	0.017	0.40	*S. cerevisae*	Y6.4kv6 cDNA
MATALPHA2	9	7.88728	0.027	1.31	*S. cerevisae*	Y6.4kv6 cDNA
RPN4	10	9.66342	0.026	0.39	*S. cerevisae*	Y6.4kv6 cDNA
HSF1	7	16.4034	0	0.98	*S. cerevisae*	Y6.4kv6 cDNA
PAC	5	11.3592	0.039	1.07	*S. cerevisae*	Y6.4kv6 cDNA
THI2	9	18.2617	0.02	0.66	*S. cerevisae*	Y6.4kv6 cDNA
REB1	9	3.12416	0.035	1.42	*S. cerevisae*	Y6.4kv6 cDNA
ABF1	15	2.89839	0.011	0.54	*S. paradoxus*	Y6.4kv6 cDNA
RAP1	9	15.9336	0.002	1.02	*S. paradoxus*	Y6.4kv6 cDNA
DOUBLEPAC	11	38.2778	0.023	0.96	*S. paradoxus*	Y6.4kv6 cDNA
DOUBLEPAC	13	81.0947	0.018	0.55	*S. paradoxus*	Y6.4kv6 cDNA
THI2	3	20.9998	0.027	0.97	*S. paradoxus*	Y6.4kv6 cDNA
STB5	1	85.3544	0	0.51	*S. paradoxus*	Y6.4kv6 cDNA
CIN5	9	39.8133	0	1.28	*S. paradoxus*	Y6.4kv6 cDNA
RPN4	10	17.2365	0.041	0.62	*S. paradoxus*	Y6.4kv6 cDNA
RCS1	9	20.3875	0	0.97	*S. paradoxus*	Y6.4kv6 cDNA
FKH2	6	15.6846	0.005	1.40	*S. paradoxus*	Y6.4kv6 cDNA
THI2	7	20.9998	0.023	0.97	*S. paradoxus*	Y6.4kv6 cDNA
REB1	9	7.2033	0.039	1.60	*S. paradoxus*	Y6.4kv6 cDNA
ABF1	9	4.01149	0.002	0.36	*S. paradoxus*	Y6.4kv6 cDNA
MCM1	6	17.6815	0	0.96	*S. mikatae*	Y6.4kv6 cDNA
RCS1	8	12.4192	0.035	0.037	*S. mikatae*	Y6.4kv6 cDNA
DOUBLEPAC	11	56.4898	0	1.09	*S. mikatae*	Y6.4kv6 cDNA
YOX1	13	4.89925	0	0.96	*S. mikatae*	Y6.4kv6 cDNA
PAC	6	20.4907	0.037	1.54	*S. mikatae*	Y6.4kv6 cDNA
SWI4	2	11.2765	0.028	0.09	*S. mikatae*	Y6.4kv6 cDNA
REB1	9	2.42043	0.037	1.52	*S. mikatae*	Y6.4kv6 cDNA
SPT15	2	2.89672	0.028	1.17	*S. mikatae*	Y6.4kv6 cDNA
SWI4	6	12.8533	0.019	0.96	*S. mikatae*	Y6.4kv6 cDNA

Transcription Factor Binding Site Family	Position	Score (*F*)	p-value	Information Content (bits)	Species	Platform
ABF1	6	1.28983	0.034	0.97	*S. mikatae*	Y6.4kv6 cDNA
MBP1	1	4.9658	0.035	1.06	*S. mikatae*	Y6.4kv6 cDNA
RAP1	10	4.89422	0.007	1.02	*S. mikatae*	Y6.4kv6 cDNA
DOUBLEPAC	3	36.5188	0	1.14	*S. kudriavzevii*	Y6.4kv6 cDNA
PAC	12	4.90279	0.031	0.29	*S. kudriavzevii*	Y6.4kv6 cDNA
RAP1	10	5.51761	0.031	0.86	*S. kudriavzevii*	Y6.4kv6 cDNA
DOUBLEPAC	7	36.5188	0	1.14	*S. kudriavzevii*	Y6.4kv6 cDNA
HSF1	6	58.0168	0.015	0.04	*S. kudriavzevii*	Y6.4kv6 cDNA
STB5	6	10.9097	0	0.96	*S. kudriavzevii*	Y6.4kv6 cDNA
SPT15	1	10.3586	0	0.20	*S. kudriavzevii*	Y6.4kv6 cDNA
FKH2	9	8.12117	0.012	1.61	*S. kudriavzevii*	Y6.4kv6 cDNA
DOUBLEPAC	11	16.7642	0.047	1.00	*S. kudriavzevii*	Y6.4kv6 cDNA
MAC1	8	22.245	0	0.96	*S. kudriavzevii*	Y6.4kv6 cDNA
FKH2	11	6.79457	0.033	1.61	*S. kudriavzevii*	Y6.4kv6 cDNA
CIN5	9	5.86465	0.043	0.82	*S. kudriavzevii*	Y6.4kv6 cDNA
RPN4	2	14.9538	0.029	1.45	*S. kudriavzevii*	Y6.4kv6 cDNA
MCM1	8	16.162	0.015	1.00	*S. kudriavzevii*	Y6.4kv6 cDNA
MATALPHA2	2	7.19348	0.042	0.77	*S. kudriavzevii*	Y6.4kv6 cDNA
ABF1	6	1.27823	0.035	1.16	*S. kudriavzevii*	Y6.4kv6 cDNA
