# Supplementary material for: Contribution of Transcription Factor Binding Site Motif Variants to Condition-Specific Gene Expression Patterns in Budding Yeast
Source: PLoS One. 2012 Feb 23;7(2):e32274. doi: 10.1371/journal.pone.0032274 (PMC3285675; doi:10.1371/journal.pone.0032274)
Supplement: Table S2 — Transcription factor binding site motif positions that have functional variants inferred according to the variant distance of ranked experiments statistic (p<0.05) in both target genes with single primary inputs and multiple primary inputs. (PDF) [file pone.0032274.s007.pdf]

Table S2. Transcription factor binding motif positions identified with functionally variant ($p<0.05$) positions in both targets with single primary inputs and multiple primary inputs (for Affymetrix data only).

Tf	position	Score (F)	pvalue
SUM1	8	37.7401	0
SUM1	7	25.6358	0.002
ABF1	8	7.54051	0.023
