# Supplementary material for: Contribution of Transcription Factor Binding Site Motif Variants to Condition-Specific Gene Expression Patterns in Budding Yeast
Source: PLoS One. 2012 Feb 23;7(2):e32274. doi: 10.1371/journal.pone.0032274 (PMC3285675; doi:10.1371/journal.pone.0032274)
Supplement: Table S3 — Condition specificity of functional binding site motif variants (BSMVs). Significance of segregation of experimental conditions dependent upon upregulation of the major base or minor base. Cases are shown where the position has a functional BSMV and where there is clustering of similar experimental conditions when the experiments are sorted according to the difference in regulation of each-BSMV's target set. (PDF) [file pone.0032274.s008.pdf]

Table S3. Condition specificity of functional TF binding site variants. Significance of segregation of experimental conditions dependent upon upregulation of the major base or minor base. Cases are shown where the position has a functional heterogeneity and where there is clustering of similar experimental conditions when the experiments are sorted according to the difference in regulation of each-variant's target set.

Transcription Factor Binding Site Family	Position	p-value of condition specificity	Variant 1	Variant 2	Platform	Species
GCN4	1	0.0018	G	T	Y6.4kv6 cDNA	*S. cerevisae*
HSF1	7	0	G	T	Y6.4kv6 cDNA	*S. cerevisae*
MATalpha2	9	0.0001	G	A	Y6.4kv6 cDNA	*S. cerevisae*
NRG1	2	0	G	A	Y6.4kv6 cDNA	*S. cerevisae*
PAC	5	0.003	A	C	Y6.4kv6 cDNA	*S. cerevisae*
RAP1	7	0.0002	A	G	Y6.4kv6 cDNA	*S. cerevisae*
RAP1	7	0	A	T	Y6.4kv6 cDNA	*S. cerevisae*
RAP1	10	0	T	C	Y6.4kv6 cDNA	*S. cerevisae*
REB1	9	0	A	G	Y6.4kv6 cDNA	*S. cerevisae*
RPN4	10	0	A	C	Y6.4kv6 cDNA	*S. cerevisae*
RPN4	10	0	A	T	Y6.4kv6 cDNA	*S. cerevisae*
RPN4	10	0	C	T	Y6.4kv6 cDNA	*S. cerevisae*
SPT15	2	0.0087	A	C	Y6.4kv6 cDNA	*S. cerevisae*
SPT15	2	0	A	T	Y6.4kv6 cDNA	*S. cerevisae*
STB5	1	0	A	T	Y6.4kv6 cDNA	*S. cerevisae*
THI2	3	0	A	C	Y6.4kv6 cDNA	*S. cerevisae*
THI2	9	0	A	T	Y6.4kv6 cDNA	*S. cerevisae*
ABF1	8	0	A	G	Affymetrix S98	*S. cerevisae*
ABF1	8	0	A	C	Affymetrix S98	*S. cerevisae*
ABF1	8	0	A	T	Affymetrix S98	*S. cerevisae*
ABF1	8	0	C	G	Affymetrix S98	*S. cerevisae*
ABF1	8	0	C	T	Affymetrix S98	*S. cerevisae*
ABF1	8	0	G	T	Affymetrix S98	*S. cerevisae*
doublePAC	1	0	T	A	Affymetrix S98	*S. cerevisae*
FKH2	1	0	A	T	Affymetrix S98	*S. cerevisae*
FKH2	1	0	C	G	Affymetrix S98	*S. cerevisae*
FKH2	1	0	A	C	Affymetrix S98	*S. cerevisae*
FKH2	1	0	C	T	Affymetrix S98	*S. cerevisae*
FKH2	1	0	A	G	Affymetrix S98	*S. cerevisae*
FKH2	1	0	G	T	Affymetrix S98	*S. cerevisae*
FKH2	6	0	T	C	Affymetrix S98	*S. cerevisae*
MCM1	4	0	A	C	Affymetrix S98	*S. cerevisae*
MCM1	4	0	A	T	Affymetrix S98	*S. cerevisae*
MCM1	4	0	C	T	Affymetrix S98	*S. cerevisae*
PAC	5	0	C	T	Affymetrix S98	*S. cerevisae*
PAC	5	0	A	C	Affymetrix S98	*S. cerevisae*
PAC	5	0	A	T	Affymetrix S98	*S. cerevisae*
REB1	2	0	C	T	Affymetrix S98	*S. cerevisae*
REB1	2	0	C	G	Affymetrix S98	*S. cerevisae*
REB1	2	0	A	T	Affymetrix S98	*S. cerevisae*
REB1	2	0	A	G	Affymetrix S98	*S. cerevisae*
REB1	2	0	A	C	Affymetrix S98	*S. cerevisae*

Transcription Factor Binding Site Family	Position	p-value of condition specificity	Variant 1	Variant 2	Platform	Species
REB1	2	0	G	T	Affymetrix S98	*S. cerevisae*
RGT1	2	0	T	C	Affymetrix S98	*S. cerevisae*
ROX1	9	0	T	C	Affymetrix S98	*S. cerevisae*
RPN4	10	0	A	G	Affymetrix S98	*S. cerevisae*
RPN4	10	0	C	T	Affymetrix S98	*S. cerevisae*
RPN4	10	0	A	C	Affymetrix S98	*S. cerevisae*
RPN4	10	0	C	G	Affymetrix S98	*S. cerevisae*
RPN4	10	0	G	T	Affymetrix S98	*S. cerevisae*
RPN4	10	0	A	T	Affymetrix S98	*S. cerevisae*
STE12DIG1	7	0	G	T	Affymetrix S98	*S. cerevisae*
STE12DIG1	7	0	A	T	Affymetrix S98	*S. cerevisae*
STE12DIG1	7	0	A	G	Affymetrix S98	*S. cerevisae*
SUM1	7	0	G	C	Affymetrix S98	*S. cerevisae*
SUM1	8	0	C	T	Affymetrix S98	*S. cerevisae*
SUM1	8	0	A	C	Affymetrix S98	*S. cerevisae*
SUM1	8	0	A	T	Affymetrix S98	*S. cerevisae*
TEC1	4	0	A	G	Affymetrix S98	*S. cerevisae*
THI2	8	0	G	T	Affymetrix S98	*S. cerevisae*
THI2	8	0	C	T	Affymetrix S98	*S. cerevisae*
THI2	8	0	C	G	Affymetrix S98	*S. cerevisae*
THI2	10	0	T	A	Affymetrix S98	*S. cerevisae*
YOX1	5	0	C	G	Affymetrix S98	*S. cerevisae*
ABF1	6	0.0002	C	T	Y6.4kv6 cDNA	*S. kudriavzevii*
CIN5	9	0.0127	C	T	Y6.4kv6 cDNA	*S. kudriavzevii*
doublePAC	3	0.0018	C	T	Y6.4kv6 cDNA	*S. kudriavzevii*
doublePAC	7	0.0018	T	C	Y6.4kv6 cDNA	*S. kudriavzevii*
doublePAC	11	0.0171	A	T	Y6.4kv6 cDNA	*S. kudriavzevii*
FKH2	9	0.0026	A	G	Y6.4kv6 cDNA	*S. kudriavzevii*
HSF1	6	0	A	G	Y6.4kv6 cDNA	*S. kudriavzevii*
MCM1	8	0	G	A	Y6.4kv6 cDNA	*S. kudriavzevii*
PAC	12	0	C	G	Y6.4kv6 cDNA	*S. kudriavzevii*
PAC	12	0.0006	A	G	Y6.4kv6 cDNA	*S. kudriavzevii*
PAC	12	0.0151	A	C	Y6.4kv6 cDNA	*S. kudriavzevii*
RAP1	10	0	C	T	Y6.4kv6 cDNA	*S. kudriavzevii*
RPN4	2	0.0012	G	A	Y6.4kv6 cDNA	*S. kudriavzevii*
SPT15	1	0.0011	C	T	Y6.4kv6 cDNA	*S. kudriavzevii*
SPT15	1	0	A	C	Y6.4kv6 cDNA	*S. kudriavzevii*
SPT15	1	0	A	T	Y6.4kv6 cDNA	*S. kudriavzevii*
doublePAC	11	0	A	T	Y6.4kv6 cDNA	*S. mikatae*
MBP1	1	0.0222	A	T	Y6.4kv6 cDNA	*S. mikatae*
MCM1	6	0.0001	T	G	Y6.4kv6 cDNA	*S. mikatae*
PAC	6	0.0017	A	G	Y6.4kv6 cDNA	*S. mikatae*
RAP1	10	0.0006	T	C	Y6.4kv6 cDNA	*S. mikatae*
RCS1	8	0.031	A	T	Y6.4kv6 cDNA	*S. mikatae*
REB1	9	0.0041	A	G	Y6.4kv6 cDNA	*S. mikatae*
SPT15	2	0.0199	C	T	Y6.4kv6 cDNA	*S. mikatae*
SPT15	2	0.0076	A	C	Y6.4kv6 cDNA	*S. mikatae*
SWI4	2	0	A	T	Y6.4kv6 cDNA	*S. mikatae*
SWI4	2	0.0001	C	T	Y6.4kv6 cDNA	*S. mikatae*
SWI4	6	0.0096	G	C	Y6.4kv6 cDNA	*S. mikatae*

Transcription Factor Binding Site Family	Position	p-value of condition specificity	Variant 1	Variant 2	Platform	Species
ABF1	9	0.0338	C	G	Y6.4kv6 cDNA	*S. paradoxus*
ABF1	9	0.0018	C	T	Y6.4kv6 cDNA	*S. paradoxus*
ABF1	9	0	A	C	Y6.4kv6 cDNA	*S. paradoxus*
ABF1	15	0.0017	A	G	Y6.4kv6 cDNA	*S. paradoxus*
ABF1	15	0.0035	G	T	Y6.4kv6 cDNA	*S. paradoxus*
doublePAC	11	0	A	T	Y6.4kv6 cDNA	*S. paradoxus*
doublePAC	13	0	C	T	Y6.4kv6 cDNA	*S. paradoxus*
RAP1	9	0	A	C	Y6.4kv6 cDNA	*S. paradoxus*
REB1	9	0	A	G	Y6.4kv6 cDNA	*S. paradoxus*
RPN4	10	0	A	T	Y6.4kv6 cDNA	*S. paradoxus*
STB5	1	0	C	T	Y6.4kv6 cDNA	*S. paradoxus*
