# Supplementary material for: Contribution of Transcription Factor Binding Site Motif Variants to Condition-Specific Gene Expression Patterns in Budding Yeast
Source: PLoS One. 2012 Feb 23;7(2):e32274. doi: 10.1371/journal.pone.0032274 (PMC3285675; doi:10.1371/journal.pone.0032274)
Supplement: Table S4 — Gene ontology enrichment among genes with alternative binding site motif variants at functionally variant binding site motif positions. (PDF) [file pone.0032274.s009.pdf]

Table S4. Gene ontology enrichment among genes with alternative variants at functionally variant transcription factor binding site positions.

Transcription Factor Binding Site Family	Position	Variant	Gene Ontology Process	p-value of enrichment
PAC	5	C	RNA metabolism	0
RPN4	10	A	protein catabolism	0
SUM1	7	C	sporulation	1E-5
STE12DIG1	7	A	morphogenesis	0.00013
THI2	10	T	vitamin metabolism	0.00023
DOUBLEPAC	1	A	ribosome biogenesis and assembly	0.00037
SUM1	8	A	sporulation	0.00053
FKH2	1	T	DNA metabolism	0.00155
STE12DIG1	7	G	vesicle-mediated transport	0.00282
REB1	2	G	cell budding	0.00307
ABF1	8	T	ribosome biogenesis and assembly	0.00388
MCM1	4	A	protein modification	0.00444
MCM1	4	C	cell wall organization and biogenesis	0.00445
FKH2	6	T	DNA metabolism	0.00744
SUM1	7	G	protein biosynthesis	0.00831
MCM1	4	G	generation of precursor metabolites and energy	0.0087
THI2	8	C	vitamin metabolism	0.01041
RPN4	10	T	protein catabolism	0.01452
THI2	8	T	vitamin metabolism	0.01536
TEC1	4	G	morphogenesis	0.01574
MCM1	4	T	DNA metabolism	0.01644
PAC	5	C	ribosome biogenesis and assembly	0.01646
PAC	5	A	transcription	0.01805
STE12DIG1	7	G	conjugation	0.02015
THI2	8	G	vitamin metabolism	0.02092
REB1	2	A	vesicle-mediated transport	0.02153
RPN4	10	T	biological_process unknown	0.0219
ABF1	8	T	pseudohyphal growth	0.02242
THI2	8	A	organelle organization and biogenesis	0.02318
THI2	10	A	vitamin metabolism	0.02538
RGT1	2	T	response to stress	0.03126
RPN4	10	G	cytoskeleton organization and biogenesis	0.03235
RGT1	2	C	carbohydrate metabolism	0.03692
THI2	8	G	cell homeostasis	0.04004
MCM1	4	T	pseudohyphal growth	0.04057
SUM1	8	T	protein biosynthesis	0.04081
FKH2	6	T	protein biosynthesis	0.0479
THI2	10	A	cell homeostasis	0.04923
ABF1	8	T	organelle organization and biogenesis	0.0496
