# Supplementary material for: Contribution of Transcription Factor Binding Site Motif Variants to Condition-Specific Gene Expression Patterns in Budding Yeast
Source: PLoS One. 2012 Feb 23;7(2):e32274. doi: 10.1371/journal.pone.0032274 (PMC3285675; doi:10.1371/journal.pone.0032274)
Supplement: Table S5 — Secondary transcription factor binding sites correlated with binding site motif variants at nearby functionally variant binding sites. Secondary binding sites have a posterior probability of <0.7 and >0.2. Significant coincidence of secondary binding sites for each other TF with each nucleotide at each functional binding site motif variant position is given according to Fisher's exact test at a level of 0.01. (PDF) [file pone.0032274.s010.pdf]

Table S5. Secondary transcription factor binding sites correlated with variants at nearby functionally variant binding sites. Secondary sites have a posterior probability of <0.7 and >0.2. Significant coincidence of secondary binding sites for each other TF with each nucleotide at each functional variant position is given according to Fisher's exact test at a level of 0.01.

Species	Platform	Transcription Factor Binding Site Family	Position	Variant	Correlated binding site	p value
S. cerevisae	Affymetrix S98	FKH2	1	C	GLN3*	0.0023
S. cerevisae	Affymetrix S98	FKH2	1	C	GZF3	0.0068
S. cerevisae	Affymetrix S98	FKH2	6	C	GLN3*	0.004
S. cerevisae	Affymetrix S98	MCM1	4	G	MSN2	0.0077
S. cerevisae	Affymetrix S98	REB1	2	G	PHO4	0.0051
S. cerevisae	Affymetrix S98	REB1	2	G	YDR026c[¥]	0.0029
S. cerevisae	Affymetrix S98	REB1	2	G	RDS1	0.0051
S. cerevisae	Affymetrix S98	REB1	2	C	SWI5	0.0066
S. cerevisae	Affymetrix S98	REB1	2	C	YDR026c[¥]	9.50E-05
S. cerevisae	Y6.4kv6 cDNA	RAP1	7	T	RDS1	0.0096
S. cerevisae	Y6.4kv6 cDNA	REB1	9	G	MSN2	0.0096
S. paradoxus	Y6.4kv6 cDNA	FKH2	6	C	GLN3	0.0075
S. paradoxus	Y6.4kv6 cDNA	RAP1	9	G	MSN2	0.002
S. paradoxus	Y6.4kv6 cDNA	RAP1	9	G	REB1	0.002
S. paradoxus	Y6.4kv6 cDNA	REB1	9	G	MSN2	0.0041
S. mikatae	Y6.4kv6 cDNA	REB1	9	A	MSN2	0.009
S. mikatae	Y6.4kv6 cDNA	REB1	9	G	MSN2	0.009
S. mikatae	Y6.4kv6 cDNA	SPT15	2	A	ROX1	0.0098
S. mikatae	Y6.4kv6 cDNA	SPT15	2	T	ROX1	0.0063
S. mikatae	Y6.4kv6 cDNA	SWI4	2	T	RFX1	0.0049
S. mikatae	Y6.4kv6 cDNA	SWI4	2	A	STE12mot1	0.0088
S. kudriavzevii	Y6.4kv6 cDNA	FKH2	9	G	NRG1	0.0072
S. kudriavzevii	Y6.4kv6 cDNA	RPN4	2	A	MATalpha2	0.0056

*The Gln3 binding site overlaps with the Fkh2 binding site.
[¥] The YDR026c binding site overlaps with the Reb1 binding site.
